# Bioactive cytochalasans from the desert soil-derived fungus *Chaetomium madrasense* 375 obtained via a chemical engineering strategy

**DOI:** 10.3389/fmicb.2023.1292870

**Published:** 2024-01-31

**Authors:** Qingfeng Guo, Shenyu Shen, Xinyang Wang, Lei Shi, Yuwei Ren, Dandan Li, Zhenhua Yin, Juanjuan Zhang, Baocheng Yang, Xuewei Wang, Gang Ding, Lin Chen

**Affiliations:** ^1^Henan Comprehensive Utilization of Edible and Medicinal Plant Resources Engineering Technology Research Center, Zhengzhou Key Laboratory of Synthetic Biology of Natural Products, Huanghe Science and Technology College, Zhengzhou, China; ^2^School of Chemistry, Xi'an Jiaotong University, Xi'an, China; ^3^School of Pharmacy, Henan University, Kaifeng, China; ^4^State Key Laboratory of Mycology, Institute of Microbiology, Chinese Academy of Sciences, Beijing, China; ^5^Chinese Academy of Medical Science and Union Medical College, Institute of Medicinal Plant Development, Beijing, China

**Keywords:** *Chaetomium madrasense*, diversity-enhanced extracts, cytochalasans, biological effects, chemical engineering strategy

## Abstract

The chemical engineering of natural extracts has emerged as an effective strategy for the production of diverse libraries of chemicals, making it integral to drug discovery. A chemical engineering strategy based on the epoxidation and ring-opening reactions was used to prepare diversity-enhanced extracts of *Chaetomium madrasense* 375. Eleven unnatural cytochalasan derivatives (**1–11**) with unique functional groups, such as amine and isoxazole, were isolated and characterized from these chemically engineered extracts of *C. madrasense* 375. The identification of these new structures was accomplished through comprehensive spectroscopic analysis, supplemented by synthetic considerations. Notably, compounds **5** and **13**–**16** displayed potent phytotoxic effects on *Arabidopsis thaliana*, while compounds **1**, **2**, **5**, **10**, and **12** demonstrated inhibitory activities on LPS-induced NO production in RAW264.7 cells. Among them, compound **1** was found to be able to inhibit the upregulated expression of the inducible nitric oxide synthase (iNOS) protein induced by LPS, while also decreasing the production of pro-inflammatory cytokines (IL-6) and influencing the phosphorylation of p38, ERK1/2, and JNK at 100 μM. Our findings demonstrate that the chemical engineering of natural product extracts can be an efficient technique for the generation of novel bioactive molecules.

## GRAPHICAL ABSTRACT

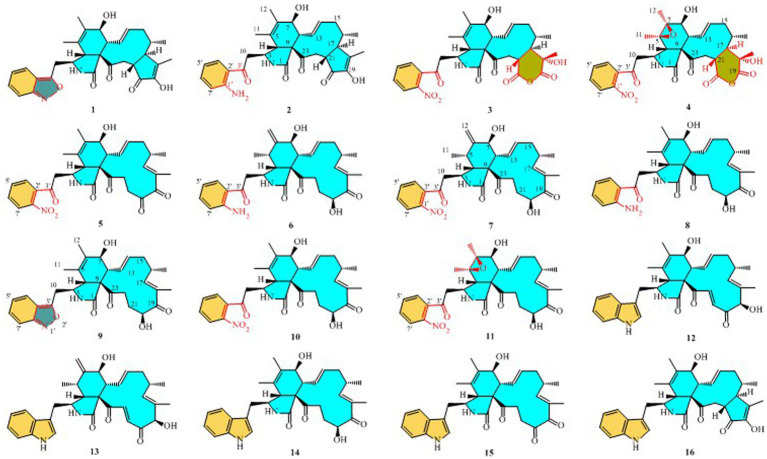

Structures of compounds **1**–**16**.

## Introduction

Natural products (NPs) have traditionally been a vital source of lead molecules for pharmaceutical and agrochemical discoveries due to their structural diversity and various biological activities (Newman and Cragg, 2020). Nevertheless, obtaining novel bioactive structures from natural sources has become challenging due to the extensive scale of chemical screening (Kikuchi et al., 2016). As a result, different strategies have been ushered in to increase the chemical diversity of natural product mixtures, such as robotic separation technologies alongside structural analysis, metabolic engineering, and synthetic biology (Tomohara et al., 2016; Li and Lou, 2018). Over the past decade, a technique known as the “chemical engineering strategy” has emerged; this focuses on the direct chemical derivatization of natural extracts, also known as diversity-enhanced or chemically engineered extracts. This strategy has proven effective in enhancing chemical diversity, enabling the conversion of functional groups and the formation of new carbon–carbon bonds from natural resource extracts to generate a bioactive chemical library (López et al., 2007; Kamauchi et al., 2018; Oshima and Kikuchi, 2018; Ramallo et al., 2018; Solís et al., 2019; He et al., 2021; Sulistyowaty et al., 2021).

The cytochalasans are a large group of fungal secondary metabolites possessing a multi-substituted perhydro-isoindolone moiety fused with a varied macrocyclic ring. More than 400 cytochalasans have been described, some of which have been found to display a wide range of biological activities, such as cytotoxic (Yang et al., 2018), antiviral (Chen et al., 2015), phytotoxic (Li et al., 2014, 2019), antimicrobial (Gao et al., 2019), and nematicidal effects (Skellam, 2017). Cytochalasans have attracted great interest among pharmaceutical chemists and biologists, with extensive investigation having been conducted on their structural diversity, biological activities, biosynthesis, and chemical total synthesis since the first one was reported in 1966 (Skellam, 2017; Long et al., 2018; Wang C. et al., 2019). Nonetheless, the complex and diverse structure of cytochalasans presents challenges in synthesis and separation for their structural modification and transformation. The use of chemically engineered extracts of *C. madrasense* 375 to generate unique biologically active cytochalasan derivatives could provide intriguing models for future medical or pharmacological applications. Recently, we have reported on the isolation of several bioactive cytochalasans from *C. madrasense* 375 obtained from desert soil (Guo et al., 2019, 2021). Kikuchi and his team have previously utilized ring-opening reactions of epoxides to remodel the molecular scaffolds of terpenes extracted from *Curcuma zedoaria*, resulting in a novel range of sesquiterpene-like compounds (Kikuchi et al., 2014). As the primary metabolites of *C. madrasense*, cytochalasans fall into different functional groups, including indole rings, olefins, hydroxyls, and carbonyls. In order to discover novel cytochalasans and explore their biological applications, we implemented a chemical engineering strategy to prepare a diversity-enhanced extract of *C. madrasense*. The preparation of this comprised two stages: initially, the extract underwent reaction with 1,1,1-trifluoroacetone and oxone to produce epoxide extracts; subsequently, lanthanum (III) trifluoromethanesulfonate was used as a Lewis acid catalyst for the ring-opening reaction. Ultimately, eleven novel or new molecular skeletons of cytochalasan derivatives (**1**–**11**) were obtained from the diversity-enhanced extracts of *C. madrasense* 375. This paper presents details of the isolation of these compounds, elucidation on their structure, their bioactivities, and plausible synthesis pathways.

## Materials and methods

### General experimental procedures

Ultraviolet (UV) measurements were taken using a NanoDrop 2000 ultra-micro spectrophotometer. CD spectra were recorded using a JASCO J-815 spectropolarimeter. NMR spectra were determined using a Bruker Avance III 400 NMR spectrometer (^1^H, 400 MHz, ^13^C, 100 MHz). Low-resolution electrospray ionization mass spectrometry (ESIMS) data were collected on an Agilent1200 series LC/MS system, and high-resolution ESIMS (HRESIMS) data were acquired on an Agilent 6250TOF LC/MS. Semipreparative HPLC was performed on a Calmflowplus system that was equipped with a 50D UV–vis Detector (Lumiere Tech Ltd) and a YMC Pack ODS-A column (10 mm × 250 mm 5 μm, Japan). Silica gel (200–300 mesh, Qingdao Marine Chemical Factory, Qingdao, China), ODS (YMC, Japan), and Sephadex LH-20 (GE Healthcare BioSciences AB, Sweden) were used as packing materials for column chromatography. All chemicals used in the study were of analytical grade.

### Chemicals and reagents

The reagents of 1,1,1-trifluoroacetone, oxone, and lanthanum (III) trifluoromethanesulfonate were purchased from Aladdin Holdings Group Co., Ltd. Murine macrophage RAW 264.7 cells were purchased from the Shanghai Institutes for Biological Sciences in DMEM (high glucose). LPS was obtained from Sigma-Aldrich (St. Louis, MO, United States). The nitric oxide kit was obtained from Nanjing Jancheng Bioengineering Institute (Nanjing, China). IL-6 ELISA kits were obtained from Beijing 4A Biotech Co., Ltd. (Beijing, China). Antibodies were obtained from Cell Signaling (Beverly, MA, United States).

### Fungal culture

Following a previously reported procedure (Guo et al., 2021), *Chaetomium madrasense* 375 (CCTCC M2019517 CLC375) was isolated from a soil sample collected in Hotan city, located in Sinkiang province, People’s Republic of China. The identification of the fungus was based on an examination of the internal transcribed spacer (ITS) sequence of its rDNA, the beta-tubulin encoding gene from its genomic DNA, and its morphological characteristics. The strain’s sequences were deposited in GenBank as KP269060.1. The strain was cultured on potato dextrose agar (PDA) at 25°C for 7 days and transferred into each of a total of 300 Erlenmeyer flasks (500 mL) containing autoclaved rice medium (60 g of rice and 100 mL of distilled water). All flasks were incubated at 25°C for 35 days.

### Preparation of the chemically engineered extract of *Chaetomium madrasense* 375

The fungal cultures were extracted with ethyl acetate (EtOAc) (200 mL × 3 for each flask), and the solvent was combined and evaporated *in vacuo* under reduced pressure to leave a residue. The residue was then suspended in H_2_O and extracted sequentially with petroleum ether (EtOAc). The EtOAc extract (77 g) was packed with cytochalasans based on the analysis in the TLC and LC–MS experiments. The EtoAc extract (20 g) was dissolved in acetonitrile (1200 mL) and water (500 mL). 1,1,1-trifluoroacetone (20 mL), sodium bicarbonate (28 g), and potassium monopersulfate (oxone) (40 g) were added to this solution sequentially at 0°C, and the mixture was kept at 0°C for 3 h. Oxone (40 g) was then added, followed by stirring for another 3 h at 0°C. The reaction mixture was poured into water, extracted three times with ethyl acetate, combined with the organic solvent, washed with water and brine, dried with sodium sulfate, and evaporated to produce the reaction residue (23 g). Lanthanum (III) trifluoromesylate (La (OTf)_3_) and 2,6-di-tert-butylpyridine (30 mL) were added to a solution of the residue (20 g) in 1,2-dichloroethane (320 mL) at room temperature. After 15 h of refluxation, lanthanum (III) trifluorometasulfonate (La (OTf)_3_) (10 g) was added and the mixture refluxated for an additional 24 h. The reaction mixture was then poured into 0.5 M hydrochloric acid and extracted three times with EtOAc to merge the organic layer, washed with saturated sodium bicarbonate solution and saline, dried with sodium sulfate, and evaporated to obtain the diversity-enhanced extract of *Chaetomium madrasense* 375 (22 g).

### Isolation of compounds 1–11 and structural characterization

The diversity-enhanced extracts were subjected to an ODS column chromatography gradient elution with CH_3_OH in H_2_O (100:0, 80:20, 40:60, 20:80, 0:100, v: v) to give four fractions (A–D). Fraction B was subdivided into five subfractions (B-1–B-5) through Sephadex LH-20 chromatography, employing methanol as the mobile phase. Subfractions B-1, B-2, B-4, and B-5 were purified using repeated HPLC, successfully yielding **1** (5 mg), **2** (4 mg), **6** (3 mg), and **7** (2 mg), respectively. Next, fraction C was separated via Sephadex LH-20 column chromatography (CC), eluting with CH_3_OH to afford three subfractions (C-1 − C-3). Subfraction C-2 was purified via semi-prepared HPLC eluted with CH_3_CN-H_2_O (62.5, 37.5) to give **8** (6 mg, *t*_R_ = 35.1 min) and **9** (3 mg, *t*_R_ = 22.9 min). Subfraction C-3 was purified via semi-prepared HPLC with CH_3_CN/H_2_O (v/v, 40:60, 2 mL/min) to yield **10** (3 mg, *t*_R_ = 29.1 min) and **11** (6 mg, *t*_R_ = 30.7 min). Fraction D was subjected to separation via Sephadex LH-20 CC, using methanol as the eluent, to give three subfractions (D-1–D-3). Among these, D-2 was purified via semi-prepared HPLC with CH_3_CN/H_2_O (v/v, 40:60, 2 mL/min) to afford **3** (2.8 mg, *t*_R_ = 27.4 min) and **4** (1.9 mg, *t*_R_ = 29.7 min). D-3 was purified via semi-prepared HPLC with CH_3_CN/H_2_O (v/v, 53:47, 2 mL/min) to yield **5** (7 mg, *t*_R_ = 19.4 min).

Chaetogobosin V_b_-a (**1**): white amorphous powder; UV (MeOH) λmax (log ε) 315 (0.64), 269 (0.84); ECD (MeOH) λmax (Δ*ε*) = 310 nm (+12.7), 263 (−26.5); for ^1^H NMR (400 MHz) and ^13^C NMR (100 MHz) data, see Tables 1, 2; HR-ESI-MS m/z 531.2482 [M + H]^+^ (calcd for C_31_H_35_N_2_O_6_, 531.2495[M + H]^+^).

**Table 1:** ^1^H (400 MHz) NMR data for compounds 1–5.

NO	**1** ^a^	**2** ^a^	**3** ^b^	**4** ^b^	**5** ^a^
4′	7.20 (1H, d, *J* = 8.4)	7.51 (1H, d, *J* = 7.7)	7.67 (1H, d, *J* = 7.5)	7.62 (1H, d, *J* = 7.1)	7.34 (1H, d, *J* = 7.6)
5′	6.82 (1H, t, *J* = 6.8)	6.62 (1H, t, *J* = 8.0)	7.85 (1H, t, *J* = 7.0)	7.84 (1H, t, *J* = 7.3)	7.78 (1H, t, *J* = 7.5)
6′	7.18 (1H, t, *J* = 7.7)	7.23, (1H,d, *J* = 7.7)	7.78 (1H, t, *J* = 7.8)	7.77 (1H, t, *J* = 7.7)	7.67 (1H, t, *J* = 7.8)
7′	7.42 (1H, d, *J* = 9.0)	6.60 (1H, d, *J* = 8.0)	8.11 (1H, d, *J* = 8.8)	8.11 (1H,d, *J* = 8.0)	8.17 (1H,d, *J* = 8.3)
2	7.50 (1H, s)	6.68 (1H, s)	8.10 (1H, s)	8.34 (1H, s)	6.35 (1H, s)
3	3.76 (1H, t, *J* = 7.0)	3.81 (1H, d, *J* = 9.9)	3.75 (1H, t, *J* = 6.4)	4.00 (1H, t, *J* = 7.0)	3.92 (1H, t, *J* = 12.0)
4	3.23 (1H, s)	3.25 (1H, s)	3.18 (1H, s)	3.00 (1H, s)	2.88 (1H, s)
7	4.18 (1H, d, *J* = 9.7)	4.23 (1H, d, *J* = 9.0)	3.90 (1H, m)	3.69 (1H, dd, *J* = 10.2, 7.3)	3.94 (1H, m)
8	2.29 (1H, m)	2.35 (1H, t, *J* = 10.4)	2.02 (1H, m)	2.39 (1H, m)	2.24 (1H, t, *J* = 10.0)
10	3.04 (2H, m)	3.11 (1H, d, *J* = 17.1), 2.76 (1H, dd, *J* = 17.1, 10.6)	2.93 (2H, m)	2.90 (2H, m)	2.84 (1H, m) 2.61 (1H, dd, *J* = 18.3, 10.6)
11	1.43 (3H, s)	1.74 (3H, s)	1.75 (3H, s)	1.48 (3H, s)	1.73 (3H, s)
12	1.67 (3H, s)	1.75 (3H, s)	1.63 (3H, s)	1.31 (3H, s)	1.75 (3H, s)
13	6.22 (1H, dd, *J* = 15.3, 10.2)	6.27 (1H, dd *J* = 15.3, 10.3)	6.02 (1H, dd, *J* = 15.3, 10.3)	6.08 (1H, dd, *J* = 15.7, 10.5)	6.27 (1H, dd, *J* = 15.3, 10.3)
14	5.40 (1H, m)	5.43 (1H, m)	4.94 (1H, m)	5.01 (1H, m)	5.23 (1H, m)
15	2.33 (1H, m), 2.05 (1H, m)	2.33 (1H, m), 2.02 (1H, m)	2.27 (1H, m), 1.95 (1H, m)	2.32 (1H, m), 1.96 (1H, m)	2.46 (1H, m), 2.04 (1H, m)
16	1.63 (1H, m)	1.63 (1H, m)	1.91 (1H, m)	1.74 (1H, m)	2.76 (1H, m)
17	2.21 (1H, d, *J* = 7.2)	2.20 (1H, d, *J* = 9.3)	1.68 (1H, m)	2.47 (1H, m)	6.05 (1H, d, *J* = 10.0)
21	2.43 (1H, m)	2.41 (1H, m)	3.33 (1H, m)	2.94 (1H, m)	3.31(1H, m), 3.01(1H, m)
22	3.52 (1H, m), 2.01 (1H, m)	3.46 (1H, m), 2.04 (1H, m)	3.48 (1H, m), 2.36 (1H, m)	3.53 (1H, m), 2.33 (1H, m)	3.19 (1H, m), 2.38 (1H, m)
16-Me	1.08 (3H, d, *J* = 6.8)	1.05 (3H d, *J* = 6.4)	1.07 (3H, d, *J* = 5.8)	0.86 (3H, d, *J* = 6.0)	1.08 (3H, d, *J* = 6.7)
18-Me	2.09 (3H, s)	2.04 (3H, s)	1.43 (3H, s)	1.32 (3H, s)	1.80 (3H, s)

a Measured in CDCl_3._

b Measured in DMSO-_*d6*._

**Table 2:** ^13^C (100 MHz) NMR data for compounds 1–5.

NO	**1** ^a^	**2** ^a^	**3** ^b^	**4** ^b^	**5** ^a^
1′	156.8, C	150.2, C	146.4, C	145.7, C	145.0, C
1′a	156.8, C				
2′		117.2, C	135.6, C	134.8, C	136.8, C
3′	163.8, C	199.6, C	199.8, C	199.1, C	200.3, C
3′a	116.4, C				
4′	119.4, CH	131.0, CH	128.5, CH	127.8, CH	127.1, CH
5′	123.9, CH	116.2, CH	134.9, CH	134.2, CH	135.0, CH
6′	131.1, CH	135.0, CH	132.5, CH	132.0, CH	131.2, CH
7′	114.8, CH	117.4, CH	125.0, CH	124.4, CH	124.8, CH
1	175.6, C	174.3, C	173.7, C	172.8, C	174.4, C
3	55.8, CH	53.4, CH	52.1, CH	49.5, CH	52.6, CH
4	49.8, CH	49.9, CH	49.3, CH	46.0, CH	51.2, CH
5	125.7, C	126.5, C	125.8, C	63.0, C	133.2, C
6	133.6, C	132.9, C	134.8, C	65.1, C	125.5, C
7	69.5, CH	69.7, CH	68.7, CH	68.9, CH	68.0, CH
8	53.4, CH	53.6, CH	52.7, CH	46.2, CH	52.1, CH
9	65.1, C	65.2, C	64.2, C	64.6, C	60.9, C
10	33, CH_2_	44.9, CH_2_	47.6, CH_2_	47.0, CH_2_	49.0, CH_2_
11	17.3, CH_3_	17.7, CH_3_	17.5, CH_3_	20.1, CH_3_	14.3, CH_3_
12	14.3, CH_3_	14.2, CH_3_	15.1, CH_3_	14.6, CH_3_	17.5, CH_3_
13	128.4, CH	128.8, CH	131.4, CH	130.3, CH	126.7, CH
14	136.6, CH	136.2, CH	132.6, CH	132.3, CH	137.3, CH
15	44.3, CH_2_	44.3, CH_2_	43.8, CH_2_	42.1, CH_2_	40.4, CH_2_
16	43.0, CH	42.8, CH	37.6, CH	35.0, CH	33.5, CH
17	54.9, CH	54.8, CH	59.9, CH	53.4, CH	155.7, CH
18	149.3, C	147.9, C	89.0, C	87.3, C	132.8, C
19	149.6, C	148.8, C	172.8, C	172.0, C	196.7, C
20	204.2, C	203.5, C	181.1, C	179.4, C	204.9, C
21	49.6, CH	49.9, CH	41.9, CH	38.3, CH	37.9, CH_2_
22	43.5, CH_2_	43.2 CH_2_	47.2 CH_2_	45.2 CH_2_	32.9 CH_2_
23	209.5, C	210.0, C	209.7, C	208.2, C	208.1, C
16-Me	21.8, CH_3_	21.7, CH_3_	23.0, CH_3_	21.2, CH_3_	19.5, CH_3_
18-Me	17.0, CH_3_	16.7, CH_3_	26.2, CH_3_	18.1, CH_3_	10.3, CH_3_

a Measured in CDCl_3._

b Measured in DMSO-_*d6*._

Chaetogobosin V_b_-b (**2**): white amorphous powder; UV (MeOH) λmax (log ε) 368 (0.40), 261 (0.96), 228 (1.59) nm; ECD (MeOH) λmax (Δ*ε*) = 329 (+5.5), 296 (−0.35), 276 (+0.35), 249 (−5.6), 207 (+21.7) nm; for ^1^H NMR (400 MHz) and ^13^C NMR (100 MHz) data, see Tables 1, 2; HR-ESI-MS m/z 533.2640 [M + H]^+^ (calcd for C_31_H_37_N_2_O_6_, 533.2652 [M + H]^+^).

Chaetogobosin V_b_-c (**3**): white amorphous powder; UV (MeOH) λmax (log ε) 249 (0.88), 222 (1.32), 217 (1.48) nm; ECD (MeOH) λmax (Δ*ε*) = 331 (+3.06), 293 (−5.42), 257 (−4.06), 204 (+38.40) nm; for ^1^H NMR (400 MHz) and ^13^C NMR (100 MHz) data, see Tables 1, 2; HR-ESI-MS m/z 595.2297 [M + H]^+^ (calcd for C_31_H_35_N_2_O_10_, 595.2286 [M + H]^+^).

Chaetogobosin V_b_-d (**4**): white amorphous powder; UV (MeOH) λmax (log ε) 223 (1.33), 219 (1.41), 205 (0.44) nm; ECD (MeOH) λmax (Δ*ε*) = 331 (+4.71), 296 (−4.39), 246 (−6.84), 203 (+42.20) nm; for ^1^H NMR (400 MHz) and ^13^C NMR (100 MHz) data, see Tables 1, 2; HR-ESI-MS m/z 611.2246 [M + H]^+^ (calcd for C_31_H_35_N_2_O_11_, 611.2135 [M + H]^+^).

Chaetogobosin G-a (**5**): white amorphous powder; UV (MeOH) λmax (log ε) 332 (1.26), 299 (1.51), 248 (1.54) nm; for ^1^H NMR (400 MHz) and ^13^C NMR (100 MHz) data, see Tables 1, 2; HR-ESI-MS m/z 585.2208 [M + Na]^+^ (calcd for C_31_H_34_N_2_NaO_8_, 585.2207[M + Na]^+^).

Chaetogobosin D-a (**6**): white amorphous powder; UV (MeOH) λmax (log ε) 368 (0.39), 230 (1.91) nm; for ^1^H NMR (400 MHz) and ^13^C NMR (100 MHz) data, see Tables 3, 4; HR-ESI-MS m/z 535.2808 [M + H]^+^ (calcd for C_31_H_39_N_2_O_6_, 535.2803 [M + H]^+^).

**Table 3:** ^1^H (400 MHz) NMR data for compounds 6–11.

NO	**6** ^a^	**7** ^a^	**8** ^a^	**9** ^a^	**10** ^a^	**11** ^b^
4′	7.57 (1H, d, *J* = 8.2)	7.35 (1H, d, *J* = 7.5)	7.51(1H, d, *J* = 8.4)	7.41(1H, d, *J* = 8.8)	7.37 (1H, d, *J* = 7.4)	7.67 (1H, d, *J* = 7.5)
5′	6.65 (1H, t, *J* = 6.8)	7.77 (1H, t, *J* = 7.5)	6.65 (1H, t, *J* = 6.8)	7.00 (1H, t, *J* = 7.5)	7.78 (1H, t, *J* = 7.4)	7.90 (1H, t, *J* = 7.4)
6′	7.29 (1H, t, 7.1)	7.65 (1H, t, *J* = 7.7)	7.30 (1H, t, *J* = 7.5)	7.28 (1H, t, *J* = 7.7)	7.65 (1H, t, *J* = 7.7)	7.78 (1H, t, *J* = 7.8)
7′	6.66 (1H, t, *J* = 7.8)	8.17 (1H, d, *J* = 8.2)	6.66 (1H, d, *J* = 8.3)	7.49 (1H, d, *J* = 9.1)	8.15 (1H, d, *J* = 8.2)	8.15 (1H, d, *J* = 8.1)
2	6.57 (1H, s)	6.42 (1H, s)	6.42 (1H, s)	6.75 (1H, s)	6.48 (1H, s)	8.2 (1H, s)
3	3.63 (1H, d, *J* = 9.9)	3.74 (1H, m)	3.79 (1H, d, *J* = 10.5)	3.77 (1H, t, *J* = 7.3)	3.95 (1H, m)	3.96 (1H, t, *J* = 6.5)
4	2.72 (1H, t, *J* = 4.0)	2.64 (1H, m)	3.07 (1H, s)	3.07 (1H, s)	3.04 (1H, s)	2.79 (1H, s)
5	2.83 (1H, m)	2.79 (1H, m)				
7	4.02 (1H, d, *J* = 10.4)	4.0 (1H, d, *J* = 10.0)	3.98(1H, d, *J* = 9.1)	3.90 (1H, d, *J* = 9.2)	3.97(1H, m)	3.44 (1H, m)
8	2.65 (1H, t, *J* = 10.1)	2.60 (1H, d, *J* = 9.9)	2.19 (1H, m)	2.16 (1H, m)	2.18 (1H, m)	2.44 (1H, t, *J* = 10.2)
10	3.16 (1H, m), 2.96 (1H, m)	2.97 (1H, m), 2.72 (1H, m)	3.11(1H, m), 2.81 (1H, m)	3.14 (2H, m)	2.93 (1H, m), 2.68 (1H, m)	2.88 (2H, m)
11	1.10 (3H, d, *J* = 6.9)	1.12 (3H, d, *J* = 6.6)	1.71 (3H, s)	1.42 (3H, s)	1.79 (3H, s)	1.45 (3H, s)
12	5.42 (1H, s), 5.16 (1H, s)	5.43 (1H, s), 5.21 (1H, s)	1.75 (3H, s)	1.68 (3H, s)	1.77 (3H, s)	1.30 (3H, s)
13	6.36 (1H, dd, *J* = 15.3, 9.3)	6.42 (1H, m)	6.50 (1H, dd, *J* = 15.4, 10.0)	6.46 (1H, dd, *J* = 14.0, 10.4)	6.52 (1H, m)	6.15 (1H, m)
14	5.41(1H, m)	5.41 (1H, m)	5.37 (1H, m)	5.35 (1H, m)	5.37 (1H, m)	5.09 (1H, m)
15	2.52 (1H, d, *J* = 10.1), 2.17 (1H, m)	2.52 (1H, d, *J* = 10.1), 2.17 (1H, m)	2.49 (1H, m), 2.15 (1H, m)	2.47 (1H, m), 2.14, (1H, m)	2.50 (1H, m), 2.16 (1H, m)	2.36 (1H, d, *J* = 13.4), 1.96 (1H, m)
16	2.85 (1H, m)	2.84 (1H, m)	2.78 (1H, m)	2.77 (1H, m)	2.79 (1H, m)	2.73 (1H, m)
17	6.23 (1H, d, *J* = 9.4)	6.23 (1H, d, *J* = 9.9)	6.17(1H, d, *J* = 9.2)	6.20 (1H, d, *J* = 8.8)	6.17(1H, d, *J* = 9.0)	6.21(1H, d, *J* = 9.4)
20	4.77 (1H, t, *J* = 5.3)	4.78 (1H, brs)	4.71 (1H, m)	4.78 (1H, brs)	4.72 (1H, t, *J* = 5.6)	4.68 (1H, t, *J* = 5.3)
21	1.91 (2H, m)	1.89 (2H, m)	1.89 (1H, m),1.83 (1H, m)	1.92 (2H, m)	1.78 (2H, m)	1.51 (2H, m)
22	2.92 (1H, m), 2.84 (1H, m)	2.89 (2H, m)	3.00 (1H, m), 2.89 (1H, m)	3.06 (1H, m), 2.96 (1H, m)	2.96 (2H, m)	2.83 (1H, m), 2.58 (1H, m)
16-Me	1.09 (3H d, *J* = 6.4)	1.08 (3H d, *J* = 6.4)	1.07 (3H d, *J* = 6.6)	1.05 (3H d, *J* = 6.6)	1.07(3H d, *J* = 6.6)	0.98 (3H d, *J* = 6.6)
18-Me	1.87 (3H, s)	1.86 (3H, s)	1.85 (3H, s)	1.83 (3H, s)	1.83 (3H, s)	1.70 (3H, s)

a *Measured in CDCl*_*3*._

b *Measured in DMSO-_d6._*

**Table 4:** ^13^C (100 MHz) NMR data for compounds 6–11.

NO	**6** ^a^	**7** ^a^	**8** ^a^	**9** ^a^	**10** ^a^	**11** ^b^
1′	150.5, C	145.3, C	150.5, C		145.3, C	145.4, C
1′a				157.0, C		
2′	117.1, C	137.0, C	117.2, C		136.9, C	135.5, C
3′	199.9, C	201.3, C	199.6, C	164.0, C	200.8, C	199.7, C
3′a				116.7, C		
4′	130.7, CH	127.0, CH	130.8, CH	119.3, CH	127.1, CH	127.7, CH
5′	116.1, CH	134.7, CH	116.2, CH	124.3, CH	134.9, CH	134.5, CH
6′	135.1, CH	131.0, CH	135.1, CH	131.3, CH	131.0, CH	131.7, CH
7′	117.5, CH	124.7, CH	117.5, CH	115.0, CH	124.7, CH	124.4, CH
1	173.4, C	173.2, C	174.0, C	174.7, C	174.1, C	173.2, C
3	48.2, CH	48.0, CH	53.4, CH	55.9, CH	52.9, CH	49.1, CH
4	47.2, CH	47.3, CH	49.8, CH	49.6, CH	49.8, CH	46.7, CH
5	32.1, CH	32.0, CH	126.1, C	125.3, C	125.8, C	62.7, C
6	147.5, C	147.2, C	132.3, C	133.1, C	132.7, C	65.0, C
7	69.9, CH	69.9, CH	68.4, CH	68.2, CH	68.3, CH	68.3, CH
8	49.3, CH	49.2, CH	51.9, CH	51.6, CH	51.5, CH	44.1, CH
9	62.5, C	62.3, C	62.0, C	61.9, C	61.9, C	63.1, C
10	46.8, CH_2_	50.1, CH_2_	45.2, CH_2_	33.6, CH_2_	49.1, CH_2_	47.5, CH_2_
11	19.9, CH_3_	19.9, CH_3_	17.6, CH_3_	17.2, CH_3_	17.6, CH_3_	20.1, CH_3_
12	114.9, CH_2_	115.2, CH_3_	14.2, CH_3_	14.3, CH_3_	14.3, CH_3_	14.7, CH_3_
13	128.9, CH	129.0, CH	128.8, CH	128.4, CH	128.6, CH	127.3, CH
14	135.7, CH	135.7, CH	136.1, CH	136.4, CH	136.3, CH	134.2, CH
15	41.0, CH_2_	41.0, CH_2_	41.2, CH_2_	41.2, CH_2_	41.1, CH_2_	40.4, CH_2_
16	33.7, CH	33.8, CH	33.4, CH	33.3, CH	33.4, CH	32.8, CH
17	149.4, CH	149.6, CH	149.2, CH	149.5, CH	149.4, CH	148.4, CH
18	134.6, C	134.8, C	134.8, C	134.8, C	134.8, C	135.0, C
19	203.4, C	203.5, C	203.6, C	203.8, C	203.8, C	204.1, C
20	71.4, CH	71.3, CH	71.5, CH	71.4, CH	71.2, CH	69.5, CH
21	31.5, CH_2_	31.6, CH_2_	31.6, CH_2_	31.8, CH_2_	31.6, CH_2_	30.3, CH_2_
22	38.1, CH_2_	38.4, CH_2_	38.1, CH_2_	37.8, CH_2_	37.6, CH_2_	35.3, CH_2_
23	207.9, C	207.7, C	208.8, C	208.8, C	208.9, C	209.1, C
16-Me	13.9, CH_3_	13.8, CH_3_	20.0, CH_3_	19.9, CH_3_	19.9, CH_3_	19.5, CH_3_
18-Me	12.3, CH_3_	12.2, CH_3_	12.3, CH_3_	12.3, CH_3_	12.3, CH_3_	11.8, CH_3_

a Measured in CDCl_3_.

b Measured in DMSO-*_d6_*.

Chaetogobosin D-b (**7**): white amorphous powder; UV (MeOH) λmax (log ε) 366 (0.19), 243 (1.74), 230 (2.02) nm; for ^1^H NMR (400 MHz) and ^13^C NMR (100 MHz) data, see Tables 3, 4; HR-ESI-MS m/z 565.2533 [M + H]^+^ (calcd for C_31_H_37_N_2_O_8_, 565.2544 [M + H]^+^).

Chaetogobosin E-a (**8**): white amorphous powder; UV (MeOH) λmax (log ε) 368 (0.53), 230 (2.53), 204 (1.65) nm; for ^1^H NMR (400 MHz) and ^13^C NMR (100 MHz) data, see Tables 3, 4; HR-ESI-MS m/z 535.2814 [M + H]^+^ (calcd for C_31_H_39_N_2_O_6_, 535.2803[M + H]^+^).

Chaetogobosin E-b (**9**): white amorphous powder; UV (MeOH) λmax (log ε) 325 (1.80), 308 (1.91), 248 (2.13), 230 (1.60) nm; for ^1^H NMR (400 MHz) and ^13^C NMR (100 MHz) data, see Tables 3, 4; HR-ESI-MS m/z 533.2635 [M + H]^+^ (calcd for C_31_H_37_N_2_O_6_, 533.2646 [M + H]^+^).

Chaetogobosin E-c (**10**): white amorphous powder; UV (MeOH) λmax (log ε) 229 (0.57), 218 (0.64), 205 (0.71) nm; for ^1^H NMR (400 MHz) and ^13^C NMR (100 MHz) data, see Tables 3, 4; HR-ESI-MS m/z 587.2371 [M + Na]^+^ (calcd for C_31_H_36_N_2_NaO_8_, 587.2364 [M + Na]^+^).

Chaetogobosin E-d (**11**): white amorphous powder; UV (MeOH) λmax (log ε) 235 (0.14), 217 (0.17), 208 (0.19) nm; ECD (MeOH) λmax (Δ*ε*) = 288(−1.49), 261(+0.41), 230 (−14.26), 203 (+23.54) nm; for ^1^H NMR (400 MHz) and ^13^C NMR (100 MHz) data, see Tables 3, 4; HR-ESI-MS m/z 581.2495 [M + H]^+^ (calcd for C_31_H_37_N_2_O_9_, 581.2494 [M + H]^+^).

### Seedling growth test

The seedling growth test procedures were drawn from the methods described in Li et al. (2020) and Zhang et al. (2018).

### Cell culture and cell viability analysis

The cell culture and cell viability methods were implemented according to the procedures previously reported by Zhang et al. (2021).

### Quantification of nitric oxide (NO) production

Nitric oxide secretion in RAW264.7 cells was quantified using the nitric acid reduction method. The test compounds were administered in varying concentrations (6.25, 12.5, 25, 50, or 100 μM) to the experimental group for a duration of 1 h, followed by LPS (1 μg/mL) for 24 h at a temperature of 37°C. In contrast, the control group was exclusively exposed to DMEM medium. Following treatment, culture supernatants were harvested and NO levels were determined in accordance with the manufacturer’s guidelines.

### Detection of IL-6

The RAW264.7 cells were treated with various concentrations (6.25, 12.5, 25, 50, or 100 μM) of compounds **1** and **12** for 1 h and with 1 μg/mL LPS for 12 h in sequence. The cellular supernatants were then collected, and the presence of IL-6 was determined using an ELISA kit.

### Western blot analysis

Western blot analysis was performed as described previously (Zhang et al., 2021). According to the manufacturer’s instructions, the total protein/nucleoprotein was extracted using the total protein/nucleoprotein extraction kit. The protein concentration was quantified via the BCA protein assay kit and equalized before loading. Equivalent protein samples (30 μg) were separated via 8% SDS-PAGE electrophoresis and transferred to a polyvinylidene difluoride (PVDF) membrane. The membrane (Solarbio Millipore, Beijing, China) was blocked with 5% skimmed milk for 2 h, after which it was incubated with primary antibodies at 4°C overnight. Subsequent to a rinse with Tween 20 (TBST), the protein was exposed to HRP-conjugated secondary antibody for 2 h and visualized using enhanced chemiluminescence (ECL) Plus hypersensitive luminescence solution. Image analysis was performed using the Imag J software package.

### Statistical analysis

The data are presented in the form mean ± standard deviation (SD) based on three independent experiments (*n* = 3). Statistical significance was determined using the paired Student’s *t*-test. A *p*-value of less than 0.05 was deemed to indicate statistical significance.

### Quantum chemical calculations

All density functional theory (DFT) calculations were performed using Gaussian 16 Rev. A.03. Precise structure optimization was carried out at the B3LYP/6-31G(d) level of theory with the SMD solvation model (MeOH as solvent). For the calculation of electronic circular dichroism (ECD) spectra, the time-dependent density-functional theory (TD-DFT) method was utilized at the same level. A total of 50 excited states were computed to determine the rotatory strengths (Guo et al., 2019, 2021).

## Results and discussion

### Structure elucidation

**Figure 1:**
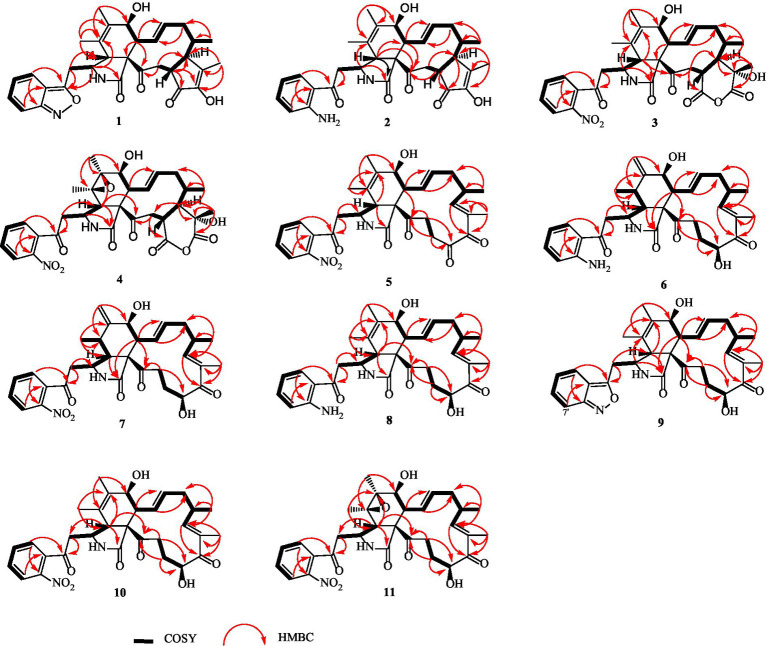
^1^H-^1^H COSY, selected HMBC correlations of compounds **1**–**11**.

**Figure 2:**
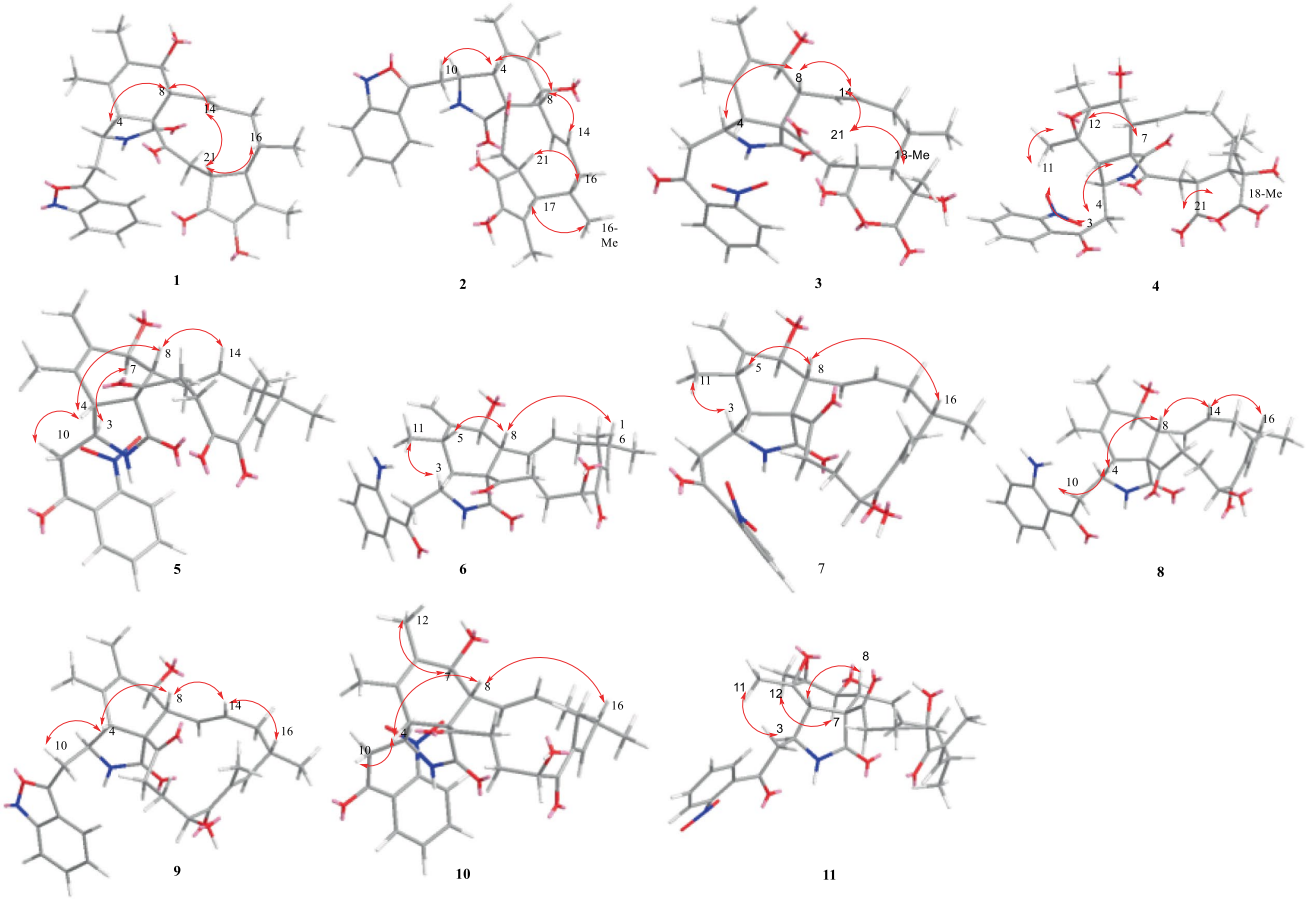
Key NOESY correlations of compounds **1**–**11**.

**SCHEME 1:**
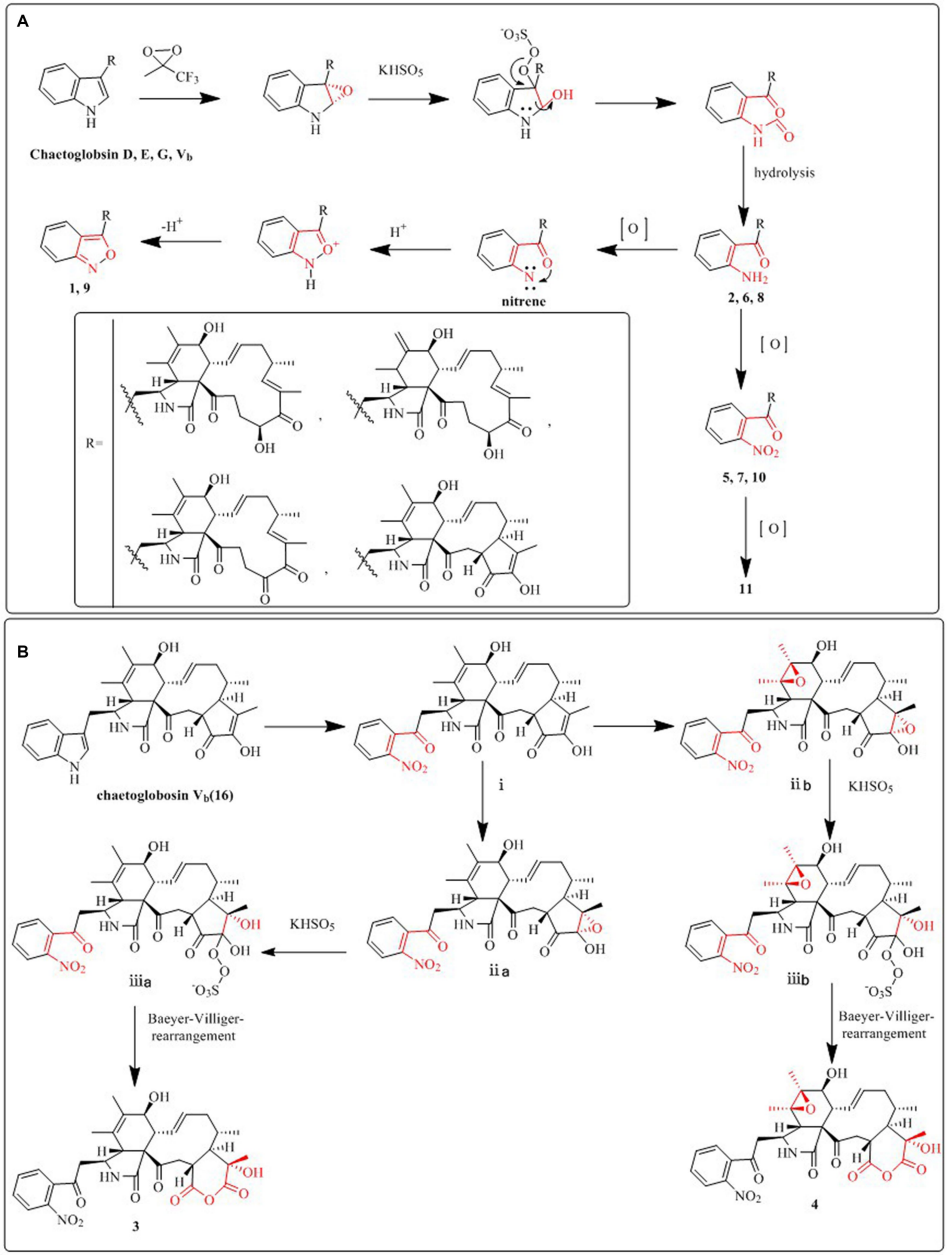
Plausible synthesis pathway of **1**, **2**, **5**–**11 (A)**, and **3**, **4 (B)**.

**Figure 3:**
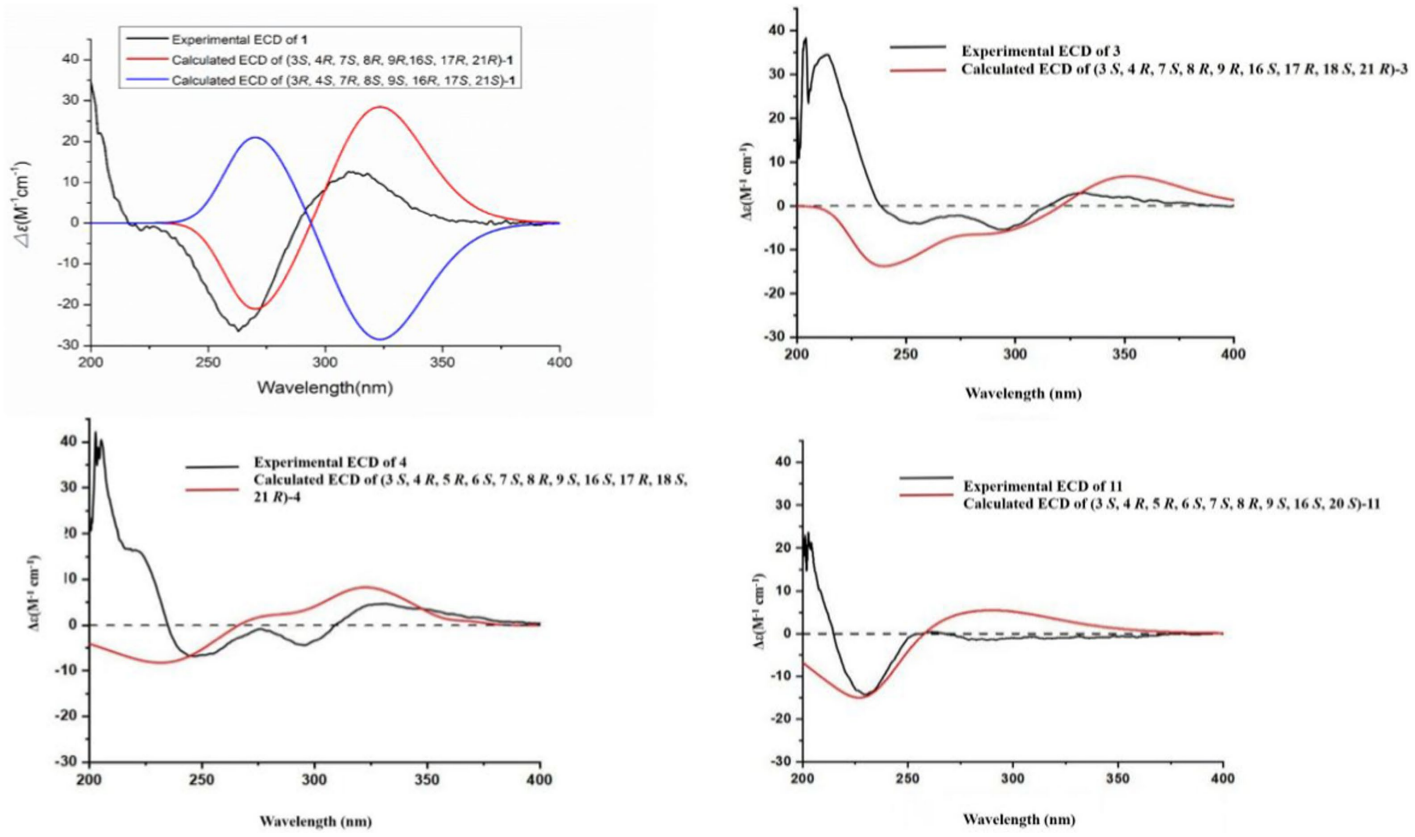
Experimental and calculated ECD spectra of compounds **1**, **3**, **4** and **11**.

Compound **1** was a white amorphous powder with a molecular formula of C_31_H_34_N_2_O_6_ according to the HRESIMS peak at *m/z* 531.2482 [M+ H]^+^. The 1D NMR (Tables 1, 2) and HSQC spectra of **1** (Supplementary Figure S4) indicated the presence of 31 carbon signals, including four methyls, three methylenes, thirteen methines (seven sp^3^, six sp^2^ methines), and eleven quaternary carbons (including three carbonyl carbons), indicating that **1** was likely a chaetoglobosin-type cytochalasan. Further examination of the 1D and 2D NMR spectra of **1** (Supplementary Figures S1–S6) revealed a similarity in the perhydro-isoindolone and macrocyclic ring moiety to that reported for chaetogobosin V_b_ (**16**) (Xue et al., 2012). However, a methine from the indole moiety in chaetogobosin V_b_ (**16**) was replaced by an oxygen atom attached to the 1′-N and C-3′ that formed an isoxazole ring in **1**. The speculation was confirmed by the HMBC (Figure 1) correlations from H-4′ and H-10 to C-3′ (*δ*_C_ 163.8), as well as the molecular formula of **1**. Compound **1** lacks one carbon atom and has one more oxygen atom than chaetogobosin V_b_. The relative configuration of compound **1** was determined through its NOESY spectrum (Figure 2) and was found to be identical to that of chaetogobosin V_b_ (**16**). It is assumed that compound **1** was produced through sequential reactions starting from chaetogobosin V_b_ (Scheme 1). Considering that the stereocenters of **1** are located in the perhydro-isoindolone and macrocyclic ring moiety, and the stereochemistry is retained throughout these reactions, the absolute configuration is assumed to be the same as that of chaetogobosin V_b_ (**16**). To confirm this hypothesis, ECD experiments and ECD simulation of **1** (Figure 3) were both performed. The results indicated that the calculated ECD curve of (3*S*, 4*R*, 7S, 8*R*, 9*R*, 16*S*, 17*R*, 21*R*)-1 matched well with the experimental curve and was consistent with the absolute configuration of chaetogobosin V_b_ (**16**). Thus, the stereochemistry of compound **1** was elucidated. This is the first report of a cytochalasan containing a rare benzisoxazole.

**Figure 4:**
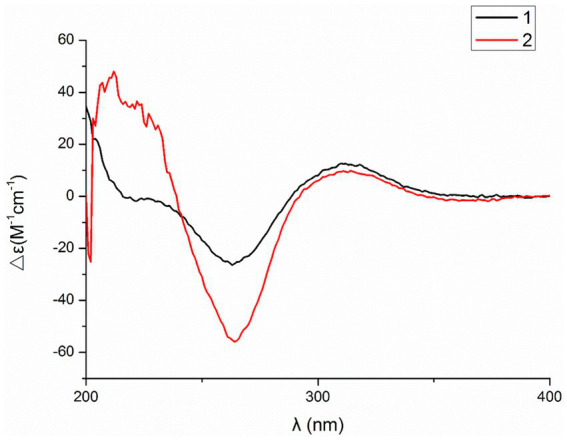
Experimental ECD spectra of compounds **1** and **2** in MeOH.

Compound **2** was obtained as a white amorphous powder, and HRESIMS analysis indicated a molecular ion peak at m/z 533.2640 [M + H]^+^ (Supplementary Figure S17), calculated for 533.2652. This suggests the molecular formula of **2** to be C_31_H_36_N_2_O_6_ (15 unsaturations), which is two mass units higher than that of **1**. Comparison of the NMR data (Tables 1, 2) for **2** and **1** revealed differences: the oxygen bridge between the 1′-N and C-3′ in **1** was absent, and the olefinic carbon (*δ*_C_ 163.8) at C-3′ in **1** was replaced by a carbonyl (*δ*_C_ 199.6) in **2**. This speculation was supported by the HMBC correlations (Figure 1) from H-4′ (*δ*_H_ 7.51) and H-10 to C-3′ (*δ*_C_ 163.8) and a comparative analysis of the HRESIMS for **1** and **2**. Additionally, based on the similar NOESY spectra (Figure 2) and identical ECD curves (Figure 4), the stereochemistry of **2** was assigned.

Compound **3** was obtained as a white amorphous powder with a molecular formula of C_31_H_34_N_2_O_10_, determined from the HRESIMS peak at *m/z* 595.2297 [M+ H]^+^. Evaluation of the 1H and 13C NMR data (Tables 1, 2) for compounds **3** and **2** revealed structural similarities, with a few notable differences. The double-bond carbons at *δ*_C_ 147.9 (C-18) and 148.8 (C-19) in **2** were replaced in **3** by an oxygenated quaternary carbon at *δ*_C_ 89.0 (C-18) and a carbonyl carbon at 172.8 (C-19), respectively. Additionally, the carbonyl carbon signal at *δ*_C_ 203.5 (C-20) was shifted upfield to *δ*_C_ 181.1 (C-20) in the case of **3**. These disparities could only be explained by positing the formation of an oxygen bridge bond between C-19 and C-20, resulting in a pyran-diones unit inside the macrocyclic ring in **3** instead of the cyclopentenone moiety in **2**. This hypothesis was substantiated by the HMBC correlations from H-22 to C-20 and from Me-18 to C-17, C-18, and C-19, as well as from H-17 to C-18 (Figure 1). Furthermore, the amino group (1´-NH_2_) in **2** was replaced by a nitro group (1´-NO_2_) in **3**. This deduction was supported by the resonances of C-1′, C-4′ and C-6′ of **3** being shifted upfield by Δ*δ*_C_ 3.8, 2.5, and 2.5 ppm, respectively. Conversely, the signals of C-2′, C-5′, and C-7′ were shifted downfield by Δδ_C_ 18.4, 18.7, and 7.6 ppm, respectively. These alterations, along with a comparative analysis of the molecular formulae of compounds **2** and **3**, confirmed the planar structure of **3**.

The relative configurations of all the chiral centers in **3**, except for C-18, were consistent with those of **1** and **2** based on their similar NOESY spectra (Figure 2) and NMR patterns. Consequently, like compounds **1** and **2**, compound **3** is thought to be derived from chaetogobosin V_b_ (**16**) and to share identical absolute configurations of all the chiral centers except for C-18. In addition, the NOESY correlation of H-21 to Me-18 and H-16 revealed the *β*-orientation of H-21 and Me-18. The absolute configurations of C-18 in **3** was assumed to be (18*S*), as evidenced by the comparison of experimental and calculated ECD spectra (Figure 3).

Compound **4** was obtained as a white amorphous powder with a molecular formula of C_31_H_34_N_2_O_11,_ indicating an additional oxygen atom compared to **3**. This was suggested by the HRESIMS peak at *m/z* 611.2246 [M+ H]^+^. The ^1^H NMR and^13^C NMR spectra of **4** (Tables 1, 2) exhibited a high degree of similarity to those of **3**, save for the absence of the double-bond carbon signals at C-5 and C-6 and the presence of an additional epoxide between C-5 and C-6 in **4**. This hypothesis was corroborated by the HMBC correlations from Me-12 to C-5, C-6, and C-7, as well as Me-11 to C-4, C-5, and C-6, paired with the chemical shifts of C-5 (*δ*_C_ 63.0) and C-6 (*δ*_C_ 65.1) in **4**. Furthermore, comprehensive ^1^H-^1^H COSY, HMBC, and NOESY spectrum analysis (Supplementary Figures S28–S34) elucidated the complete planar structure and relative configuration of **4**. Based on comparative analysis of the NMR data, it was assumed that the co-isolated compound **4** was an epoxidation product of **3**, derived from chaetogobosin V_b_ (**16**). This suggests that they have the same absolute configurations, except for C-5 and C-6. The relative configurations of C-5 and C-6 were identified through NOESY correlation from Me-11 to H-3 and Me-12, suggesting their α-orientations. As a result, the absolute configurations of C-5 and C-6 were assigned as (5*R*, 6*S*) based on a comparison of the experimental ECD spectrum with the calculated spectrum (Figure 3).

Compound **5** was obtained as a white amorphous powder. Its molecular formula was determined to be C_31_H_34_N_2_O_8_ (16 unsaturations) based on the HRESIMS peak at *m/z* 585.2208 [M + Na]^+^. Detailed analysis of the ^1^H NMR and^13^C NMR spectra of **5** (Tables 1, 2) indicated that they showed strong resemblance to the spectra reported for chaetogobosin G (**15**) (Sekita et al., 1982, 1983). However, two fundamental differences were observed: the absence of a methine at C-2′ in the indole portion and the presence of an additional carbonyl group at C-3′ with a chemical shift value of 200.3 ppm. This observation was supported by the HMBC correlations (Figure 1) from H-4′ (*δ*_H_ 7.34) and H-10 to C-3′. Comparison of the ^1^H and ^13^C NMR data for the di-substituted benzene unit in **5** (Tables 1, 2) with that of compounds **3** and **4** indicated a shared structural similarity. Thus, it was concluded that a nitro group (1′-NO_2_) was the substituent group attached to C-1′. Moreover, comprehensive analysis of the ^1^H-^1^H COSY, HMBC, and NOESY spectra (Figures 1, 2) provided further insight into the complete planar structure and relative configuration of **5**. Compound **5** was assumed to be produced through a series of reactions starting from chaetogobosin G (Scheme 1). The stereochemistry of **5** is preserved via these reactions, indicating that they share the same absolute configurations.

Compound **6** was isolated as a white amorphous powder, with its molecular formula determined to be C_31_H_38_N_2_O_6_ (14 unsaturations) based on the HRESIMS peak at *m/z* 535.2808 [M+ H]^+^. In-depth analysis of the 1D and 2D NMR spectra (Tables 3, 4, Supplementary Figures S47–S52) of **6** implied structural similarity to the known chaetogobosin D (Sekita et al., 1982, 1983). However, the primary distinction was the absence of the methine at C-2′ from the indole moiety, along with an additional carbonyl observed at C-3′, exhibiting a chemical shift value of 199.9 ppm. These deductions were further confirmed by HMBC cross-peaks from H-4′ (*δ*_H_ 7.34) and H-10 to C-3′ (Figure 1) and the observable the chemical shift difference in the di-substituted benzene ring moiety. Based on comparative analysis of their 1D and 2D NMR spectra, the structure of **6** is nearly identical to that of chaetogobosin D, except for the indole moiety. Therefore, it was assumed that compound **6** is a derivative of chaetogobosin D, indicating that they have the same absolute configurations.

Compound **7**, which was isolated as a white amorphous powder, has the molecular formula C_31_H_36_N_2_O_8_ (15 unsaturations) as determined by HRESIMS (m/z 565.2533 [M + H]^+^ (Supplementary Figure S62), calculated for 565.2544). The ^1^H NMR and ^13^C NMR data of **7** align closely with those of compound **6**, except for the substitution of the amino group (1-NH_2_) in **6** with a nitro group (1-NO_2_) in **7**. This substitution was confirmed by the chemical shift difference in the di-substituted benzene ring moiety and analysis of the HRESIMS data. Furthermore, detailed analysis of the ^1^H-^1^H COSY and HMBC spectra (Figures 1, 2) elucidated the complete planar structure and relative configuration of **7**. Thus, compound **7** could be produced from chaetogobosin D as **6**, indicating that they share the same absolute configurations at the chiral centers.

Compound **8** was obtained as a white amorphous powder and was found to have the same molecular formula as **6**, implying that **8** is an isomer of **6**. Comparison of the NMR data (Tables 3, 4) for **8** and **6** revealed differences, such as the absence of an exocyclic double bond between C-6 and C-12 in **6**, and the presence of the double-bond carbon signals between C-5 (*δ*_C_ 126.1) and C-6 (*δ*_C_ 132.3) in **8**. This proposal was further confirmed by the HMBC correlations (Figure 1) from Me-12 to C-5, C-6, and C-7, as well as Me-11 to C-4, C-5, and C-6. In addition, the planar structure and relative configuration of **8** were assigned through extensive analysis of 1D and 2D NMR (Supplementary Figures S65–S70). As a result, compound **8** may be derived from chaetogobosin E (**14**) (Sekita et al., 1976), which suggests that they share the same absolute configurations at the remaining chiral centers.

Compound **9** was isolated as a white amorphous powder. Its molecular formula, C_31_H_36_N_2_O_6_, was assigned by the HRESIMS peak at *m/z* 533.2635 [M+ H]^+^. The configuration of **9** was deduced by analysis of the corresponding NMR data (Supplementary Figures S74–S80). By comparing the ^13^C NMR data (Tables 2, 4) for compound **9** with compounds **1** and **8**, it was found that compound **9** shares the same benzisoxazole ring unit as compound **1** and the same perhydro-isoindolone and macrocyclic ring moiety as compound **8**. This suggests a structural similarity and possible relation between these compounds.

Compound **10** was obtained as a white amorphous powder. The molecular ion peak at *m/z* 587.2371 (calculated for C_31_H_36_N_2_NaO_8_ [M+ Na]^+^ 587.2464) confirmed that it has the same molecular formula as compound **7**. Exhaustive interpretation of the ^1^H and ^13^C NMR data for **10** (Tables 3, 4) indicated that this compound had the same molecular skeleton as **7**. However, unlike compound **7**, compound **10** did not show the presence of exocyclic double bonds at the C-6 and C-12 positions. Furthermore, the NMR data for compound **10** revealed additional double-bond carbon signals resonating at *δ*_C_ = 125.8 (C-5) and 132.7 (C-6) ppm. These differences help to distinguish compound **10** from compound **7**, despite their identical molecular formulas.

Compound **11** was obtained as a white amorphous powder with a molecular formula of C_31_H_36_N_2_O_9;_ it contains one more oxygen atom than **10**, as measured by the HRESIMS. Exhaustive interpretation of the 1D and 2D NMR spectra for **11** (Supplementary Figures S93–S97) indicated that this compound could be assumed to be an epoxidation product of **10.** This hypothesis was further confirmed by the HMBC correlations (Figure 1) from Me-12 to C-5, C-6, and C-7, along with the chemical shifts of C-5 (*δ*_C_ = 62.7 ppm) and C-6 (*δ*_C_ = 65.0 ppm) in **10**. Comparative analysis of the NMR data for **11** with the co-isolated compounds **8**–**10** indicated that they all possess the same molecular skeleton, which is derived from chaetogobosin E. They also share the same absolute configurations, except at C-5 and C-6. In the NOESY spectrum (Figure 2), the relative configurations of Me-11 and Me-12 were assigned as α-orientations. Consequently, by comparing the experimental ECD spectrum with the calculated spectrum (Figure 3), the absolute configurations of C-5 and C-6 in **11** were assumed to be (5*R*, 6*S*).

### Plausible pathways for the synthesis of 1–11

We put forward probable synthetic pathways for compounds **1**–**11**, commencing from chaetoglobosin D, E, G, or V_b_. The initial steps involve epoxidation to produce 2′, 3′-epoxy derivatives, which then undergo ring-opening reactions, followed by hydrolysis, to deliver the amino group (1′-NH_2_) derivatives (**2**, **6**, **8**). The next stage of the process involves the further oxidation of the aforementioned amino group (1′-NH_2_) to yield 1′-NO_2_ derivatives, such as compounds **5**, **7**, **10**, and **11**. Subsequently, the amino group (1′-NH_2_) derivatives undergo another round of oxidation, which yields intermediates containing nitrene. These then undergo hydrogenation and elimination reactions to generate compounds **1** and **9**. This proposed synthetic pathway is illustrated in Scheme 1A.

We also suggest plausible synthesis pathways for compounds **3** and **4**. These pathways are thought to involve epoxidation of an olefin, ring-opening reactions of the resulting epoxides, and a Baeyer–Villiger rearrangement as the key steps in the synthesis. The intermediate i is derived from chaetogobosin V_b_ via a series of reactions that are shared with compounds **5**, **7**, **10**, and **11**. From this point, intermediates i are transformed through olefin epoxidation and ring-opening reactions of the created epoxides, resulting in intermediates ii a, b. A Baeyer–Villiger rearrangement is then undergone to construct the pyran-diones moiety, ultimately yielding compounds **3** and **4**. The proposed synthetic pathway is depicted in Scheme 1B.

### Seed growth inhibition activity for *Arabidopsis thaliana*

**Figure 5:**
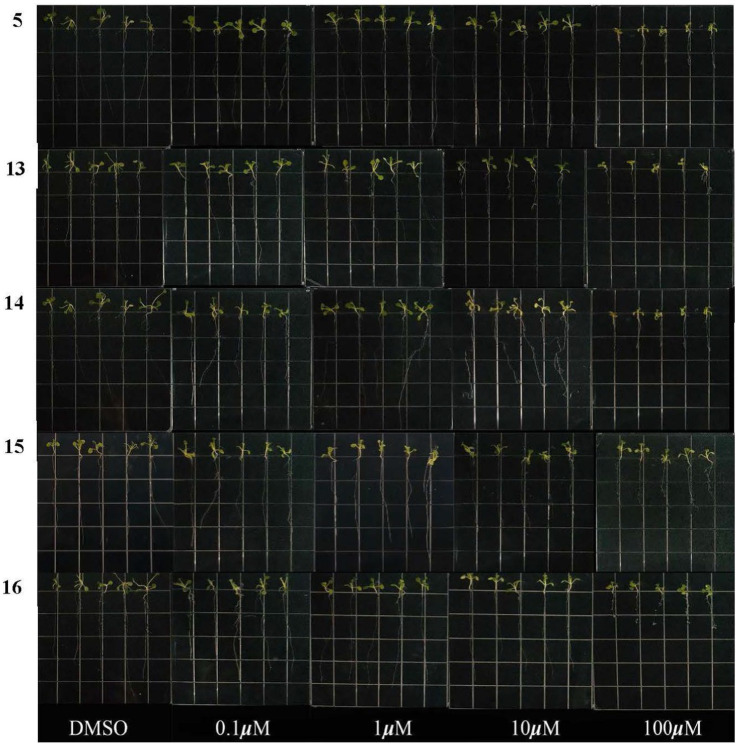
Effects of compounds **5** and **13**–**16** on root growth in *Arabidopsis thaliana*.

The inhibitory activities of compounds **5** and **13**–**16** on root growth of *Arabidopsis thaliana* were tested. As shown in Figure 5, the root length of *Arabidopsis thaliana* seeds was significantly reduced compared to a control (DMSO). At a concentration of 100 μM, the inhibition rates of compounds **5** and **13**–**16** were 51.2, 64.1, 54.5, 43.2, and 66.3%, respectively, indicating they all exhibited inhibitory activity toward seedling growth. However, at a concentration of 50 μM, only compound **13** demonstrated strong inhibitory activity. The researchers suggest that this could be due to the presence of a double-bond group at C-6 (as seen in Figure 5). Interestingly, compounds **5** and **14**–**16** exhibited similar actions, which suggests that their inhibitory effects are not related to the indole moiety.

### Anti-inflammatory activity and mechanism

**Figure 6:**
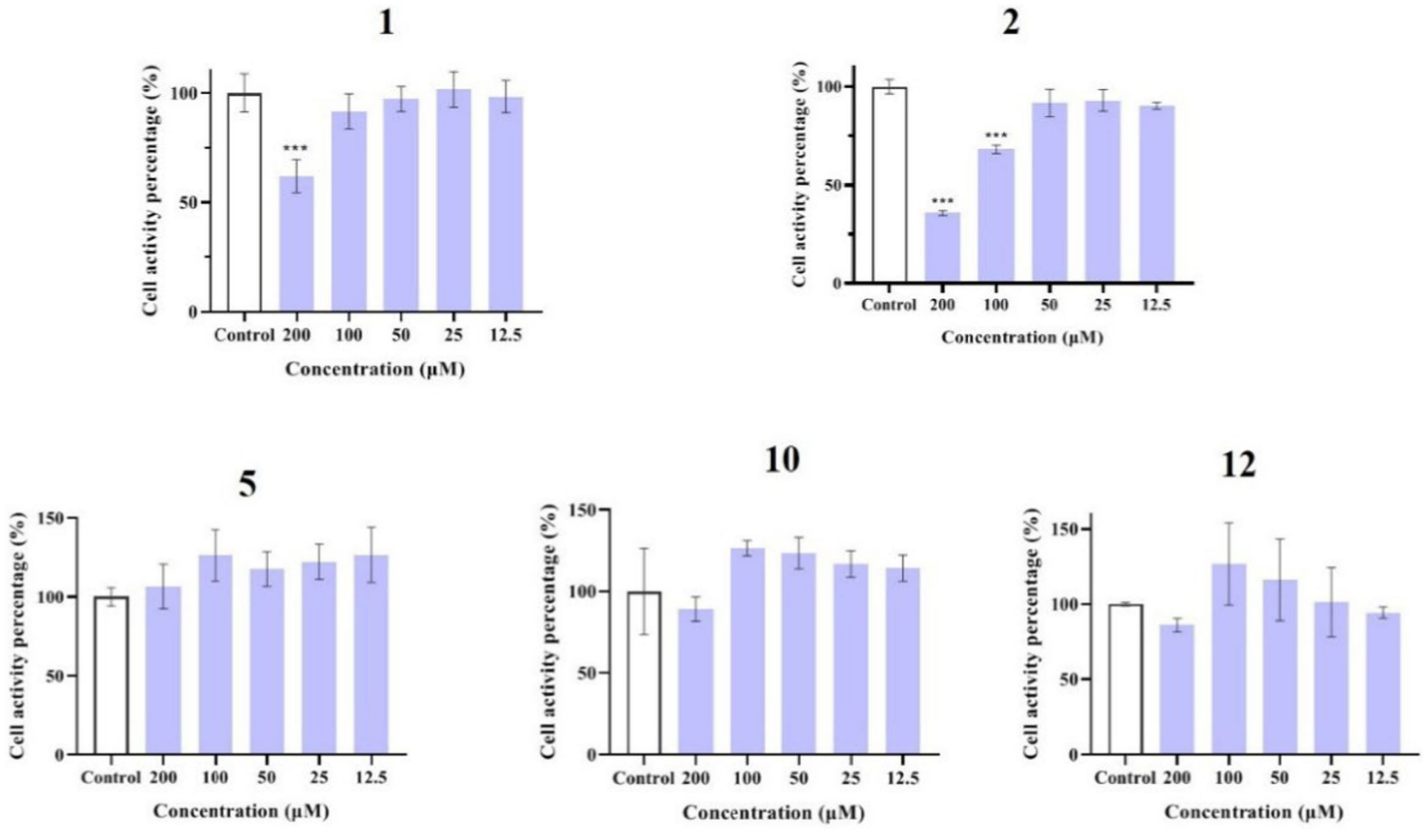
Effects of compounds **1**, **2**, **5**, **10**, and **12** on the viability of RAW264.7 cells. Data plotted represent the mean ± SD, *n* = 3. ^***^p < 0.001 vs. Control.

**Figure 7:**
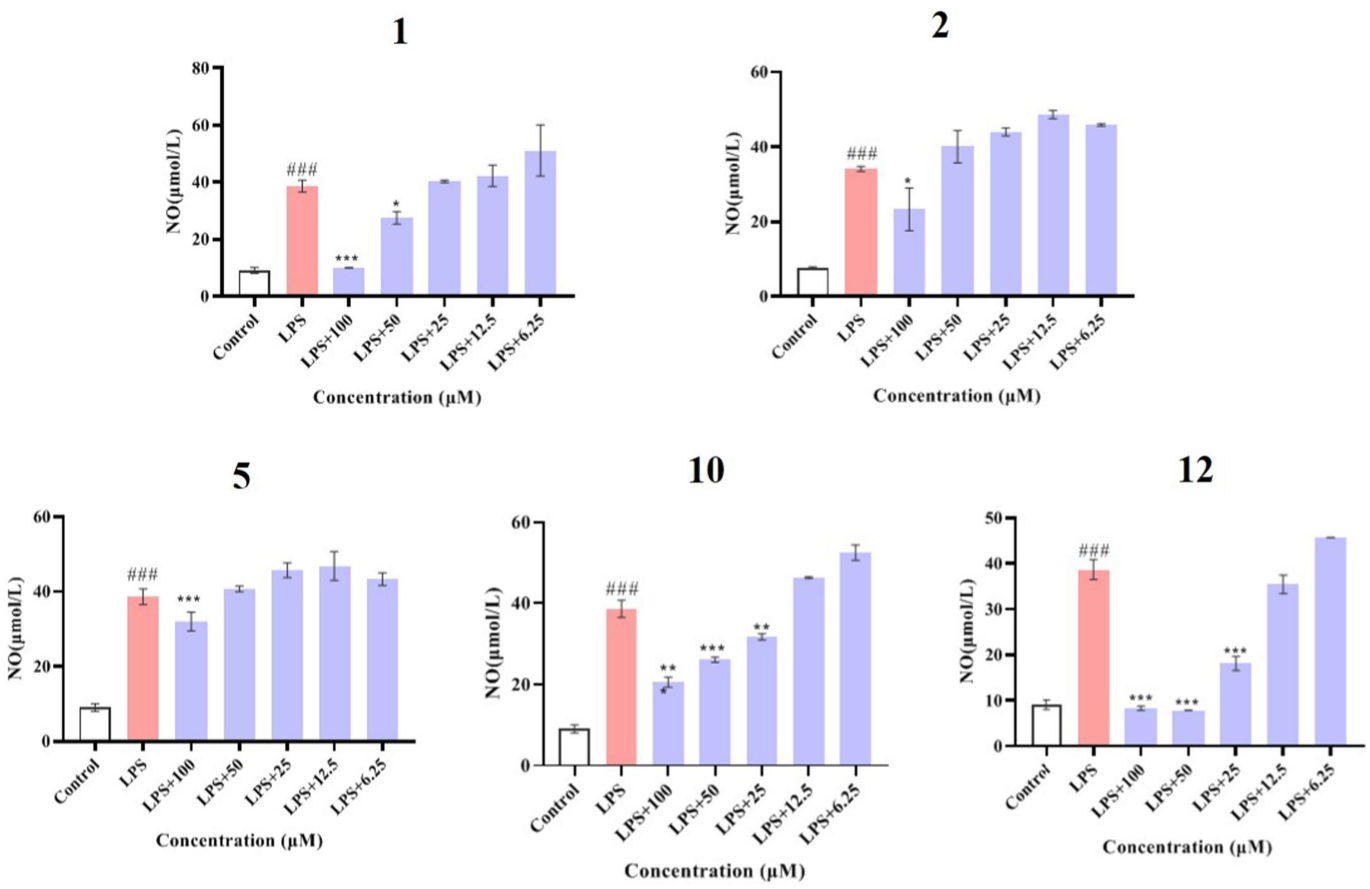
Effects of compounds **1**, **2**, **5**, **10**, and **12** on NO production in LPS-induced RAW264.7 cells. Data plotted represent the mean ± SD, *n* = 3. ^###^*p* < 0.001 vs. Control, ^***^p < 0.001 vs. LPS, ^**^p < 0.01 vs. LPS, ^*^p < 0.1 vs. LPS.

**Figure 8:**
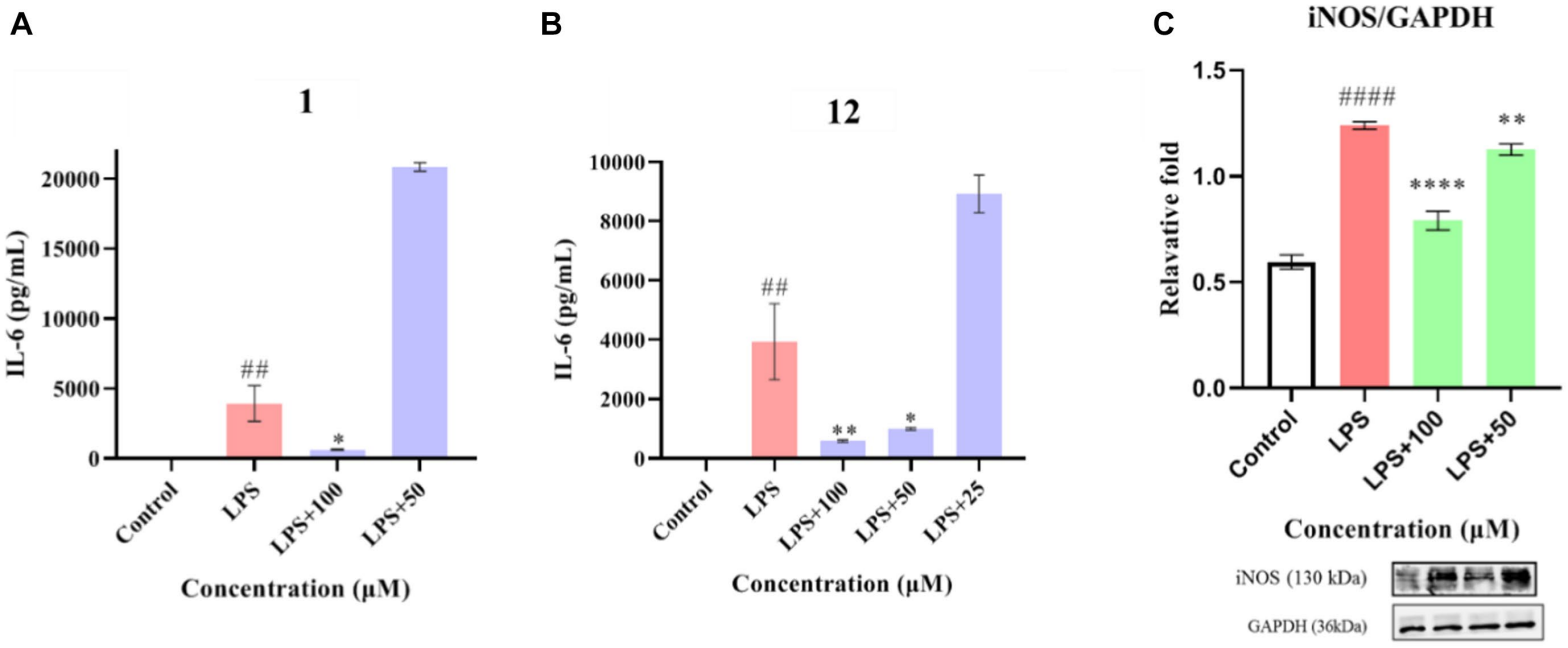
Effects of compounds **1** and **12** on the inflammatory cytokines IL-6 **(A,B)** and of compound **1** on protein expression of iNOS **(C)** in LPS-stimulated RAW264.7 cells. Data plotted represent the mean ± SD, *n* = 3. ^####^*p* < 0.0001 vs. Control, ^##^*p* < 0.01 vs. Control, ****p < 0.0001 vs. LPS, **p < 0.01 vs. LPS, *p < 0.1 vs. LPS.

Compounds **1**–**12** were evaluated for their anti-inflammatory properties using the LPS-induced NO production model in RAW264.7 cells. It was found that compounds **3**, **4**, **6**–**9**, and **11** were cytotoxic at a concentration of 12.5 μM, as determined via MTT assay. Due to this, compounds **1**, **2**, **5**, **10**, and **12** were chosen for further evaluation of their anti-inflammatory activity, at concentrations of 6.25, 12.5, 25, 50, and 100 μM, with most showing cytotoxic effects at concentrations of 200 μM (Figure 6). As displayed in Figure 7, at concentrations of 100 μM, these compounds significantly decreased the NO content in LPS-induced RAW264.7 cells, indicating anti-inflammatory effects (*p* < 0.001). The inhibition rates were 96.9, 51.9, 22.3, and 61.0% for compounds **1**, **2**, **5**, and **10**, respectively. Interestingly, compound **12** completely inhibited LPS-induced NO secretion at both 50 and 100 μM. Furthermore, the effects of these compounds on pro-inflammatory factor IL-6 were investigated. It was found that compounds **1** and **12** at 100 μM significantly suppressed the increase in IL-6 content in LPS-stimulated RAW264.7 cells (*p* < 0.001) (Figures 8A,B). Due to compound **12** becoming more cytotoxic after a few days of placement, compound **1** was chosen for further mechanistic study.

**Figure 9:**
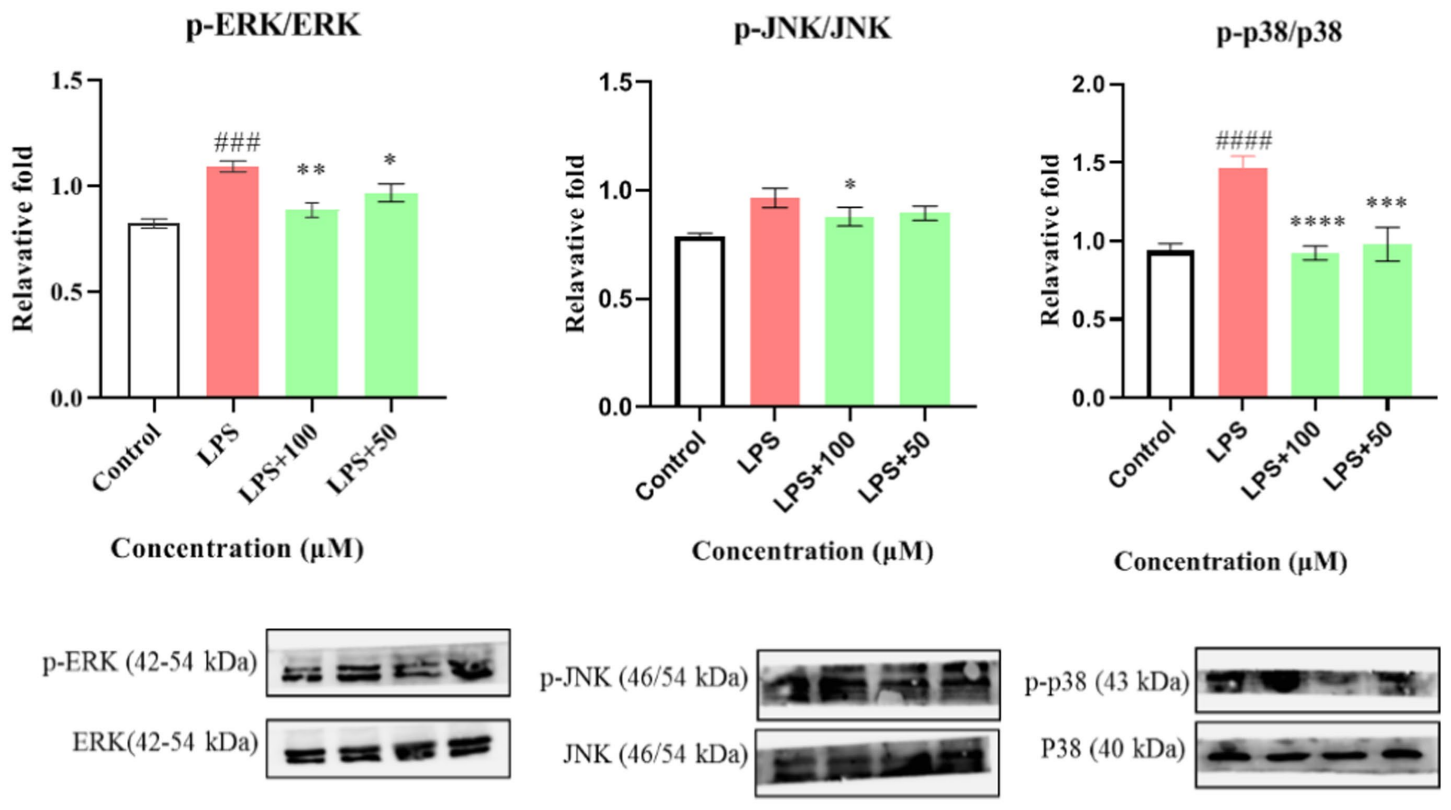
The inhibitory effect of compound **1** on activation of the MAPK pathway. Data plotted represent the mean ± SD, *n* = 3. ^####^*p* < 0.0001 vs. Control, ^###^*p* < 0.001 vs. Control, ^****^p < 0.0001 vs. LPS, ^***^p < 0.001 vs. LPS, ^**^p < 0.01 vs. LPS, ^*^p < 0.1 vs. LPS.

First, the effect of compound **1** on the expression of iNOS protein in LPS-stimulated RAW264.7 cells was studied. As shown in Figure 8C, compound **1** significantly reduced the expression of iNOS protein in LPS-induced macrophages (*p* < 0.001) at a concentration of 100 μM. Further evaluation showed that compound **1** also inhibited the protein expression of phosphorylated p38, ERK1/2, and JNK (P-p38, P-ERK1/2, and P-JNK) in LPS-induced macrophages (Figure 9). This suggests that the anti-inflammatory effect of compound **1** may be achieved through inhibition of the MAPK signaling pathway.

## Conclusion

Cytochalasans are challenging to synthesize or separate for structural modification and transformation due to their complex and variable structure. The “diversity-enhanced extracts” approach in chemical engineering can successfully address this issue. Eleven unidentified cytochalasan derivatives, some of which exhibited anti-inflammatory activity or phytotoxic effects on *Arabidopsis thaliana*, were isolated from the diversity-enhanced extract of *C. madrasense* 375. Unique ring systems, such as isoxazoles and pyranediones, as well as distinctive functional groups, such as nitro (amino) groups, are present in these compounds. Natural extracts, particularly those derived from microbial resources, are abundant in a wide range of molecules with a diverse range of structural, stereochemical, and functional groups and varied biological activities. The application of “diversity-enhanced extracts” in this study has not only increased the chemical diversity of *C. madrasense* but also expanded the utilization of desert microbial resources. The findings imply that the chemical engineering of natural product extracts could be an effective method for generation of biological molecules with novel structures for the purpose of exploring drug lead compounds and their potential applications in the pharmaceutical industry.

## Data availability statement

The datasets presented in this study can be found in online repositories. The names of the repository/repositories and accession number(s) can be found in the article/Supplementary material.

## Author contributions

QG: Conceptualization, Data curation, Formal analysis, Funding acquisition, Investigation, Methodology, Visualization, Writing – original draft. SS: Visualization, Writing – original draft. XW: Investigation, Data curation, Formal analysis, Writing – review & editing. LS: Investigation, Writing – review & editing. YR: Formal analysis, Writing – review & editing. DL: Investigation, Formal analysis, Writing – review & editing. ZY: Investigation, Methodology, Data curation, Formal analysis, Writing – review & editing. JZ: Investigation, Data curation, Formal analysis, Writing – review & editing. BY: Supervision, Writing – review & editing. XW: Resources, Formal analysis, Writing – review & editing. GD: Resources, Writing – review & editing, Data curation. LC: Conceptualization, Methodology, Project administration, Supervision, Funding acquisition, Writing – original draft.
