# Supplementary material for: Bioactive cytochalasans from the desert soil-derived fungus Chaetomium madrasense 375 obtained via a chemical engineering strategy
Source: Front Microbiol. 2024 Jan 31;14:1292870. doi: 10.3389/fmicb.2023.1292870 (PMC10864095; doi:10.3389/fmicb.2023.1292870)
Supplement: Supplementary file 1 [file Data_Sheet_2.PDF]

Electronic Supplementary Information (ESI)

Bioactive Cytochalasans from a Desert Soil-Derived Fungus *Chaetomium madrasense* 375 Based on Chemically Engineered Strategy

Qingfeng Guo,[1] Shenyu Shen,[2] Xinyang Wang,[1, 3] Lei Shi,[1] Yuwei Ren,[1] Dandan Li, [1, 3] Zhenhua Yin,[1] Juanjuan Zhang,[1] Baocheng Yang,[1] Xuewei Wang,[4] Gang Ding,[5]* and Lin Chen[1]*

[1] *Henan Comprehensive Utilization of Edible and Medicinal Plant Resources Engineering Technology Research Center, Zhengzhou Key Laboratory of Synthetic Biology of Natural Products, Huanghe Science and Technology College, Zhengzhou, 450063, China*

[2] *School of Chemistry, Xi'an Jiaotong University, Xi'an, 710049, China*

[3] *Henan University, Kaifeng 475004, China*

[4] *State Key Laboratory of Mycology, Institute of Microbiology, Chinese Academy of Sciences, Beijing, 100101, China*

[5] *Institute of Medicinal Plant Development, Chinese Academy of Medical Science and Union Medical College, Beijing 100193, China*

Corresponding Author:

**E-mail: Lin Chen: lchenchina@163.com; Gang Ding: gding@implad.ac.cn*

Table of contents

Figure S1. ^{1}H NMR spectrum of compound **1** in $CDCl_3$
Figure S2. ^{13}C NMR spectrum of compound **1** in $CDCl_3$
Figure S3. COSY spectrum of compound **1** in $CDCl_3$
Figure S4. HSQC spectrum of compound **1** in $CDCl_3$
Figure S5. HMBC spectrum of compound **1** in $CDCl_3$
Figure S6. NOESY spectrum of compound **1** in $CDCl_3$
Figure S7. HRESIMS spectrum of compound **1**
Figure S8. EIMS spectrum of Compound **1**
Figure S9. UV spectrum of Compound **1**
Figure S10. ^{1}H NMR spectrum of compound **2** in $CDCl_3$
Figure S11. ^{13}C NMR spectrum of compound **2** in $CDCl_3$
Figure S12. DEPT spectrum of compound **2** in $CDCl_3$
Figure S13. COSY spectrum of compound **2** in $CDCl_3$
Figure S14. HSQC spectrum of compound **2** in $CDCl_3$
Figure S15. HMBC spectrum of compound **2** in $CDCl_3$
Figure S16. NOESY spectrum of compound **2** in $CDCl_3$
Figure S17. HRESIMS spectrum of compound **2**
Figure S18. EIMS spectrum of Compound **2**
Figure S19. UV spectrum of Compound **2**
Figure S20. ^{1}H NMR spectrum of compound **3** in DMSO-d_6
Figure S21. ^{13}C NMR spectrum of compound **3** in DMSO-d_6
Figure S22. COSY spectrum of compound **3** in DMSO-d_6
Figure S23. HSQC spectrum of compound **3** in DMSO-d_6
Figure S24. HMBC spectrum of compound **3** in DMSO-d_6
Figure S25. HRESIMS spectrum of compound **3**
Figure S26. EIMS spectrum of Compound **3**
Figure S27. UV spectrum of Compound **3**
Figure S28. ^{1}H NMR spectrum of compound **4** in DMSO-d_6
Figure S29. ^{13}C NMR spectrum of compound **4** in DMSO-d_6
Figure S30. DEPT spectrum of compound **4** in DMSO-d_6
Figure S31. COSY spectrum of compound **4** in DMSO-d_6
Figure S32. HSQC spectrum of compound **4** in DMSO-d_6
Figure S33. HMBC spectrum of compound **4** in DMSO-d_6
Figure S34. NOESY spectrum of compound **4** in DMSO-d_6
Figure S35. HRESIMS spectrum of compound **4**
Figure S36. EIMS spectrum of Compound **4**
Figure S37. UV spectrum of Compound **4**
Figure S38. ^{1}H NMR spectrum of compound **5** in $CDCl_3$
Figure S39. ^{13}C NMR spectrum of compound **5** in $CDCl_3$
Figure S40. COSY spectrum of compound **5** in $CDCl_3$
Figure S41. HSQC spectrum of compound **5** in $CDCl_3$
Figure S42. HMBC spectrum of compound **5** in $CDCl_3$

Figure S43. NOESY spectrum of compound **5** in $CDCl_3$

Figure S44. HRESIMS spectrum of compound **5**

Figure S45. EIMS spectrum of Compound **5**

Figure S46. UV spectrum of Compound **5**

Figure S47. ^{1}H NMR spectrum of compound **6** in $CDCl_3$

Figure S48. ^{13}C NMR spectrum of compound **6** in $CDCl_3$

Figure S49. COSY spectrum of compound **6** in $CDCl_3$

Figure S50. HSQC spectrum of compound **6** in $CDCl_3$

Figure S51. HMBC spectrum of compound **6** in $CDCl_3$

Figure S52. NOESY spectrum of compound **6** in $CDCl_3$

Figure S53. HRESIMS spectrum of compound **6**

Figure S54. EIMS spectrum of Compound **6**

Figure S55. UV spectrum of Compound **6**

Figure S56. ^{1}H NMR spectrum of compound **7** in $CDCl_3$

Figure S57. ^{13}C NMR spectrum of compound **7** in $CDCl_3$

Figure S58. COSY spectrum of compound **7** in $CDCl_3$

Figure S59. HSQC spectrum of compound **7** in $CDCl_3$

Figure S60. HMBC spectrum of compound **7** in $CDCl_3$

Figure S61. NOESY spectrum of compound **7** in $CDCl_3$

Figure S62. HRESIMS spectrum of compound **7**

Figure S63. EIMS spectrum of Compound **7**

Figure S64. UV spectrum of Compound **7**

Figure S65. ^{1}H NMR spectrum of compound **8** in $CDCl_3$

Figure S66. ^{13}C NMR spectrum of compound **8** in $CDCl_3$

Figure S67. COSY spectrum of compound **8** in $CDCl_3$

Figure S68. HSQC spectrum of compound **8** in $CDCl_3$

Figure S69. HMBC spectrum of compound **8** in $CDCl_3$

Figure S70. NOESY spectrum of compound **8** in $CDCl_3$

Figure S71. HRESIMS spectrum of compound **8**

Figure S72. EIMS spectrum of Compound **8**

Figure S73. UV spectrum of Compound **8**

Figure S74. ^{1}H NMR spectrum of compound **9** in $CDCl_3$

Figure S75. ^{13}C NMR spectrum of compound **9** in $CDCl_3$

Figure S76. DEPT spectrum of compound **9** in $CDCl_3$

Figure S77. COSY spectrum of compound **9** in $CDCl_3$

Figure S78. HSQC spectrum of compound **9** in $CDCl_3$

Figure S79. HMBC spectrum of compound **9** in $CDCl_3$

Figure S80. NOESY spectrum of compound **9** in $CDCl_3$

Figure S81. HRESIMS spectrum of compound **9**

Figure S82. EIMS spectrum of Compound **9**

Figure S83. UV spectrum of Compound **9**

Figure S84. ^{1}H NMR spectrum of compound **10** in $CDCl_3$

Figure S85. ^{13}C NMR spectrum of compound **10** in $CDCl_3$

Figure S86. COSY spectrum of compound **10** in $CDCl_3$

Figure S87. HSQC spectrum of compound **10** in $CDCl_3$

Figure S88. HMBC spectrum of compound **10** in $CDCl_3$

Figure S89. NOESY spectrum of compound **10** in $CDCl_3$

Figure S90. HRESIMS spectrum of compound **10**

Figure S91. EIMS spectrum of Compound **10**

Figure S92. UV spectrum of Compound **10**

Figure S93. ^{1}H NMR spectrum of compound **11** in DMSO-d_6

Figure S94. ^{13}C NMR spectrum of compound **11** in DMSO-d_6

Figure S95. COSY spectrum of compound **11** in DMSO-d_6

Figure S96. HSQC spectrum of compound **11** in DMSO-d_6

Figure S97. HMBC spectrum of compound **11** in DMSO-d_6

Figure S98. HRESIMS spectrum of compound **11**

Figure S99. EIMS spectrum of Compound **11**

Figure S100. UV spectrum of Compound **11**

Figure S101. Experimental ECD spectra of **1** and calculated ECD spectra for (3*S*, 4*R*, 7*S*, 8R, 9*R*, 17*R*, 21*R*)-**1** and (3*R*, 4*S*, 7*R*, 9*S*, 16*S*, 21*S*)-**1**

Figure S102. Optimized geometries of predominant conformers for compound (3*S*, 4*R*, 7*S*, 8R, 9*R*, 17*R*, 21*R*)-**1** at the B3LYP/6-31G (d, p) level in the gas phase.

Figure S103. Experimental ECD spectra of compounds **1** and **2** in MeOH

Figure S104. Optimized geometries of predominant conformers for compound (3*S*, 4*R*, 7*S*, 8*R*, 9*R*, 17*R*, 18S, 21*R*)-**3** at the B3LYP/6-31G(d,p) level in the gas phase.

Figure S105. Experimental and calculated ECD spectra of Compound **3**

Figure S106. Optimized geometries of predominant conformers for compound (3*S*, 4*R*, 6S, 7*S*, 8*R*, 9*S*, 16*S*, 17*R*, 18*S*, 21*R*)-**4** at the B3LYP/6-31G(d,p) level in the gas phase.

Figure S107. Experimental and calculated ECD spectra of Compound **4**

Figure S108. Optimized geometries of predominant conformers for compound (3*S*, 4*R*, 5*R*, 6*S*, 7*S*, 8*R*, 9*S*, 16*S*, 20*S*)-**11** at the B3LYP/6-31G(d,p) level in the gas phase.

Figure S109. Experimental and calculated ECD spectra of Compound **11**

Table S1. Gibbs free energies[a] and equilibrium populations[b] of low-energy conformers of (3*S*, 4*R*, 7S, 9*R*, 16*S*, 19*S*)-**1**

Table S2. Cartesian coordinates for the low-energy reoptimized MMFF conformers of (3*S*, 4*R*, 7*S*, 8R, 9*R*, 17*R*, 21*R*)-**1** at B3LYP/6-31G (d, p) level of theory in MeOH

Table S3. Gibbs free energies[*a*] and equilibrium populations[*b*] of low-energy conformers of (3*S*, 4*R*, 7*S*, 8*R*, 9*R*, 17*R*, 18S, 21*R*)-**3**.

Table S4. Cartesian coordinates for the low-energy reoptimized MMFF conformers of (3*S*, 4*R*, 7*S*, 8*R*, 9*R*, 17*R*, 18S, 21*R*)-**3** at B3LYP/6-31G (d, p) level of theory in MeOH.

Table S5. Gibbs free energies[*a*] and equilibrium populations[*b*] of low-energy conformers of (3*S*, 4*R*, 6S, 7*S*, 8*R*, 9*S*, 16*S*, 17*R*, 18*S*, 21*R*)-**4**.

Table S6. Cartesian coordinates for the low-energy reoptimized MMFF conformers of (3*S*, 4*R*, 6S, 7*S*, 8*R*, 9*S*, 16*S*, 17*R*, 18*S*, 21*R*)-**4** at B3LYP/6-31G (d, p) level of theory in MeOH.

Table S7. Gibbs free energies[*a*] and equilibrium populations[*b*] of low-energy conformers of (3*S*, 4*R*, 5*R*, 6*S*, 7*S*, 8*R*, 9*S*, 16*S*, 20*S*)-**11**.

Table S8. Cartesian coordinates for the low-energy reoptimized MMFF conformers of (3*S*, 4*R*, 5*R*, 6*S*, 7*S*, 8*R*, 9*S*, 16*S*, 20*S*)-**11** at B3LYP/6-31G (d, p) level of theory in MeOH.

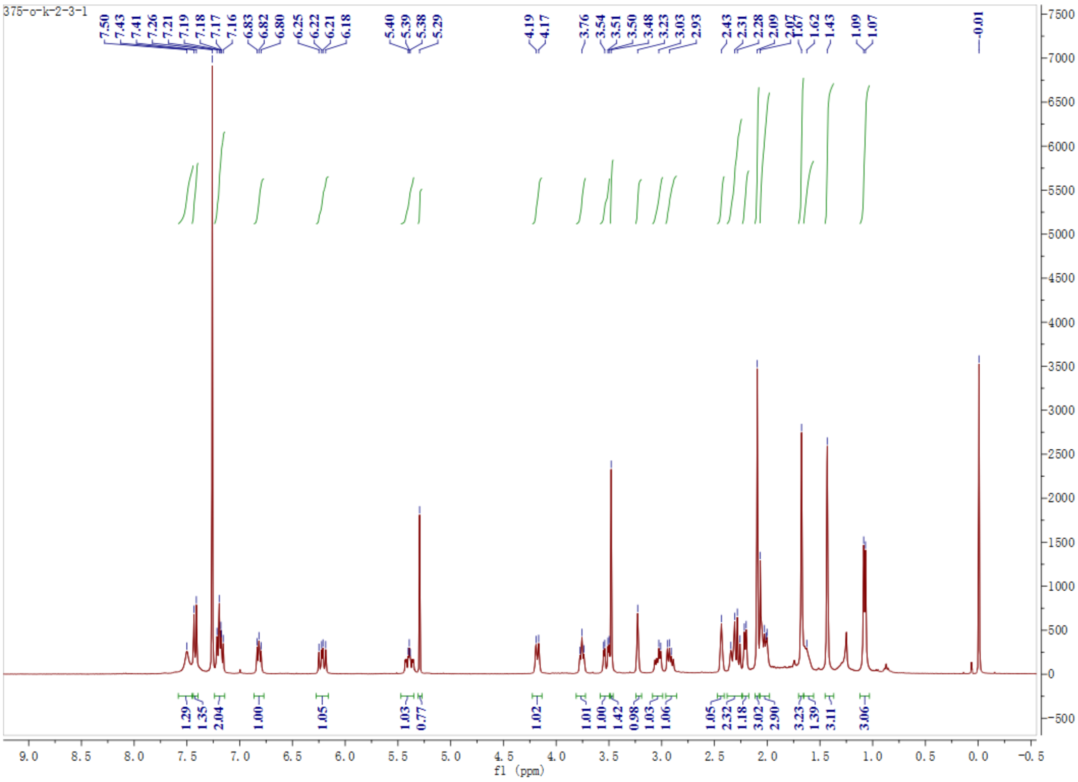

Figure S1. ^{1}H NMR spectrum of compound **1** in CD_3OD (400 MHz)

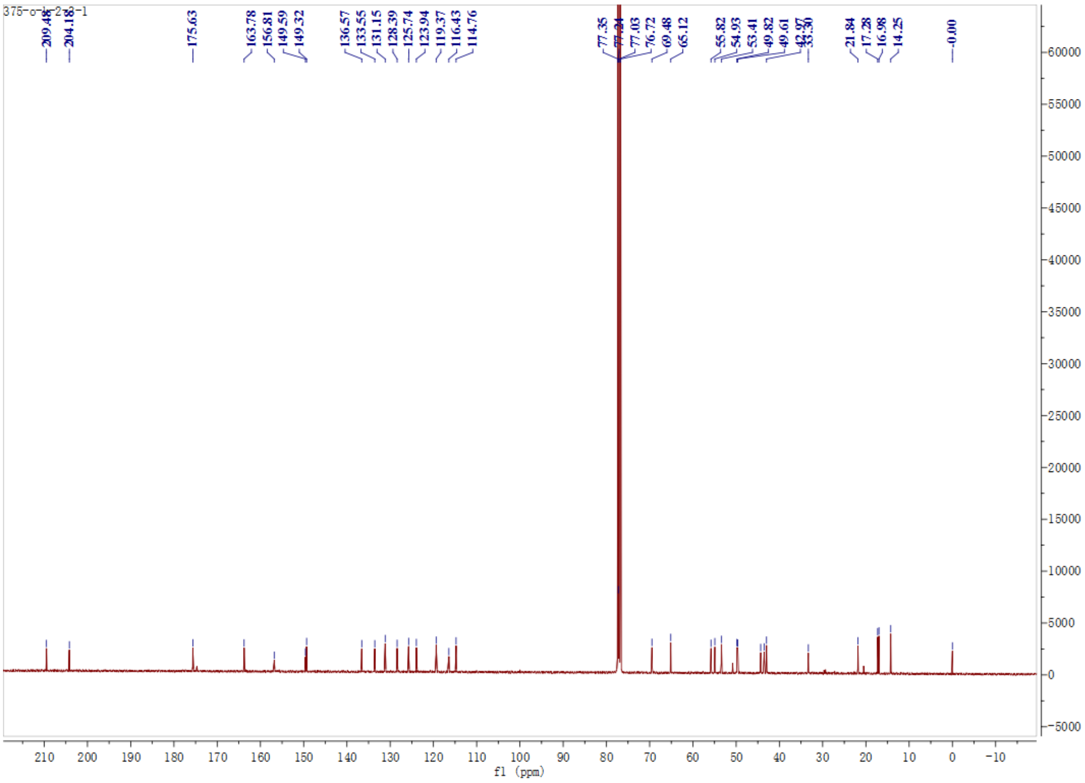

Figure S2. ^{13}C NMR spectrum of compound **1** in CD_3OD (100 MHz)

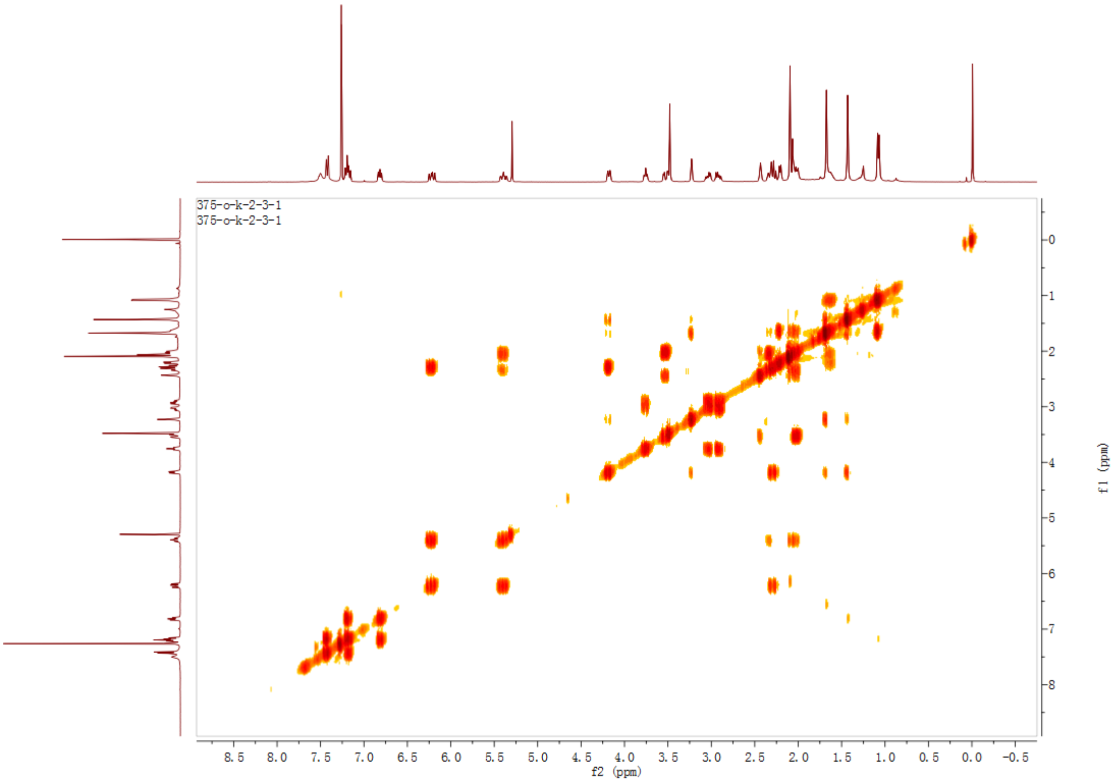

Figure S3. COSY spectrum of compound **1** in CD_3OD (400 MHz)

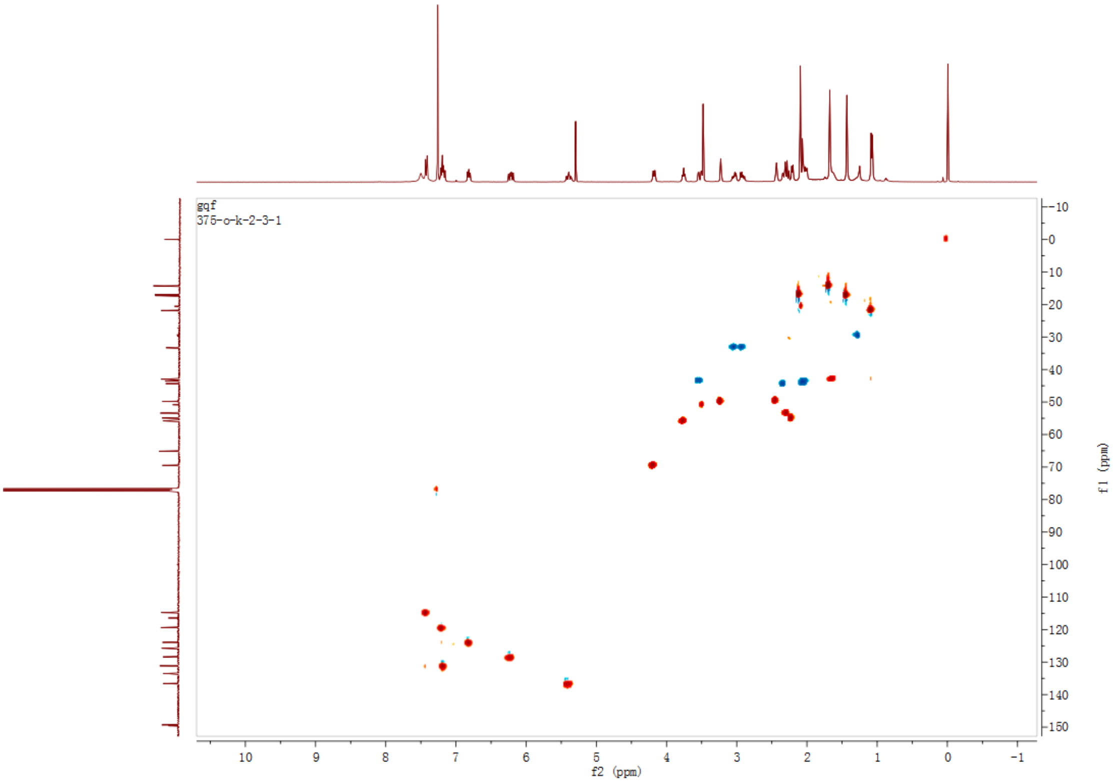

Figure S4. HSQC spectrum of compound **1** in CD_3OD (400 MHz)

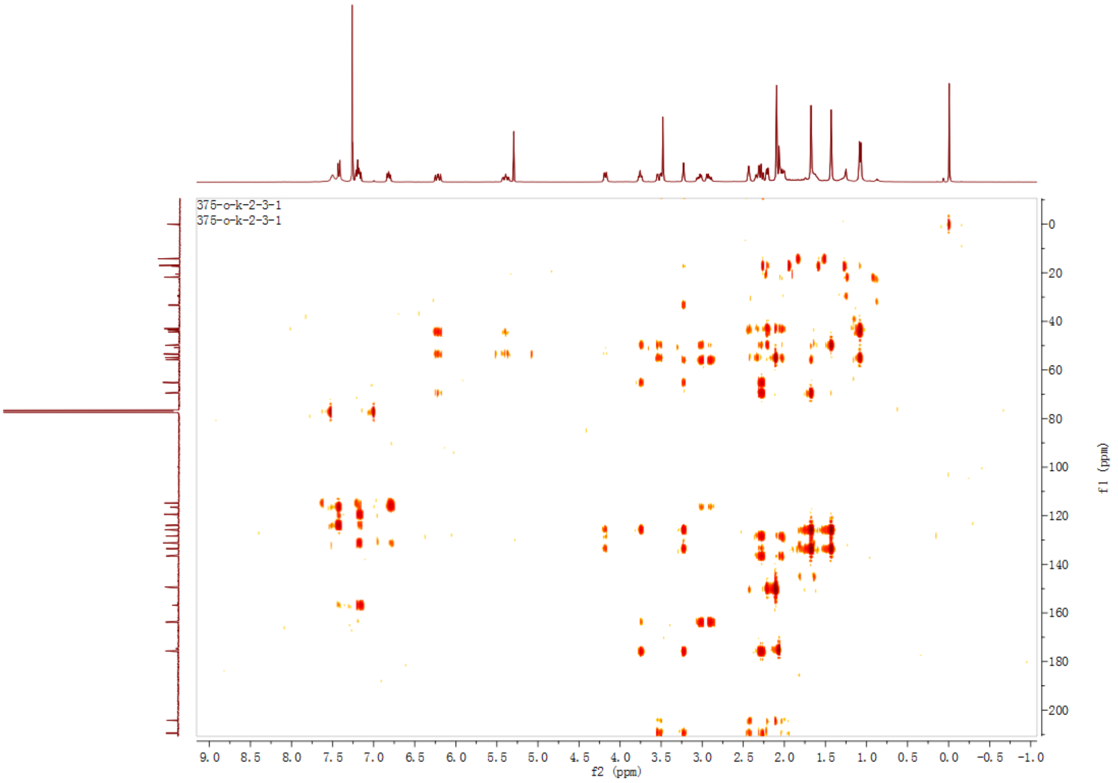

Figure S5. HMBC spectrum of compound **1** in CD_3OD (400 MHz)

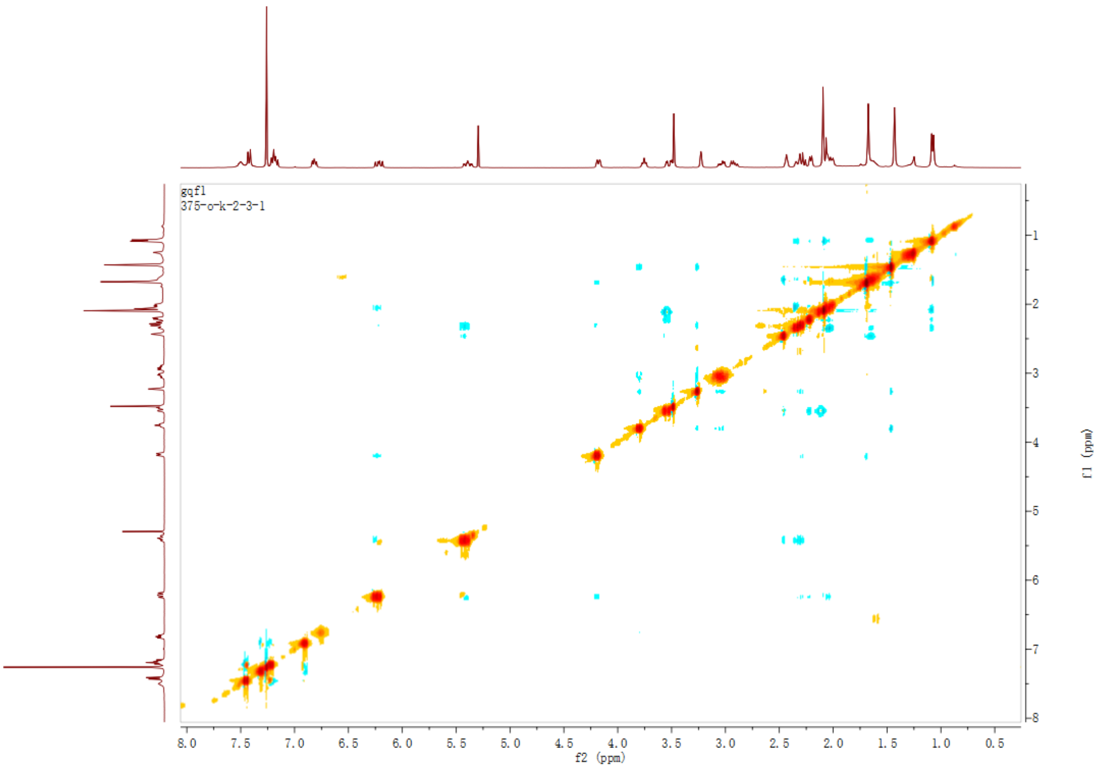

Figure S6. NOESY spectrum of compound **1** in CD_3OD (400 MHz)

Single Mass Analysis
Tolerance = 5.0 mDa / DBE: min = -1.5, max = 50.0
Element prediction: Off
Monoisotopic Mass, Even Electron Ions
2671 formula(e) evaluated with 23 results within limits (up to 50 closest results for each mass)
Elements Used:
C: 0-500 H: 0-1000 N: 0-200 O: 0-200

Mass	Calc. Mass	mDa	PPM	DBE	Formula	C	H	N	O
531.2482	531.2482	0.0	0.0	21.5	C28 H27 N12	28	27	12	
	531.2487	-0.5	-0.9	14.5	C13 H23 N24 O	13	23	24	1
	531.2487	-0.5	-0.9	3.5	C15 H35 N10 O11	15	35	10	11
	531.2473	0.9	1.7	9.5	C12 H27 N20 O5	12	27	20	5
	531.2473	0.9	1.7	-1.5	C14 H39 N6 O15	14	39	6	15
	531.2495	-1.3	-2.4	15.5	C31 H35 N2 O6	31	35	2	6
	531.2468	1.4	2.6	16.5	C27 H31 N8 O4	27	31	8	4
	531.2500	-1.8	-3.4	8.5	C16 H31 N14 O7	16	31	14	7
	531.2460	2.2	4.1	4.5	C11 H31 N16 O9	11	31	16	9
	531.2505	-2.3	-4.3	1.5	C H27 N26 O8	1	27	26	8
	531.2508	-2.6	-4.9	20.5	C32 H31 N6 O2	32	31	6	2
	531.2455	2.7	5.1	11.5	C26 H35 N4 O8	26	35	4	8
	531.2514	-3.2	-6.0	13.5	C17 H27 N18 O3	17	27	18	3
	531.2514	-3.2	-6.0	2.5	C19 H39 N4 O13	19	39	4	13
	531.2447	3.5	6.6	-0.5	C10 H35 N12 O13	10	35	12	13
	531.2446	3.6	6.8	10.5	C8 H23 N26 O3	8	23	26	3
	531.2519	-3.7	-7.0	6.5	C2 H23 N30 O4	2	23	30	4
	531.2442	4.0	7.5	6.5	C25 H39 O12	25	39		12
	531.2441	4.1	7.7	17.5	C23 H27 N14 O2	23	27	14	2
	531.2527	-4.5	-8.5	7.5	C20 H35 N8 O9	20	35	8	9
	531.2436	4.6	8.7	24.5	C38 H31 N2 O	38	31	2	1
	531.2433	4.9	9.2	5.5	C7 H27 N22 O7	7	27	22	7
	531.2532	-5.0	-9.4	11.5	C3 H19 N34	3	19	34	

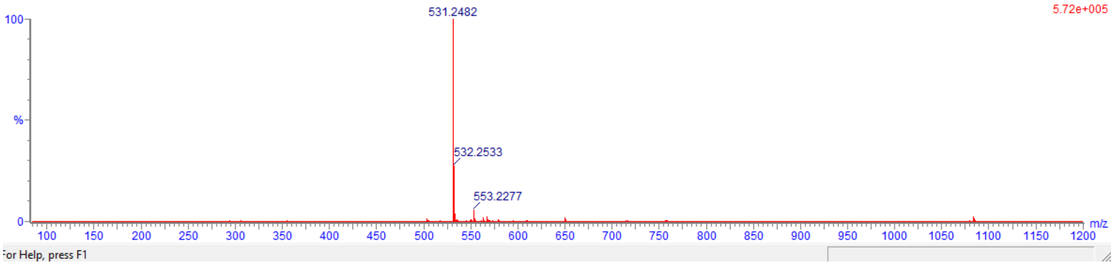

For Help, press F1

Figure S7. HRESIMS spectrum of compound **1**

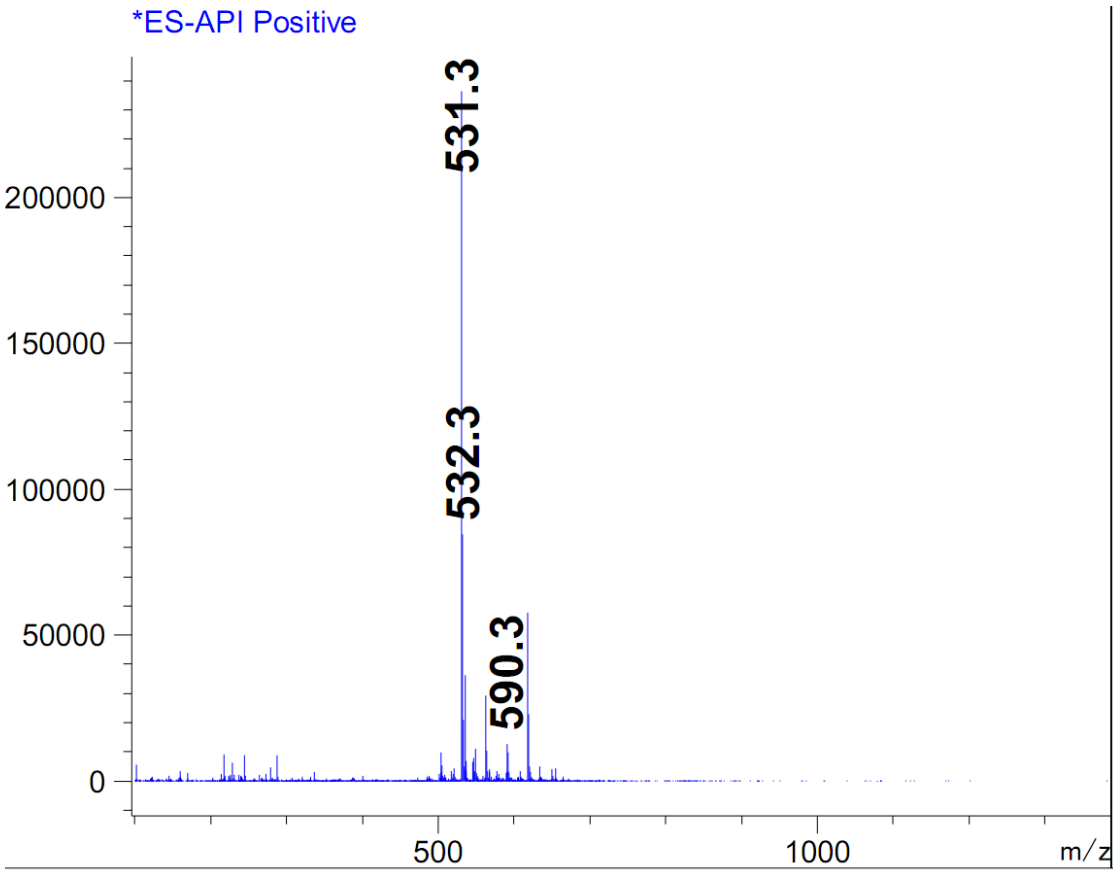

Figure S8. EIMS spectrum of Compound **1**

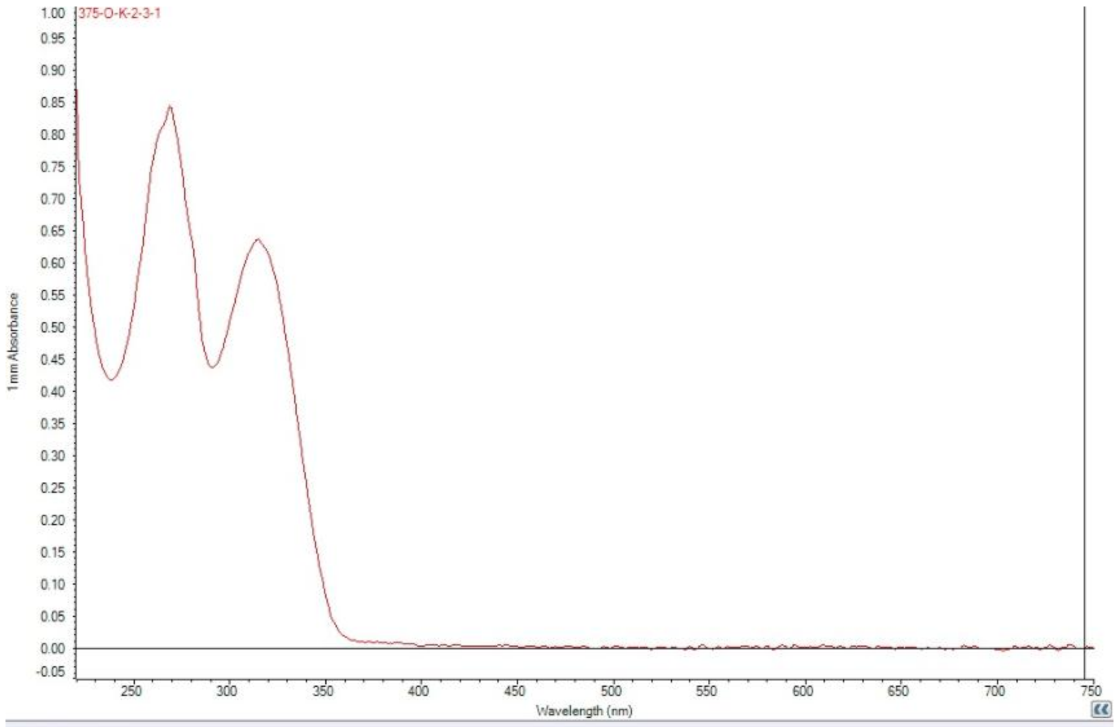

Figure S9. UV spectrum of Compound **1**

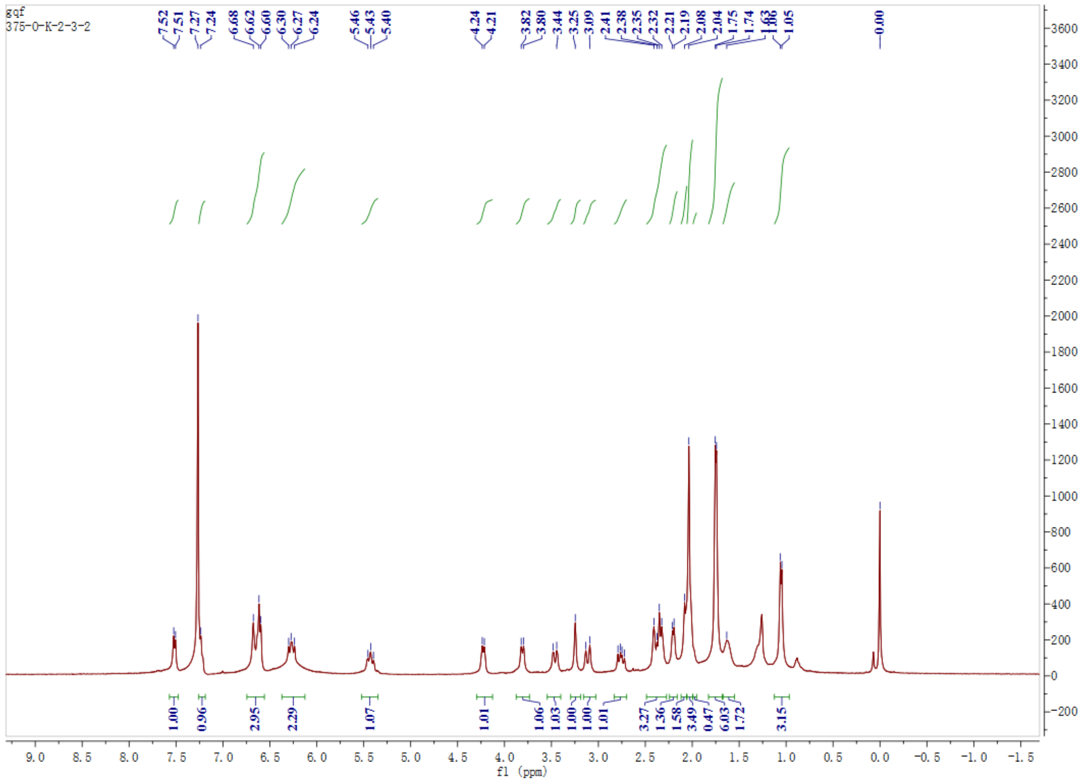

Figure S10. ^{1}H NMR spectrum of compound **2** in DMSO-d$_6$ (400 MHz)

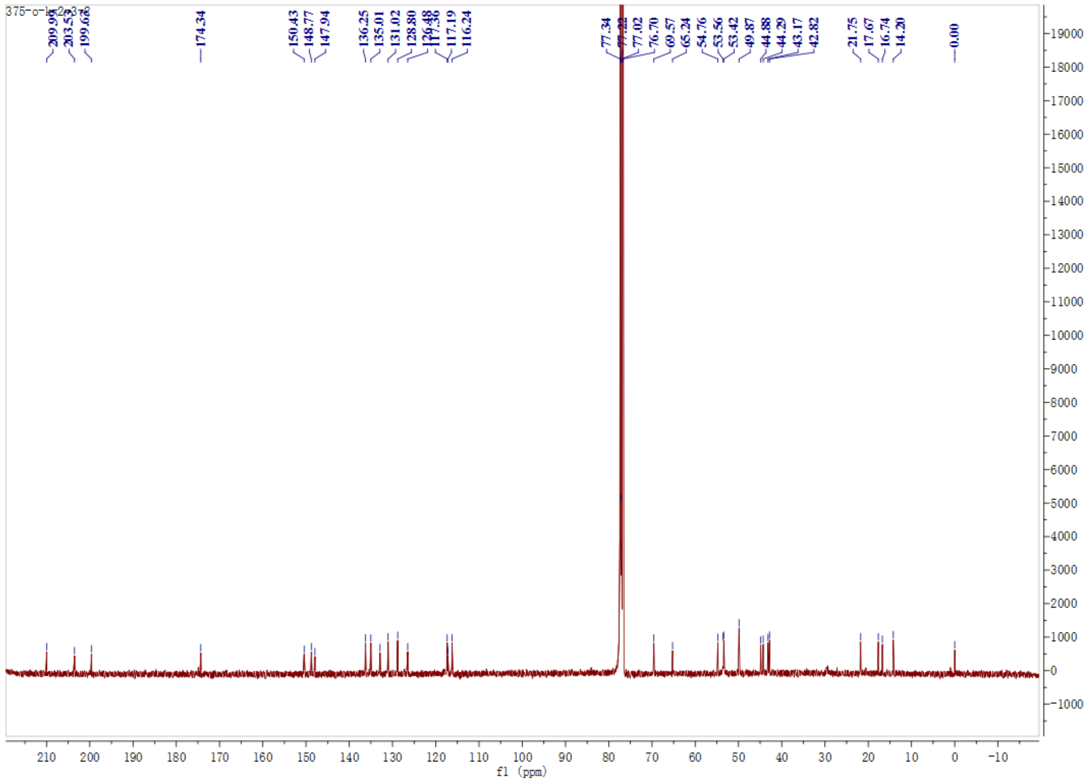

Figure S11. ^{13}C NMR spectrum of compound **2** in DMSO-*d*$_6$ (100 MHz)

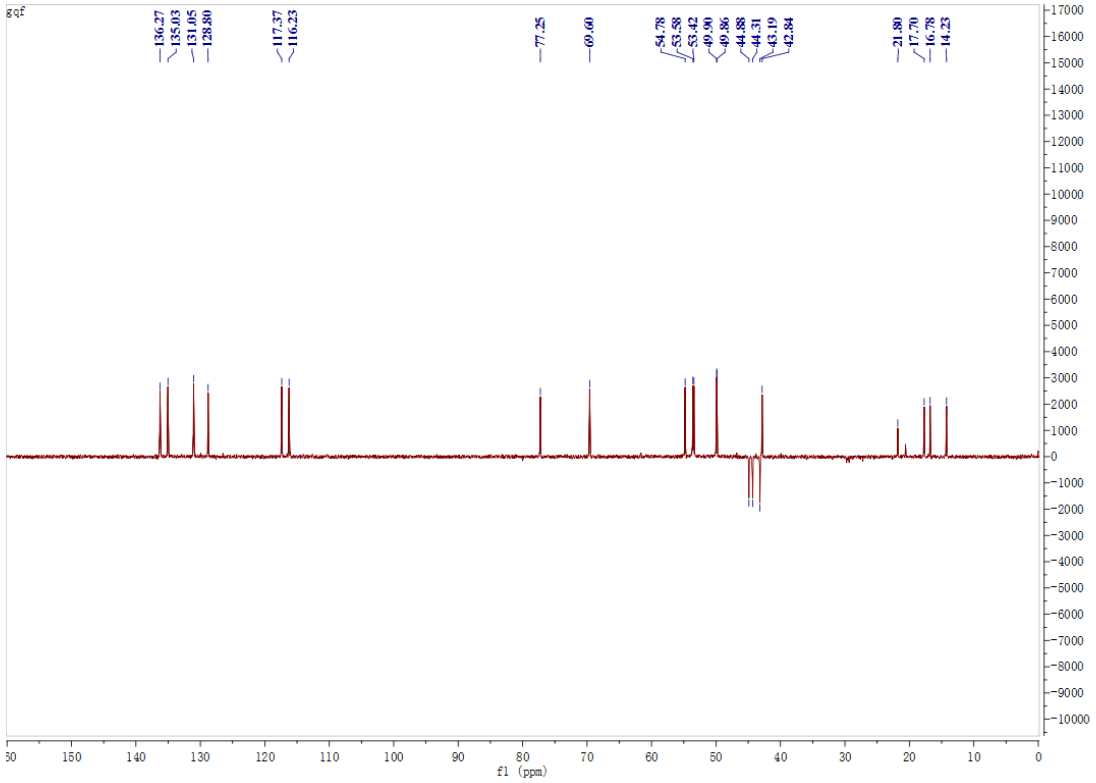

Figure S12. DEPT spectrum of compound **2** in DMSO-d_6 (100 MHz)

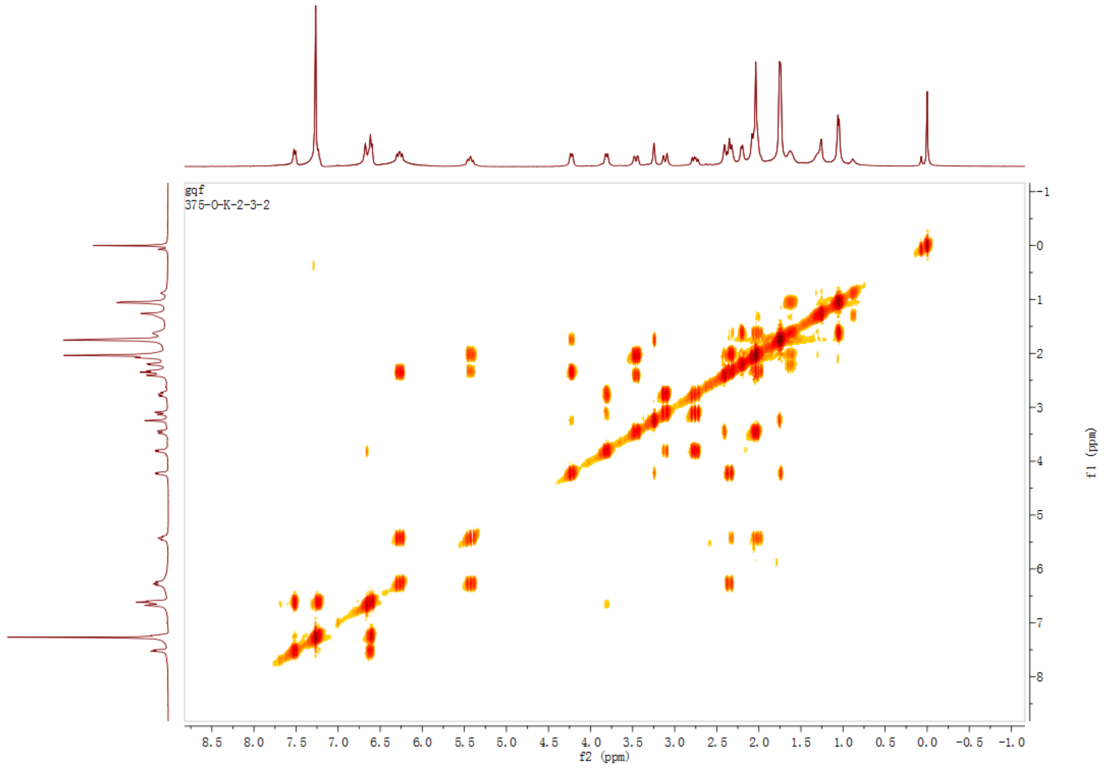

Figure S13. COSY spectrum of compound **2** in DMSO-d_6 (400 MHz)

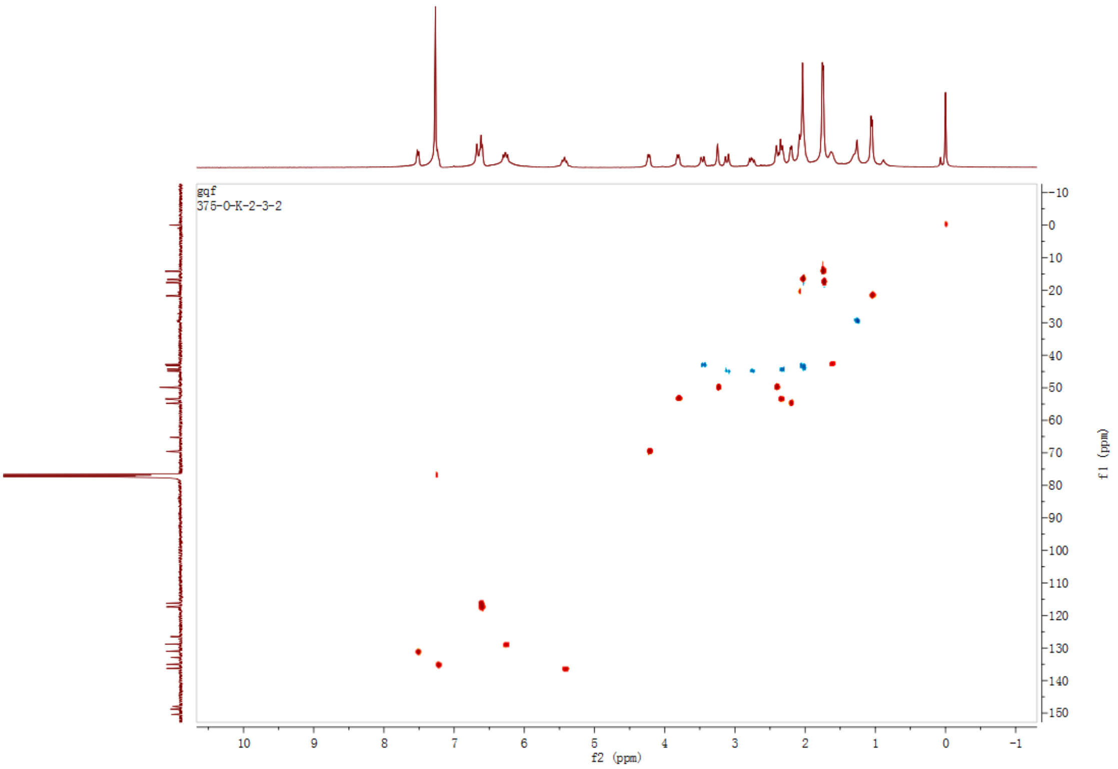

Figure S14. HSQC spectrum of compound **2** in DMSO-d_6 (400 MHz)

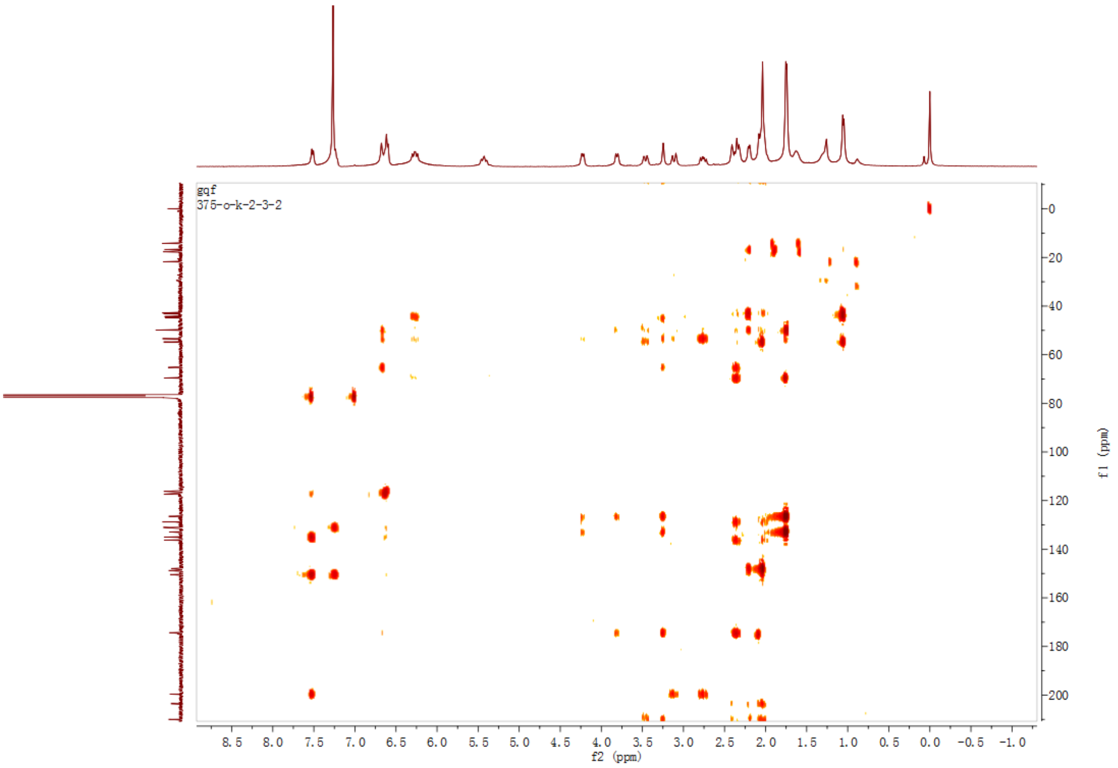

Figure S15. HMBC spectrum of compound **2** in DMSO-d_6 (400 MHz)

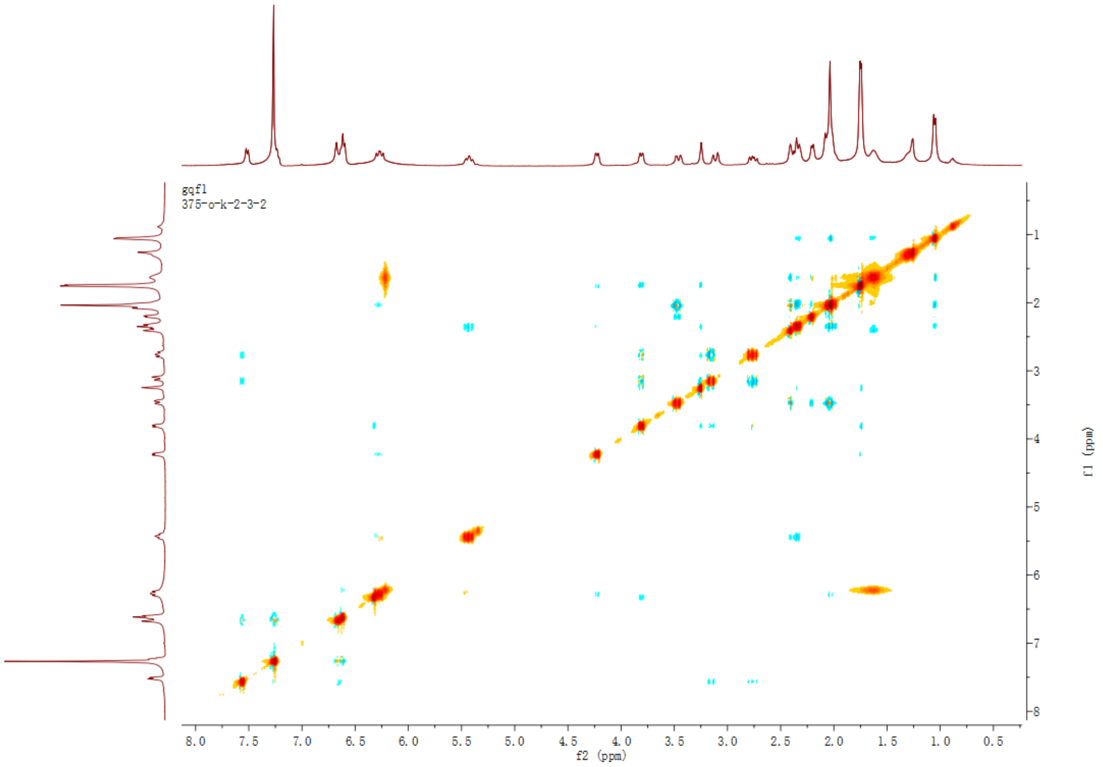

Figure S16. NOESY spectrum of compound **1** in DMSO-d_6 (400 MHz)

Single Mass Analysis

Tolerance = 5.0 mDa / DBE: min = -1.5, max = 50.0

Element prediction: Off

Monoisotopic Mass, Even Electron Ions

2695 formula(e) evaluated with 22 results within limits (up to 50 closest results for each mass)

Elements Used:

C: 0-500 H: 0-1000 N: 0-200 O: 0-200

Mass	Calc. Mass	mDa	PPM	DBE	Formula	C	H	N	O
533.2640	533.2638	0.2	0.4	20.5	C28 H29 N12	28	29	12	
	533.2643	-0.3	-0.6	2.5	C15 H37 N10 O11	15	37	10	11
	533.2643	-0.3	-0.6	13.5	C13 H25 N24 O	13	25	24	1
	533.2630	1.0	1.9	8.5	C12 H29 N20 O5	12	29	20	5
	533.2652	-1.2	-2.3	14.5	C31 H37 N2 O6	31	37	2	6
	533.2625	1.5	2.8	15.5	C27 H33 N8 O4	27	33	8	4
	533.2657	-1.7	-3.2	7.5	C16 H33 N14 O7	16	33	14	7
	533.2662	-2.2	-4.1	0.5	C H29 N26 O8	1	29	26	8
	533.2616	2.4	4.5	3.5	C11 H33 N16 O9	11	33	16	9
	533.2665	-2.5	-4.7	19.5	C32 H33 N6 O2	32	33	6	2
	533.2611	2.9	5.4	10.5	C26 H37 N4 O8	26	37	4	8
	533.2670	-3.0	-5.6	12.5	C17 H29 N18 O3	17	29	18	3
	533.2670	-3.0	-5.6	1.5	C19 H41 N4 O13	19	41	4	13
	533.2675	-3.5	-6.6	5.5	C2 H25 N30 O4	2	25	30	4
	533.2603	3.7	6.9	9.5	C8 H25 N26 O3	8	25	26	3
	533.2603	3.7	6.9	-1.5	C10 H37 N12 O13	10	37	12	13
	533.2598	4.2	7.9	5.5	C25 H41 O12	25	41		12
	533.2598	4.2	7.9	16.5	C23 H29 N14 O2	23	29	14	2
	533.2683	-4.3	-8.1	6.5	C20 H37 N8 O9	20	37	8	9
	533.2593	4.7	8.8	23.5	C38 H33 N2 O	38	33	2	1
	533.2688	-4.8	-9.0	10.5	C3 H21 N34	3	21	34	
	533.2689	-4.9	-9.2	-0.5	C5 H33 N20 O10	5	33	20	10

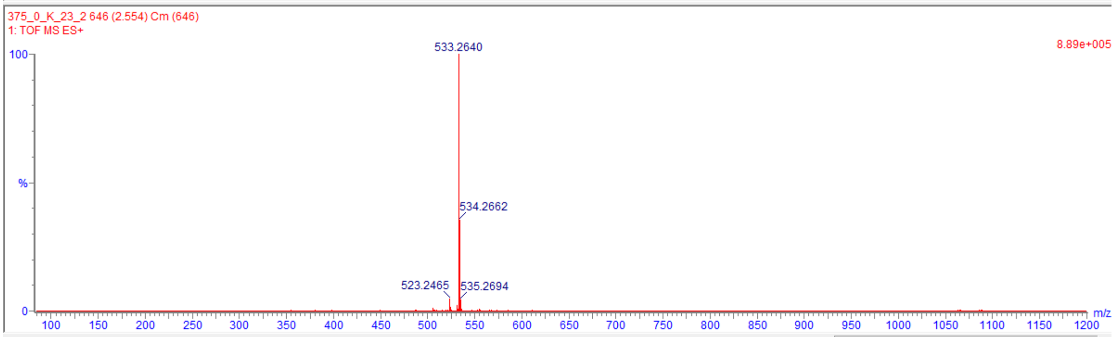

For Help, press F1

Figure S17. HRESIMS spectrum of compound **2**

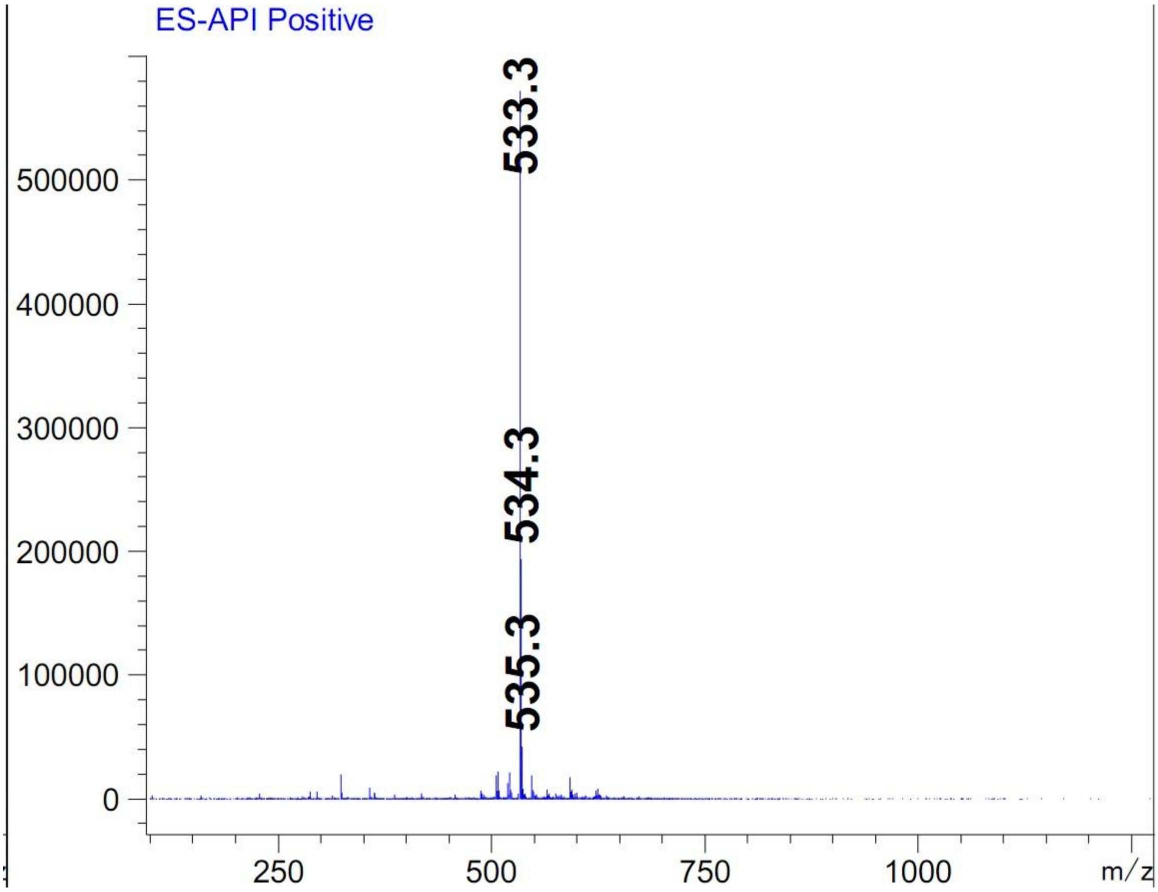

Figure S18. EIMS spectrum of Compound **2**

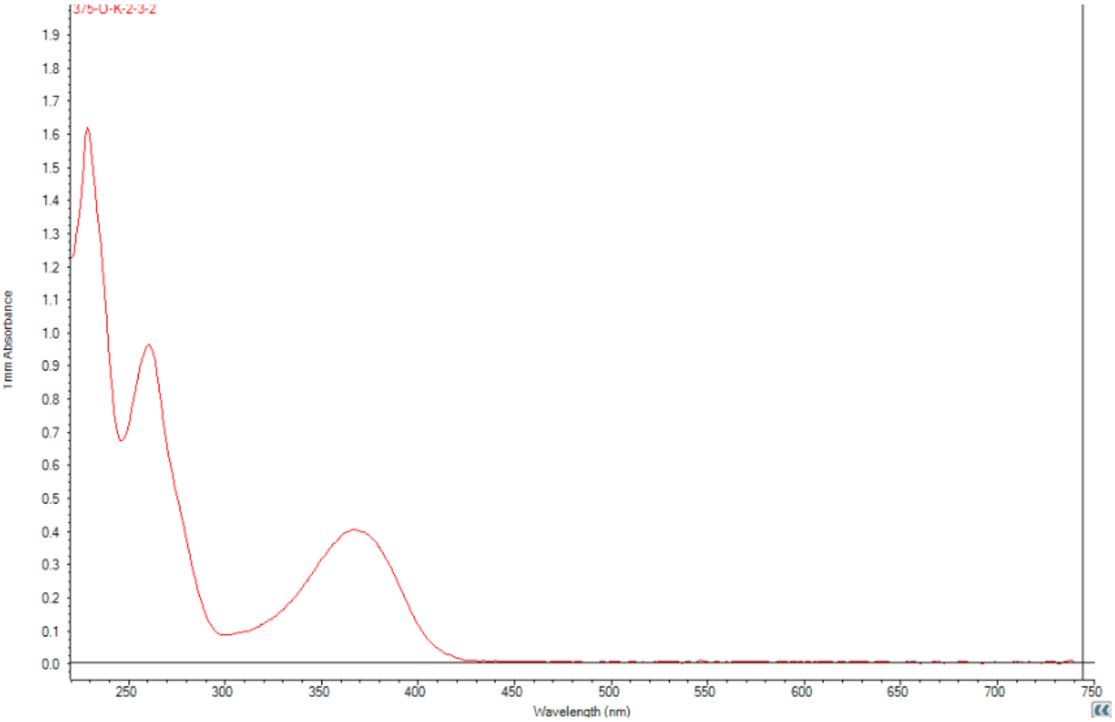

Figure S19. UV spectrum of Compound **2**

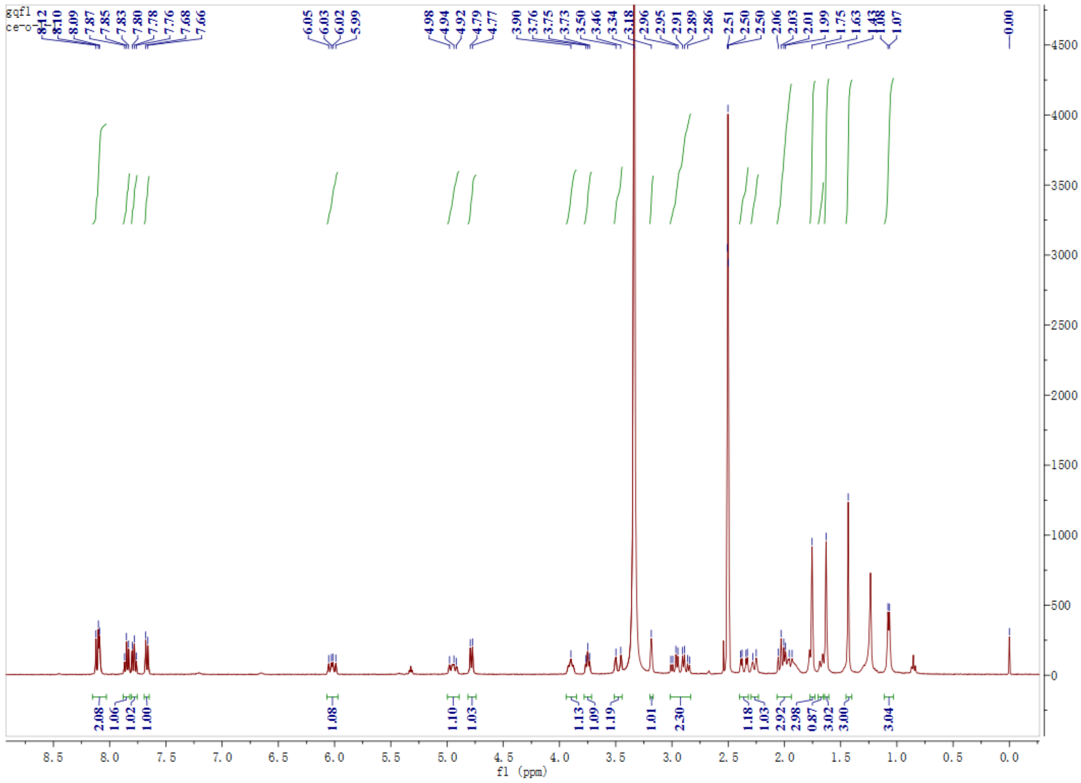

Figure S20. ^{1}H NMR spectrum of compound **3** in DMSO-d_6 (400 MHz)

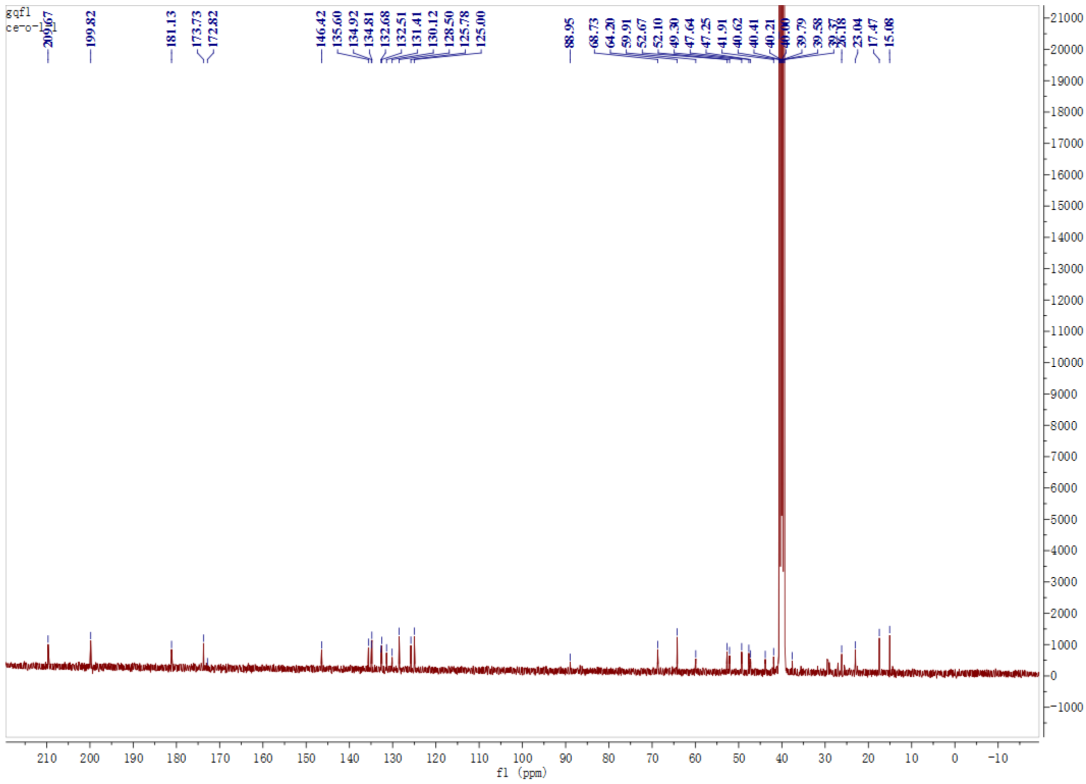

Figure S21. ^{13}C NMR spectrum of compound **3** in DMSO-d_6 (100 MHz)

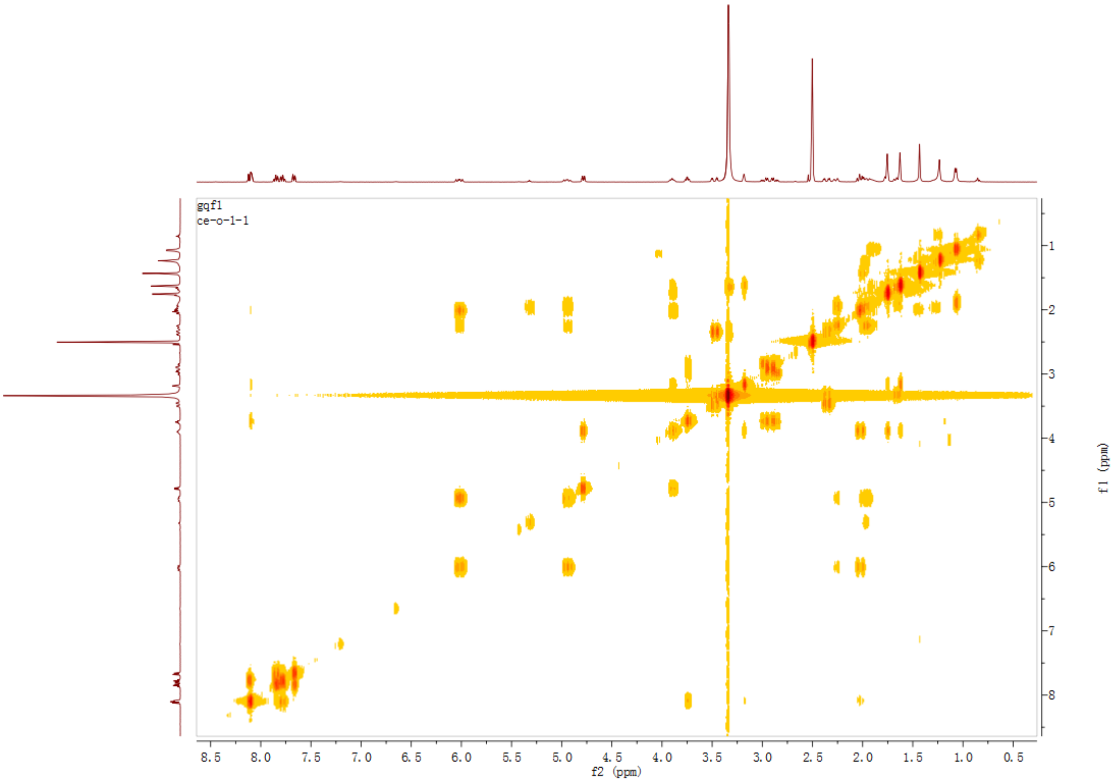

Figure S22. COSY spectrum of compound **3** in DMSO-d_6 (400 MHz)

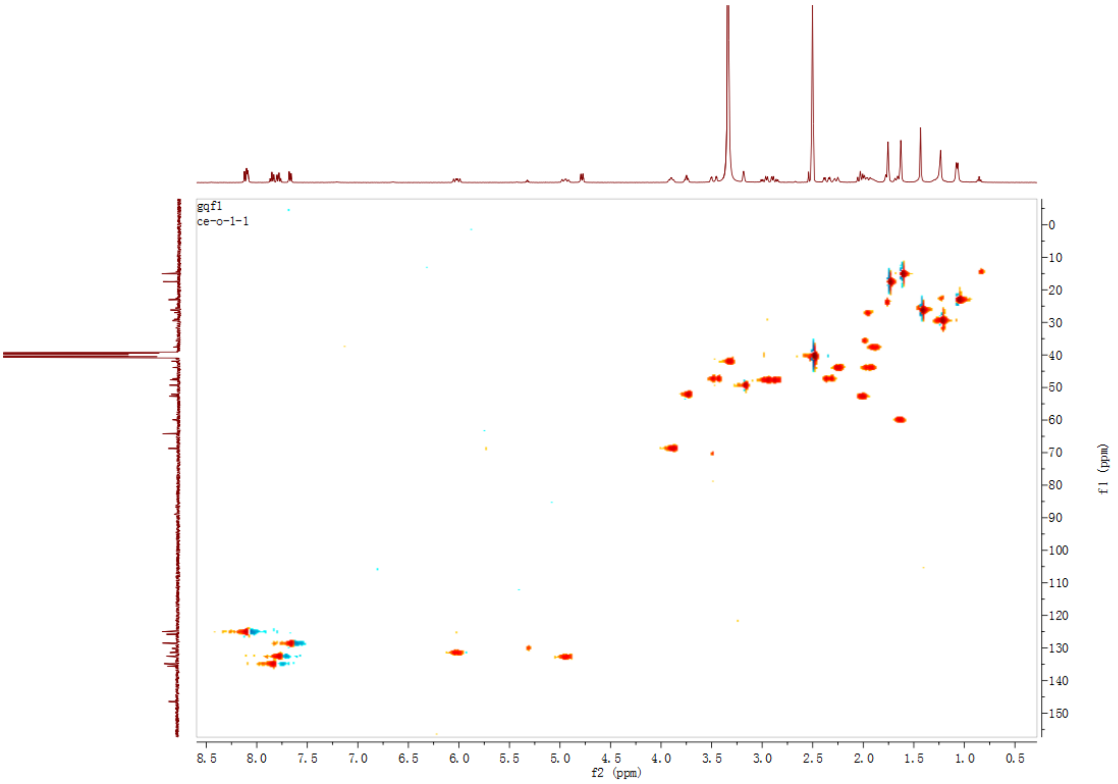

Figure S23. HSQC spectrum of compound **3** in DMSO-d_6 (400 MHz)

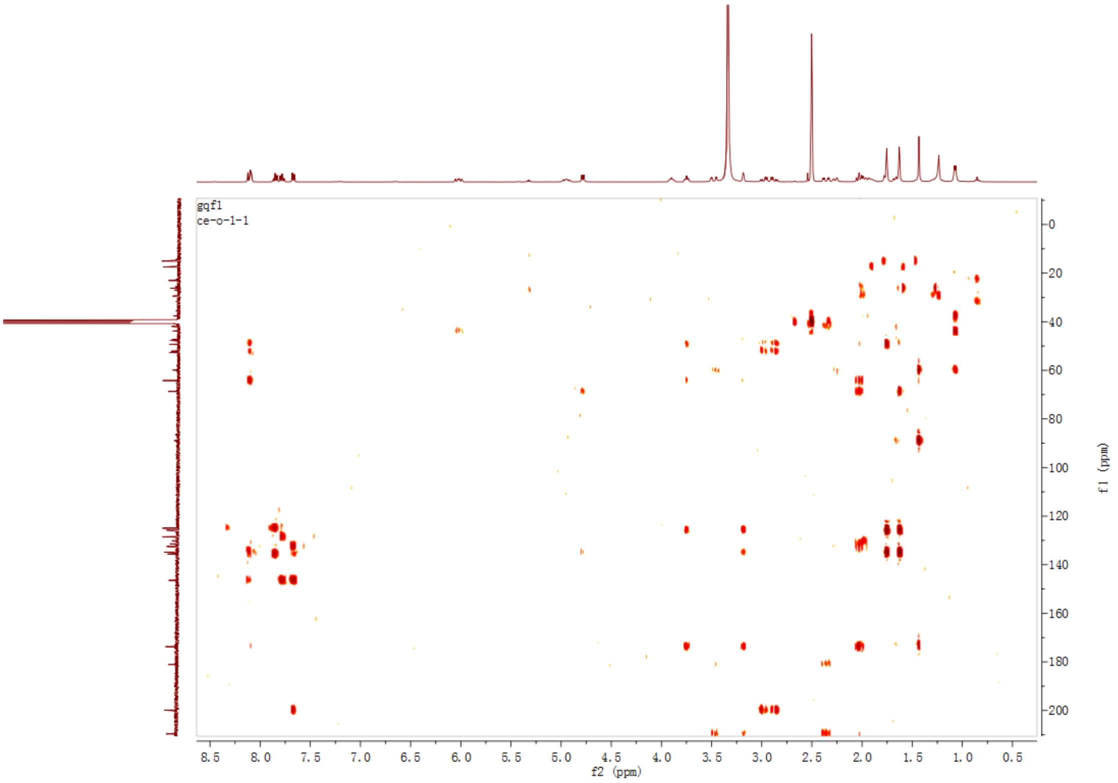

Figure S24. HMBC spectrum of compound **3** in DMSO-d_6 (400 MHz)

Spectrum Plot Report

Agilent

Name	Sample7	Rack Pos.		Instrument	Instrument 1	Operator	
Inj. Vol. (ul)	5	Plate Pos.		IRM Status	Some ions missed		
Data File	CE-0-6R.d	Method (Acq)	CL-2022.m	Comment		Acq. Time (Local)	7/29/2022 4:20:56 PM (UTC+08:00)

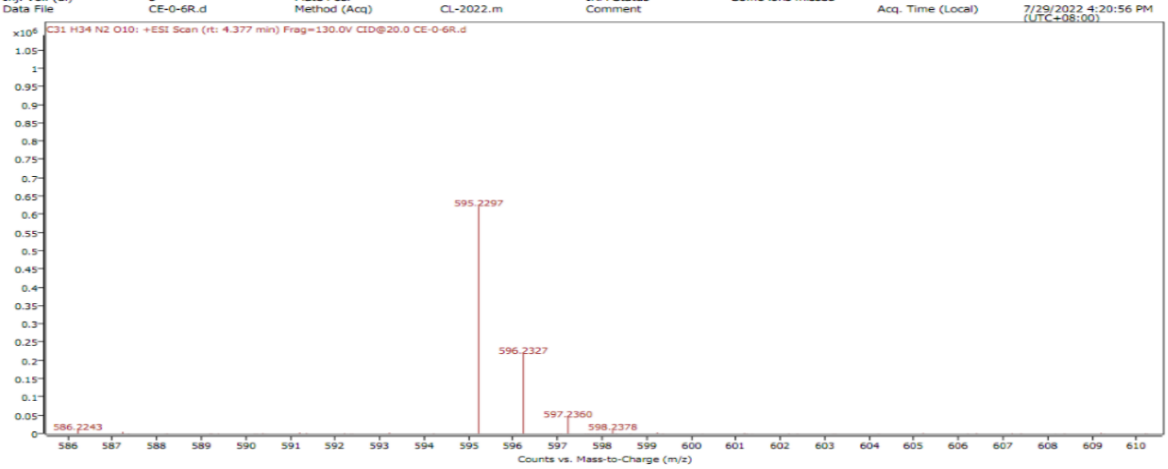

Page 1 of 1 Generated at 10:27 AM on 8/9/2022

Analysis Report

Agilent Trusted Answers

Spectrum Peaks

m/z	Z	Abund	Abund %	m/z (Calc)	Diff (ppm)	Ion Species	Formula	Ion Type
595.2297	1	621068	100.00	595.2286	1.80	(M+H)+	C31 H34 N2 O10	
596.2327	1	218197	35.13	596.2319	1.43	(M+H)+	C31 H34 N2 O10	
597.2360	1	44680	7.19	597.2346	2.43	(M+H)+	C31 H34 N2 O10	
598.2378	1	8053	1.30	598.2372	0.94	(M+H)+	C31 H34 N2 O10	
103.0750		12232	1.97					
121.0509		16493	2.66					

Spectrum Identification Table

Best	ID Source	Name	Formula	Species	m/z	Diff (ppm)	CAS	Score	Score (Lib)	Score (DB)	Score (MFG)	Lib/DB
Yes	MFG		C31 H34 N2 O10	(M+H)+	595.2297	1.74		98.01			98.01	
No	MFG		C32 H30 N6 O6	(M+H)+	595.2297	-0.39		99.24			99.24	
No	MFG		C31 H24 N13 O	(M+H)+	595.2297	-0.15		98.05			98.05	
No	MFG		C30 H28 N9 O5	(M+H)+	595.2297	1.99		97.38			97.38	
No	MFG		C29 H22 N16	(M+H)+	595.2297	2.24		95.44			95.44	

MassHunter Qual 10.0
(End of Report)

Figure S25. HRESIMS spectrum of compound **3**

打印窗口 80: 峰的顶点质谱3.345 的 2022-09-2715-20-17CE-0-1-1.D

峰的顶点质谱3.345 的 2022-09-2715-20-17CE-0-1-1.D

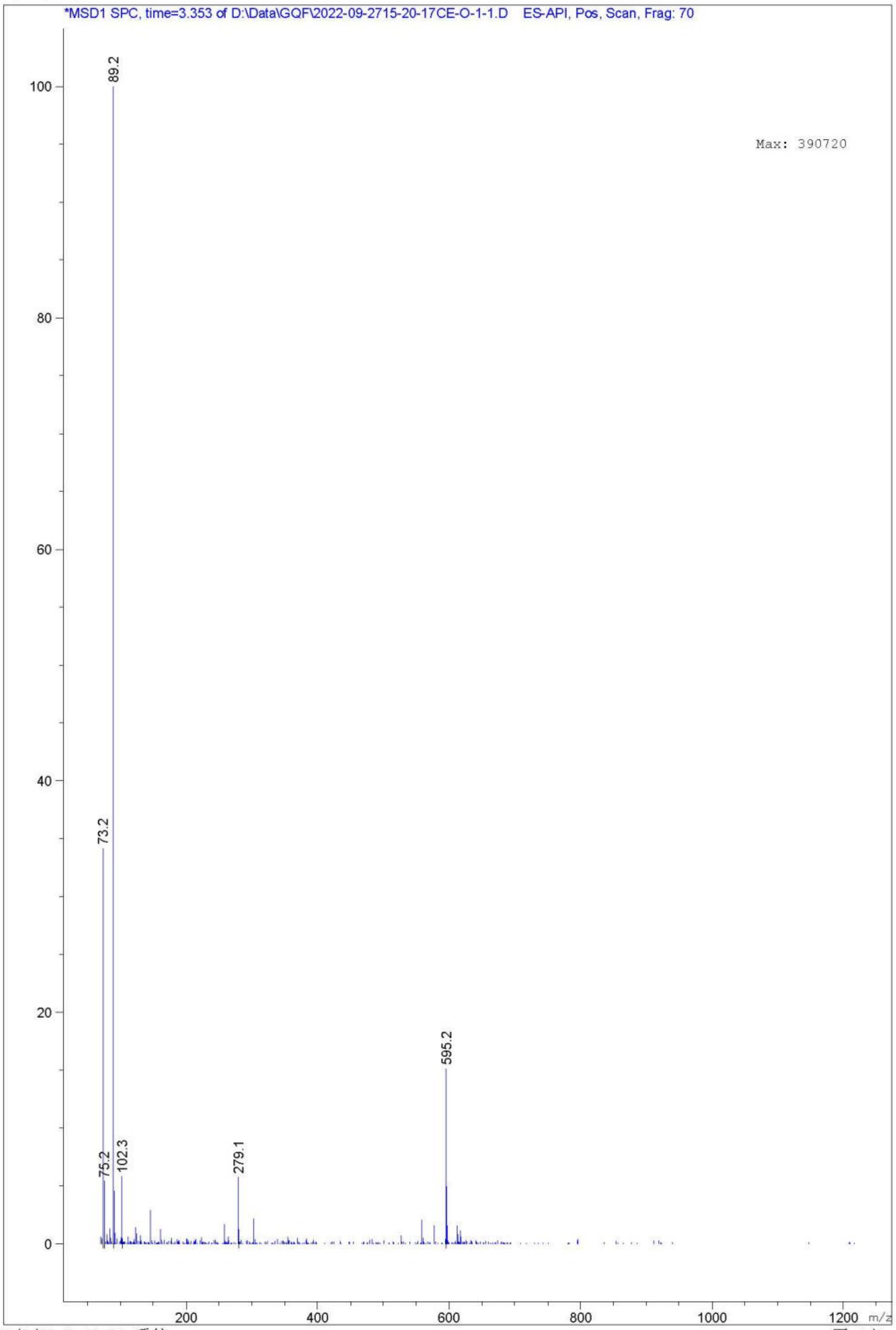

6120 2022/9/28 9:40:34 系统 页 1/1

Figure S26. EIMS spectrum of Compound **3**

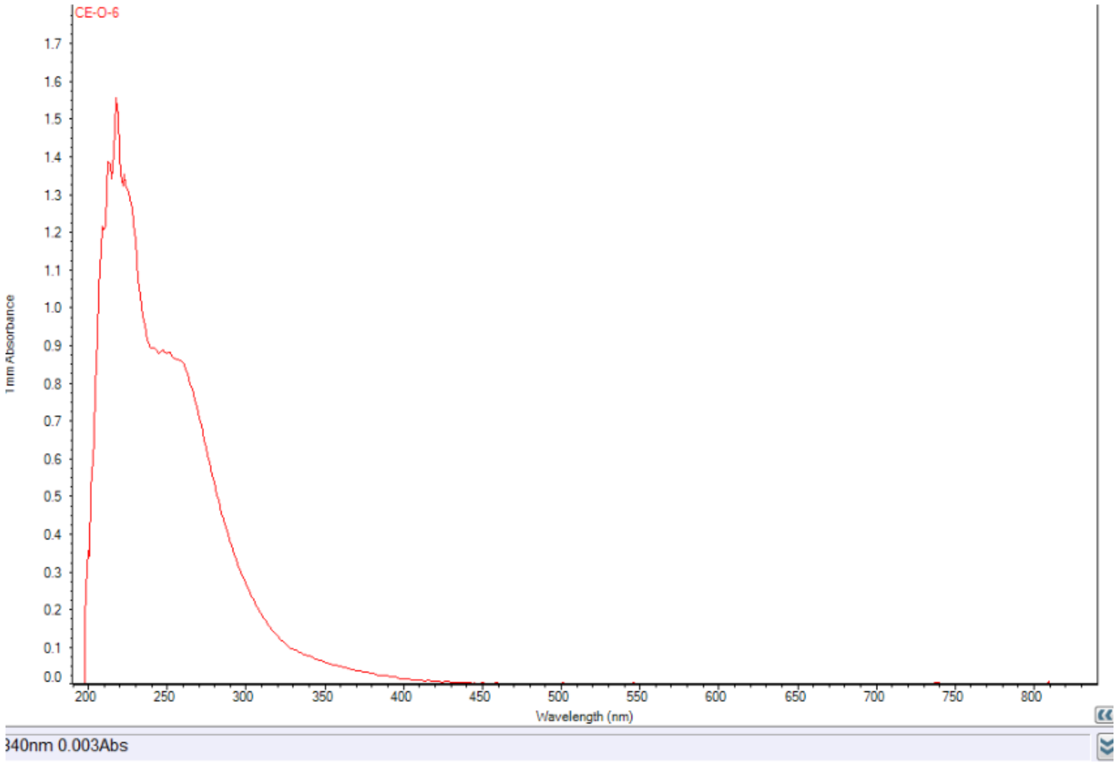

Figure S27. UV spectrum of Compound **3**

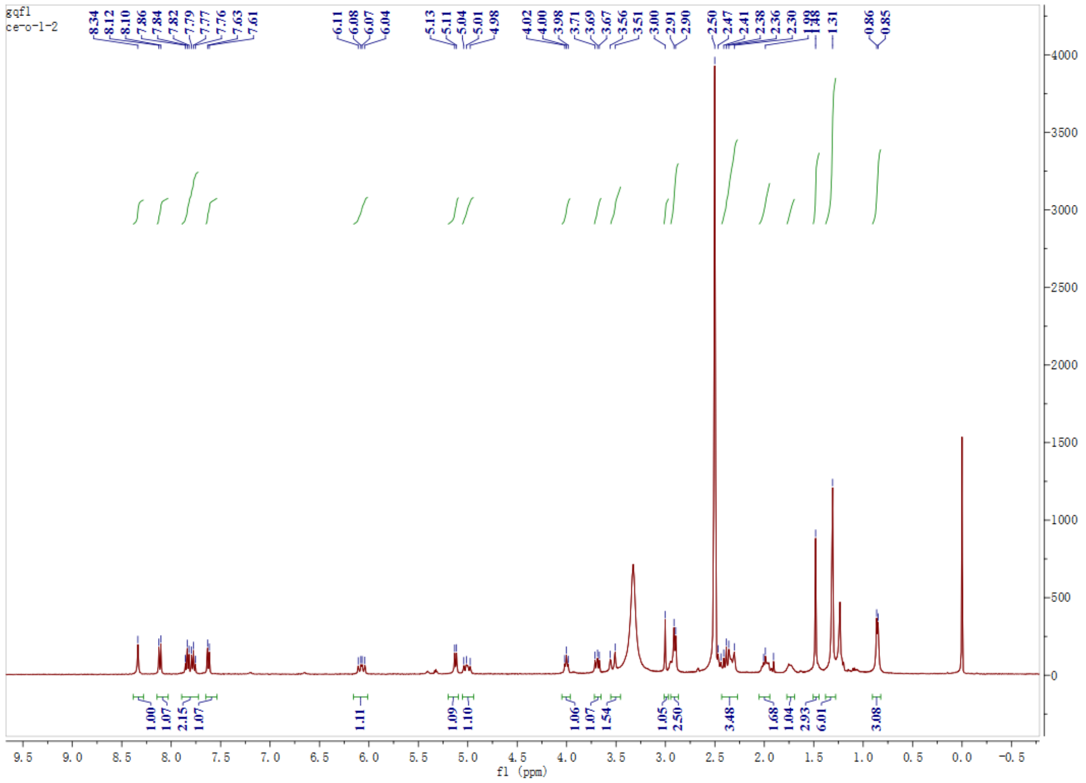

Figure S28. ^{1}H NMR spectrum of compound **4** in DMSO-d_6

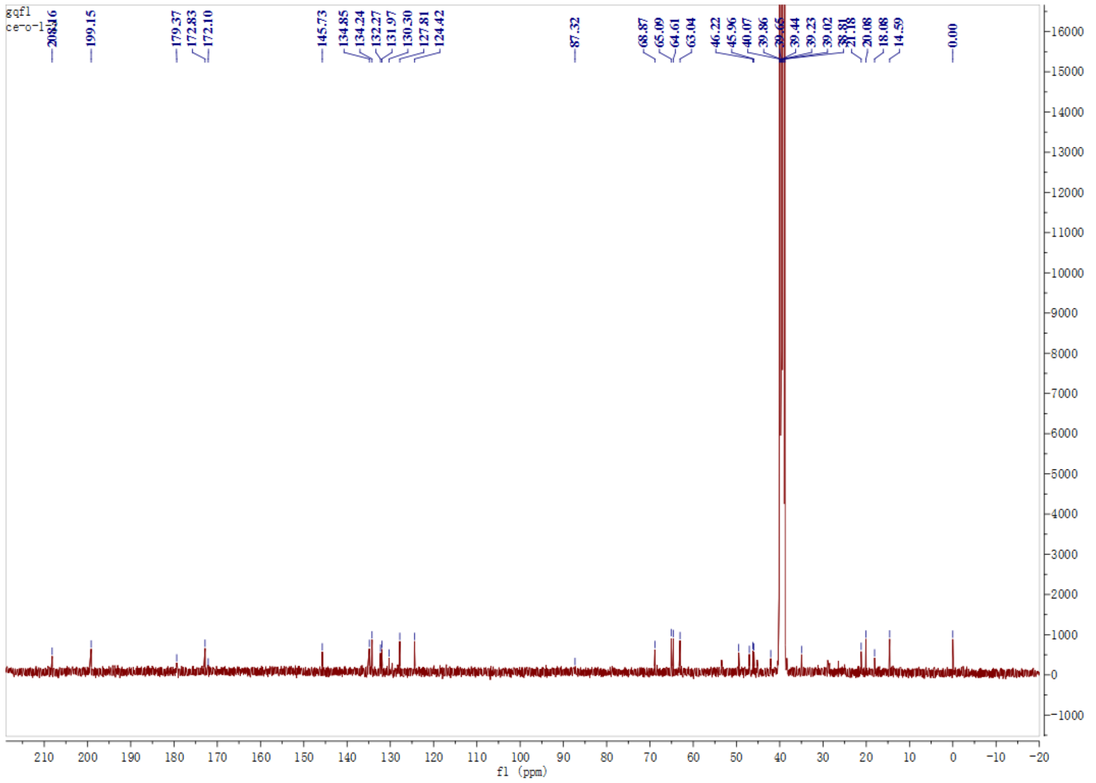

Figure S29. ^{13}C NMR spectrum of compound **4** in DMSO-d_6

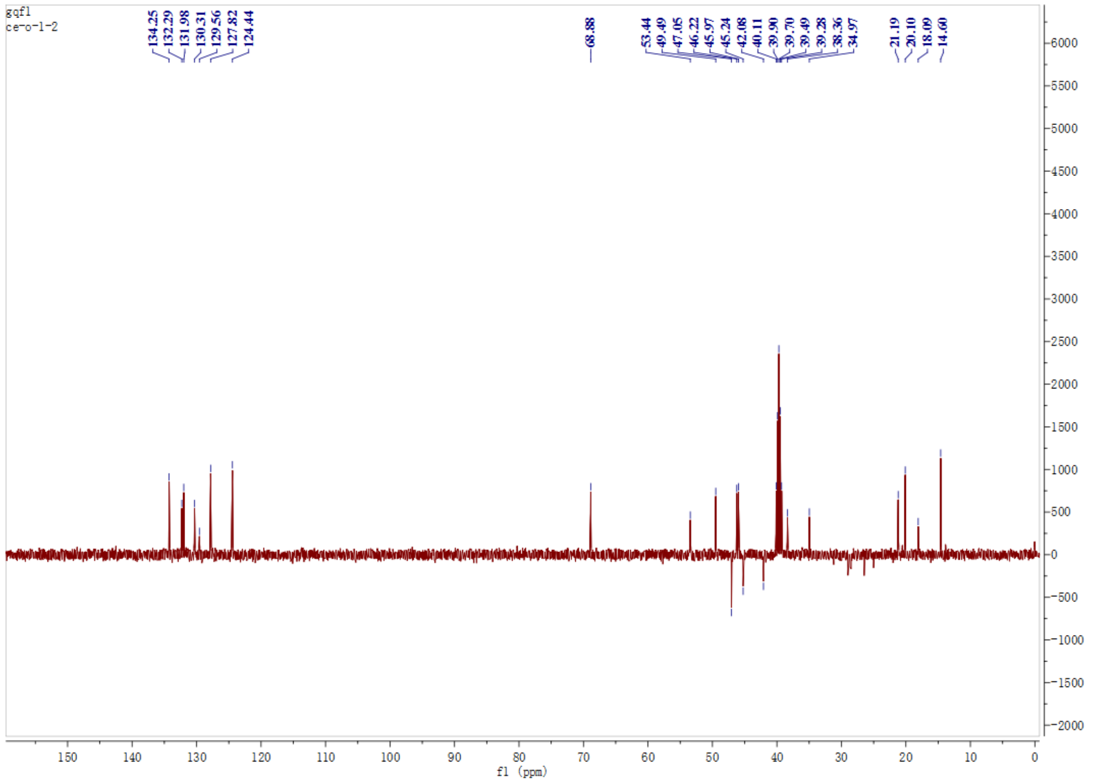

Figure S30. DEPT spectrum of compound **4** in DMSO-d_6

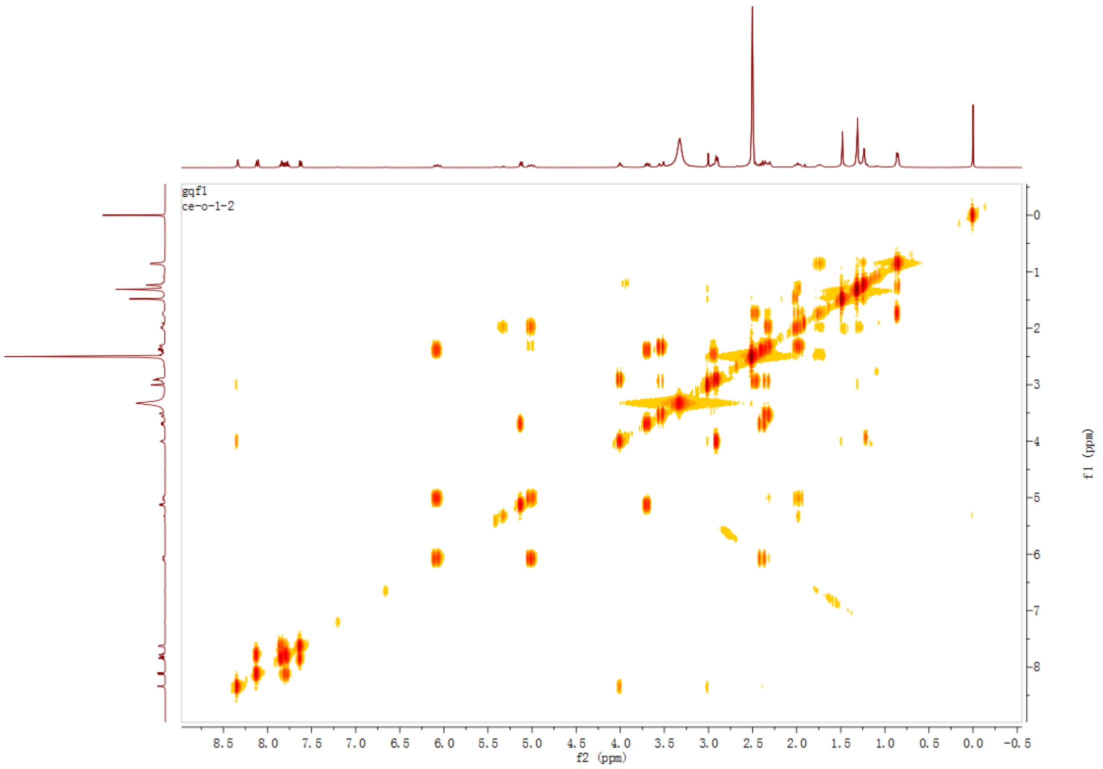

Figure S31. COSY spectrum of compound **4** in DMSO-d_6

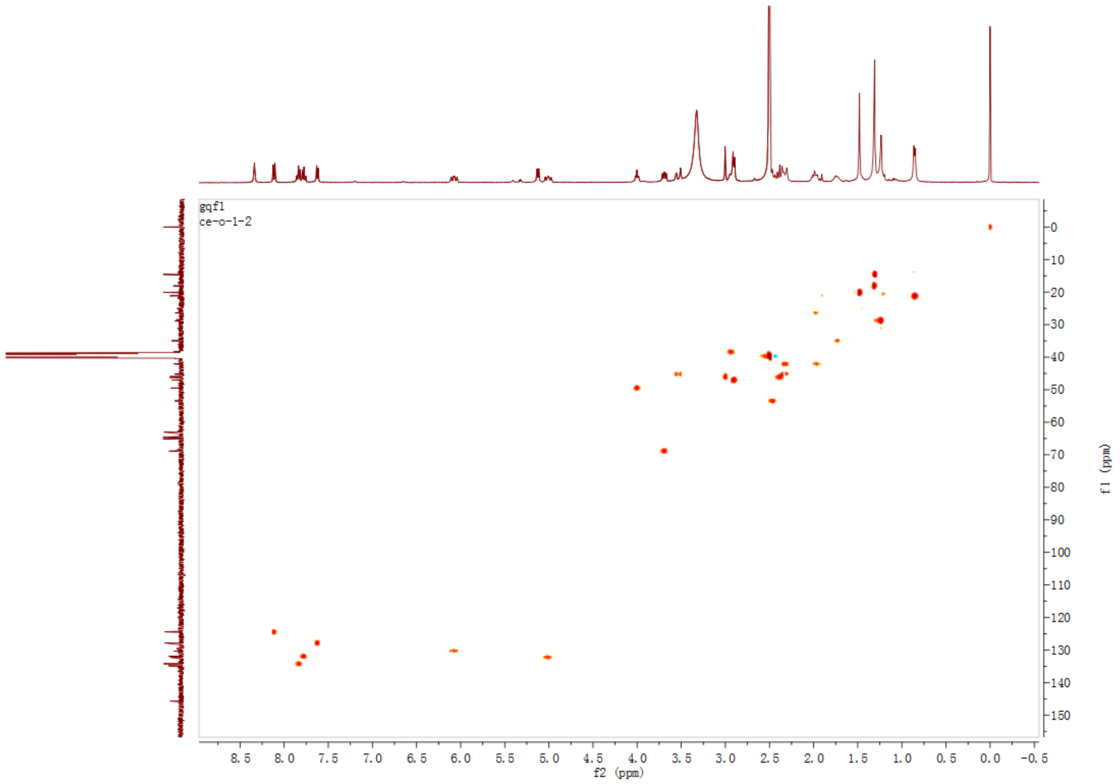

Figure S32. HSQC spectrum of compound **4** in DMSO-d_6

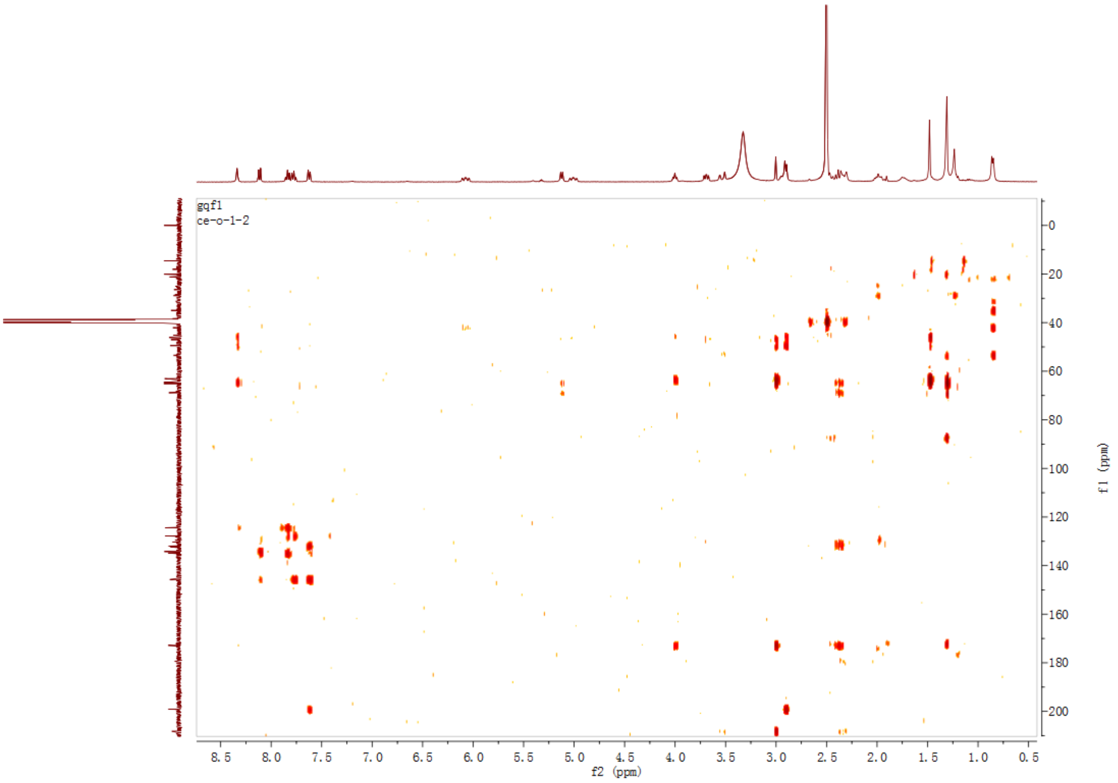

Figure S33. HMBC spectrum of compound **4** in DMSO-d_6

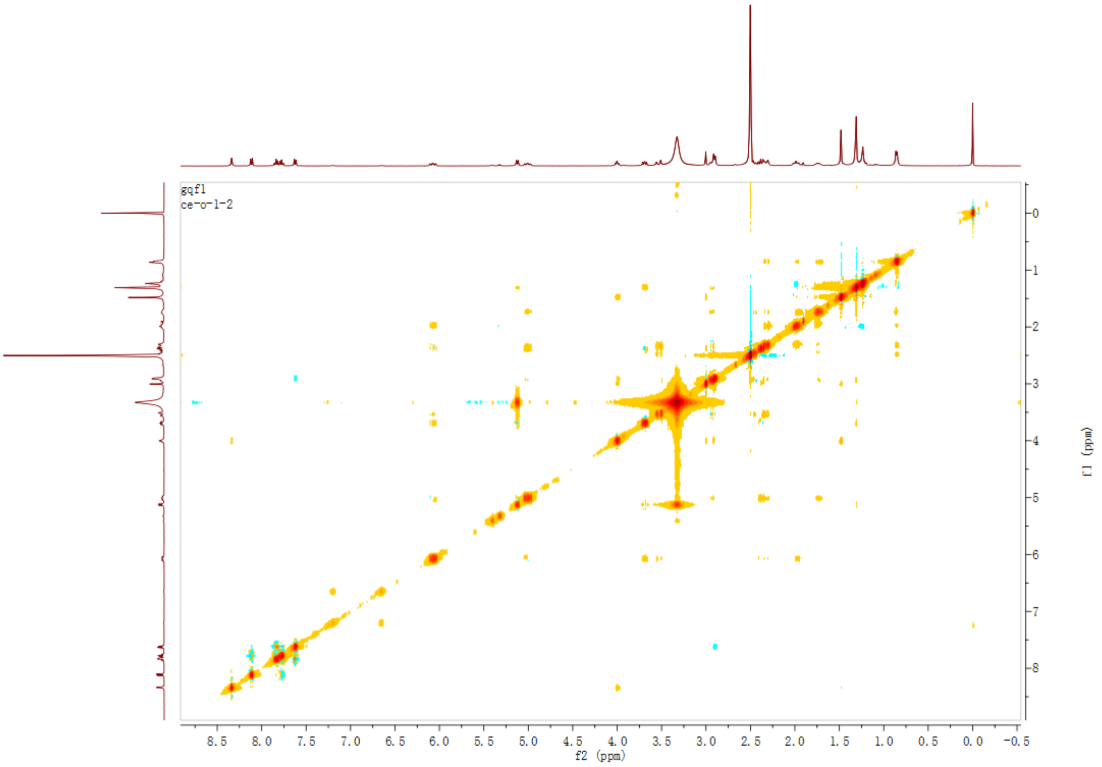

Figure S34. NOESY spectrum of compound **4** in DMSO-d_6

Spectrum Plot Report

Name	Sample6	Rack Pos.		Instrument	Instrument 1	Operator	
Inj. Vol. (ul)	5	Plate Pos.		IRM Status	Some ions missed		
Data File	CE-0-1-2R.d	Method (Acq)	CL-2022.m	Comment		Acq. Time (Local)	7/29/2022 4:07:12 PM (UTC+08:00)

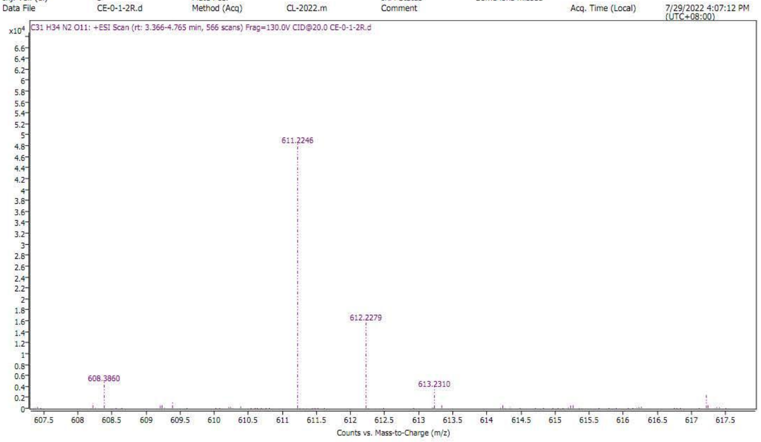

Page 1 of 1 Generated at 10:36 AM on 8/9/2022

Analysis Report

Spectrum Peaks

m/z	Z	Abund	Abund %	m/z (Calc)	Diff (ppm)	Ion Species	Formula	Ion Type
611.2246	1	48434	59.90	611.2235	1.77	(M+H)+	C31 H34 N2 O11	
612.2279	1	16032	19.83	612.2268	1.72	(M+H)+	C31 H34 N2 O11	
613.2310	1	3893	4.81	613.2294	2.51	(M+H)+	C31 H34 N2 O11	
101.0597		4626	5.72					
102.0674		3289	4.07					
103.0392		3711	4.59					
103.0753		7686	9.51					
105.0697		3014	3.73					
107.0852		4078	5.04					
109.1010		3870	4.79					
119.0851		2462	3.04					
120.0442		3984	4.93					
121.0509		23315	28.83					
121.1004		3723	4.60					
131.0704		6757	8.36					

Spectrum Identification Table

Best	ID Source	Name	Formula	Species	m/z	Diff (ppm)	CAS	Score	Score (Lib)	Score (DB)	Score (MFG)	Lib/DB
Yes	MFG		C31 H34 N2 O11	(M+H)+	611.2246	1.81		97.51			97.51	
No	MFG		C32 H30 N6 O7	(M+H)+	611.2246	-0.27		97.39			97.39	
No	MFG		C30 H28 N9 O6	(M+H)+	611.2246	2.05		95.54			95.54	
No	MFG		C31 H24 N13 O2	(M+H)+	611.2246	-0.04		95.06			95.06	
No	MFG		C34 H32 N3 O8	(M+H)+	611.2246	-2.58		92.70			92.70	

MassHunter Qual 10.0
(End of Report)

Figure S35. HRESIMS spectrum of compound **4**

打印窗口 80: 峰的顶点质谱3.206 的 CE-0-1-216-27-45.D

峰的顶点质谱3.206 的 CE-0-1-216-27-45.D

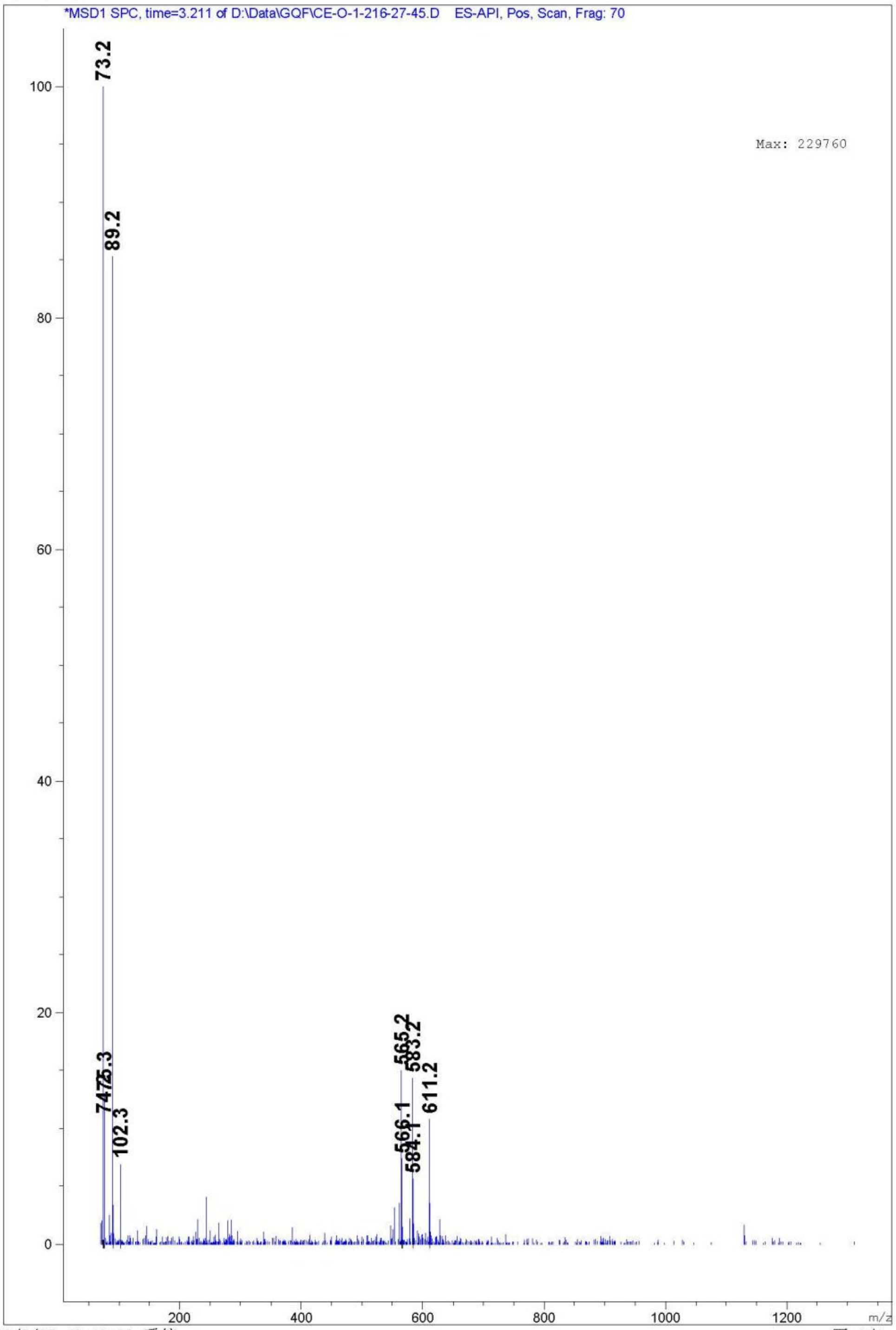

6120 2022/9/27 16:40:08 系统 页 1/1

Figure S36. EIMS spectrum of Compound **4**

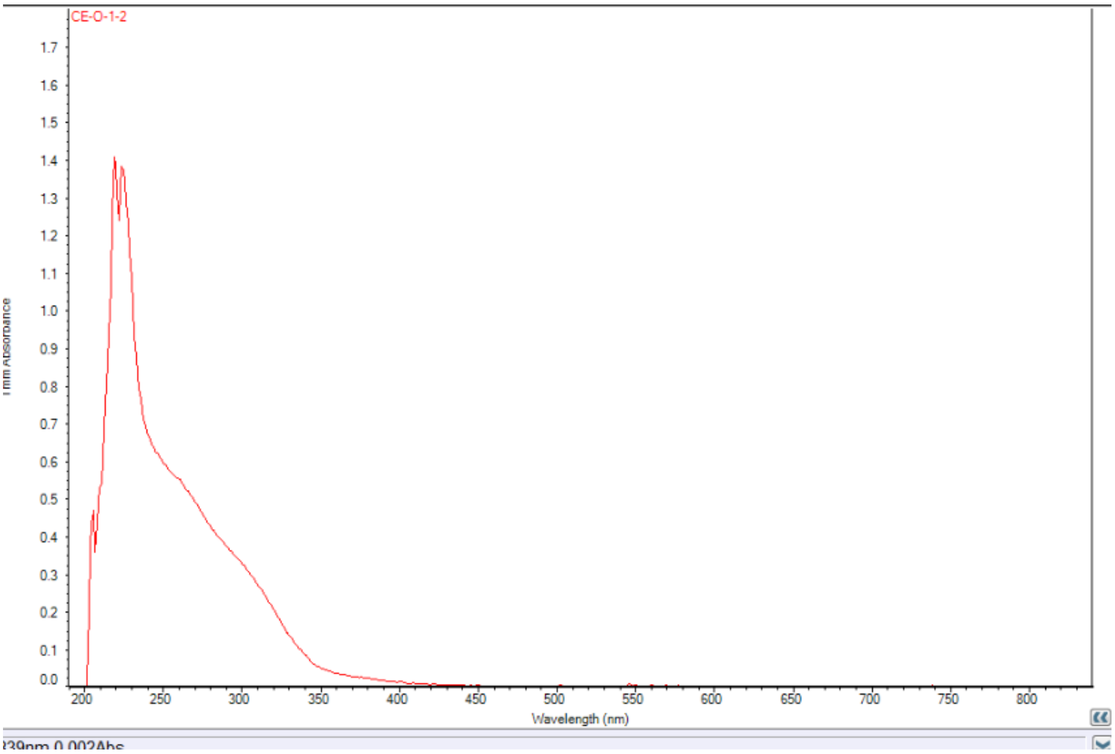

Figure S37. UV spectrum of Compound **4**

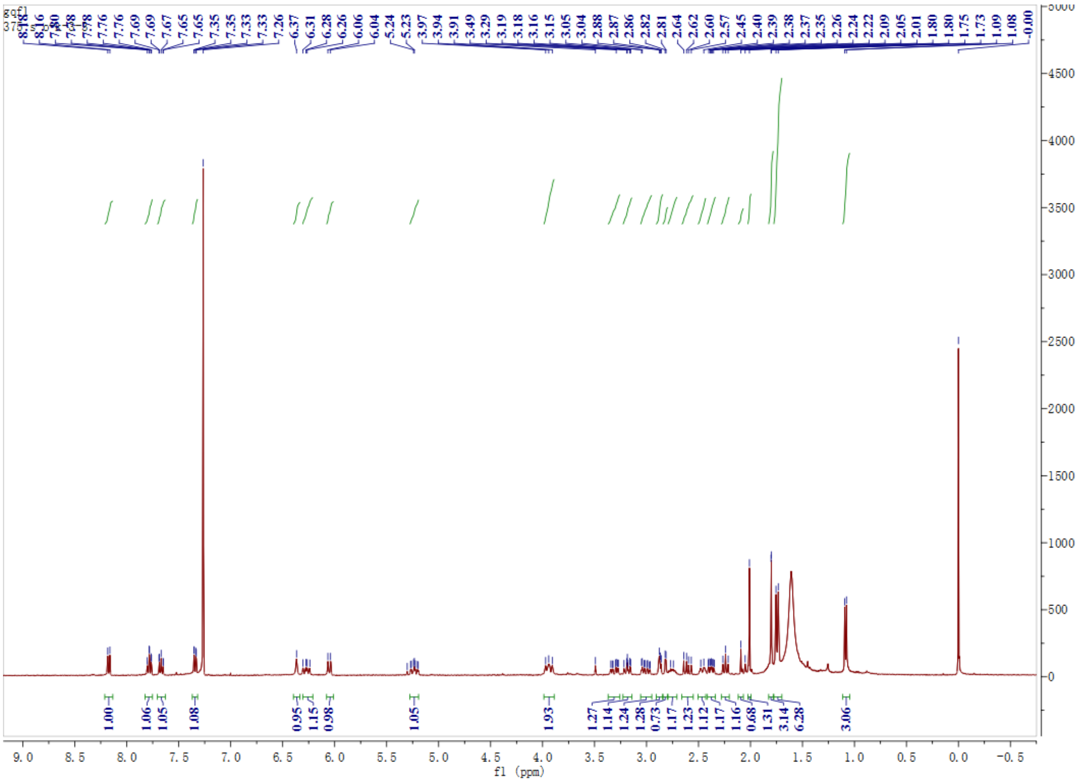

Figure S38. ^{1}H NMR spectrum of compound **5** in $CDCl_3$

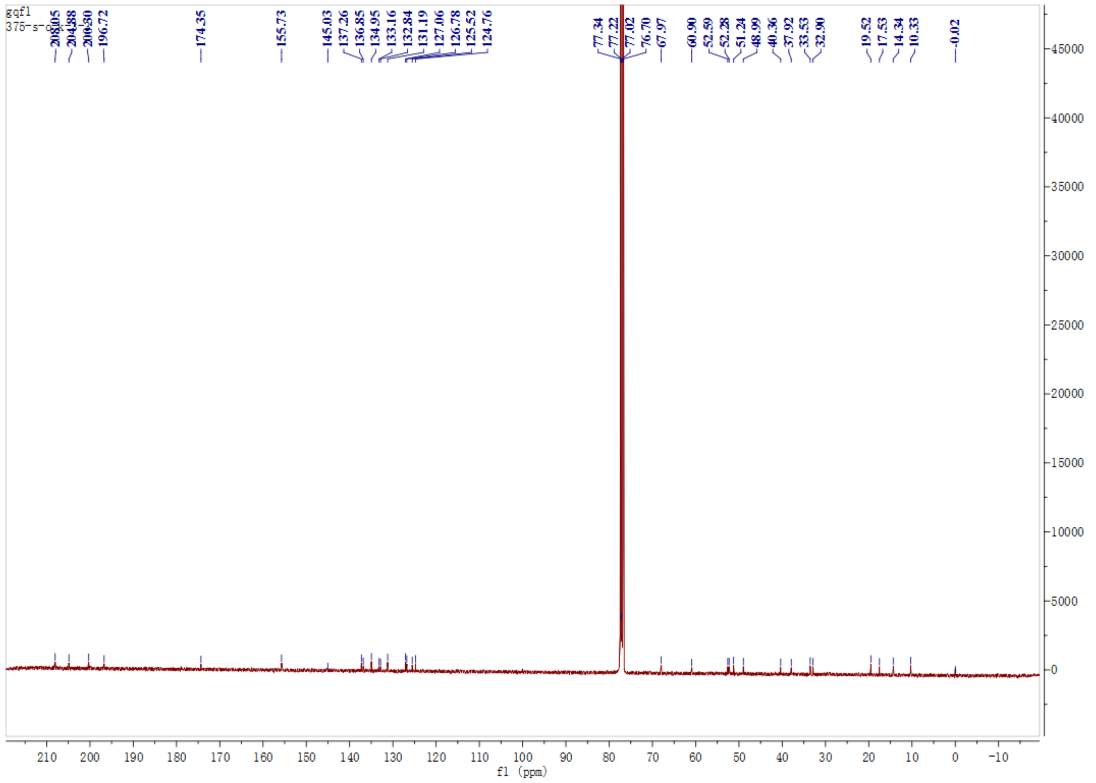

Figure S39. ^{13}C NMR spectrum of compound **5** in $CDCl_3$

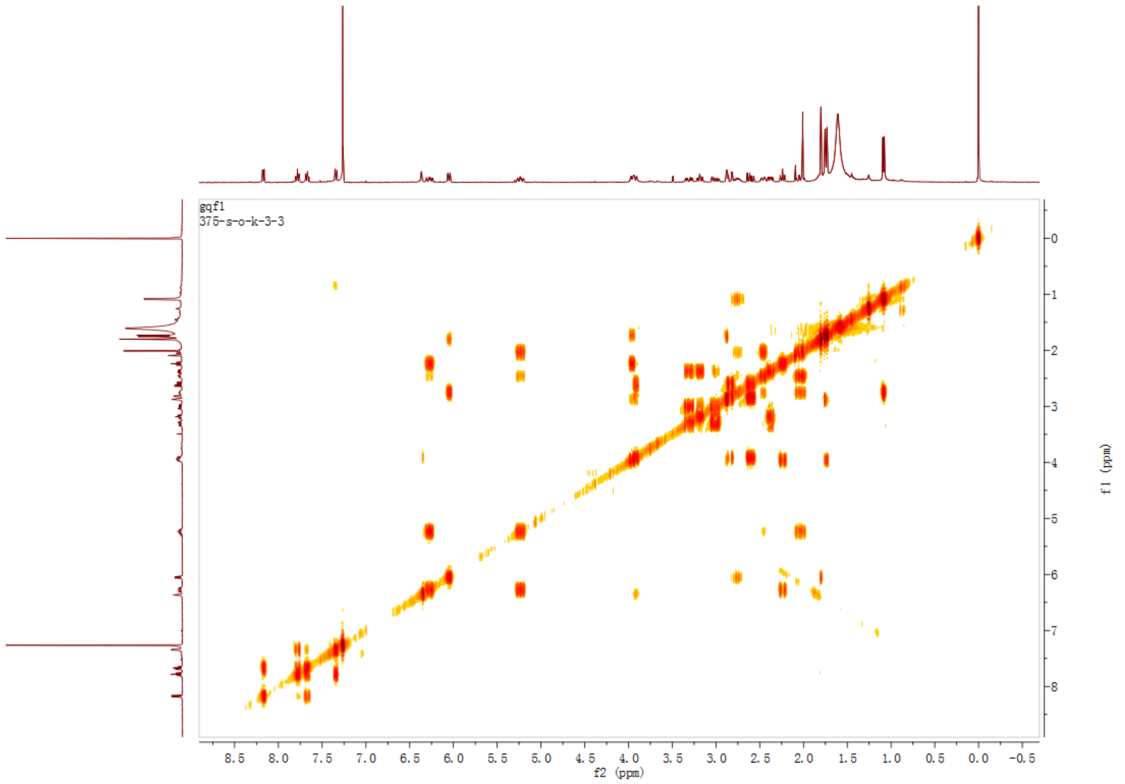

Figure S40. COSY spectrum of compound **5** in $CDCl_3$

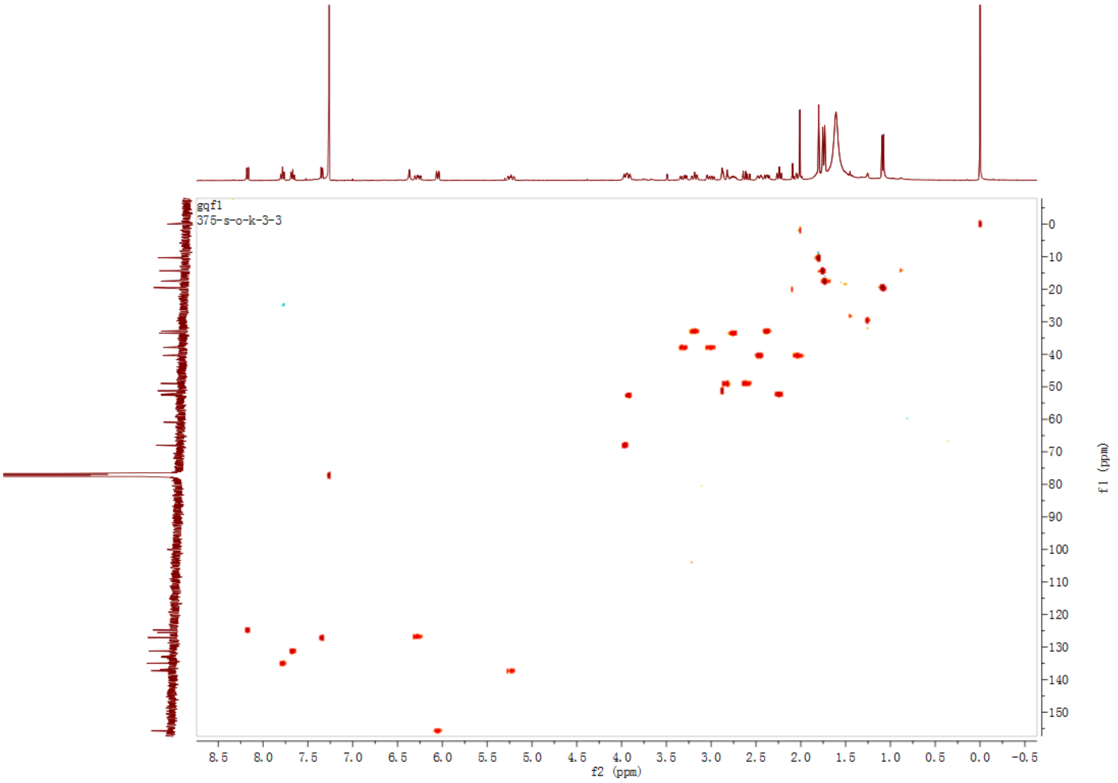

Figure S41. HSQC spectrum of compound **5** in $CDCl_3$

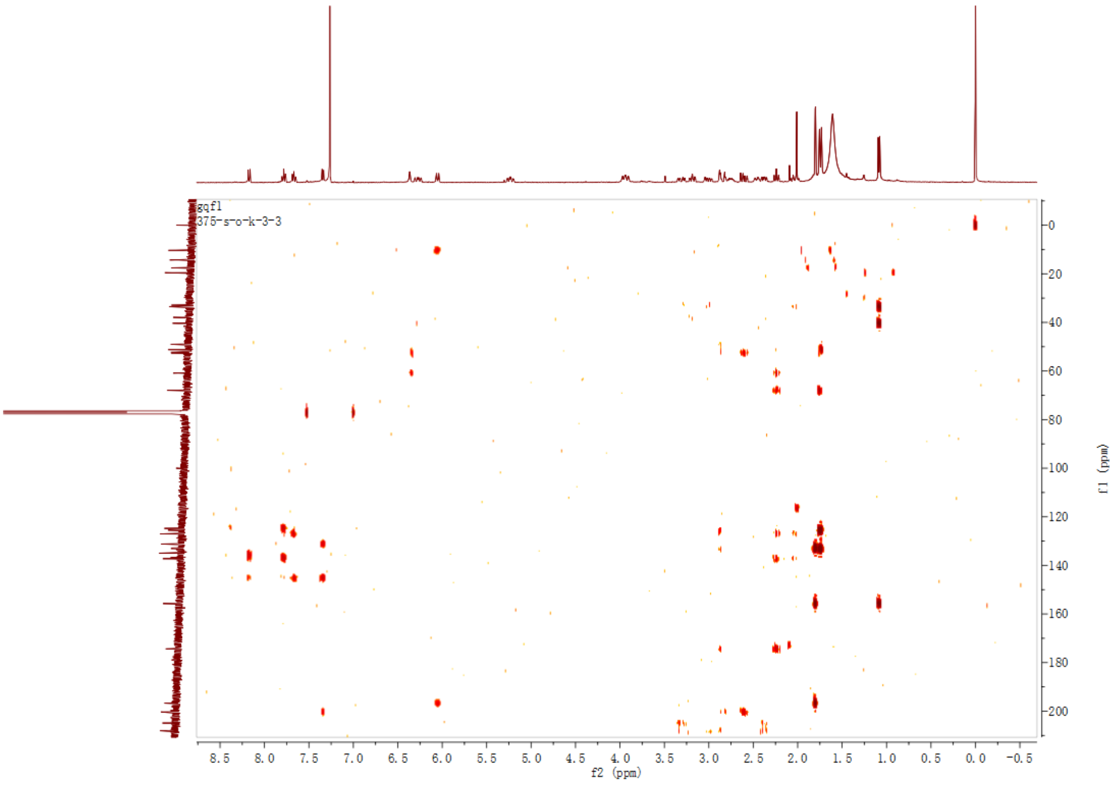

Figure S42. HMBC spectrum of compound **5** in $CDCl_3$

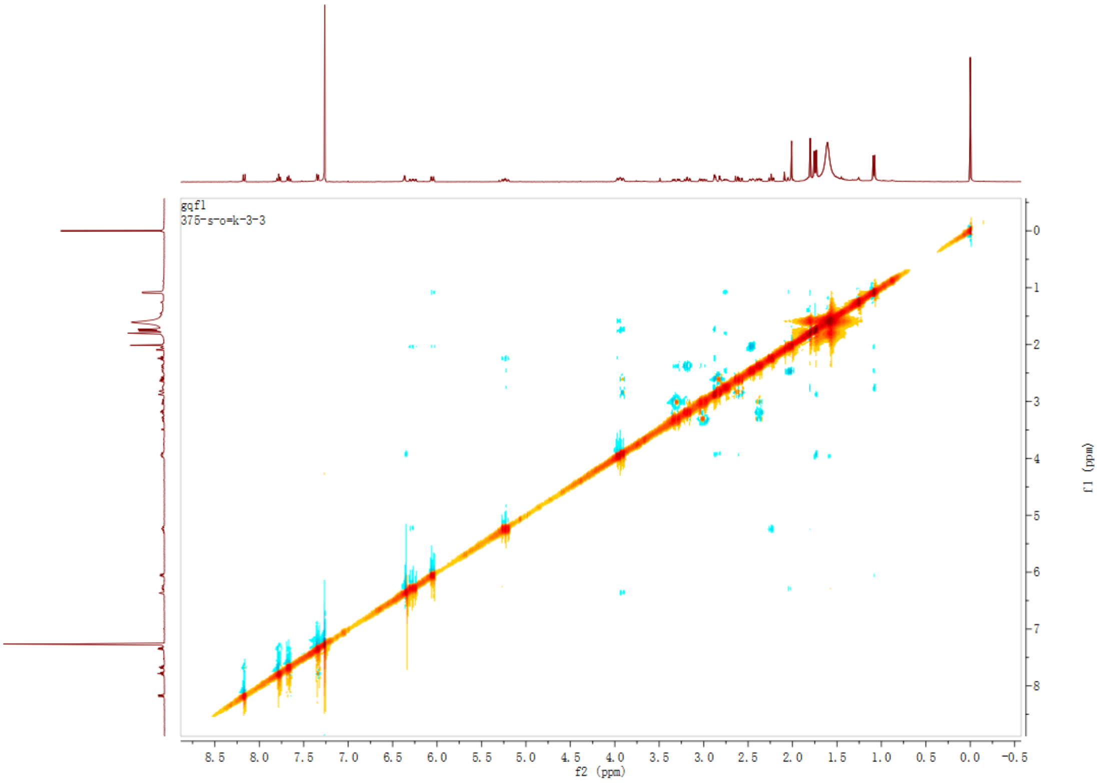

Figure S43. NOESY spectrum of compound **5** in $CDCl_3$

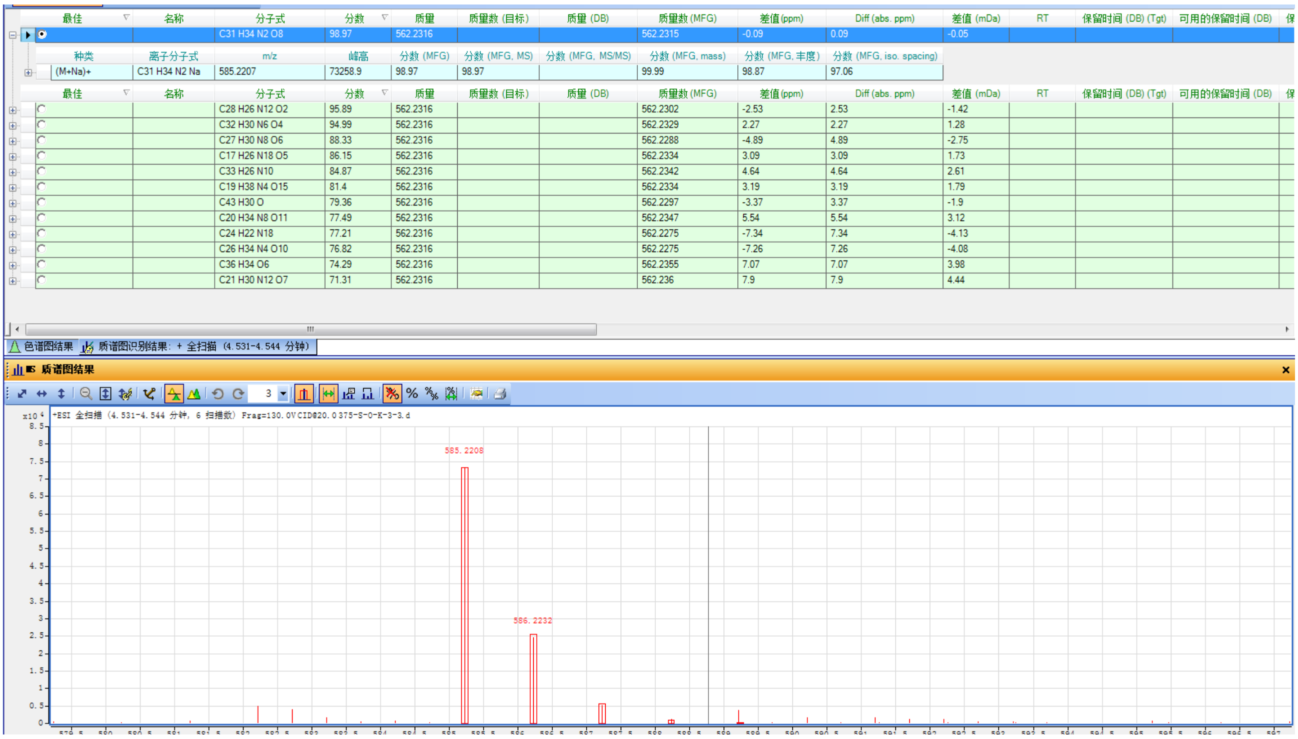

最佳	名称	分子式	分数	质量	质量数 (目标)	质量 (DB)	质量数 (MFG)	差值 (ppm)	Diff (abs. ppm)	差值 (mDa)	RT	保留时间 (DB) (Tgt)	可用的保留时间 (DB)
⦿		C31 H34 N2 O8	98.97	562.2316			562.2315	-0.09	0.09	-0.05			

种类	离子分子式	m/z	峰高	分数 (MFG)	分数 (MFG, MS)	分数 (MFG, MS/MS)	分数 (MFG, mass)	分数 (MFG, 丰度)	分数 (MFG, iso. spacing)
(M+Na)+	C31 H34 N2 Na	585.2207	73258.9	98.97	98.97		99.99	98.87	97.06

最佳	名称	分子式	分数	质量	质量数 (目标)	质量 (DB)	质量数 (MFG)	差值 (ppm)	Diff (abs. ppm)	差值 (mDa)	RT	保留时间 (DB) (Tgt)	可用的保留时间 (DB)
○		C28 H26 N12 O2	95.89	562.2316			562.2302	-2.53	2.53	-1.42			
○		C32 H30 N6 O4	94.99	562.2316			562.2329	2.27	2.27	1.28			
○		C27 H30 N8 O6	88.33	562.2316			562.2288	-4.89	4.89	-2.75			
○		C17 H26 N18 O5	86.15	562.2316			562.2334	3.09	3.09	1.73			
○		C33 H26 N10	84.87	562.2316			562.2342	4.64	4.64	2.61			
○		C19 H38 N4 O15	81.4	562.2316			562.2334	3.19	3.19	1.79			
○		C43 H30 O	79.36	562.2316			562.2297	-3.37	3.37	-1.9			
○		C20 H34 N8 O11	77.49	562.2316			562.2347	5.54	5.54	3.12			
○		C24 H22 N18	77.21	562.2316			562.2275	-7.34	7.34	-4.13			
○		C26 H34 N4 O10	76.82	562.2316			562.2275	-7.26	7.26	-4.08			
○		C36 H34 O6	74.29	562.2316			562.2355	7.07	7.07	3.98			
○		C21 H30 N12 O7	71.31	562.2316			562.236	7.9	7.9	4.44			

Figure S44. HRESIMS spectrum of compound **5**

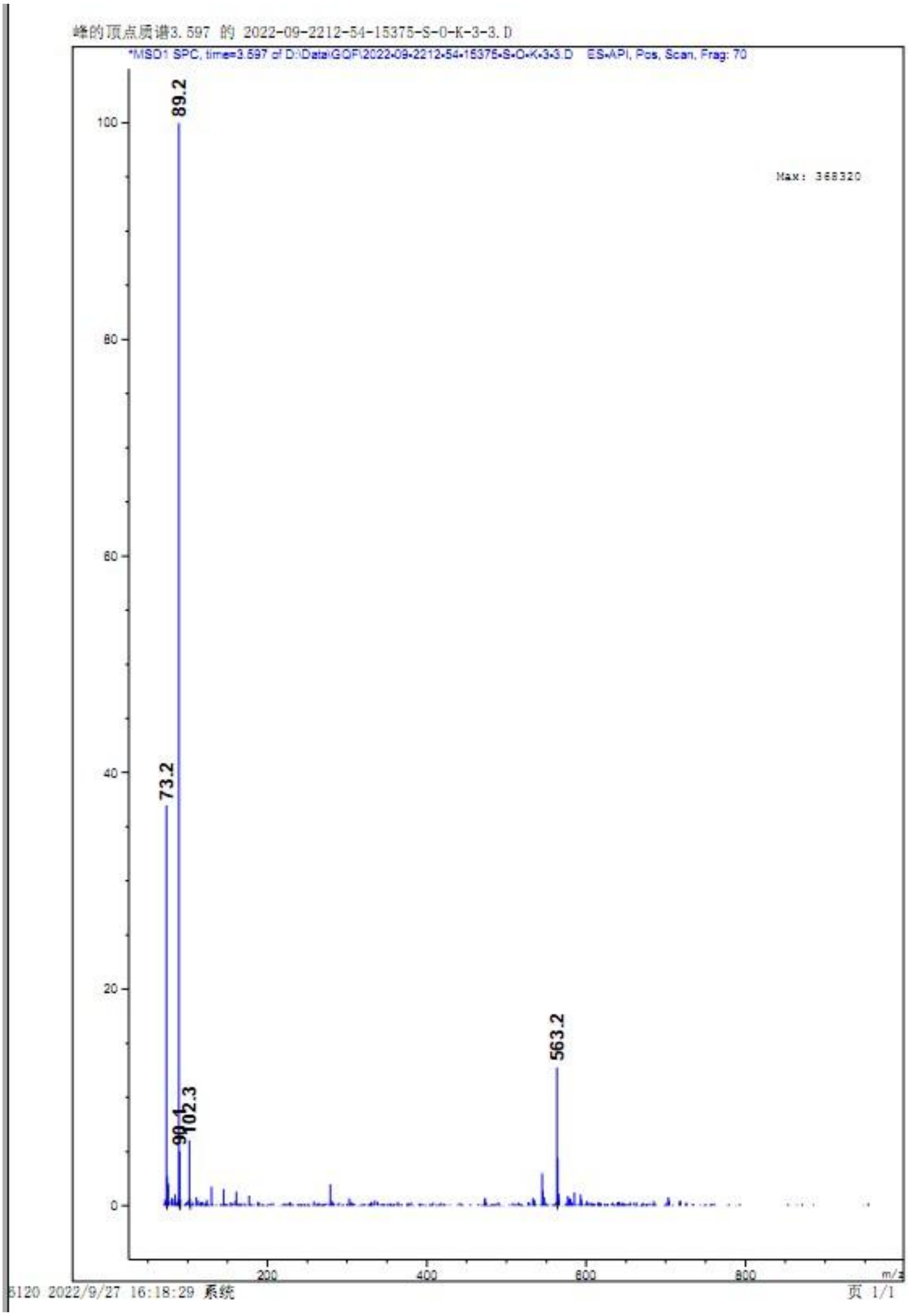

Figure S45. EIMS spectrum of Compound **5**

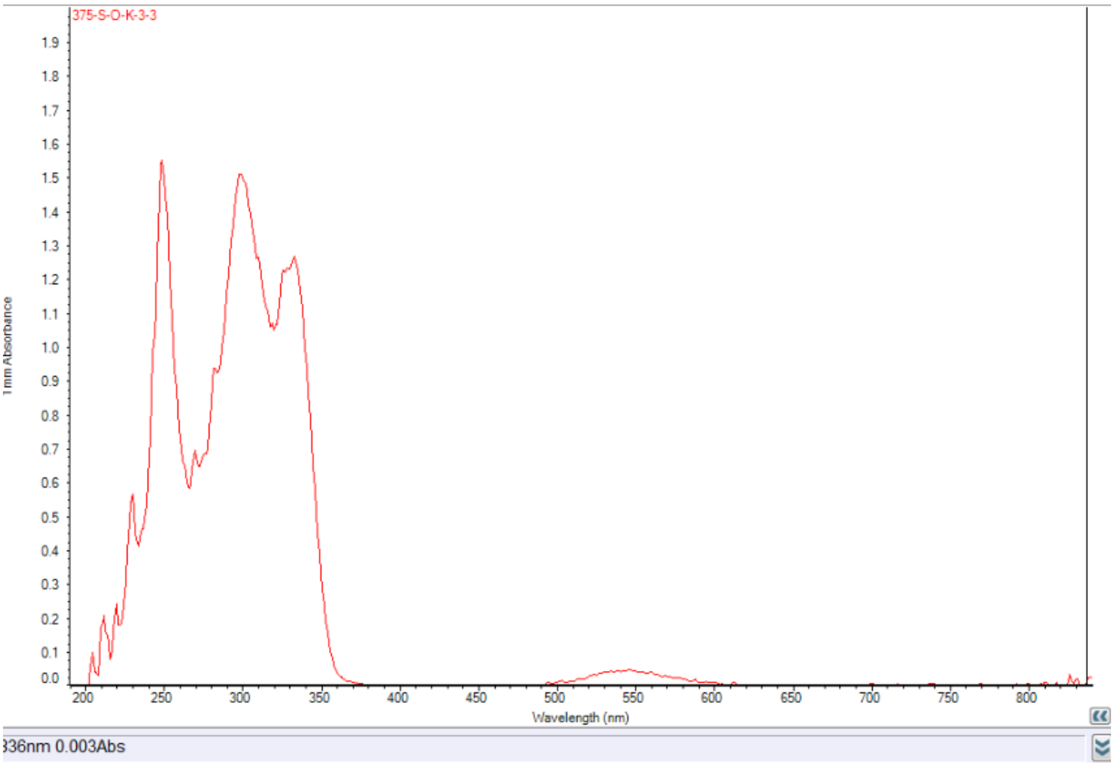

Figure S46. UV spectrum of Compound **5**

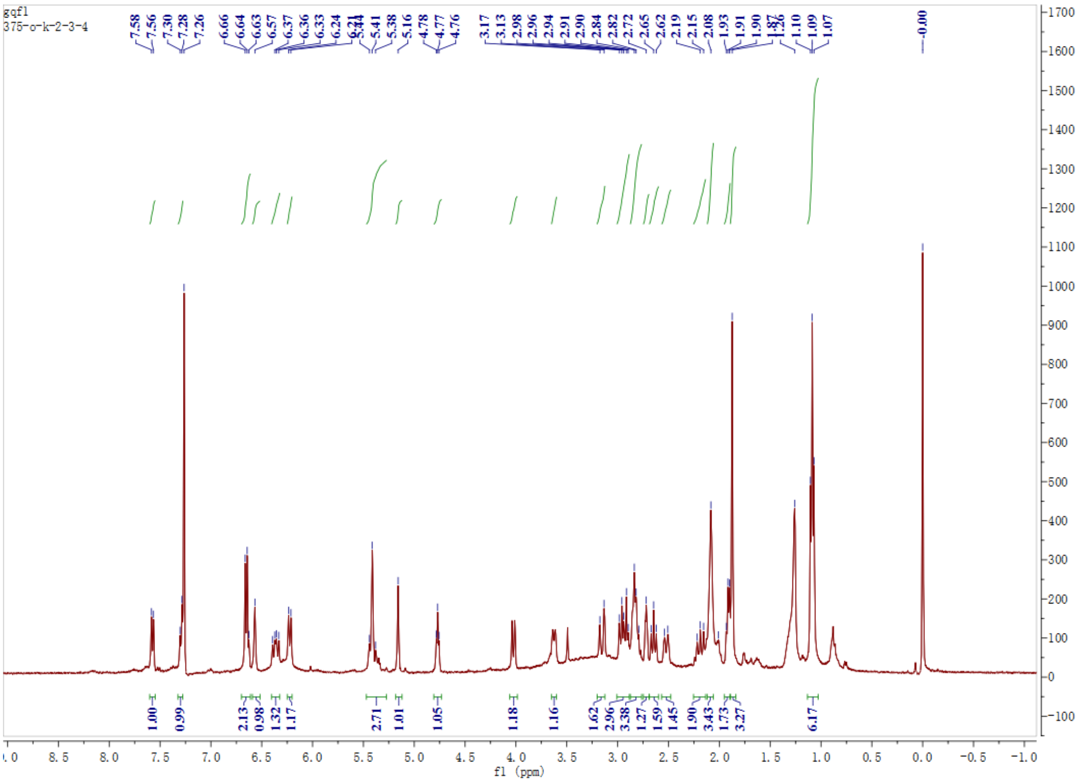

Figure S47. ^{1}H NMR spectrum of compound **6** in $CDCl_3$

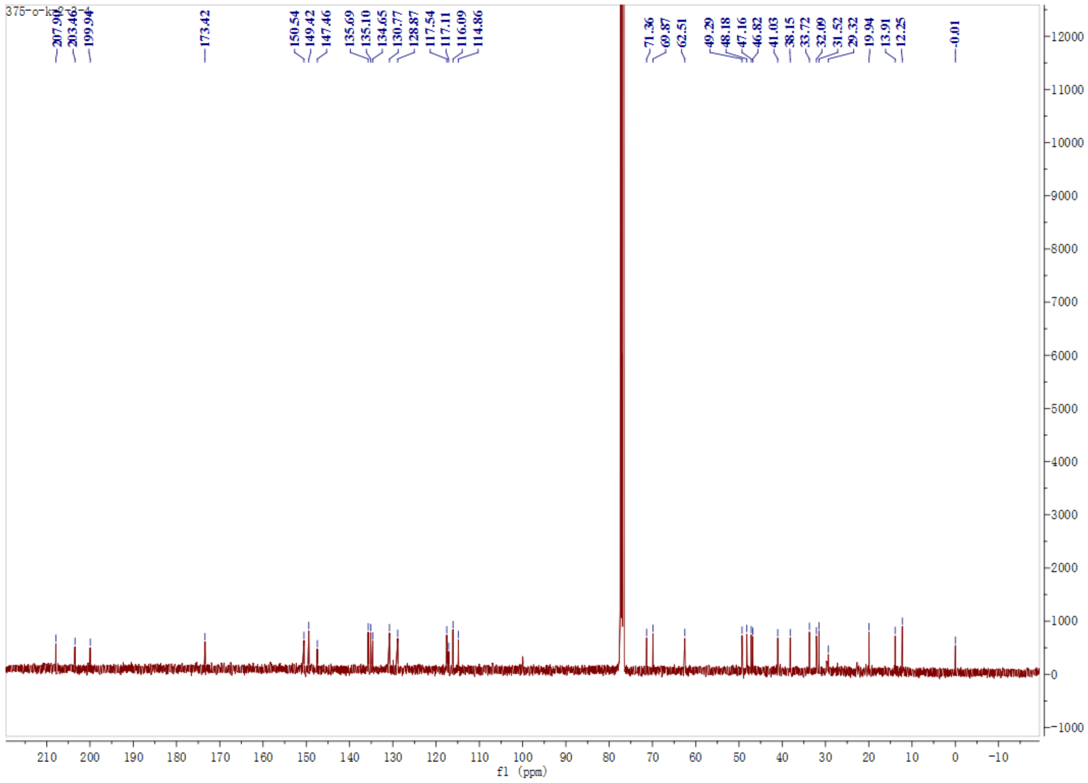

Figure S48. ^{13}C NMR spectrum of compound **6** in $CDCl_3$

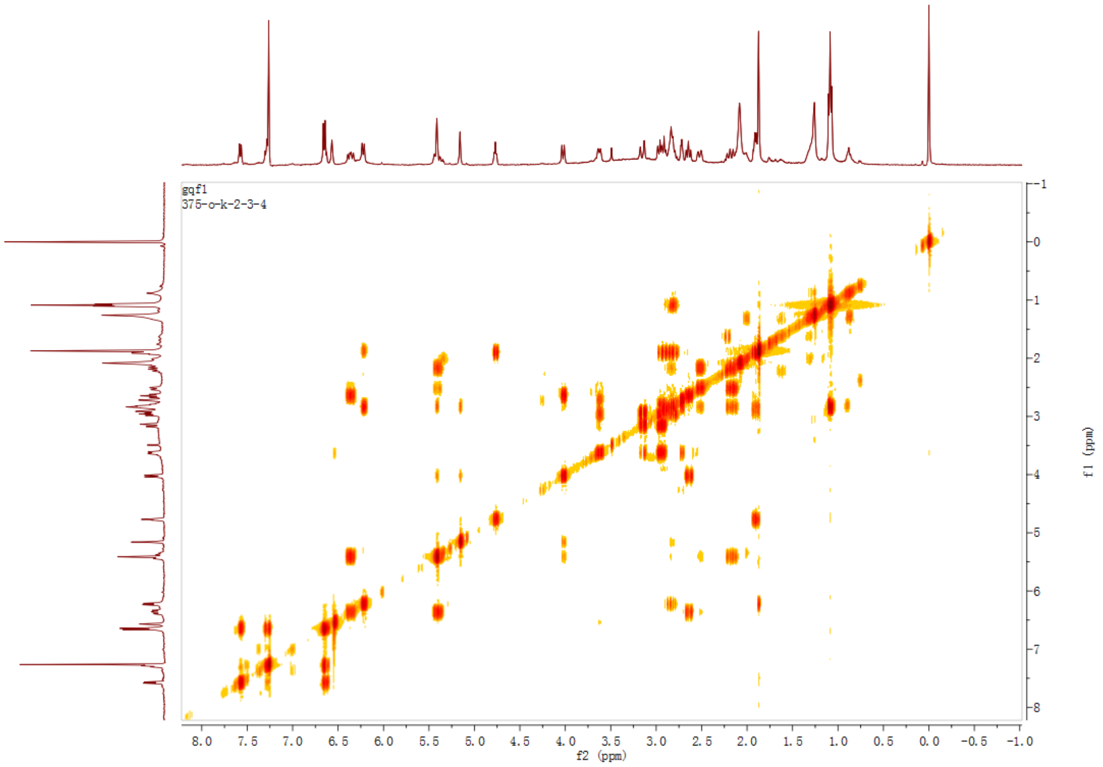

Figure S49. COSY spectrum of compound **6** in $CDCl_3$

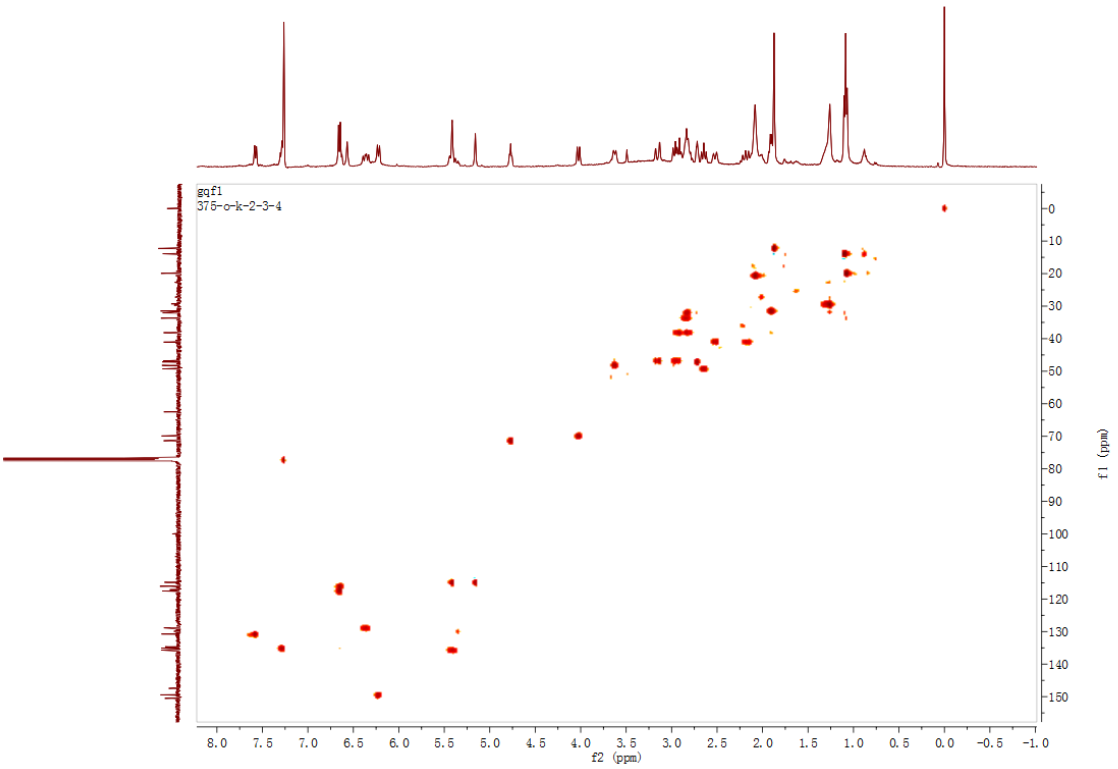

Figure S50. HSQC spectrum of compound **6** in $CDCl_3$

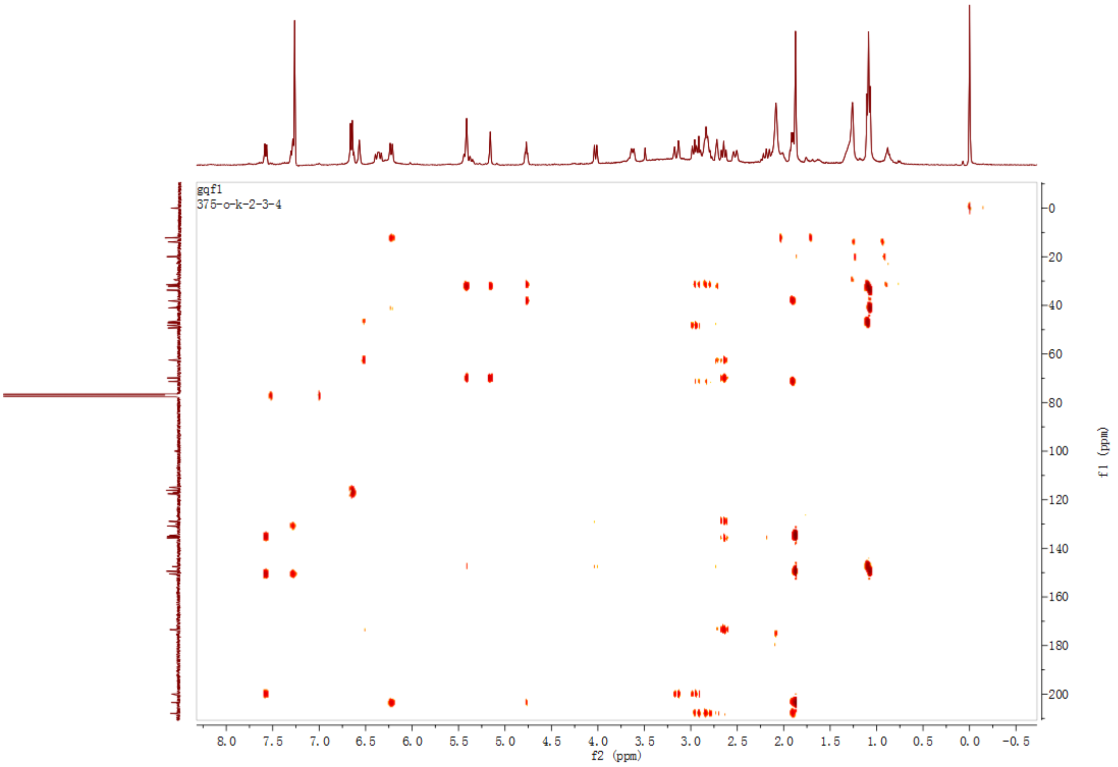

Figure S51. HMBC spectrum of compound **6** in $CDCl_3$

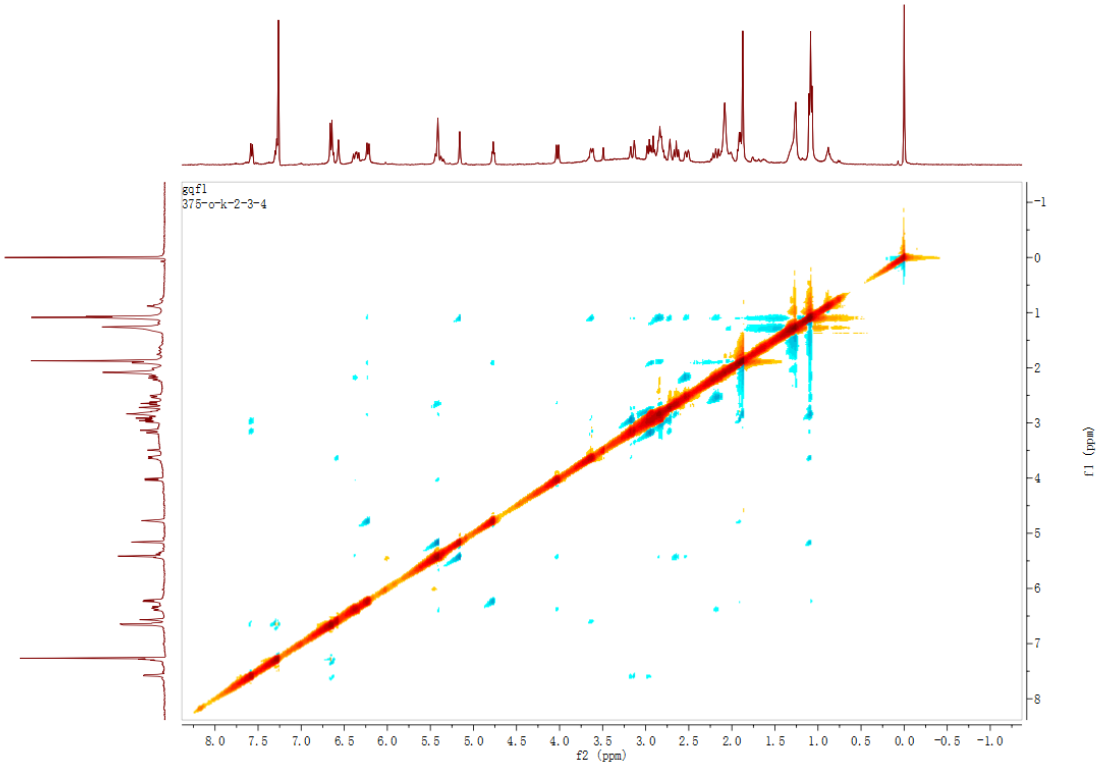

Figure S52. NOESY spectrum of compound **6** in $CDCl_3$

Spectrum Plot Report

Agilent

Name	Sample3	Rack Pos.		Instrument	Instrument 1	Operator	
Inj. Vol. (ul)	5	Plate Pos.		IRM Status	Success		
Data File	375-0-K-2-3-4R.d	Method (Acq)	CL-2022.m	Comment		Acq. Time (Local)	7/29/2022 3:25:58 PM (UTC+08:00)

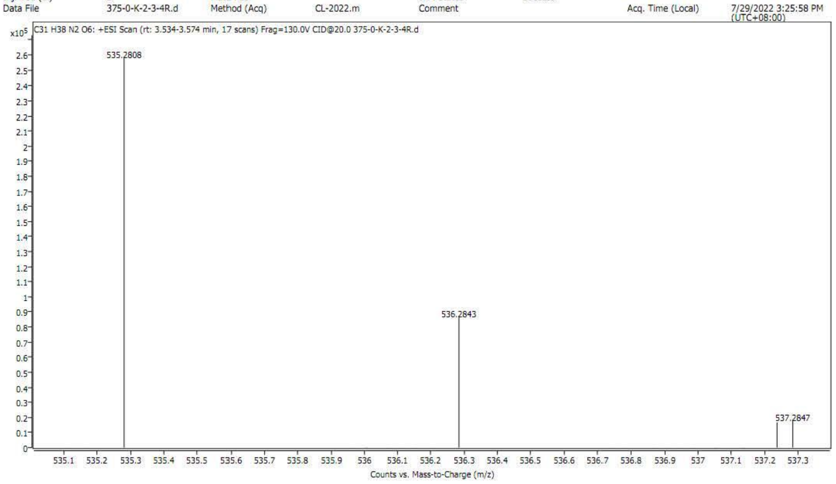

Generated at 1:58 PM on 8/9/2022

Analysis Report

Agilent Trusted Answers

Spectrum Peaks

m/z	Z	Abund	Abund %	m/z (Calc)	Diff (ppm)	Ion Species	Formula	Ion Type
535.2808	1	258077	56.57	535.2803	0.91	(M+H)+	C31 H38 N2 O6	
536.2843	1	86692	19.00	536.2835	1.47	(M+H)+	C31 H38 N2 O6	
537.2847	1	17842	3.91	537.2864	-3.25	(M+H)+	C31 H38 N2 O6	
105.0697		4650	1.02					
107.0854		4884	1.07					
109.0647		30728	6.74					
109.1009		9217	2.02					
117.0907		6531	1.43					
119.0854		6487	1.42					
120.0445		67114	14.71					
121.0509		38292	8.39					
121.1008		7825	1.72					
123.0804		4979	1.09					
134.0601		19810	4.34					
136.0757		36252	7.95					
137.0960		35565	7.80					
146.0600		20989	4.60					
147.0789		5618	1.23					
157.1010		21536	4.72					
163.0763		15574	3.41					
165.0911	1	415456	91.06					
166.0945	1	39869	8.74					
174.0915		9282	2.03					

Spectrum Identification Table

Best	ID Source	Name	Formula	Species	m/z	Diff (ppm)	CAS	Score	Score (Lib)	Score (DB)	Score (MFG)	Lib/DB
Yes	MFG		C31 H38 N2 O6	(M+H)+	535.2808	0.75		98.61			98.61	
No	MFG		C30 H32 N9 O	(M+H)+	535.2808	1.02		97.44			97.44	
No	MFG		C32 H34 N6 O2	(M+H)+	535.2808	-1.62		95.94			95.94	
No	MFG		C29 H36 N5 O5	(M+H)+	535.2808	3.39		93.29			93.29	
No	MFG		C28 H30 N12	(M+H)+	535.2808	3.68		91.45			91.45	
No	MFG		C33 H32 N2 O5	(M+H)+	537.2373	-1.06		79.82			79.82	
No	MFG		C30 H30 N9 O	(M+H)+	533.2646	0.09		79.08			79.08	
No	MFG		C31 H30 N5 O4	(M+H)+	537.2373	1.59		78.55			78.55	
No	MFG		C32 H26 N9	(M+H)+	537.2373	-0.76		77.41			77.41	
No	MFG		C31 H36 N2 O6	(M+H)+	533.2646	-0.13		77.32			77.32	
No	MFG		C28 H28 N12	(M+H)+	533.2646	2.72		76.07			76.07	
No	MFG		C29 H34 N5 O5	(M+H)+	533.2646	2.49		74.80			74.80	
No	MFG		C32 H32 N6 O2	(M+H)+	533.2646	-2.53		74.63			74.63	
No	MFG		C30 H34 N O8	(M+H)+	537.2373	3.94		73.70			73.70	
No	MFG		C19 H34 N7 O11	(M+H)+	537.2373	-1.54		72.53			72.53	

MassHunter Qual 10.0
(End of Report)

Figure S53. HRESIMS spectrum of compound **6**

打印窗口 80: 峰的顶点质谱3.346 的 375-0-K-2-3-409-05-49.D

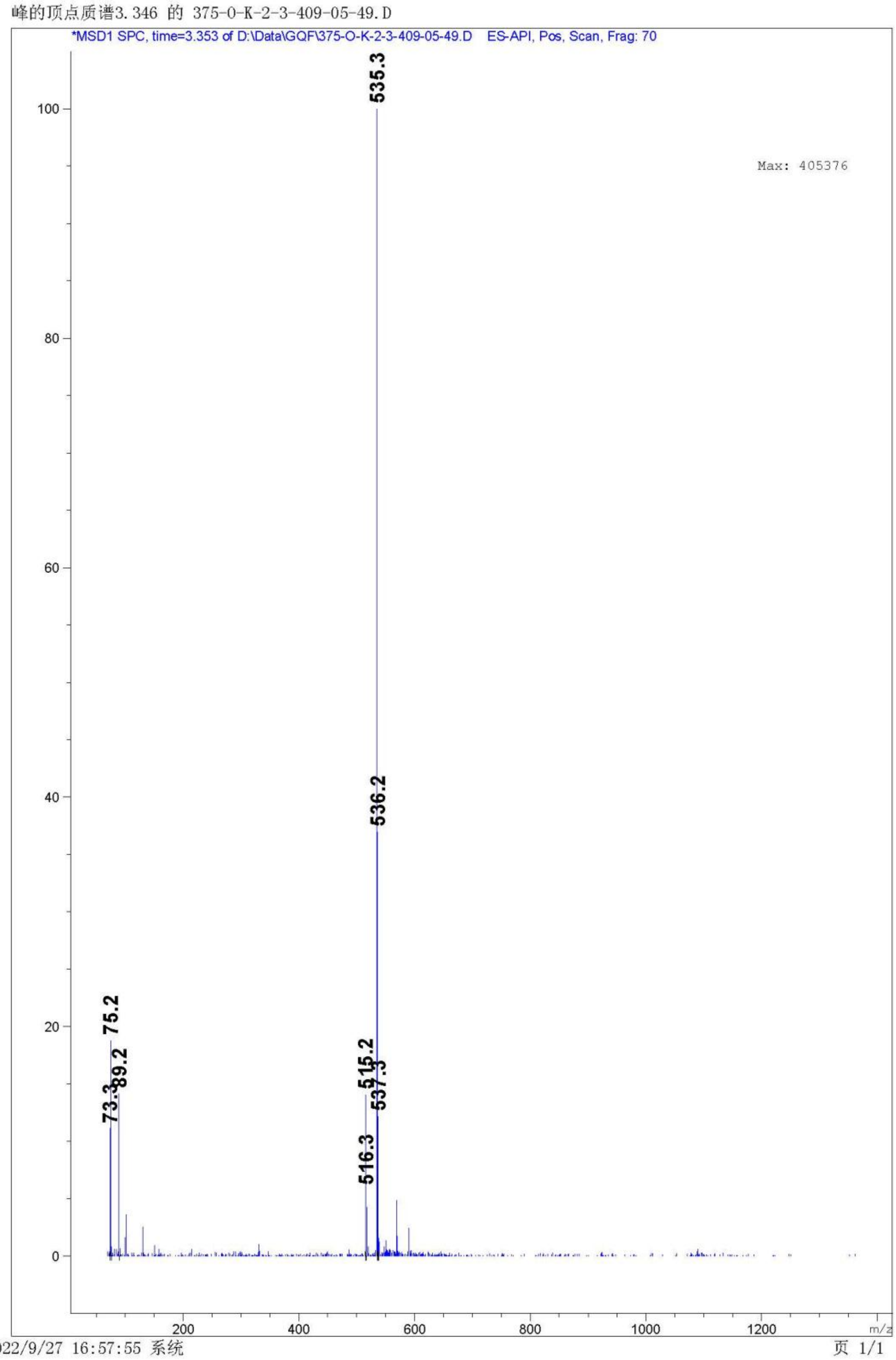

6120 2022/9/27 16:57:55 系统 页 1/1

Figure S54. EIMS spectrum of Compound **6**

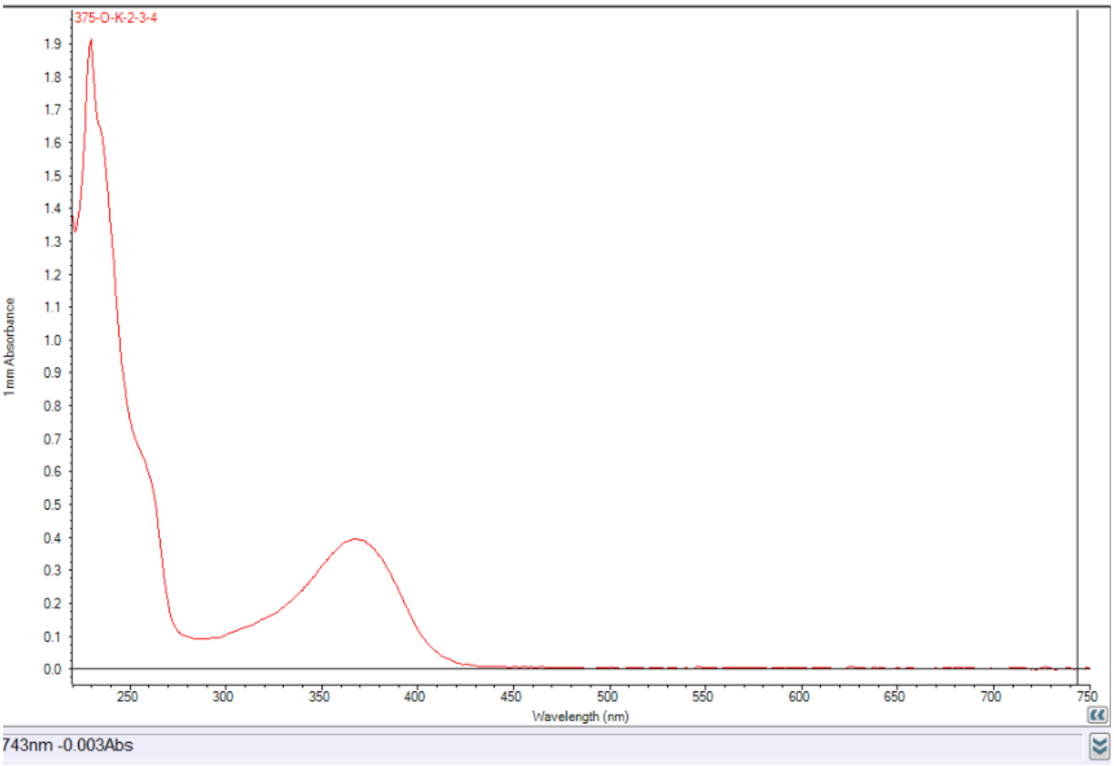

Figure S55. UV spectrum of Compound **6**

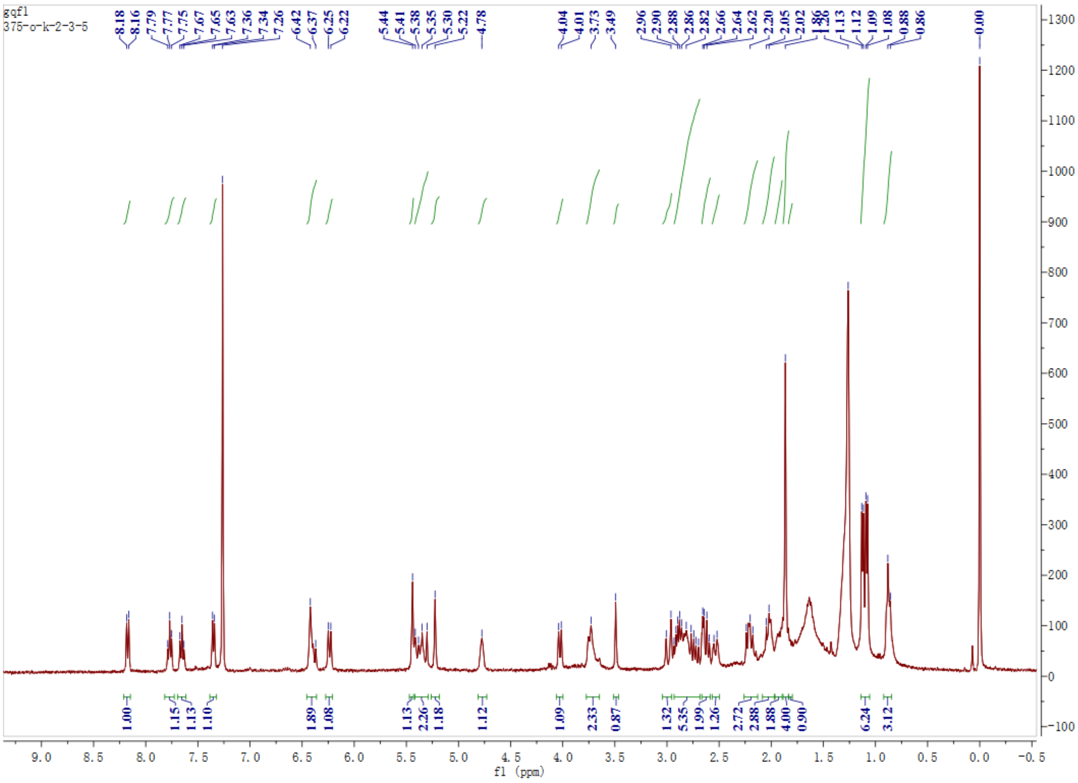

Figure S56. ^{1}H NMR spectrum of compound **7** in $CDCl_3$

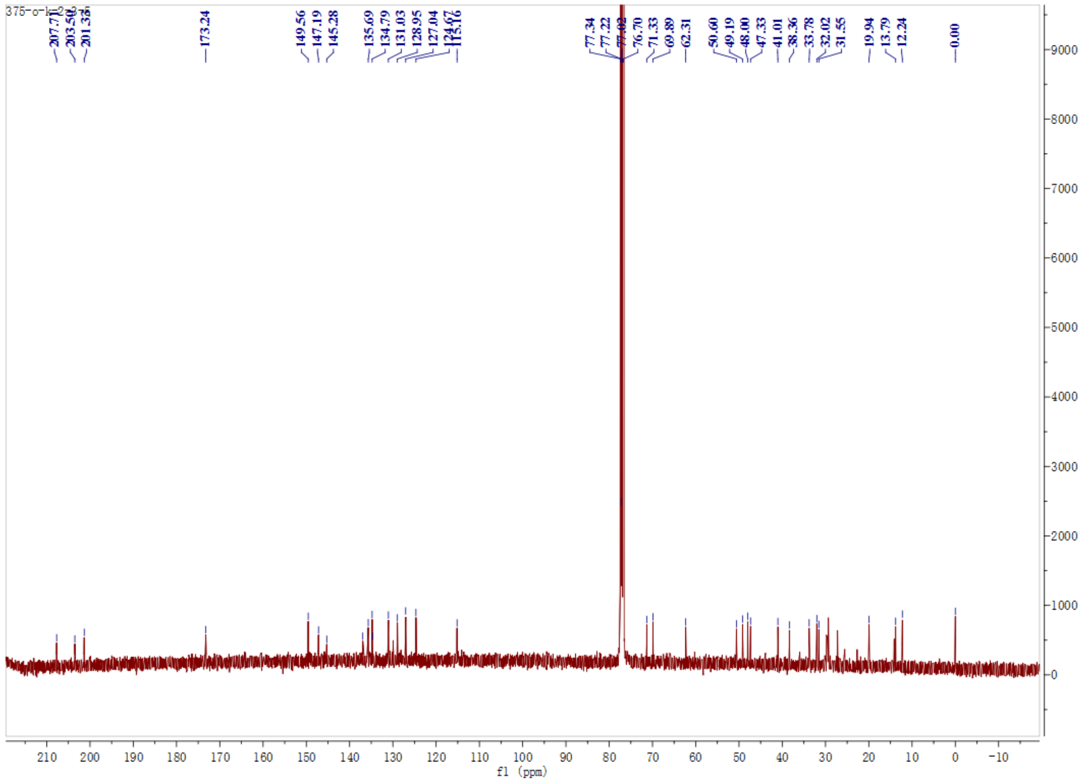

Figure S57. ^{13}C NMR spectrum of compound **7** in $CDCl_3$

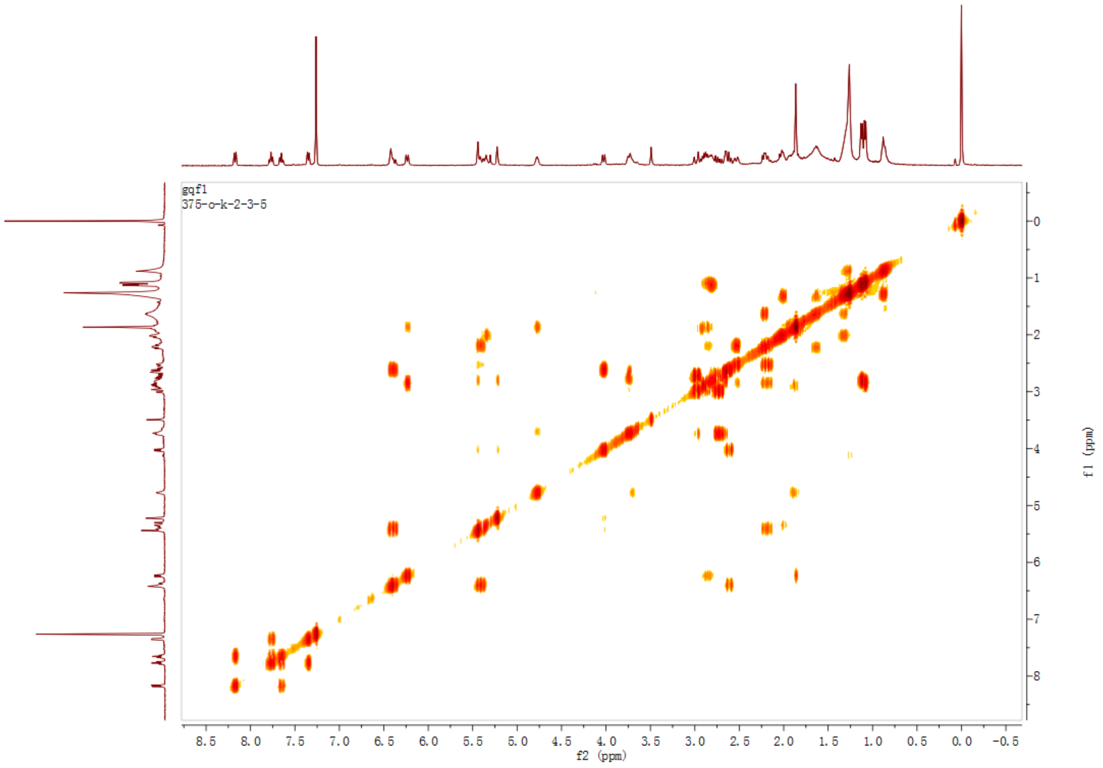

Figure S58. COSY spectrum of compound **7** in $CDCl_3$

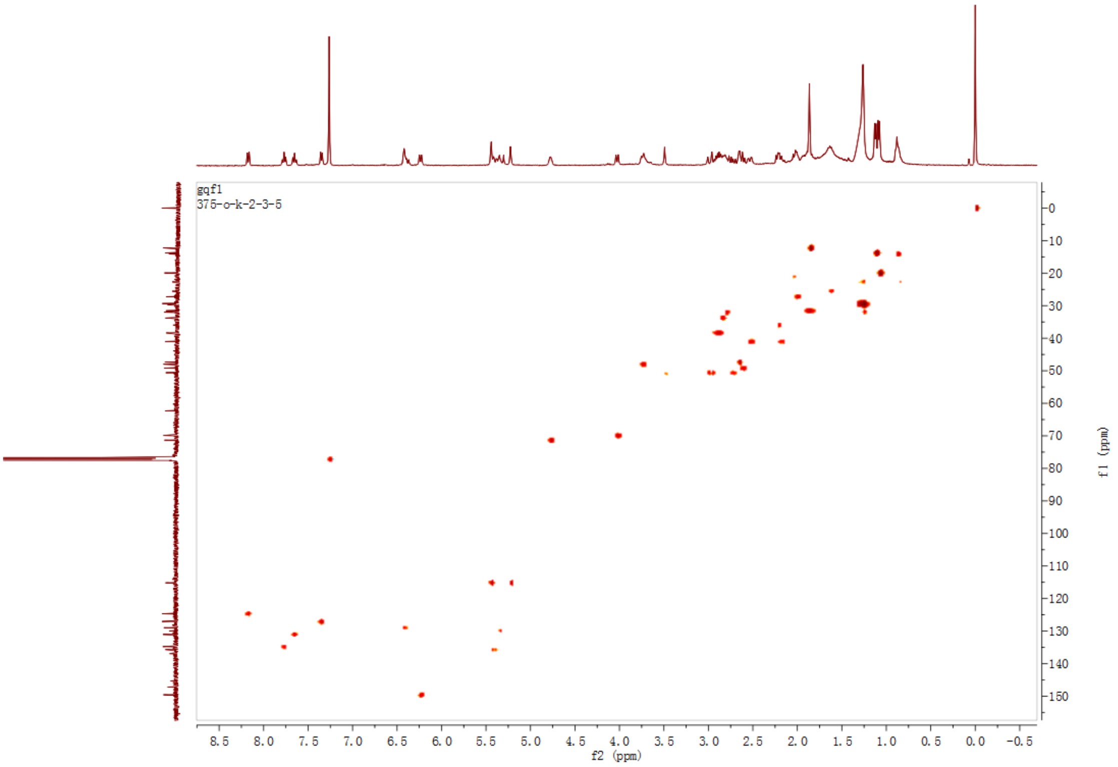

Figure S59. HSQC spectrum of compound **7** in $CDCl_3$

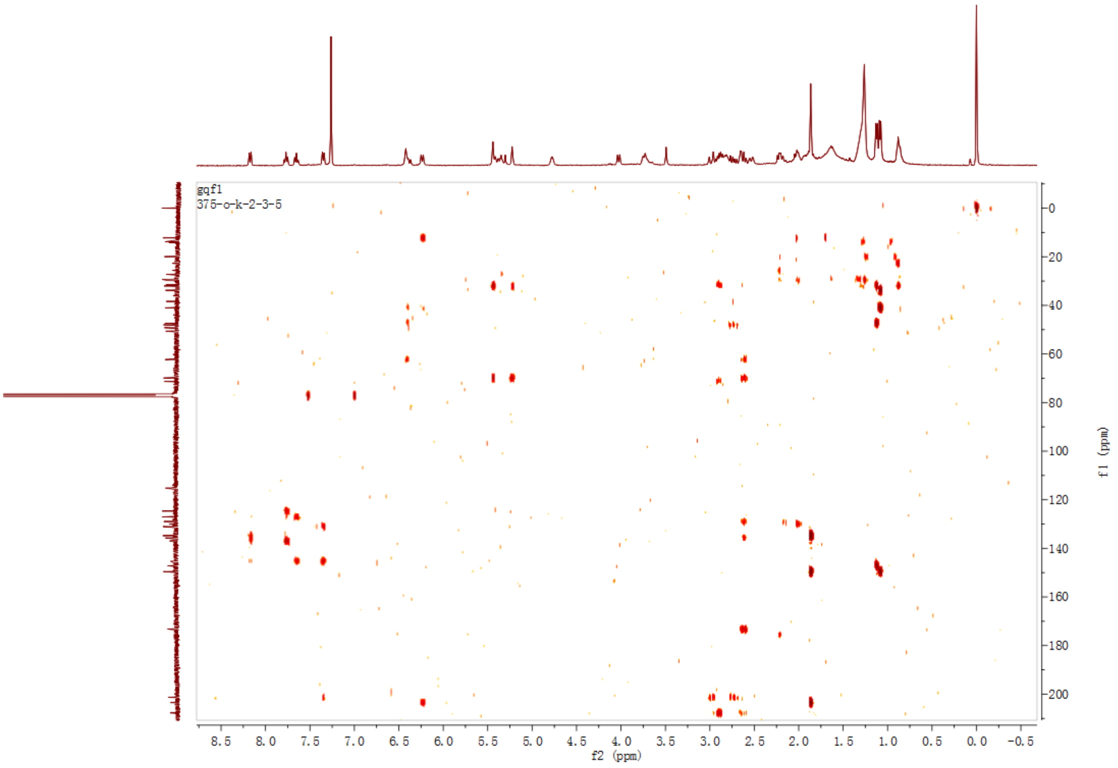

Figure S60. HMBC spectrum of compound **7** in $CDCl_3$

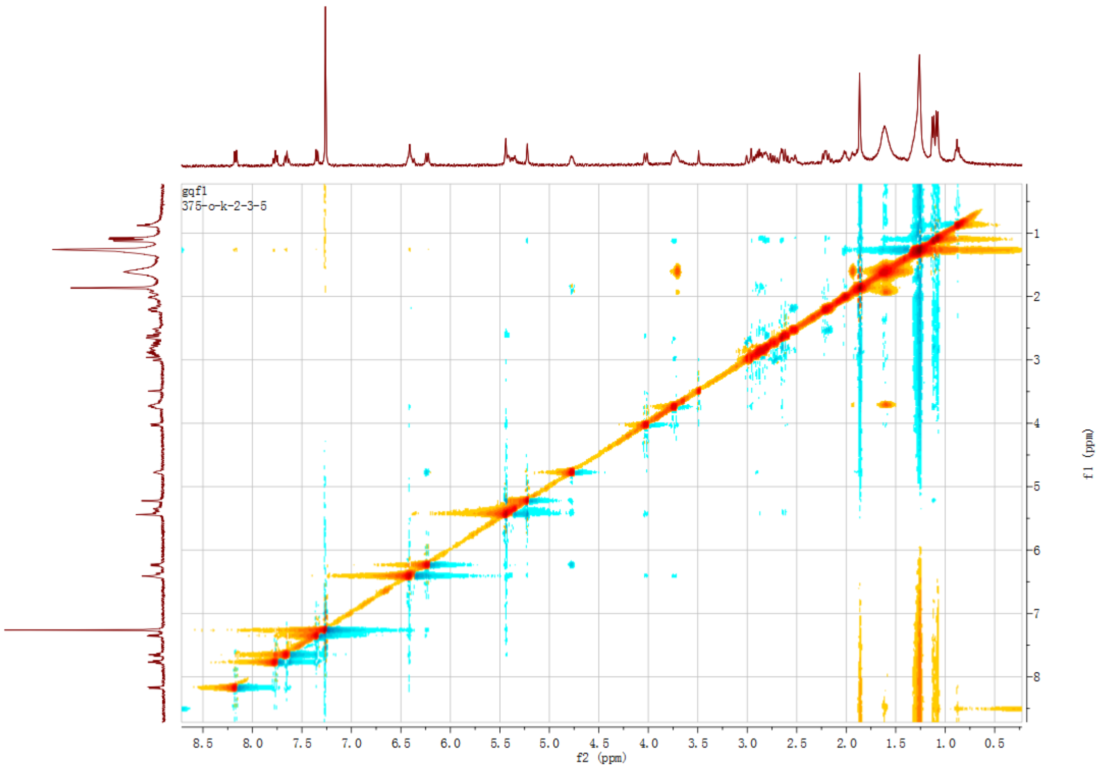

Figure S61. NOESY spectrum of compound **7** in $CDCl_3$

Spectrum Plot Report

Name	Sample2	Rack Pos.		Instrument	Instrument 1	Operator	
Inj. Vol. (ul)	5	Plate Pos.		IRM Status	Some ions missed		
Data File	375-0-K-2-3-5RR.d	Method (Acq)	CL-2022.m	Comment		Acq. Time (Local)	7/29/2022 4:34:44 PM (UTC+08:00)

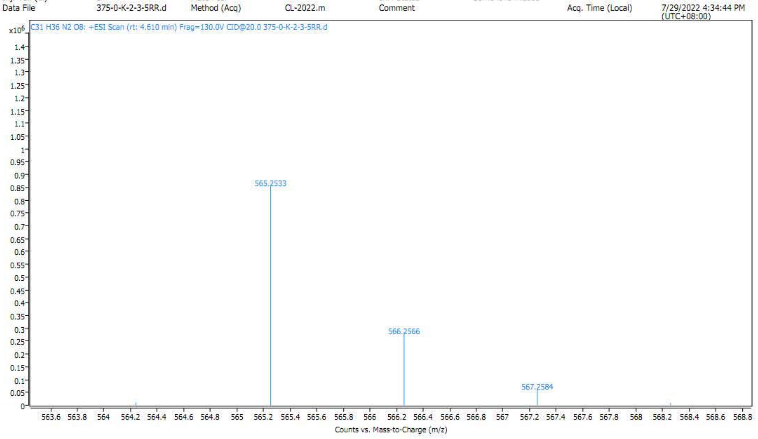

Page 1 of 1 Generated at 2:04 PM on 8/9/2022

Analysis Report

Spectrum Peaks

m/z	Z	Abund	Abund %	m/z (Calc)	Diff (ppm)	Ion Species	Formula	Ion Type
565.2533	1	855903	76.56	565.2544	-1.96	(M+H)+	C31 H36 N2 O8	
566.2566	1	278641	24.92	565.2577	-2.01	(M+H)+	C31 H36 N2 O8	
567.2584	1	60876	5.45	567.2605	-3.69	(M+H)+	C31 H36 N2 O8	
105.0666		12306	1.10					
107.0818		17443	1.56					
109.0615		15691	1.40					
109.0975		18461	1.65					
119.0823		16242	1.45					
120.0409		25356	2.27					
121.0470		13628	1.22					
121.0978		19324	1.73					
123.0765		12975	1.16					
134.0568		20286	1.81					
137.0924		110862	9.92					
147.0772		27475	2.46					
150.0153		44894	4.02					
157.0974		16114	1.44					
165.0879	1	1118011	100.00					
166.0911	1	119004	10.64					
174.0879		15750	1.41					
193.0573		18449	1.65					
198.0883		21573	1.93					
200.1036		63995	5.72					
226.1061		12128	1.08					
282.1097		22887	2.05					
300.1196		23521	2.10					
318.1832		11686	1.05					
336.1926		17180	1.54					

Spectrum Identification Table

Best ID Source	Name	Formula	Species	m/z	Diff (ppm)	CAS	Score	Score (Lib)	Score (DB)	Score (MFG)	Lib/DB
Yes MFG		C31 H36 N2 O8	(M+H)+	565.2533	-2.09		96.70			96.70	
No MFG		C29 H34 N5 O7	(M+H)+	565.2533	0.41		99.54			99.54	
No MFG		C28 H28 N12 O2	(M+H)+	565.2533	0.67		98.13			98.13	
No MFG		C30 H30 N9 O3	(M+H)+	565.2533	-1.84		96.03			96.03	
No MFG		C28 H38 N O11	(M+H)+	565.2533	2.65		95.86			95.36	

MassHunter Qualitative Analysis Page 2 of 3 Generated at 2:03 PM on 8/9/2022

Figure S62. HRESIMS spectrum of compound **7**

打印窗口 80: 峰的顶点质谱3.361 的 375-0-K-2-3-509-05-59.D

峰的顶点质谱3.361 的 375-0-K-2-3-509-05-59.D

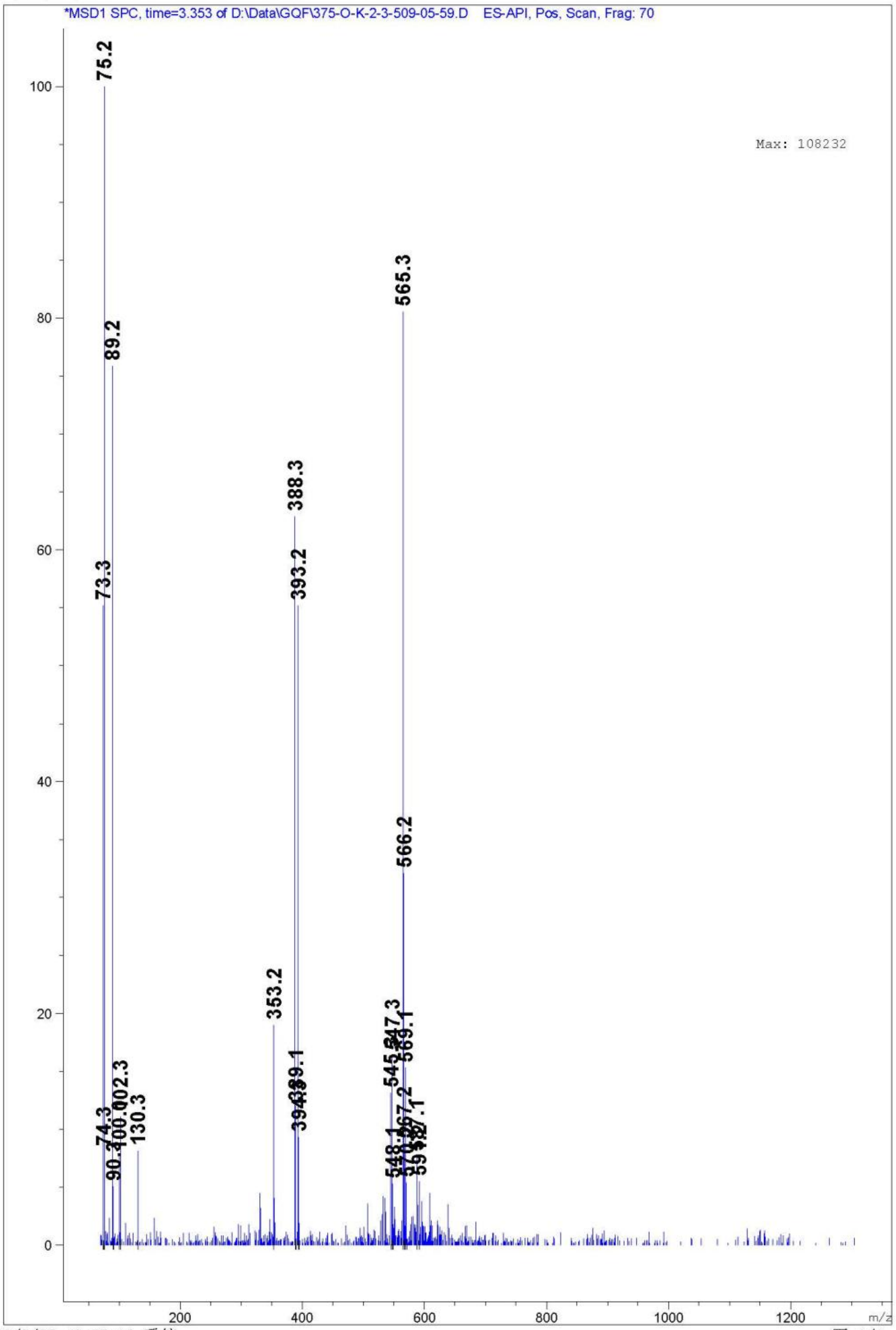

6120 2022/9/27 16:55:16 系统 页 1/1

Figure S63. EIMS spectrum of Compound **7**

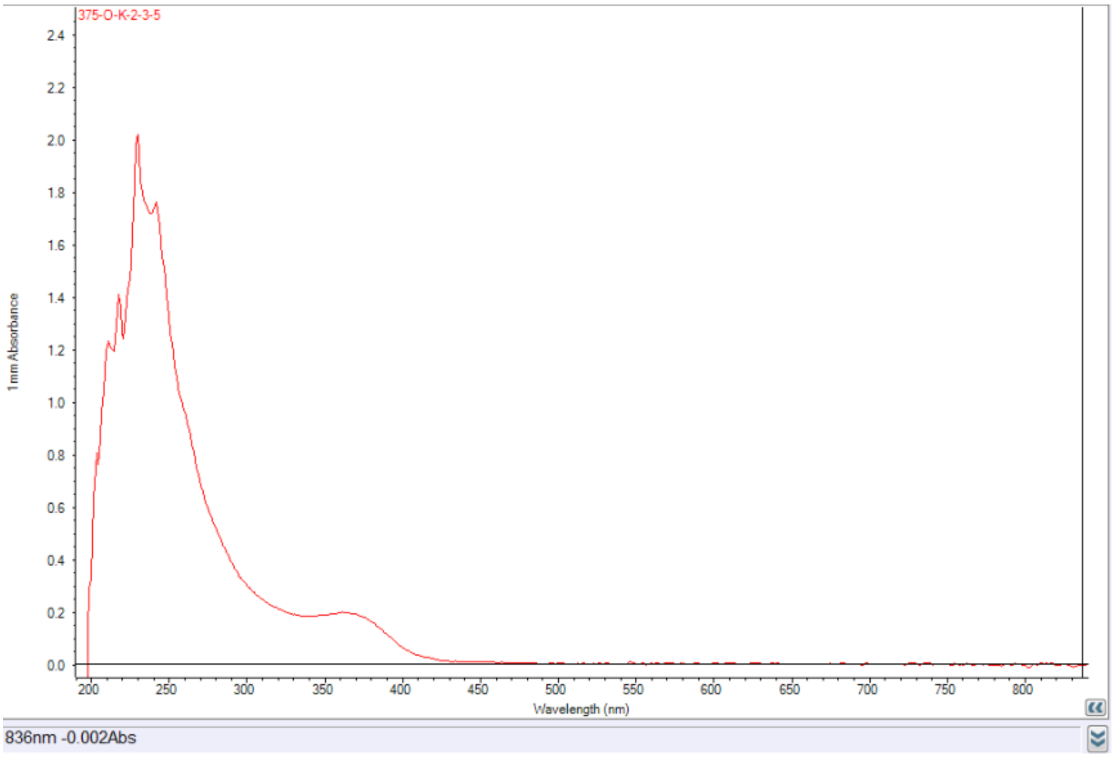

Figure S64. UV spectrum of Compound **7**

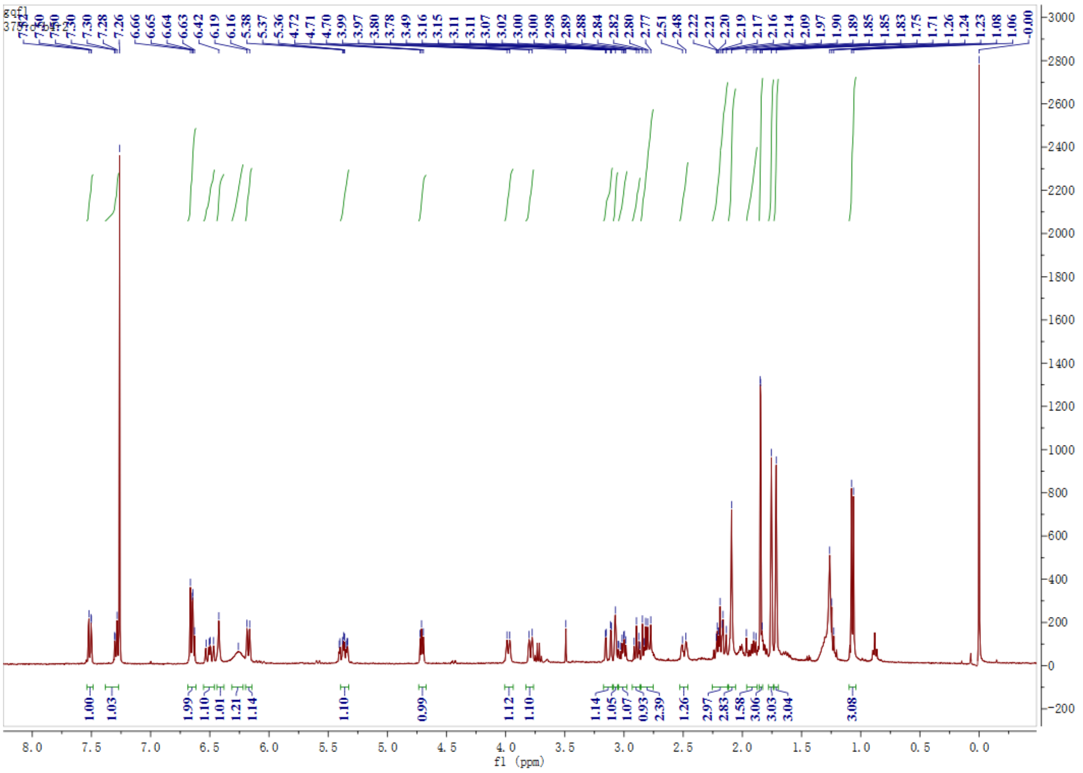

Figure S65. ^{1}H NMR spectrum of compound **8** in $CDCl_3$

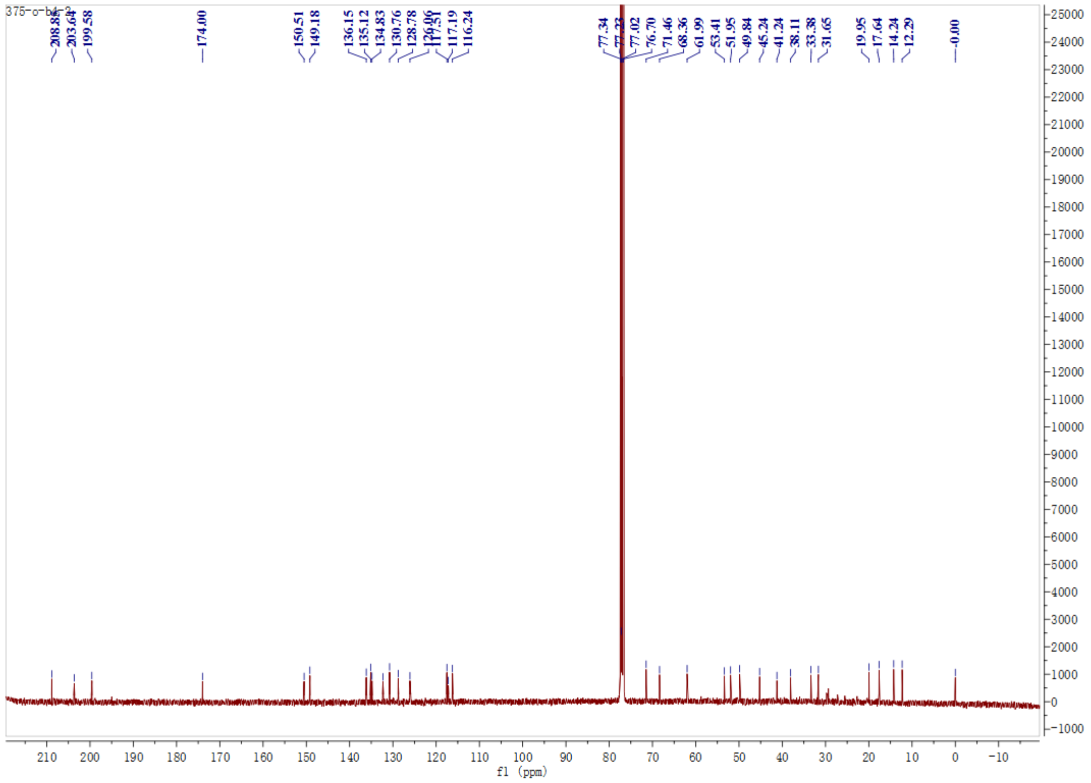

Figure S66. ^{13}C NMR spectrum of compound **8** in $CDCl_3$

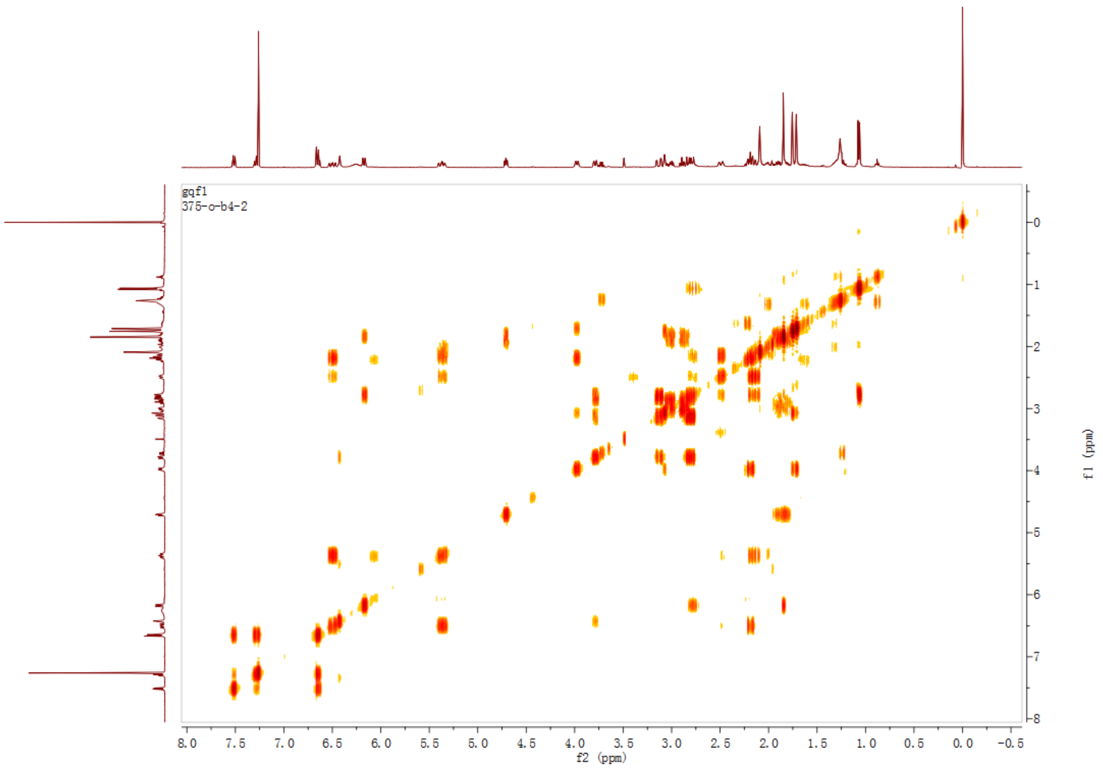

Figure S67. COSY spectrum of compound **8** in $CDCl_3$

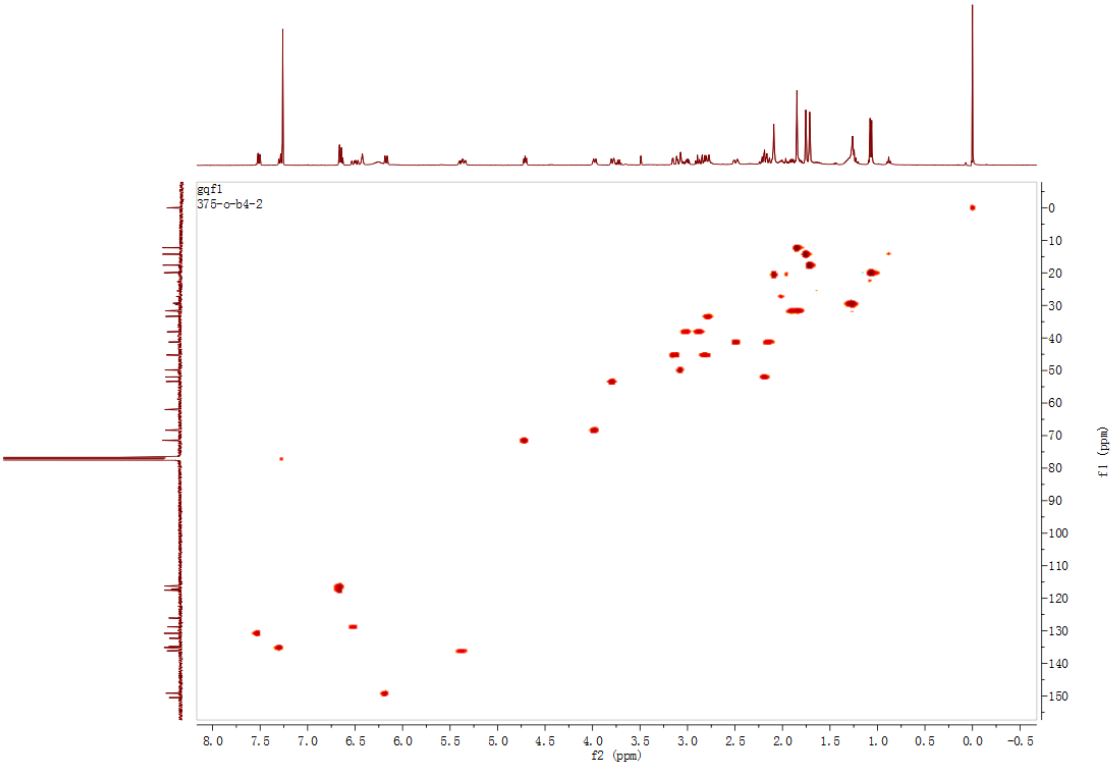

Figure S68. HSQC spectrum of compound **8** in $CDCl_3$

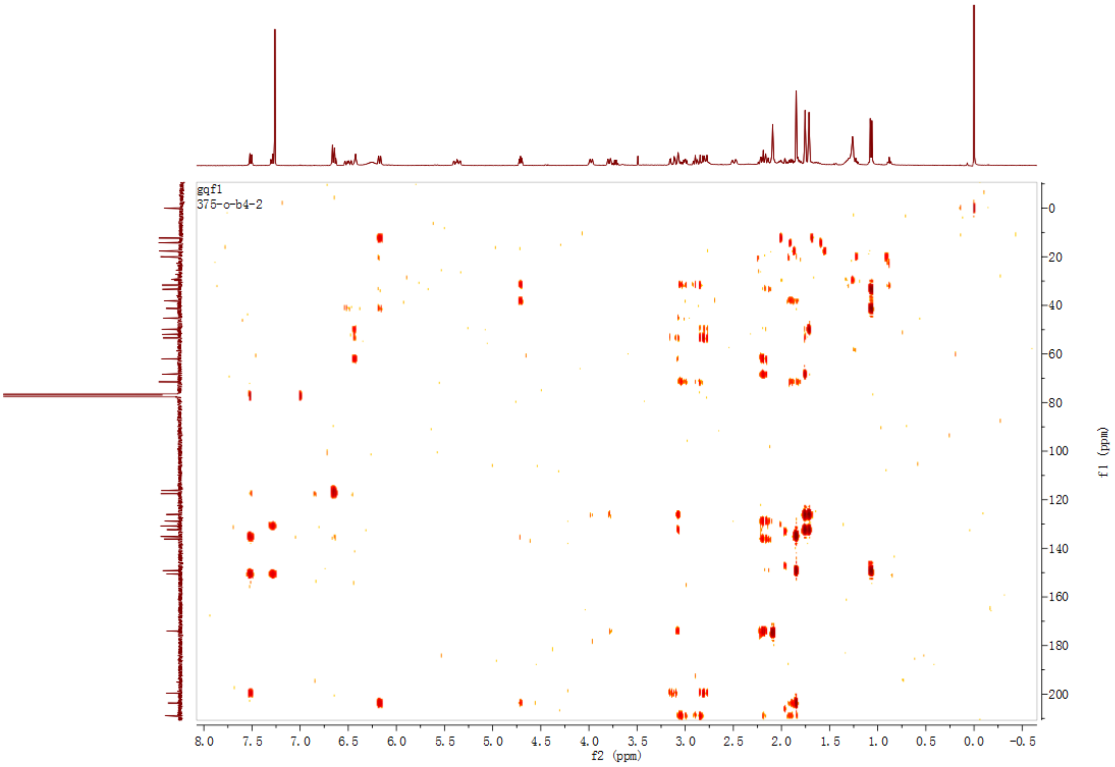

Figure S69. HMBC spectrum of compound **8** in $CDCl_3$

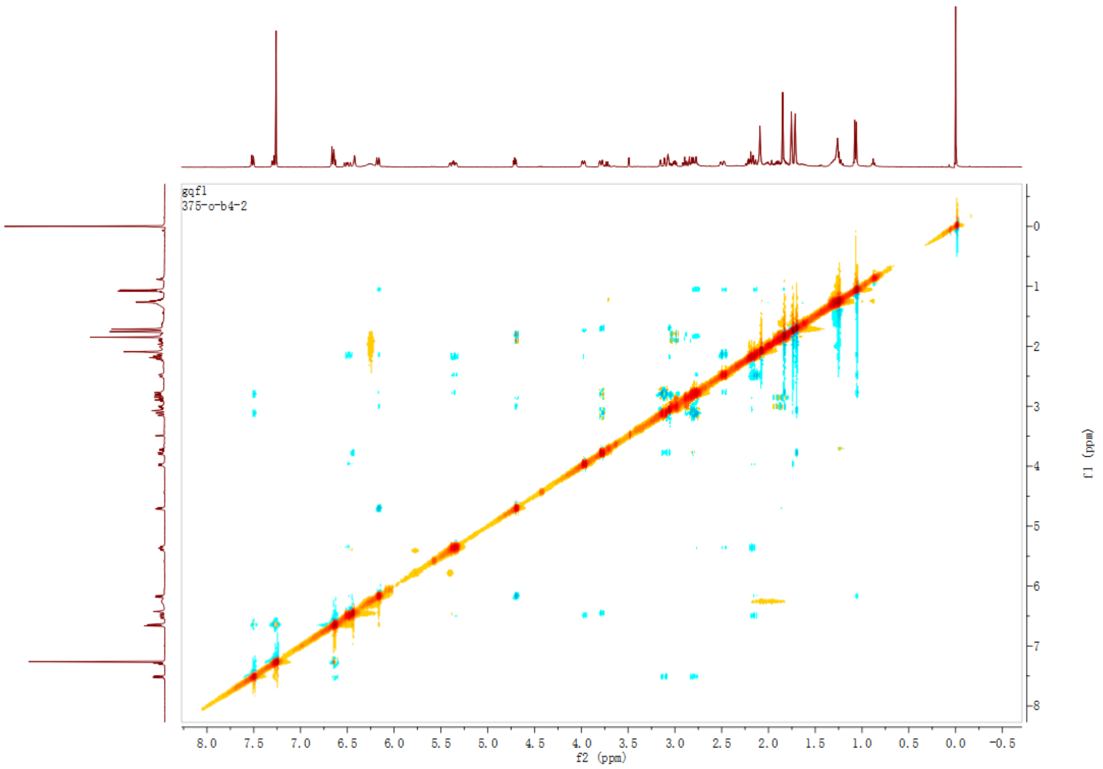

Figure S70. NOESY spectrum of compound **8** in $CDCl_3$

Spectrum Plot Report

Name	Sample5	Rack Pos.		Instrument	Instrument 1	Operator	
Inj. Vol. (ul)	5	Plate Pos.		IRM Status	Success		
Data File	375-0-B4-2R.d	Method (Acq)	CL-2022.m	Comment		Acq. Time (Local)	7/29/2022 3:53:28 PM (UTC+08:00)

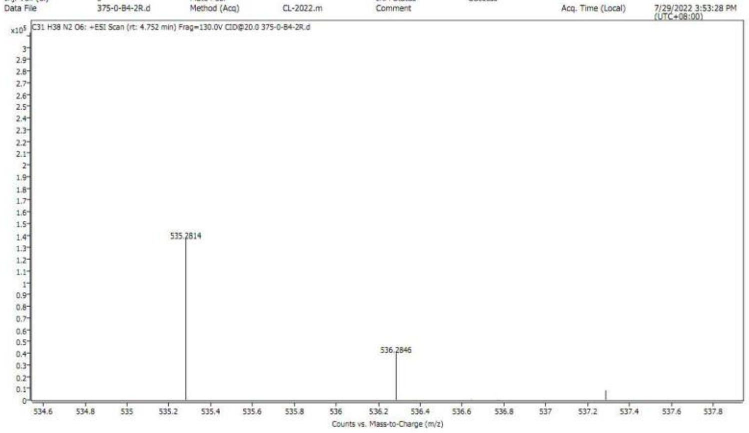

Page 1 of 1 Generated at 10:59 AM on 8/9/2022

Analysis Report

Spectrum Peaks

m/z	Z	Abund	Abund %	m/z (Calc)	Diff (ppm)	Ion Species	Formula	Ion Type
535.2814	1	137535	16.92	535.2803	2.16	(M+H)+	C31 H38 N2 O6	
536.2846	1	40063	4.93	536.2835	1.92	(M+H)+	C31 H38 N2 O6	
109.0665		27396	3.37					
109.1028		12162	1.50					
119.0874		13558	1.67					
120.0461	1	344362	42.37					
121.0509	1	30630	3.77					
136.0776		62242	7.66					
137.0980		68838	8.47					
145.1023		16399	2.02					
146.0618	1	328365	40.40					
147.0655	1	30152	3.71					
147.0804		18117	2.23					
157.1033		48667	5.99					

Spectrum Identification Table

Best	ID Source	Name	Formula	Species	m/z	Diff (ppm)	CAS	Score	Score (Lib)	Score (DB)	Score (MFG)	Lib/DB
Yes	MFG		C31 H38 N2 O6	(M+H)+	535.2814	2.07		93.31			93.31	
No	MFG		C17 H30 N18 O3	(M+H)+	535.2814	-0.51		95.35			95.35	
No	MFG		C18 H36 N11 O8	(M+H)+	535.2814	-0.86		95.19			95.19	
No	MFG		C19 H42 N4 O13	(M+H)+	535.2814	-1.20		93.31			93.31	
No	MFG		C19 H32 N15 O4	(M+H)+	535.2814	-3.22		92.23			92.23	

MassHunter Qual 10.0
(End of Report)

Figure S71. HRESIMS spectrum of compound **8**

打印窗口 80: MS Spectrum

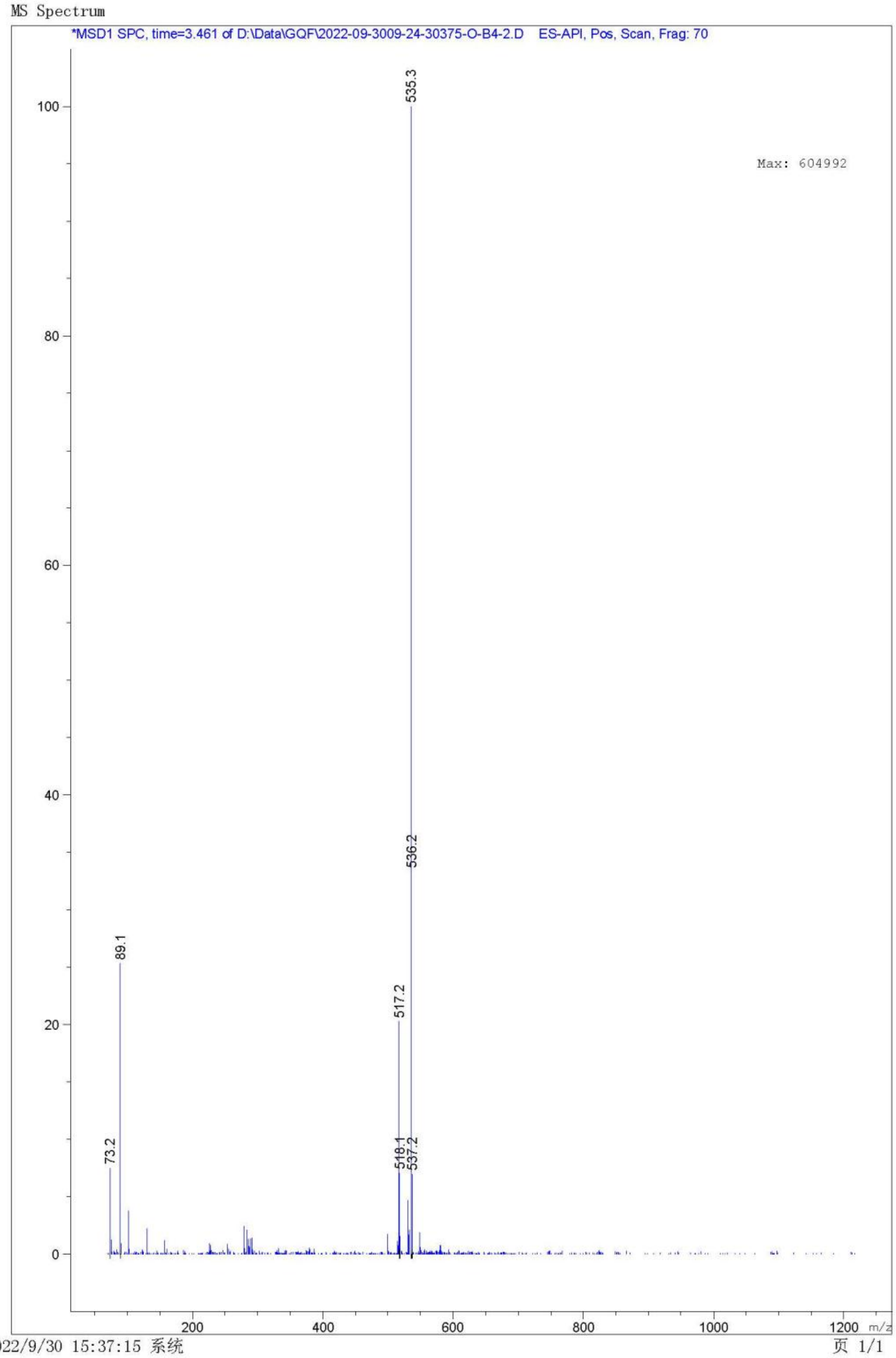

6120 2022/9/30 15:37:15 系统 页 1/1

Figure S72. EIMS spectrum of Compound **8**

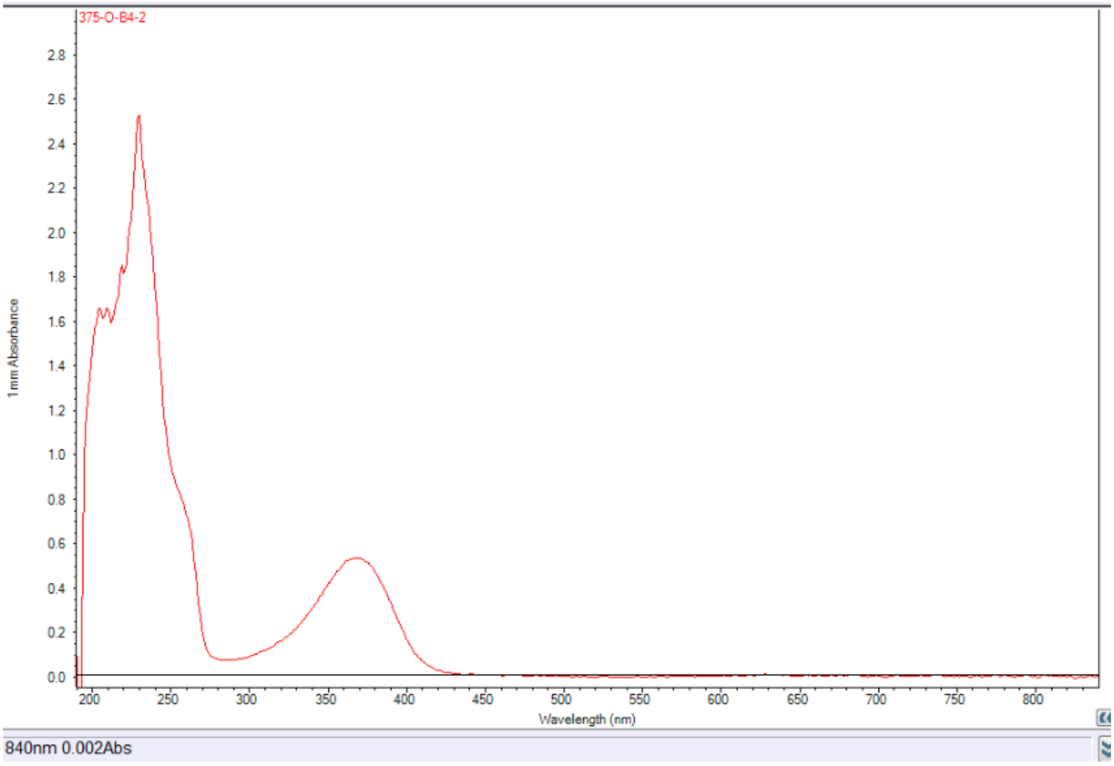

Figure S73. UV spectrum of Compound **8**

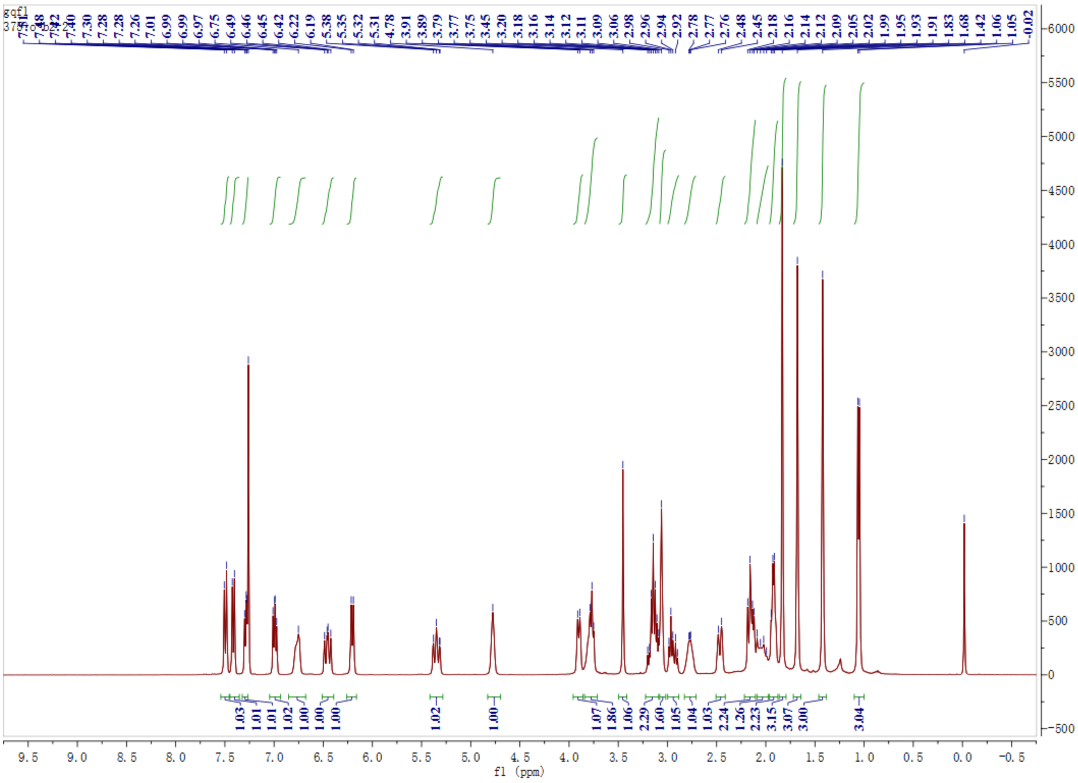

Figure S74. ^{1}H NMR spectrum of compound **9** in $CDCl_3$

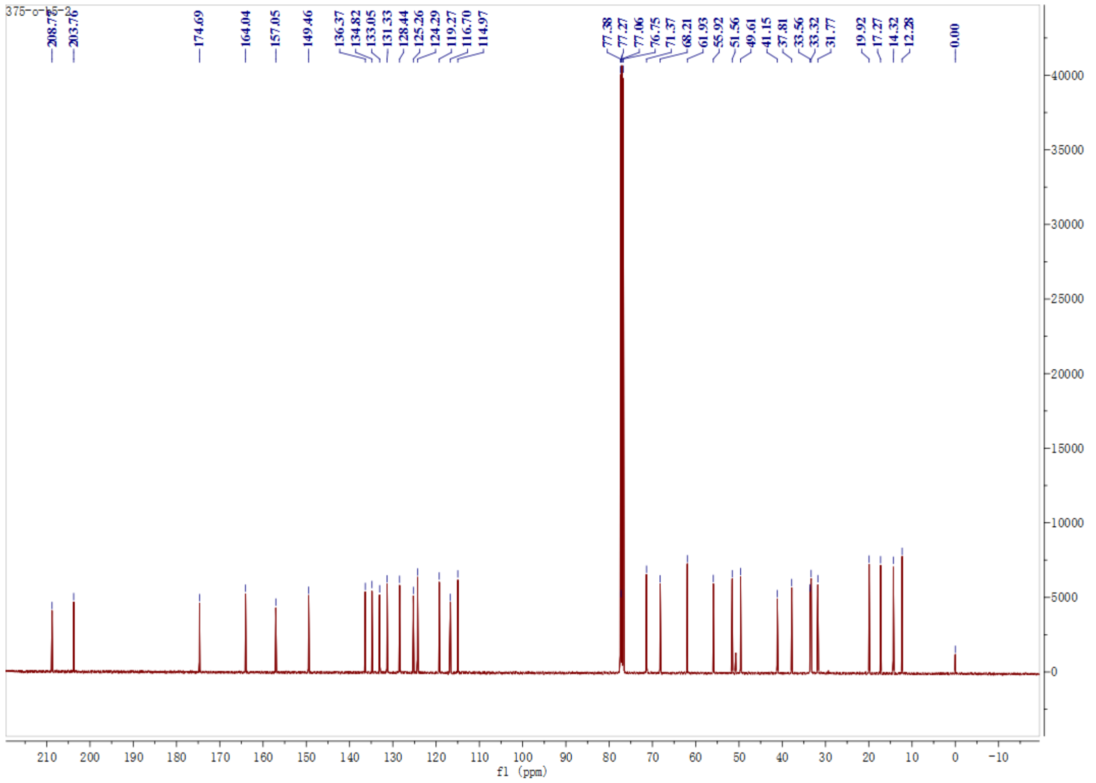

Figure S75. ^{13}C NMR spectrum of compound **9** in $CDCl_3$

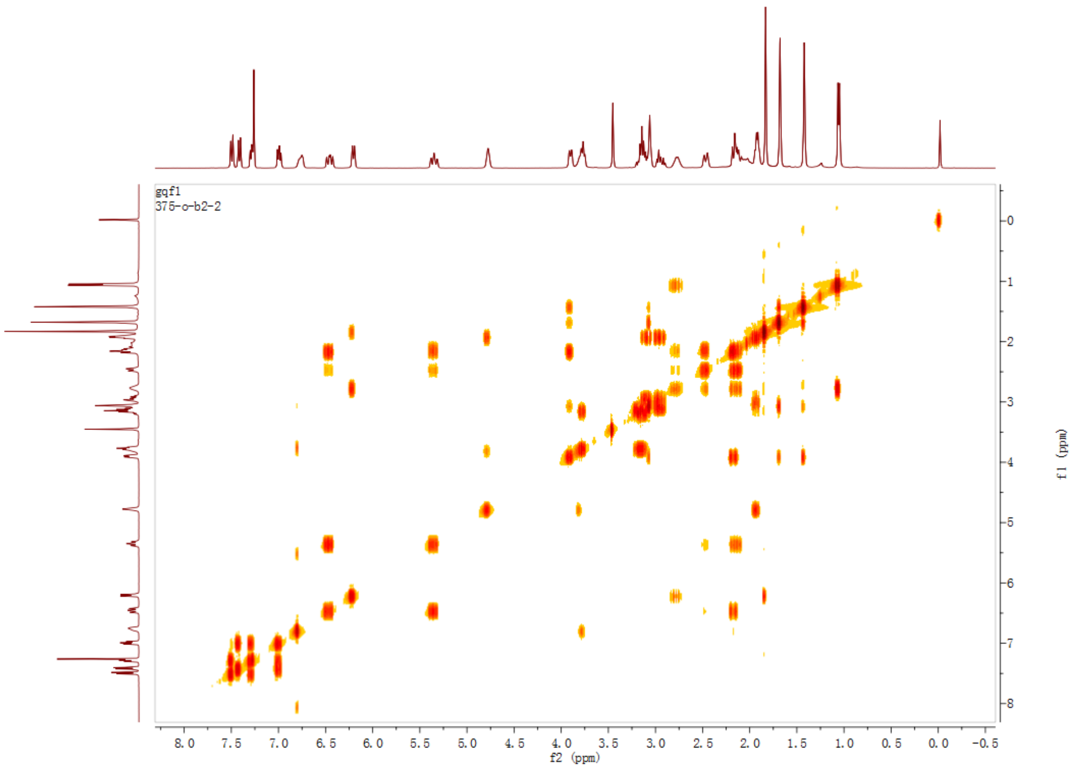

Figure S76. COSY spectrum of compound **9** in $CDCl_3$

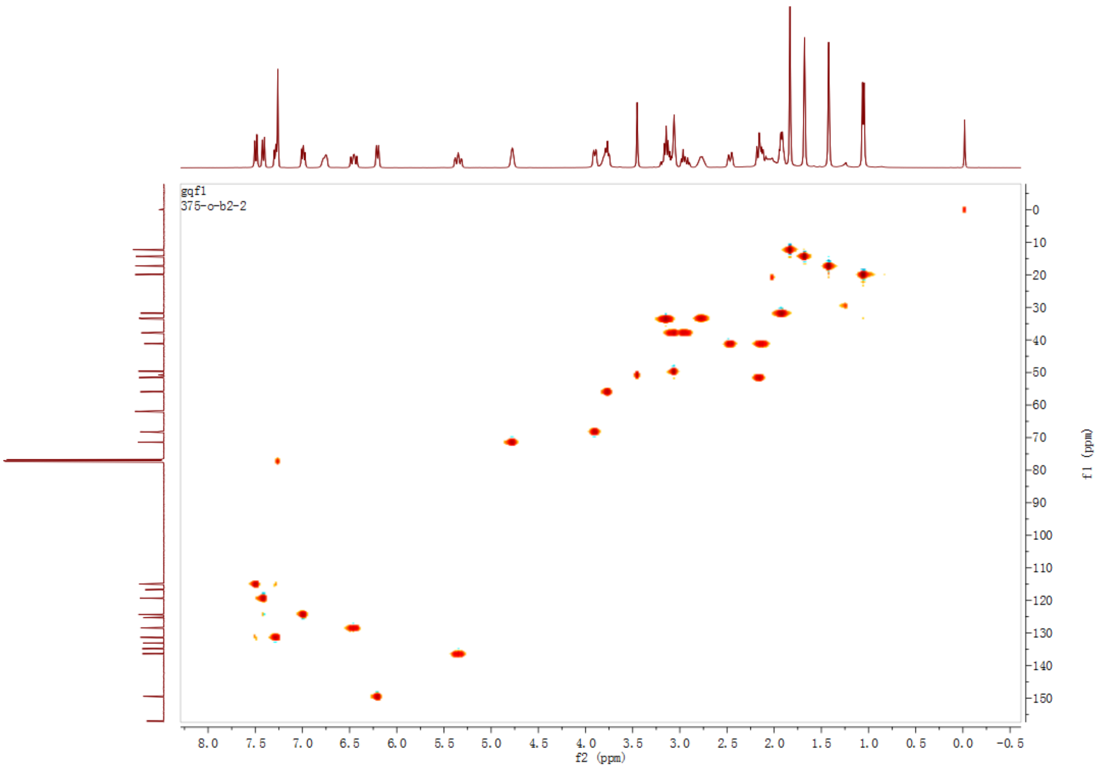

Figure S77. HSQC spectrum of compound **9** in $CDCl_3$

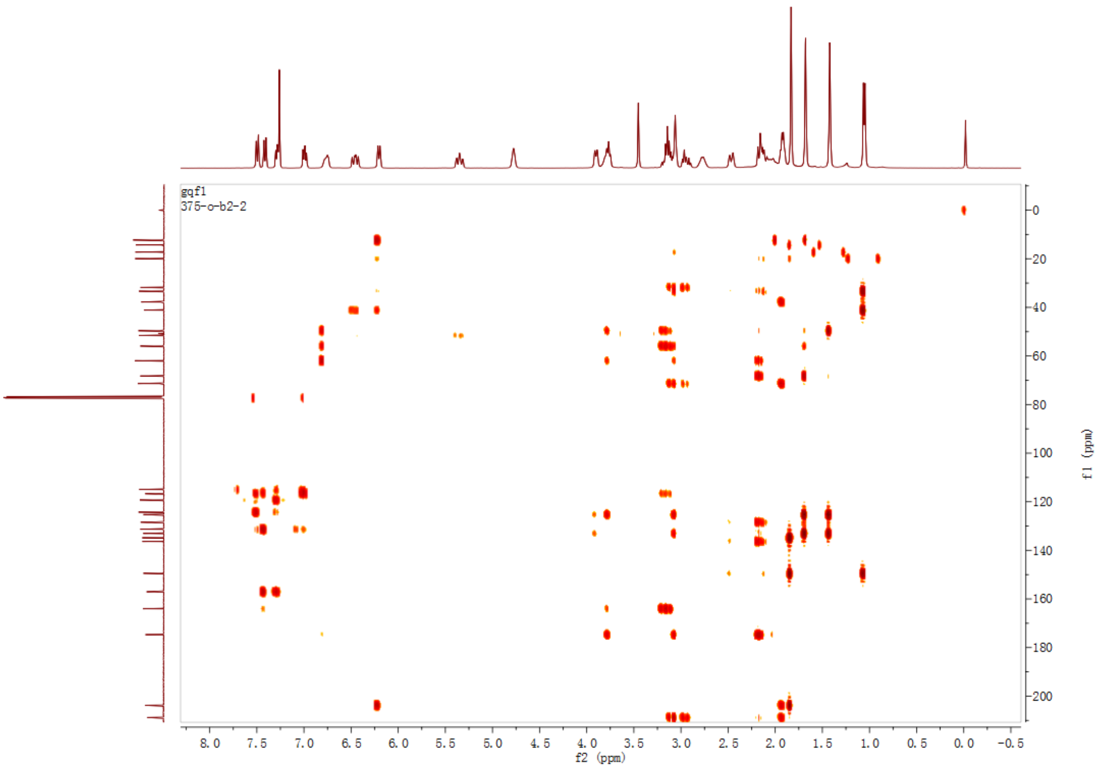

Figure S78. HMBC spectrum of compound **9** in $CDCl_3$

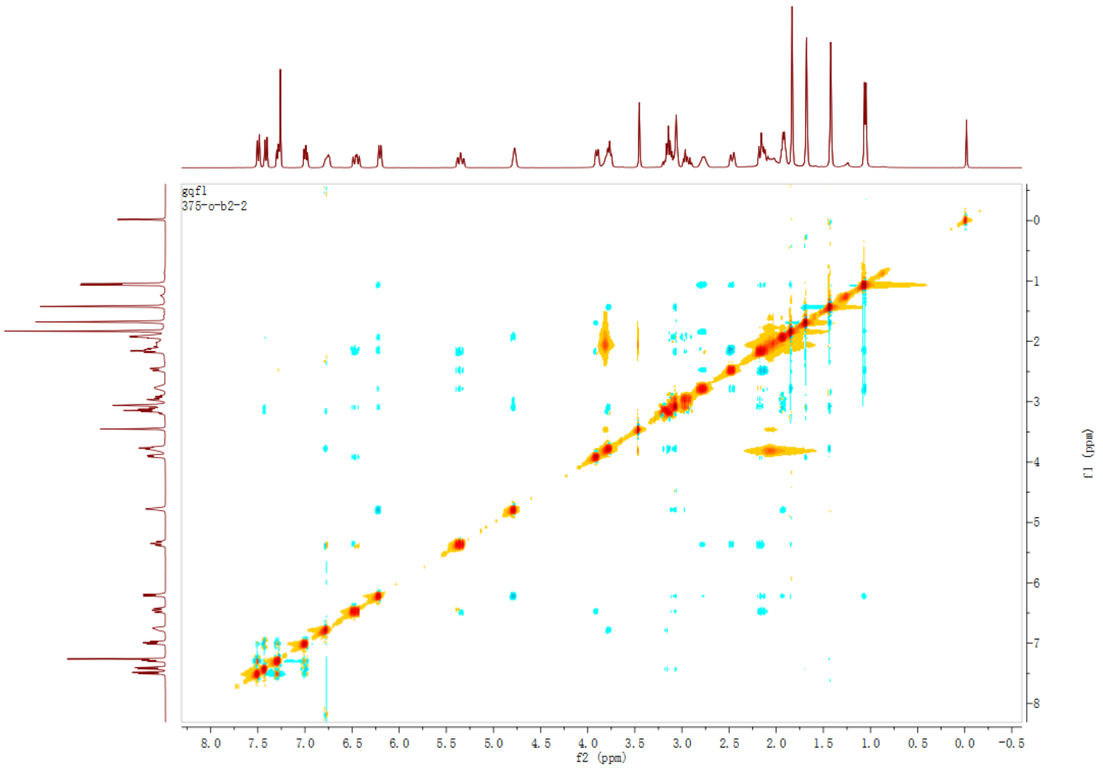

Figure S79. NOESY spectrum of compound **9** in $CDCl_3$

Spectrum Plot Report

Name	Sample4	Rack Pos.		Instrument	Instrument 1	Operator	
Inj. Vol. (ul)	5	Plate Pos.		IRM Status	Success		
Data File	375-0-B5-2R.d	Method (Acq)	CL-2022.m	Comment		Acq. Time (Local)	7/29/2022 3:39:44 PM (UTC+08:00)

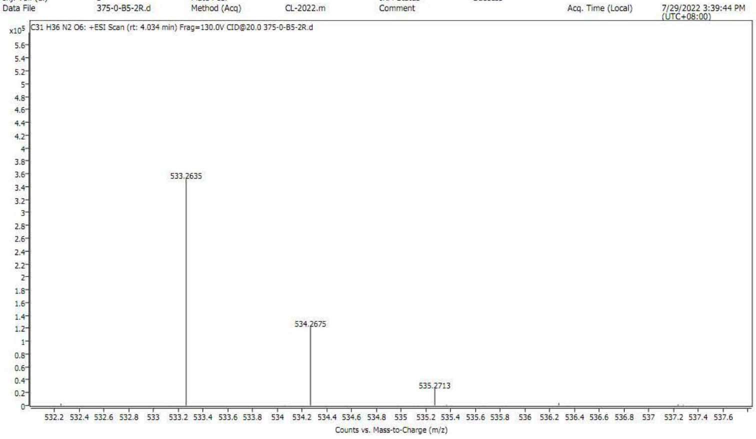

Page 1 of 1 Generated at 10:33 AM on 8/9/2022

Analysis Report

Spectrum Peaks

m/z	Z	Abund	Abund %	m/z (Calc)	Diff (ppm)	Ion Species	Formula	Ion Type
533.2635	1	351924	72.68	533.2646	-2.07	(M+H)+	C31 H36 N2 O6	
534.2675	1	122420	25.28	534.2679	-0.75	(M+H)+	C31 H36 N2 O6	
535.2713	1	26855	5.55	535.2707	1.00	(M+H)+	C31 H36 N2 O6	
109.0620		20417	4.22					
109.0982		15711	3.24					
119.0826		11256	2.32					
120.0416		17823	3.68					
121.0480		16187	3.34					
121.0596		11789	2.43					
121.0982		11693	2.41					
123.0772		14873	3.07					
134.0572	1	251319	51.90					
135.0605	1	19640	4.06					
135.0751		14936	3.08					
137.0933		57166	11.81					
142.0744		10319	2.13					
146.0570		10561	2.18					
147.0776		10017	2.07					
157.0984		35773	7.39					

Spectrum Identification Table

Best	ID Source	Name	Formula	Species	m/z	Diff (ppm)	CAS	Score	Score (Lib)	Score (DB)	Score (MFG)	Lib/DB
Yes	MFG		C31 H36 N2 O6	(M+H)+	533.2635	-1.58		97.69			97.69	
No	MFG		C29 H34 N5 O5	(M+H)+	533.2635	1.08		97.69			97.69	
No	MFG		C30 H30 N9 O	(M+H)+	533.2635	-1.30		96.41			96.41	
No	MFG		C28 H28 N12	(M+H)+	533.2635	1.37		95.54			95.54	
No	MFG		C28 H38 N O9	(M+H)+	533.2635	3.44		91.55			91.55	

MassHunter Qual 10.0
(End of Report)

Figure S80. HRESIMS spectrum of compound **9**

打印窗口 80: 峰的顶点质谱3.411 的 2022-09-2917-27-49375-0-B5-2.D

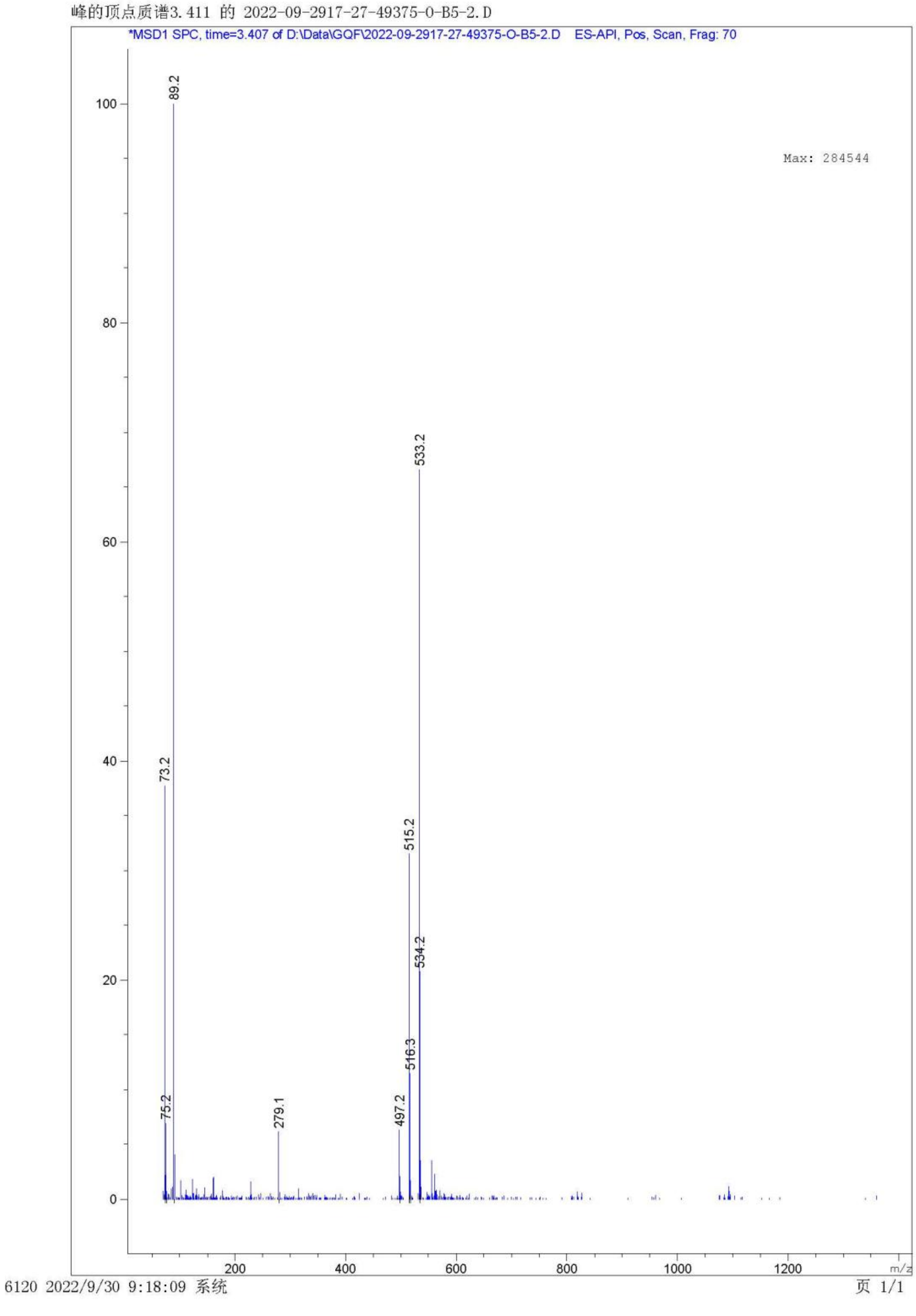

Figure S81. EIMS spectrum of Compound **9**

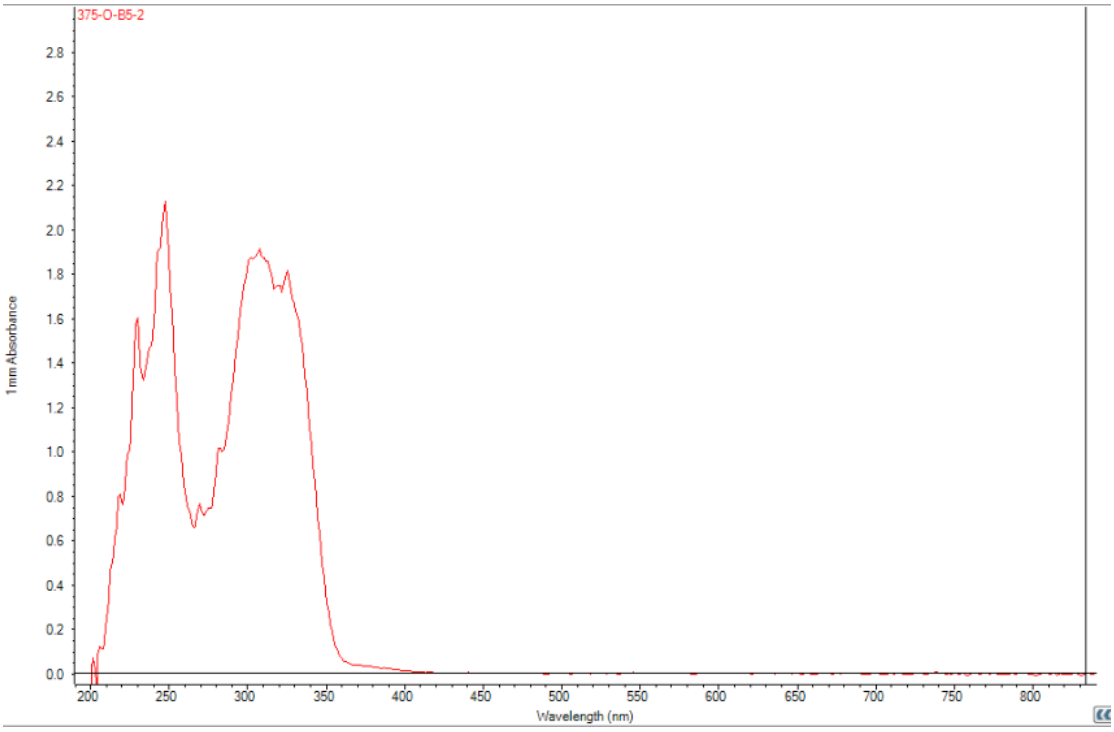

Figure S82. UV spectrum of Compound **9**

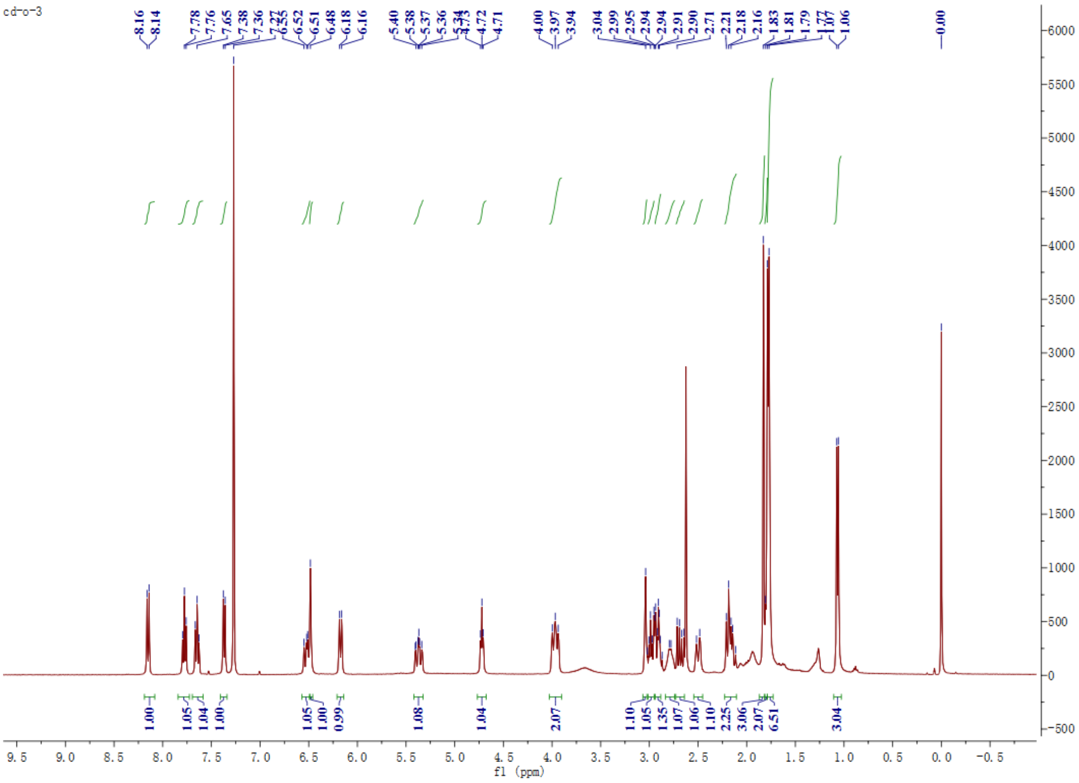

Figure S83. ¹H NMR spectrum of compound **10** in $CDCl_3$

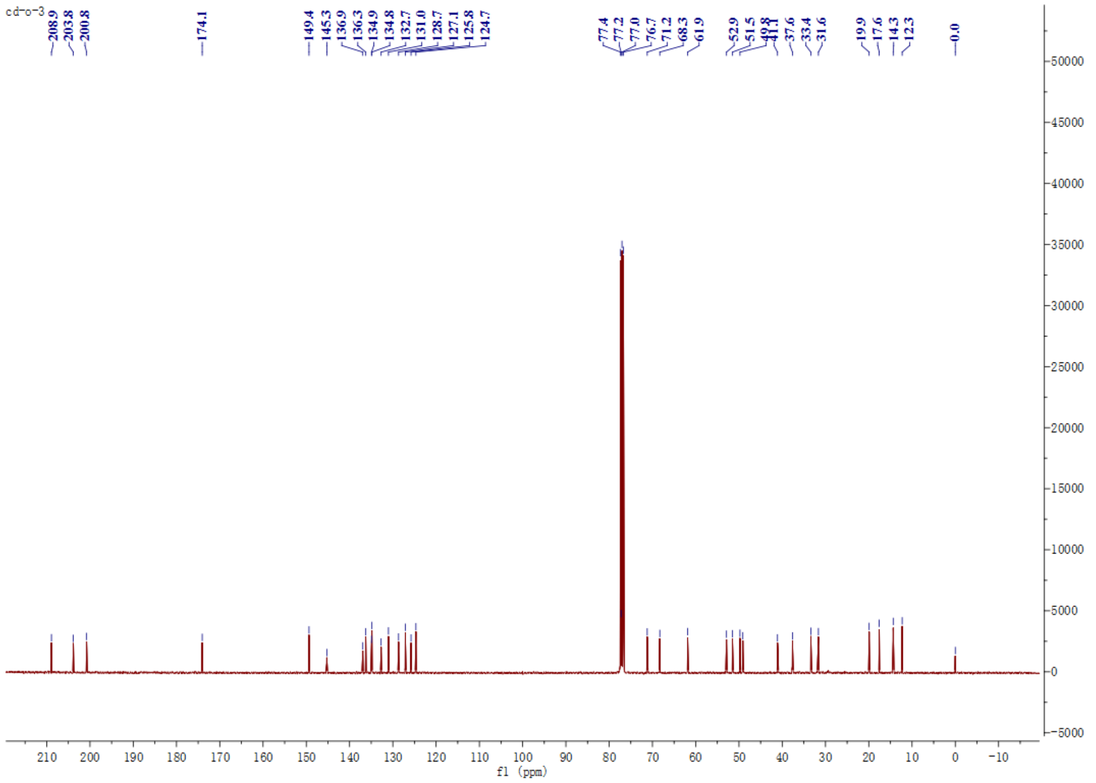

Figure S84. ^{13}C NMR spectrum of compound **10** in $CDCl_3$

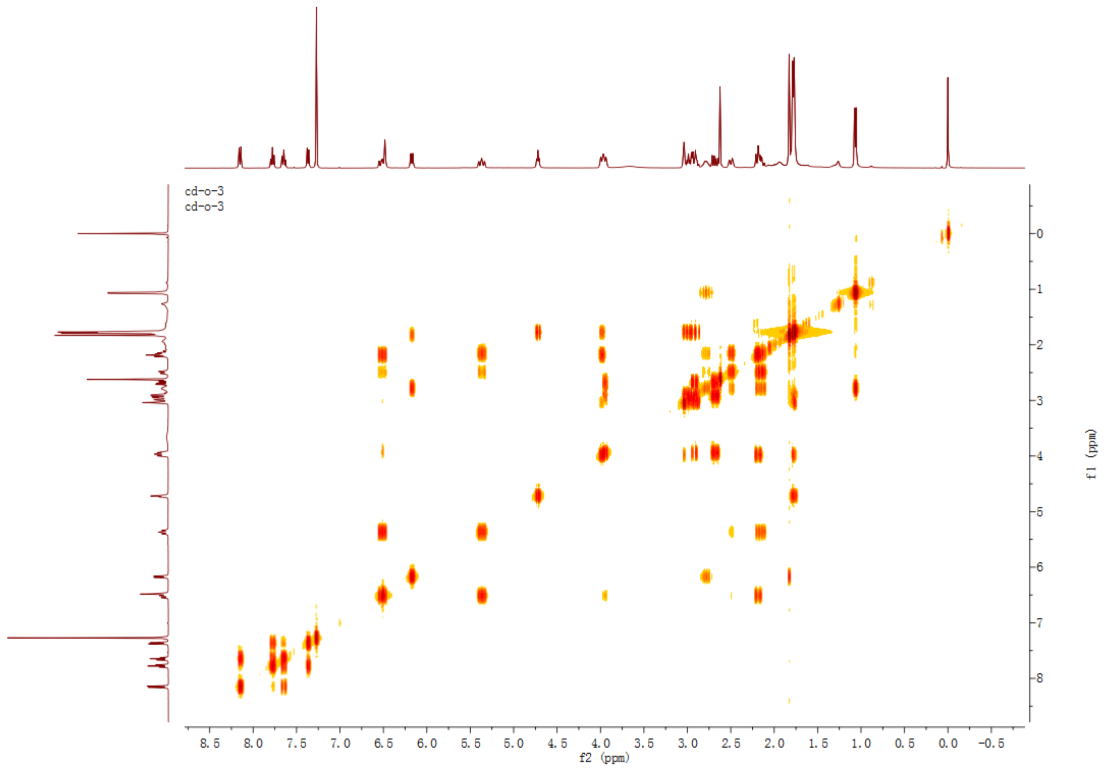

Figure S85. COSY spectrum of compound **10** in $CDCl_3$

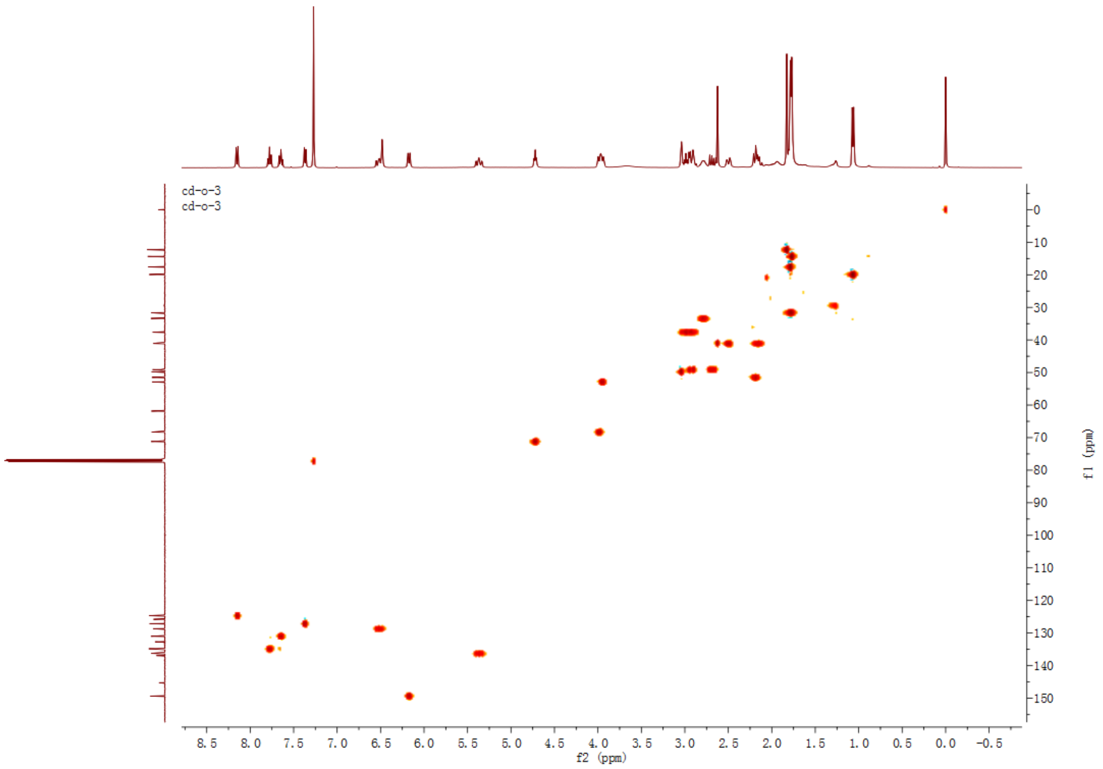

Figure S86. HSQC spectrum of compound **10** in $CDCl_3$

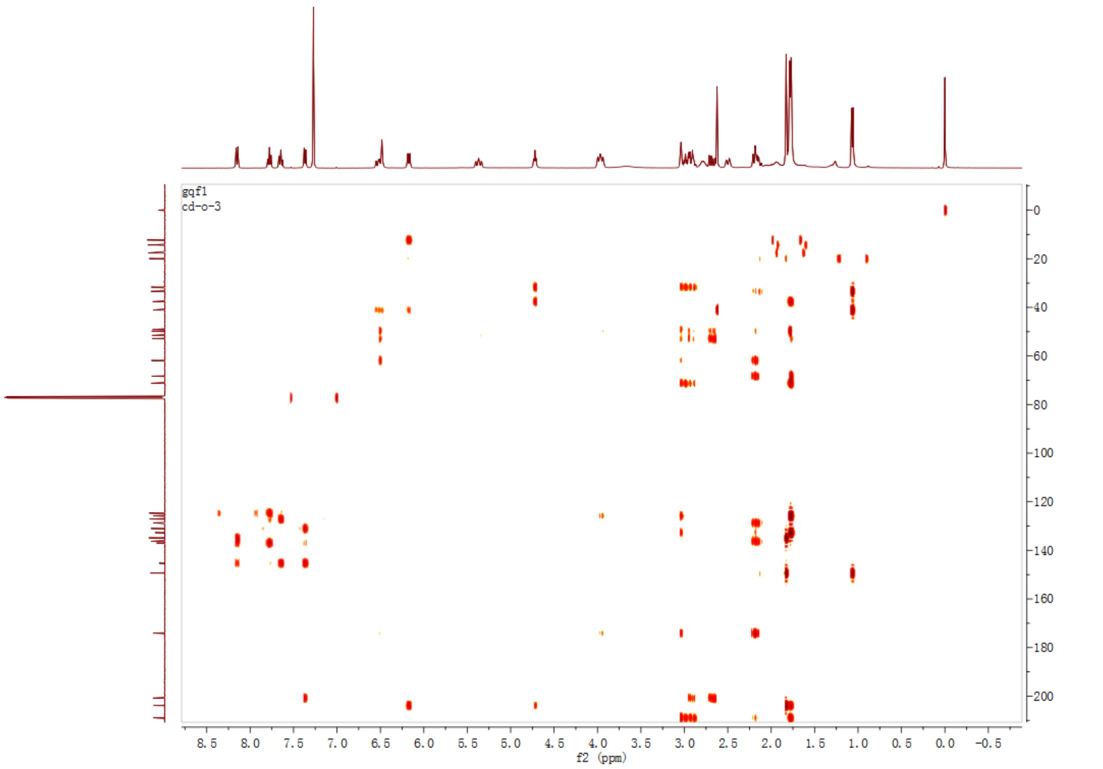

Figure S87. HMBC spectrum of compound **10** in $CDCl_3$

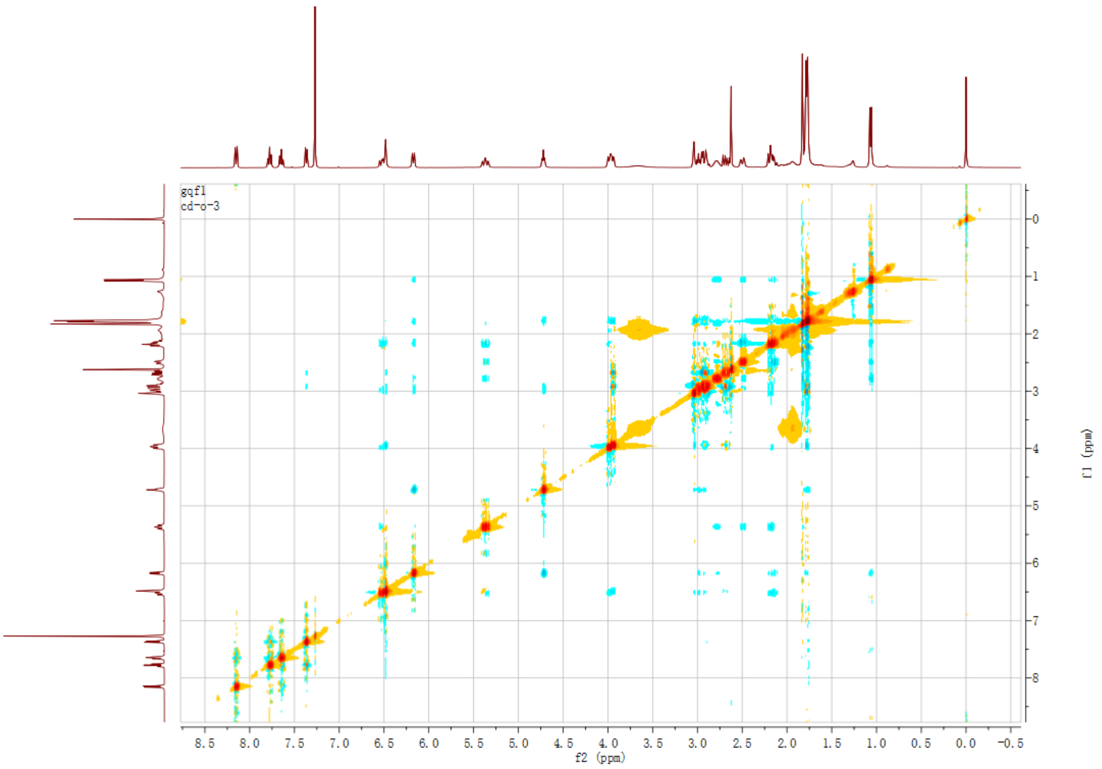

Figure S88. NOESY spectrum of compound **10** in $CDCl_3$

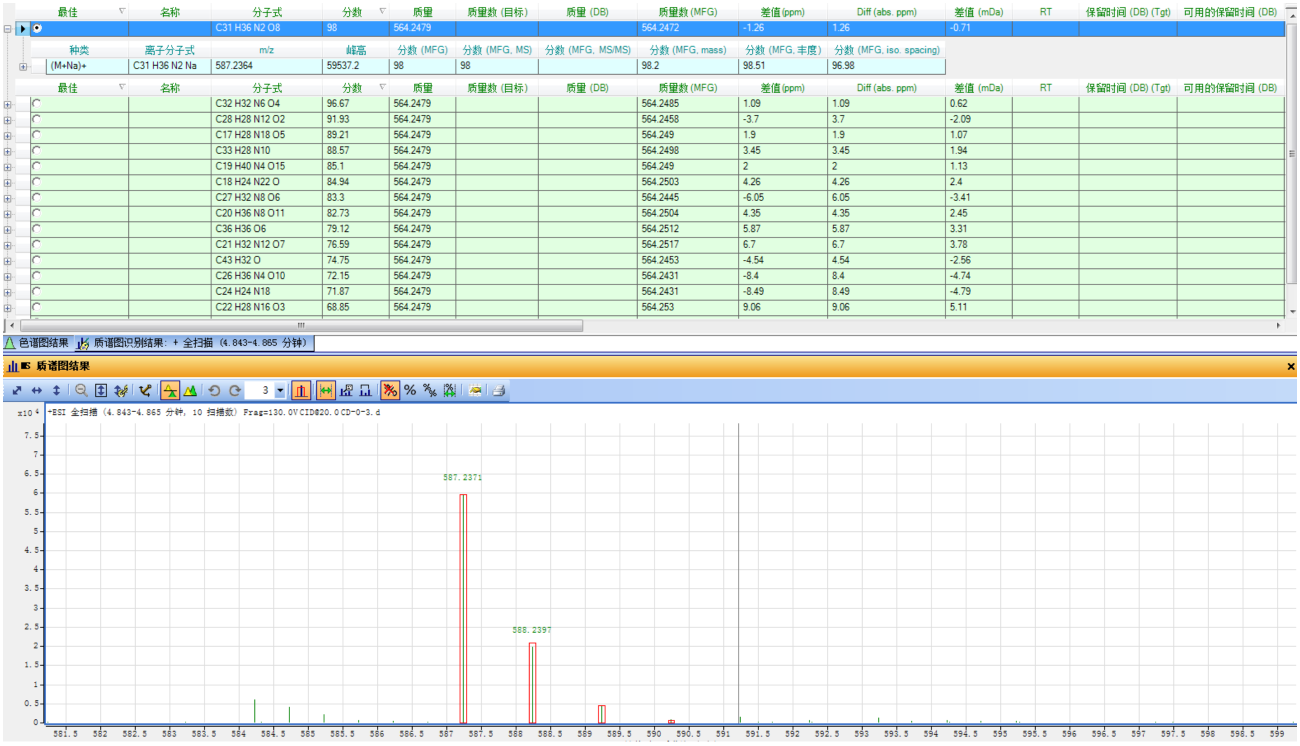

最佳	名称	分子式	分数	质量	质量数 (目标)	质量 (DB)	质量数 (MFG)	差值 (ppm)	Diff (abs. ppm)	差值 (mDa)	RT	保留时间 (DB) (Tgt)	可用的保留时间 (DB)
◉		C31 H36 N2 O8	98	564.2479			564.2472	-1.26	1.26	-0.71			

种类	离子分子式	m/z	峰高	分数 (MFG)	分数 (MFG, MS)	分数 (MFG, MS/MS)	分数 (MFG, mass)	分数 (MFG, 丰度)	分数 (MFG, iso. spacing)
(M+Na)+	C31 H36 N2 Na	587.2364	59537.2	98	98		98.2	98.51	96.98

最佳	名称	分子式	分数	质量	质量数 (目标)	质量 (DB)	质量数 (MFG)	差值 (ppm)	Diff (abs. ppm)	差值 (mDa)	RT	保留时间 (DB) (Tgt)	可用的保留时间 (DB)
○		C32 H32 N6 O4	96.67	564.2479			564.2485	1.09	1.09	0.62			
○		C28 H28 N12 O2	91.93	564.2479			564.2458	-3.7	3.7	-2.09			
○		C17 H28 N18 O5	89.21	564.2479			564.249	1.9	1.9	1.07			
○		C33 H28 N10	88.57	564.2479			564.2498	3.45	3.45	1.94			
○		C19 H40 N4 O15	85.1	564.2479			564.249	2	2	1.13			
○		C18 H24 N22 O	84.94	564.2479			564.2503	4.26	4.26	2.4			
○		C27 H32 N8 O6	83.3	564.2479			564.2445	-6.05	6.05	-3.41			
○		C20 H36 N8 O11	82.73	564.2479			564.2504	4.35	4.35	2.45			
○		C36 H36 O6	79.12	564.2479			564.2512	5.87	5.87	3.31			
○		C21 H32 N12 O7	76.59	564.2479			564.2517	6.7	6.7	3.78			
○		C43 H32 O	74.75	564.2479			564.2453	-4.54	4.54	-2.56			
○		C26 H36 N4 O10	72.15	564.2479			564.2431	-8.4	8.4	-4.74			
○		C24 H24 N18	71.87	564.2479			564.2431	-8.49	8.49	-4.79			
○		C22 H28 N16 O3	68.85	564.2479			564.253	9.06	9.06	5.11			

Figure S89. HRESIMS spectrum of compound **10**

打印窗口 80: MS Spectrum

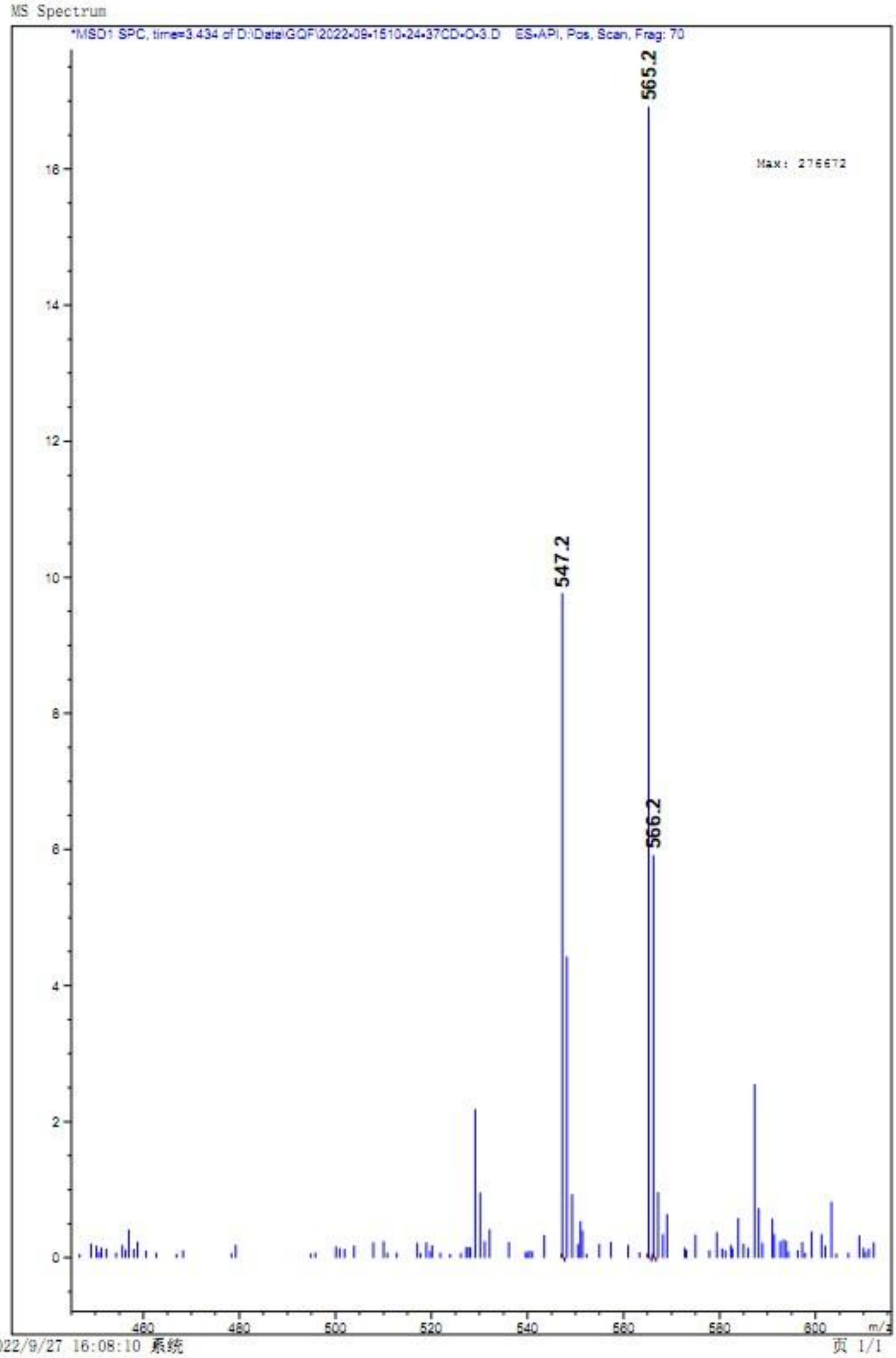

120 2022/9/27 16:08:10 系统 页 1/1

Figure S90. EIMS spectrum of Compound **10**

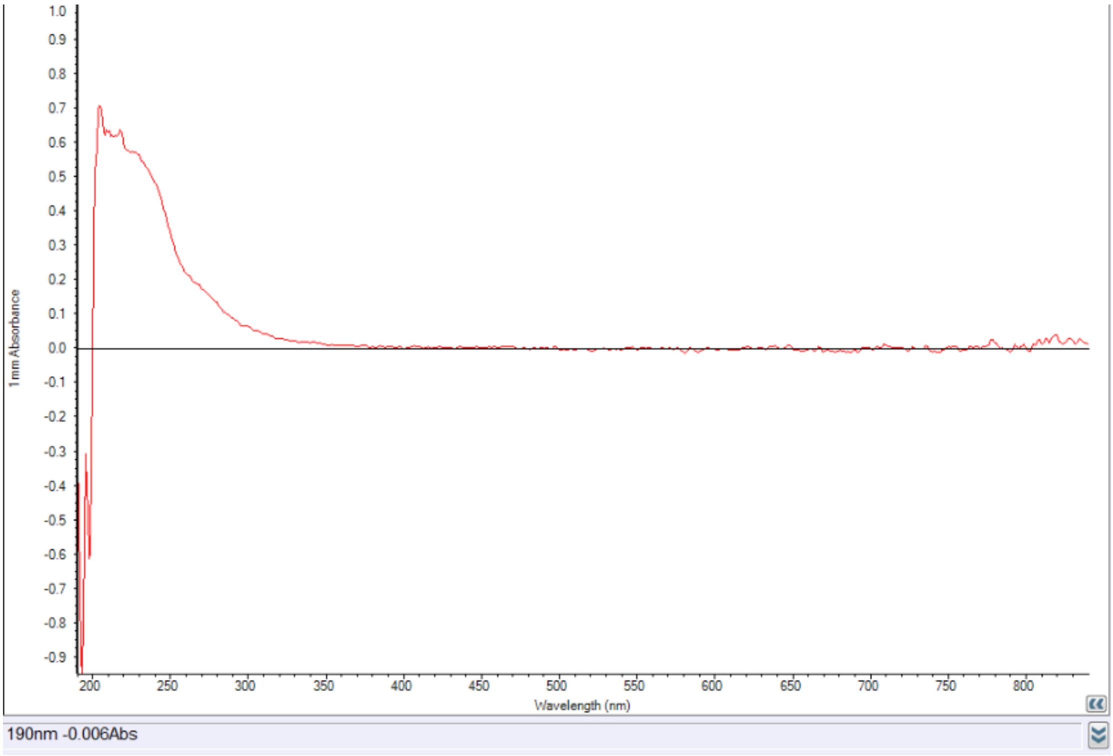

Figure S91. UV spectrum of Compound **10**

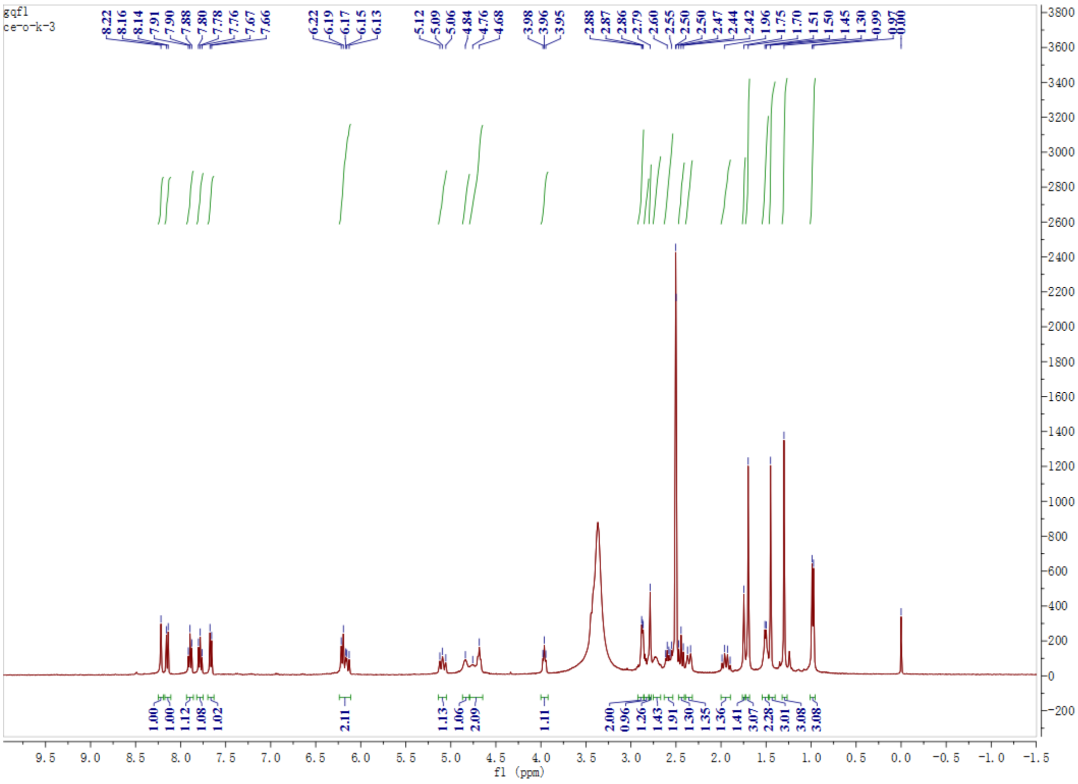

Figure S92. ^{1}H NMR spectrum of compound **11** in DMSO-d_6

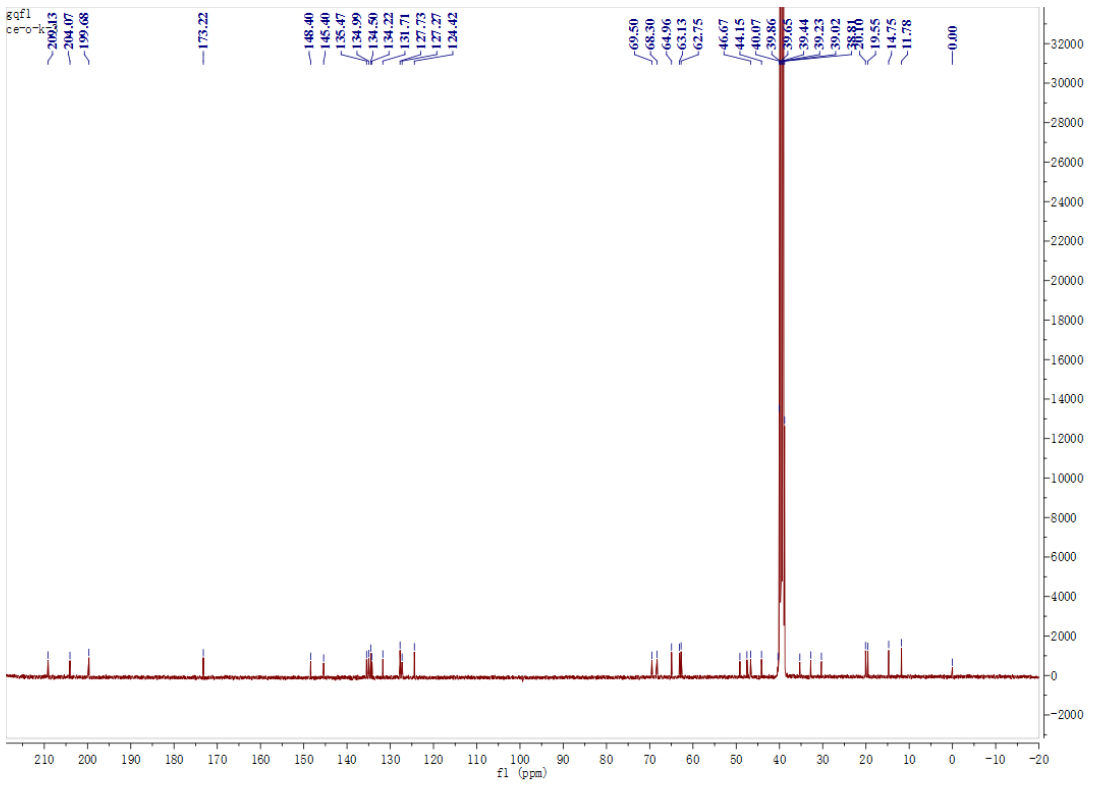

Figure S93. ^{13}C NMR spectrum of compound **11** in DMSO-d_6

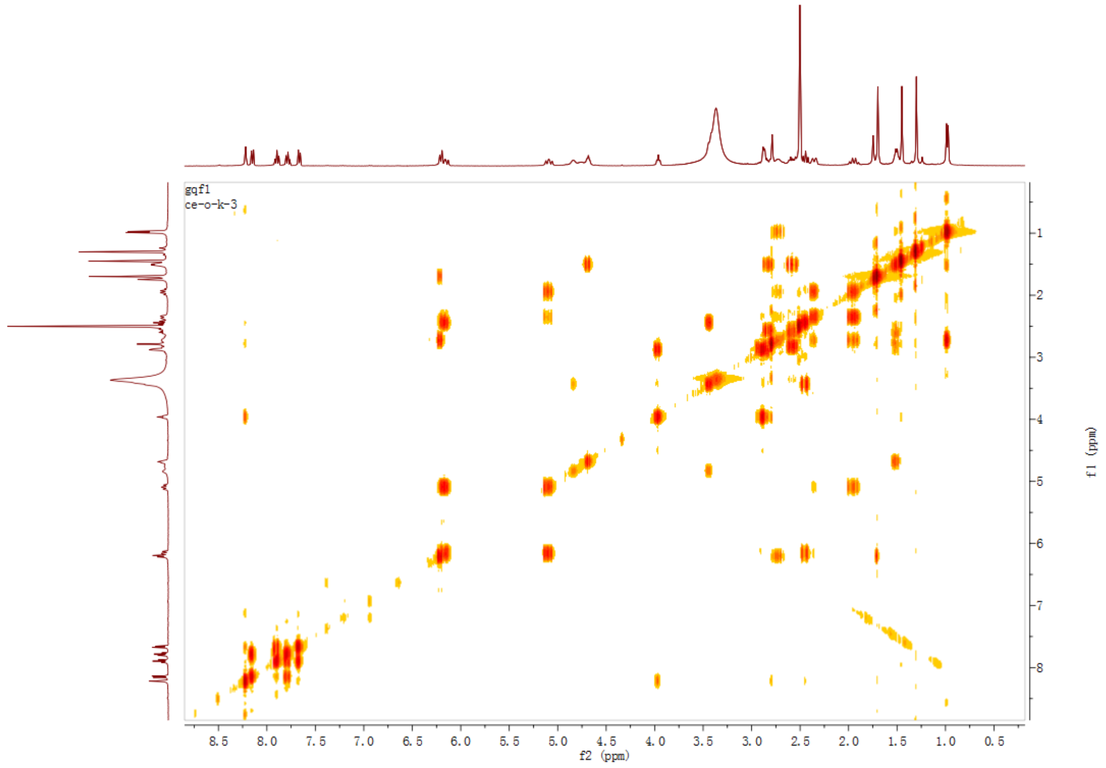

Figure S94. COSY spectrum of compound **11** in DMSO-d_6

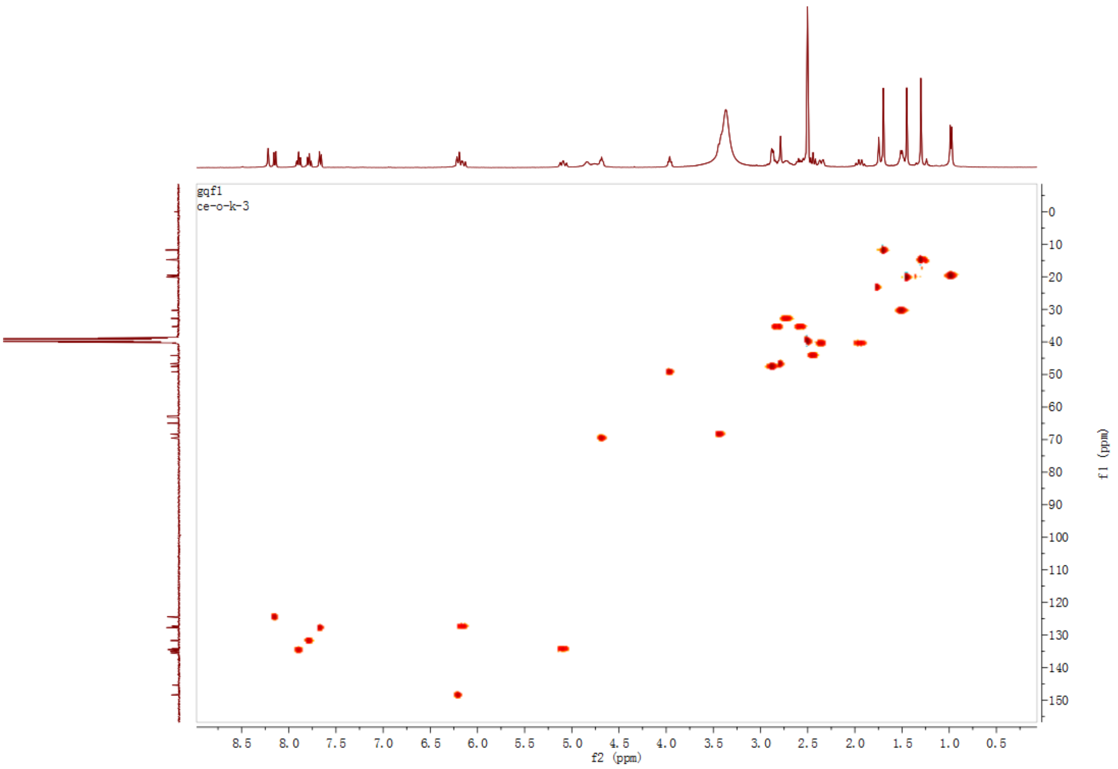

Figure S95. HSQC spectrum of compound **11** in DMSO-d_6

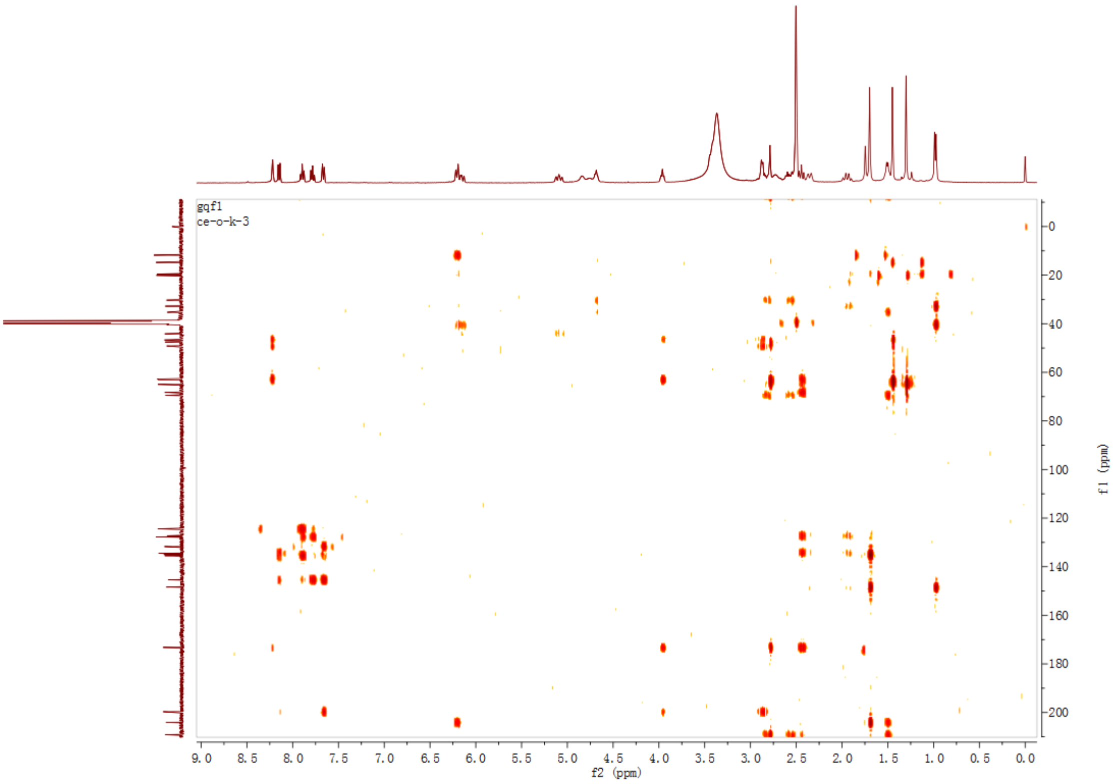

Figure S96. HMBC spectrum of compound **11** in DMSO-d_6

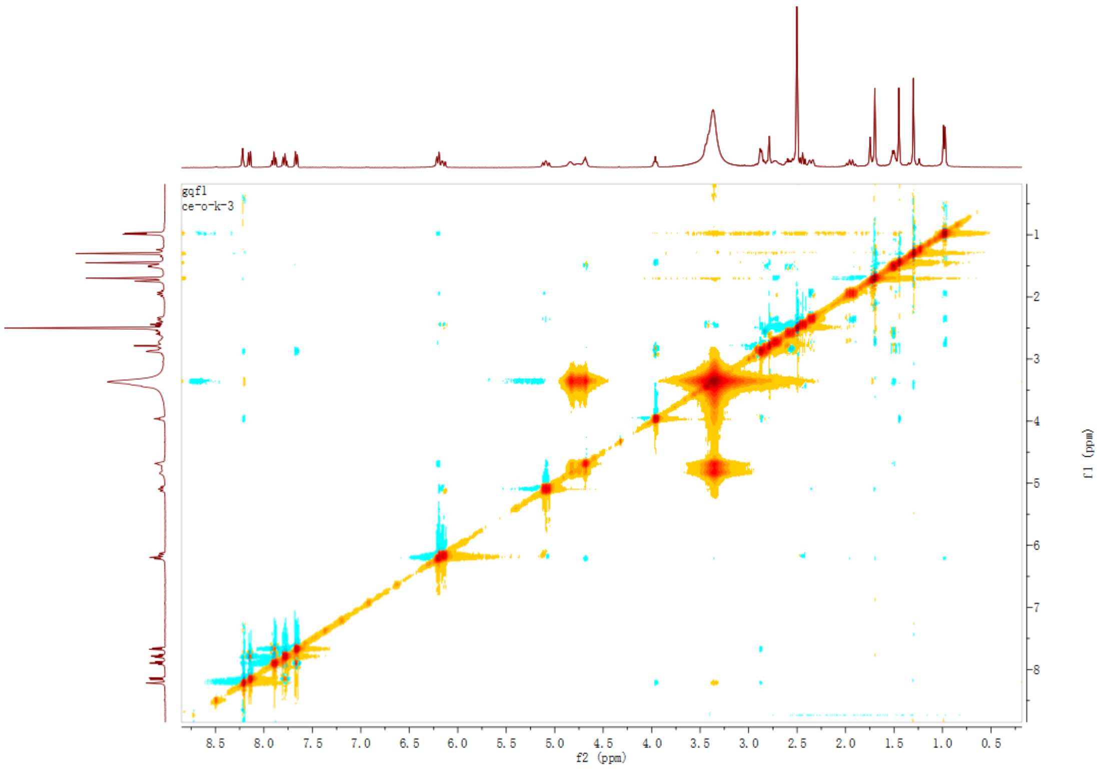

Figure S97. NOESY spectrum of compound **11** in DMSO-d_6

Spectrum Plot Report

Name	Sample2	Rack Pos.		Instrument	Instrument 1	Operator	
Inj. Vol. (ul)	5	Plate Pos.		IRM Status	Some ions missed		
Data File	CE-0-K-3R.d	Method (Acq)	CL-2022.m	Comment		Acq. Time (Local)	7/29/2022 4:48:28 PM (UTC+08:00)

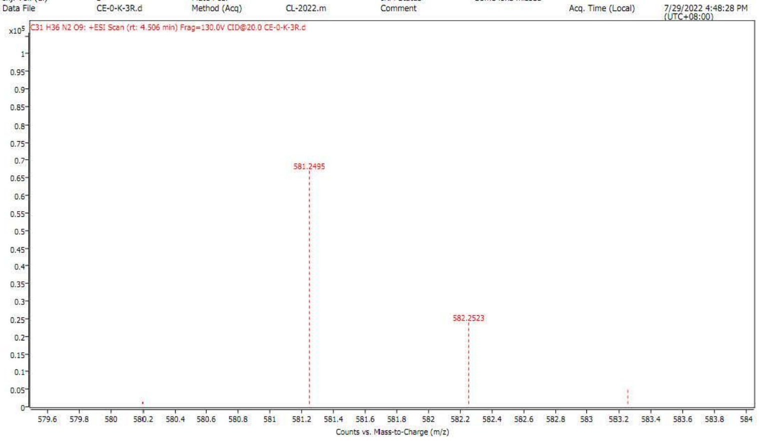

Page 1 of 1 Generated at 10:38 AM on 8/9/2022

Analysis Report

Spectrum Peaks

m/z	Z	Abund	Abund %	m/z (Calc)	Diff (ppm)	Ion Species	Formula	Ion Type
581.2495	1	67373	15.36	581.2494	0.19	(M+H)+	C31 H36 N2 O9	
582.2523	1	24373	5.56	582.2526	-0.50	(M+H)+	C31 H36 N2 O9	
101.0577		8651	1.97					
103.0733		9625	2.19					
109.0631		32538	7.42					
109.0991		10870	2.48					
119.0834		7211	1.64					
121.0485		16878	3.85					
121.0594		9670	2.20					
121.0987		10686	2.44					
123.0783		9346	2.13					
133.0846		39714	9.05					
135.0786		19516	4.45					
137.0941		28388	6.47					
147.0778		10892	2.48					

Spectrum Identification Table

Best	ID Source	Name	Formula	Species	m/z	Diff (ppm)	CAS	Score	Score (Lib)	Score (DB)	Score (MFG)	Lib/DB
Yes	MFG		C31 H36 N2 O9	(M+H)+	581.2495	-0.34		98.84			93.84	
No	MFG		C30 H30 N9 O4	(M+H)+	581.2495	-0.07		99.27			99.27	
No	MFG		C29 H34 N5 O8	(M+H)+	581.2495	2.10		96.20			95.20	
No	MFG		C28 H28 N12 O3	(M+H)+	581.2495	2.38		95.92			95.92	
No	MFG		C32 H32 N6 O5	(M+H)+	581.2495	-2.51		95.50			95.50	

MassHunter Qual 10.0
(End of Report)

Figure S98. HRESIMS spectrum of compound **11**

打印窗口 80: 峰的顶点质谱3.334 的 2022-09-1510-24-23CD-0-2.D

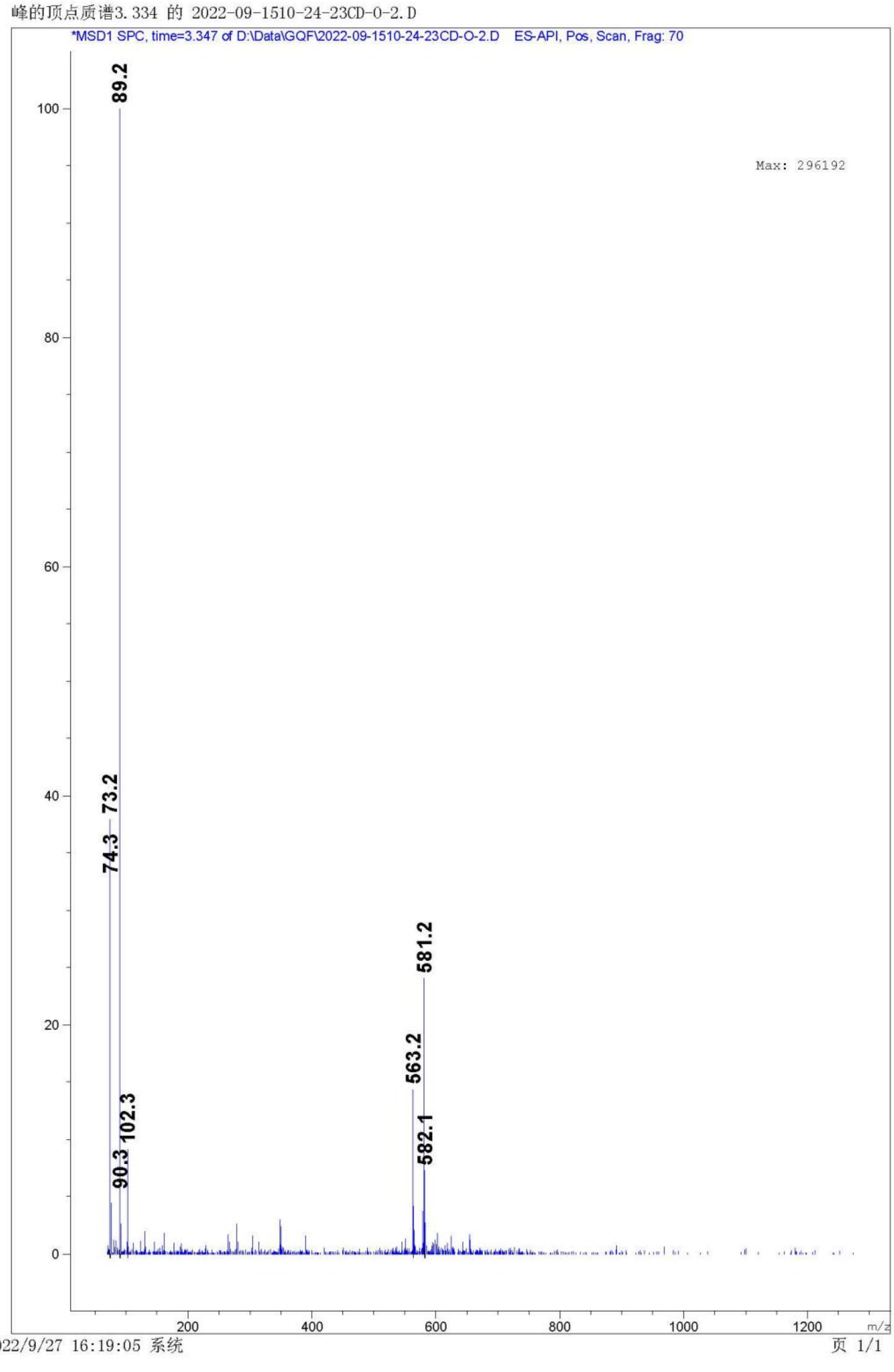

6120 2022/9/27 16:19:05 系统 页 1/1

Figure S99. EIMS spectrum of Compound **11**

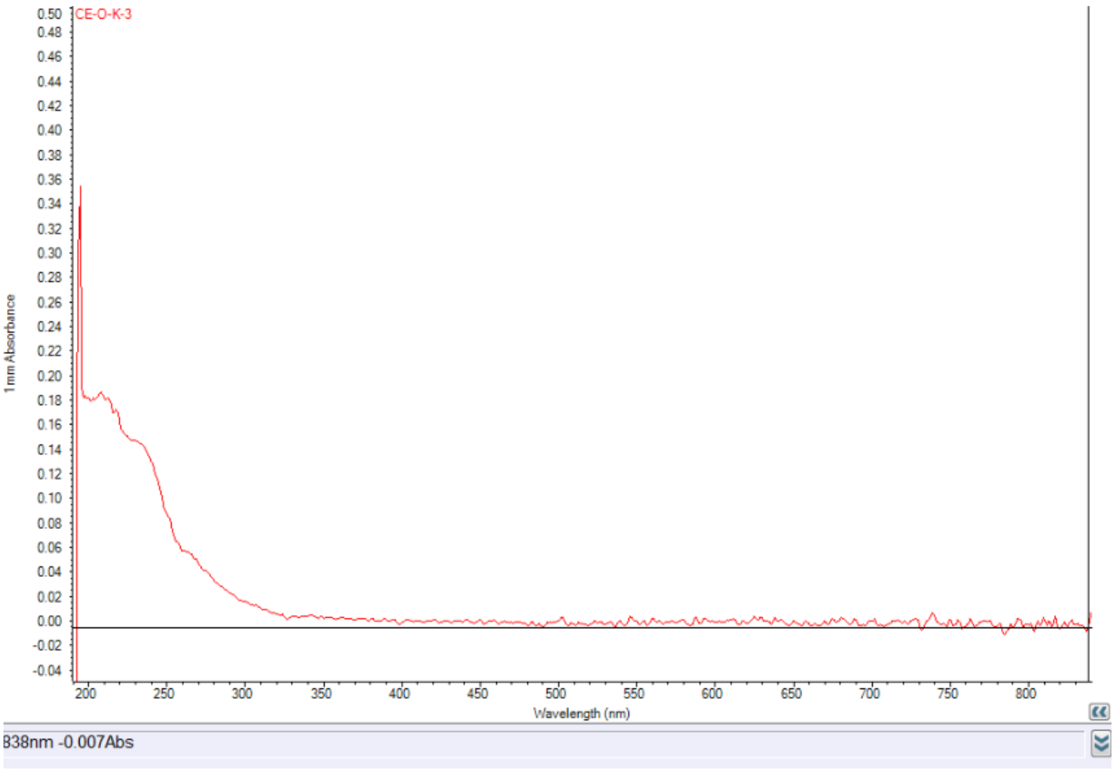

Figure S100. UV spectrum of Compound **11**

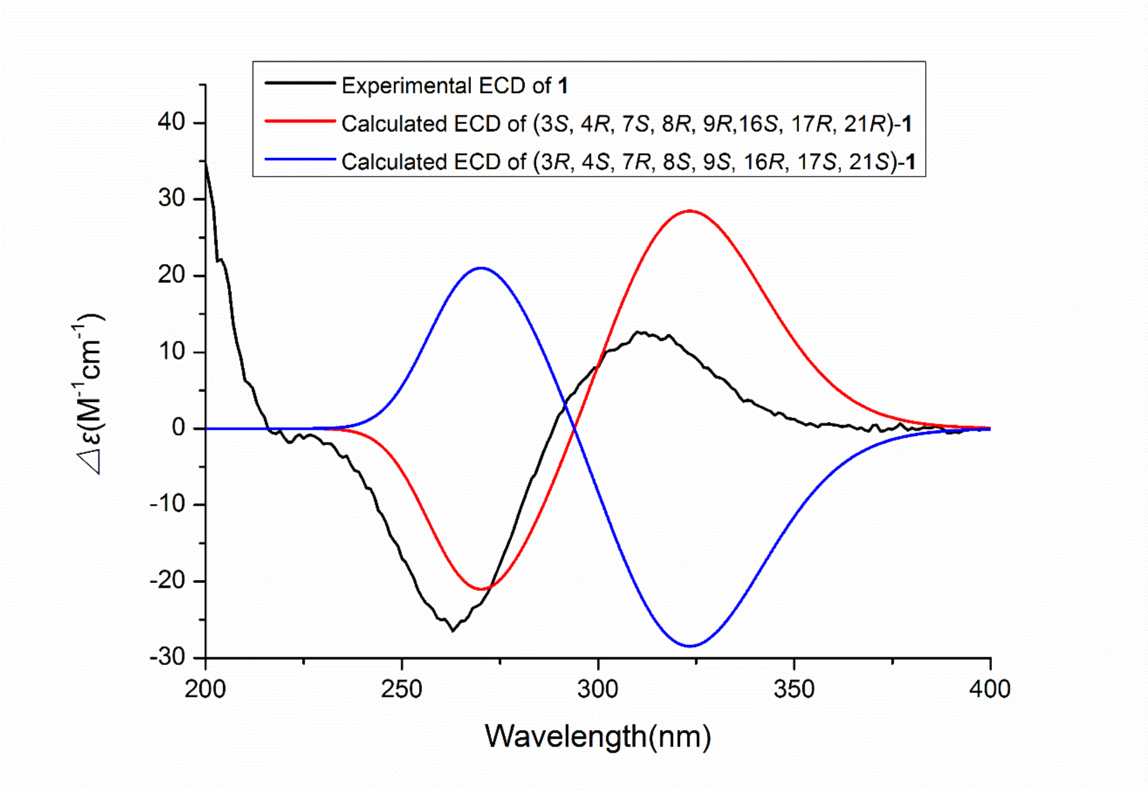

Figure S101. Experimental ECD spectra of **1** and calculated ECD spectra for (3*S*, 4*R*, 7*S*, 8R, 9*R*, 17*R*, 21*R*)-**1** and (3*R*, 4*S*, 7*R*, 9*S*, 16*S*, 21*S*)-**1**.

Table S1. Gibbs free energies[a] and equilibrium populations[b] of low-energy conformers of (3*S*, 4*R*, 7*S*, 8R, 9*R*, 17*R*, 21*R*)-**1**.

Conformers	In MeOH	
	Δ*G (kJ/mol)*	*P* (%)/100
**1a1**	0.00	98.64
**1a4**	10.71	1.36

[a]B3LYP/6-31+G(d,p), in kcal/mol. [b]From Δ*G* values at 298.15K.

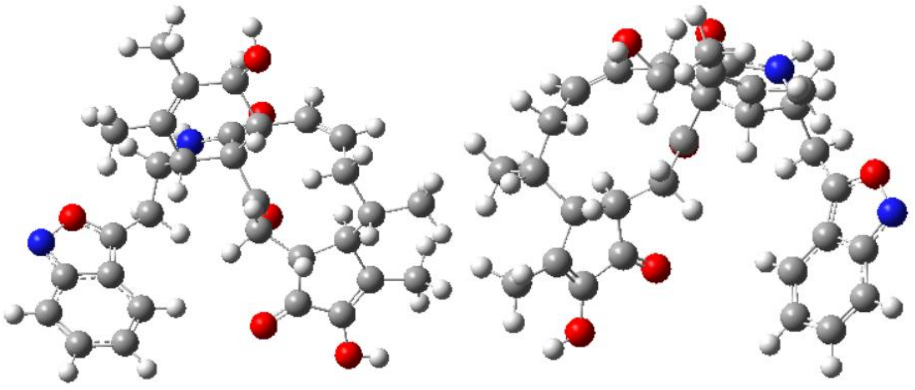

1a1 **1a4**

Figure S102. Optimized geometries of predominant conformers for compound (3*S*, 4*R*, 7*S*, 8R, 9*R*, 17*R*, 21*R*)-**1** at the B3LYP/6-31G(d,p) level in the gas phase.

Table S2. Cartesian coordinates for the low-energy reoptimized MMFF conformers of (3*S*, 4*R*, 7*S*, 8R, 9*R*, 17*R*, 21*R*)-**1** at B3LYP/6-31G (d, p) level of theory in MeOH

1a1				1a4			
Symbol	X	Y	Z	Symbol	X	Y	Z
C	-1.8938000	2.6147000	0.6107000	C	1.7155	-2.3847	-1.1886
C	-1.3771000	1.3933000	-0.1321000	C	1.4860	-1.5120	0.0473
C	0.1107000	1.4952000	-0.6025000	C	0.1988	-1.8535	0.8758
C	0.9638000	2.4352000	0.3020000	C	-0.8462	-2.7878	0.1624
C	0.2973000	3.8154000	0.3930000	C	-0.7523	-2.7479	-1.3653
C	-1.1678000	3.7295000	0.7910000	C	0.6841	-2.9418	-1.8469
C	2.4056000	2.5584000	-0.1396000	C	-2.2066	-2.6325	0.8230
C	0.7433000	0.0811000	-0.6266000	C	-0.4994	-0.5479	1.3384
C	0.8985000	-0.6412000	0.7001000	C	-0.9510	0.4269	0.2572
C	-4.5429000	-3.8713000	1.7088000	C	3.7744	4.3336	-1.2358
C	-5.4481000	-2.9261000	1.3138000	C	4.7544	3.4014	-1.4345
C	-5.0010000	-1.9363000	0.3878000	C	4.6499	2.1748	-0.7117
C	-3.6459000	-1.9511000	-0.0965000	C	3.5455	1.9499	0.1825
C	-2.7331000	-2.9560000	0.3382000	C	2.5478	2.9502	0.3665

C	-3.1903000	-3.8908000	1.2251000	C	2.6740	4.1150	-0.3380
N	-5.7019000	-0.9363000	-0.1302000	N	5.4804	1.1409	-0.7433
C	-3.5895000	-0.8712000	-0.9529000	C	3.7796	0.6882	0.6874
C	-2.5304000	-0.2834000	-1.8188000	C	3.0722	-0.1801	1.6678
O	1.0567000	-0.4588000	-1.6686000	O	-0.6403	-0.2836	2.5135
C	-0.0179000	1.9965000	-2.0617000	C	0.7511	-2.5942	2.1169
N	-1.2891000	1.7681000	-2.4577000	N	2.0826	-2.3851	2.1658
C	-2.1789000	1.1870000	-1.4660000	C	2.6396	-1.5620	1.1065
H	-1.5312000	0.5218000	0.5096000	H	1.4280	-0.4845	-0.3251
O	0.8580000	2.5088000	-2.7447000	O	0.1062	-3.2786	2.8992
C	3.4947000	2.0285000	0.4284000	C	-3.3745	-2.0925	0.4553
C	3.6118000	1.1322000	1.6332000	C	-3.8516	-1.4576	-0.8226
C	4.3326000	-0.2216000	1.3414000	C	-4.4147	-0.0116	-0.6941
C	3.5282000	-1.0332000	0.2842000	C	-3.2980	1.0690	-0.7002
C	4.1395000	-2.3053000	-0.2990000	C	-3.8316	2.4896	-0.7860
C	3.2532000	-3.3263000	-0.2903000	C	-3.2328	3.2949	0.1166
C	1.9658000	-2.9153000	0.2601000	C	-2.2108	2.5713	0.8779
C	2.1553000	-1.5182000	0.8366000	C	-2.3221	1.0929	0.5086
C	5.8093000	0.0354000	0.9985000	C	-5.4146	0.1439	0.4607
C	5.4814000	-2.3917000	-0.9603000	C	-4.8366	2.8968	-1.8132
O	0.9288000	-3.5617000	0.2699000	O	-1.3976	3.0637	1.6438
C	-1.7330000	4.9923000	1.3977000	C	0.8135	-3.7179	-3.1347
C	-3.3220000	2.4762000	1.1033000	C	3.1433	-2.4314	-1.6902
H	0.9246000	2.0302000	1.3151000	H	-0.5287	-3.8064	0.4101
H	2.5511000	3.1700000	-1.0238000	H	-2.1794	-3.0350	1.8317
H	0.0380000	-1.3184000	0.7760000	H	-0.9289	-0.0450	-0.7227
H	0.8050000	0.0445000	1.5382000	H	-0.1774	1.2032	0.2322
H	-4.8485000	-4.6392000	2.4106000	H	3.8228	5.2769	-1.7680
H	-6.4670000	-2.9176000	1.6801000	H	5.5834	3.5744	-2.1092
H	-1.7108000	-2.9817000	-0.0201000	H	1.7207	2.7948	1.0487
H	-2.5255000	-4.6704000	1.5776000	H	1.9359	4.8995	-0.2222
H	-2.8799000	-0.3002000	-2.8568000	H	2.2070	0.3603	2.0560
H	-1.6426000	-0.9153000	-1.7747000	H	3.7376	-0.3617	2.5188
H	-1.5885000	1.9947000	-3.3965000	H	2.6441	-2.7686	2.9142
H	-3.1084000	1.7562000	-1.4426000	H	3.5229	-2.0516	0.7021
H	4.4425000	2.2739000	-0.0461000	H	-4.1507	-2.1518	1.2160
H	2.6384000	0.9490000	2.0886000	H	-4.6767	-2.0803	-1.1923
H	4.2063000	1.6502000	2.3956000	H	-3.0919	-1.4741	-1.6035
H	4.3159000	-0.7918000	2.2779000	H	-4.9727	0.1522	-1.6229
				H	-2.7140	0.8957	-1.6142
				H	-2.7908	0.6144	1.3738
				H	-6.1874	-0.6279	0.4021
				H	-5.9148	1.1144	0.4218

				H	-4.9412	0.0592	1.4419
				H	-4.9359	3.9816	-1.8982
				H	-4.5545	2.5103	-2.7975
				H	-5.8286	2.4934	-1.5853
				H	1.8369	-3.7745	-3.4961
				H	0.4499	-4.7411	-3.0088
				H	0.2088	-3.2617	-3.9285
				H	3.7389	-3.1859	-1.1647
				H	3.6433	-1.4708	-1.5412

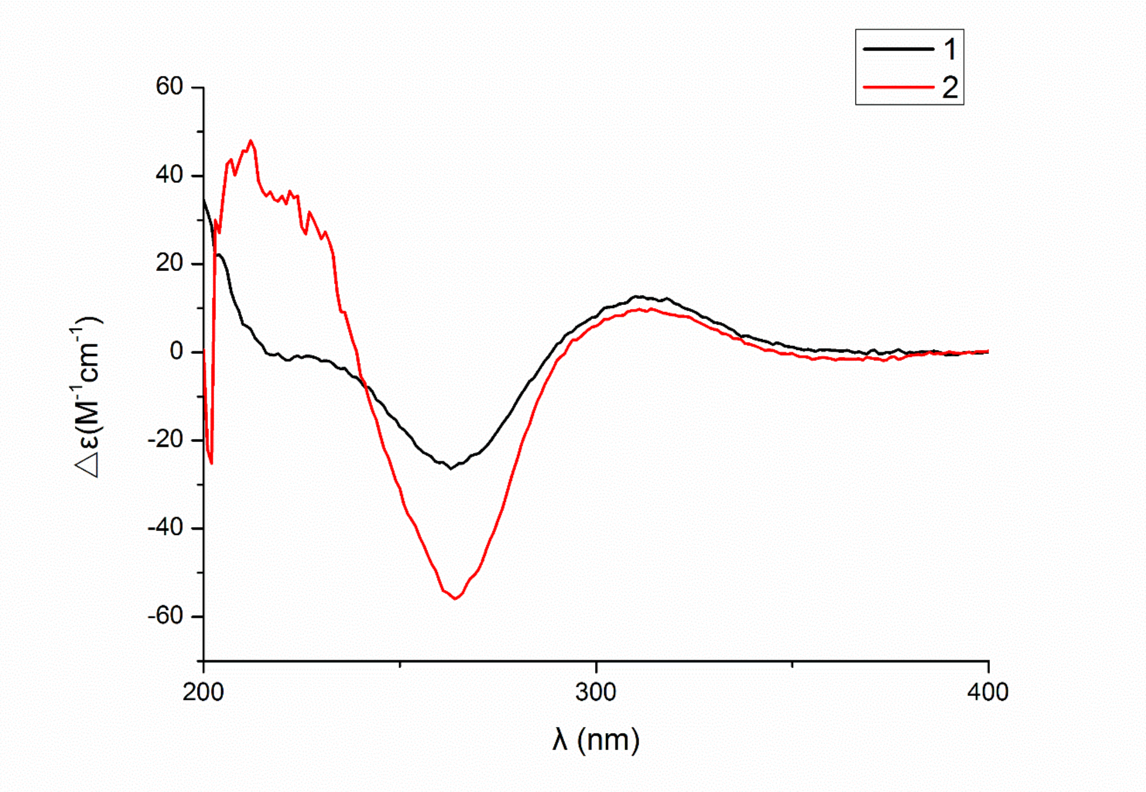

Figure S103. Experimental ECD spectra of compounds **1** and **2** in MeOH

Table S3. Gibbs free energies[a] and equilibrium populations[b] of low-energy conformers of (3*S*, 4*R*, 7*S*, 8*R*, 9*R*, 17*R*, 18S, 21*R*)-**3**.

Conformers	In MeOH	
	Δ*G (Ha)*	*P* (%)/100
**3a**	-2062.864787	50.1
**3b**	-2062.861766	49.9

[a]B3LYP/6-31+G(d,p), in kcal/mol. [b]From Δ*G* values at 298.15K.

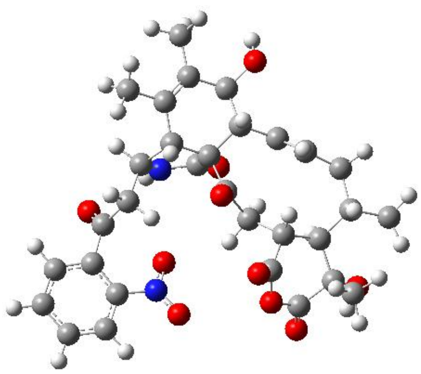

3a

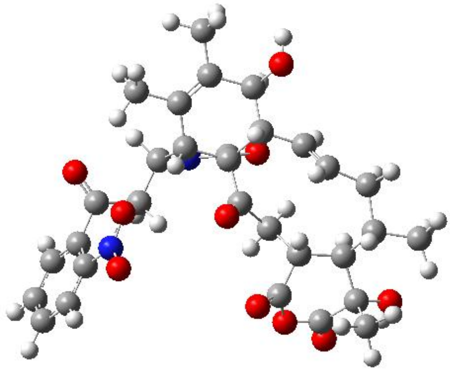

3b

Figure S104. Optimized geometries of predominant conformers for compound (3*S*, 4*R*, 7*S*, 8*R*, 9*R*, 17*R*, 18S, 21*R*)-**3** at the B3LYP/6-31G(d,p) level in the gas phase.

Table S4. Cartesian coordinates for the low-energy reoptimized MMFF conformers of (3*S*, 4*R*, 7*S*, 8*R*, 9*R*, 17*R*, 18S, 21*R*)-**3** at B3LYP/6-31G (d, p) level of theory in MeOH.

3a				3b			
Symbol	X	Y	Z	Symbol	X	Y	Z
C	-1.4636	3.5172	-1.4708	C	-1.7317	2.9747	-1.3609
C	-1.4385	2.0295	-1.1489	C	-1.4269	1.5098	-1.0774
C	-0.1289	1.5354	-0.4824	C	-0.0667	1.2674	-0.3737
C	0.5417	2.6543	0.3837	C	0.3350	2.4628	0.5558
C	0.8185	3.8846	-0.4851	C	0.3892	3.7574	-0.2595
C	-0.4314	4.3450	-1.2254	C	-0.8972	3.9816	-1.0432
C	1.7595	2.1485	1.1135	C	1.6034	2.1821	1.3209
C	-0.3948	0.3705	0.5154	C	-0.1169	0.0386	0.5771
C	0.6795	-0.6923	0.7300	C	1.1453	-0.7951	0.7873
C	-4.4880	-4.7417	-3.3029	C	-5.2772	-4.6491	-2.3331
C	-3.3537	-4.5875	-2.5115	C	-5.2503	-3.7714	-1.2541
C	-2.6327	-3.3956	-2.5862	C	-4.6317	-2.5298	-1.4062
C	-3.0132	-2.3382	-3.4277	C	-4.0335	-2.1314	-2.6100
C	-4.1470	-2.5260	-4.2246	C	-4.0871	-3.0256	-3.6840
C	-4.8805	-3.7124	-4.1619	C	-4.6985	-4.2735	-3.5480
C	-2.3105	-0.9989	-3.5272	C	-3.3340	-0.8031	-2.8403
C	-2.4120	-0.0627	-2.3357	C	-1.8856	-0.7172	-2.3834
O	-1.4287	0.3569	1.1586	O	-1.1363	-0.2009	1.1975
C	0.7250	1.0637	-1.6809	C	0.9043	1.0317	-1.5516
N	-0.1227	0.8677	-2.7163	N	0.1493	0.6935	-2.6223
C	-1.5233	1.1858	-2.4680	C	-1.2966	0.7074	-2.4208
H	-2.2914	1.7911	-0.5050	H	-2.2370	1.0863	-0.4767
O	1.9408	0.8780	-1.7019	O	2.1317	1.1128	-1.5355
C	1.7759	1.7894	2.4012	C	1.6477	1.7765	2.5943
C	2.9532	1.1339	3.0652	C	2.9107	1.3476	3.2861

C	2.7621	-0.2755	3.7102	C	2.9950	-0.0998	3.8675
C	2.2806	-1.3709	2.6970	C	2.7886	-1.2202	2.7907
C	0.7947	-1.1292	2.2301	C	1.3017	-1.2680	2.2720
C	4.1092	-0.5938	4.3927	C	4.3531	-0.1654	4.5970
C	-2.7518	3.9820	-2.1296	C	-3.0611	3.2129	-2.0599
C	-0.4083	5.7949	-1.6493	C	-1.1608	5.4213	-1.4176
O	1.2934	4.8967	0.4162	O	0.6092	4.8065	0.6967
C	-0.1132	-2.3312	2.4104	C	0.6581	-2.6381	2.3622
C	1.7703	-3.7745	2.2022	C	2.8073	-3.6466	2.1677
C	2.4630	-2.8446	3.2022	C	3.2555	-2.6486	3.2396
O	-1.2571	-2.3343	2.7689	O	-0.4703	-2.8973	2.6715
O	2.3183	-4.7068	1.6630	O	3.5561	-4.4141	1.6105
C	1.8560	-3.1339	4.5971	C	2.6718	-3.1276	4.5915
H	0.3395	-0.3542	2.8468	H	0.6746	-0.6338	2.8982
H	2.9311	-1.3151	1.8165	H	3.4427	-0.9870	1.9425
O	-1.8183	-0.6550	-4.5892	O	-3.8719	0.0529	-3.5198
O	3.8379	-3.1850	3.1981	O	4.6701	-2.6981	3.2837
O	0.4415	-3.5553	1.9985	O	1.4693	-3.6958	1.9123
N	-1.4083	-3.2904	-1.7943	N	-4.6545	-1.6067	-0.2723
O	-0.6128	-2.3864	-2.0879	O	-4.2133	-0.4647	-0.4542
O	-1.2180	-4.0954	-0.8860	O	-5.1144	-1.9952	0.7993
H	-0.2143	2.9507	1.1227	H	-0.4915	2.5716	1.2702
H	1.6178	3.6476	-1.2051	H	1.2495	3.7182	-0.9465
H	2.6407	1.9717	0.4994	H	2.5239	2.2174	0.7408
H	0.4111	-1.5429	0.0853	H	1.0749	-1.6554	0.1052
H	1.6402	-0.3380	0.3620	H	2.0244	-0.2395	0.4672
H	-5.0539	-5.6662	-3.2578	H	-5.7554	-5.6172	-2.2278
H	-3.0095	-5.3770	-1.8551	H	-5.7058	-4.0253	-0.3053
H	-4.4598	-1.7274	-4.8897	H	-3.6394	-2.7417	-4.6312
H	-5.7589	-3.8321	-4.7882	H	-4.7241	-4.9516	-4.3953
H	-3.4645	0.2519	-2.2725	H	-1.7735	-1.1857	-1.4011
H	-2.2099	-0.6060	-1.4093	H	-1.3290	-1.3570	-3.0854
H	0.1926	0.4847	-3.5983	H	0.5811	0.4628	-3.5090
H	-1.8994	1.7870	-3.2989	H	-1.7706	1.2445	-3.2450
H	0.8915	1.9620	3.0184	H	0.7254	1.7364	3.1778
H	3.3061	1.7852	3.8778	H	3.0896	2.0190	4.1383
H	3.7783	1.0686	2.3432	H	3.7574	1.4884	2.6007
H	1.9934	-0.1899	4.4918	H	2.1977	-0.2123	4.6164
H	4.1079	-1.5256	4.9570	H	4.5249	-1.1035	5.1233
H	4.3606	0.2184	5.0840	H	4.4046	0.6468	5.3309
H	4.9128	-0.6658	3.6518	H	5.1806	-0.0339	3.8914
H	-3.5675	3.2798	-1.9248	H	-3.6753	2.3083	-2.0580
H	-3.0676	4.9621	-1.7613	H	-3.6358	4.0053	-1.5693

H	-2.6615	4.0591	-3.2212	H	-2.9330	3.5087	-3.1093
H	0.5212	6.0195	-2.1921	H	-0.2735	5.8644	-1.8923
H	-1.2375	6.0548	-2.3090	H	-1.9905	5.5273	-2.1184
H	-0.4365	6.4656	-0.7820	H	-1.3822	6.0305	-0.5326
H	1.7544	5.5686	-0.1091	H	0.9236	5.5861	0.2134
H	2.3908	-2.5653	5.3584	H	3.0526	-2.5030	5.4001
H	1.9664	-4.1983	4.8265	H	2.9887	-4.1582	4.7788
H	0.7950	-2.8766	4.6687	H	1.5790	-3.0934	4.6267
H	3.9109	-4.0170	2.6916	H	4.9333	-3.4701	2.7465

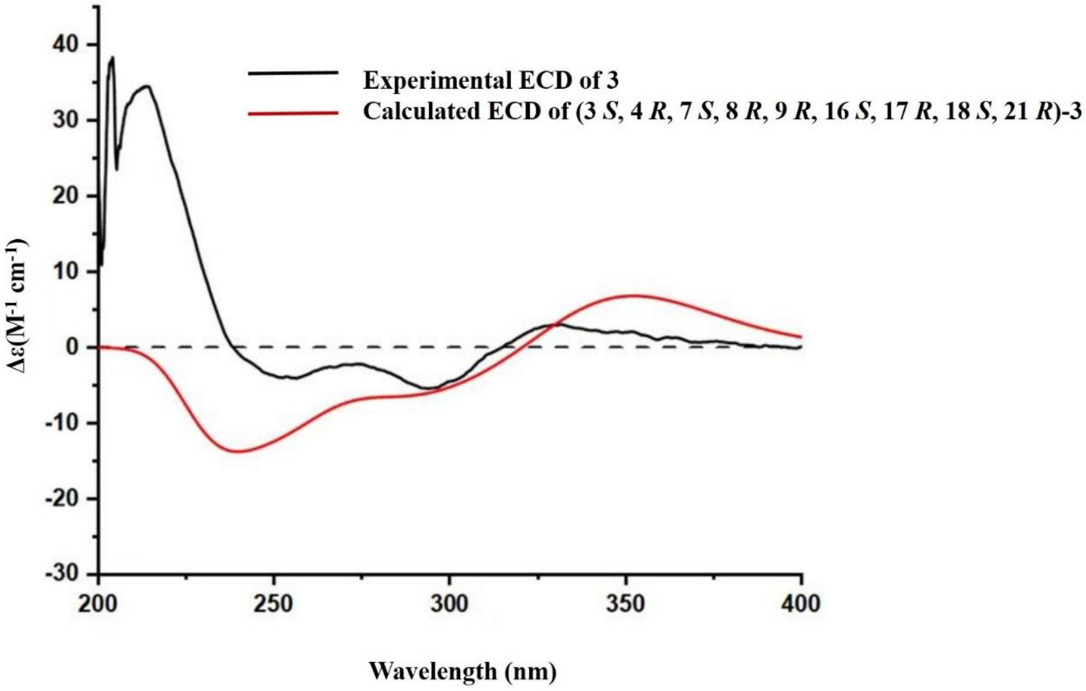

Figure 105. Experimental and calculated ECD spectra of Compound **3**

Table S5. Gibbs free energies[a] and equilibrium populations[b] of low-energy conformers of (3*S*, 4*R*, 6S, 7*S*, 8*R*, 9*S*, 16*S*, 17*R*, 18*S*, 21*R*)-**4**.

Conformers	In MeOH	
	Δ*G (Ha)*	*P* (%)/100
**4a**	-2138.051308	33.7
**4b**	-2138.027132	32.3
**4c**	-2138.055715	34.0

[a]B3LYP/6-31+G(d,p), in kcal/mol. [b]From Δ*G* values at 298.15K.

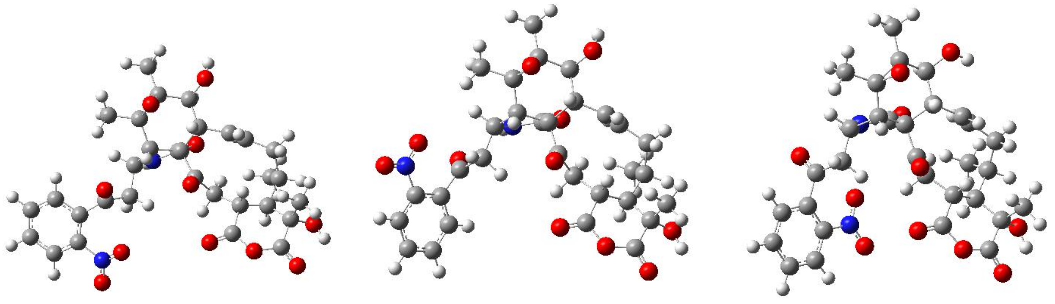

4a **4b** **4c**

Figure S106. Optimized geometries of predominant conformers for compound (3*S*, 4*R*, 6S, 7*S*, 8*R*, 9*S*, 16*S*, 17*R*, 18*S*, 21*R*)-**4** at the B3LYP/6-31G(d,p) level in the gas phase.

Table S6. Cartesian coordinates for the low-energy reoptimized MMFF conformers of (3*S*, 4*R*, 6S, 7*S*, 8*R*, 9*S*, 16*S*, 17*R*, 18*S*, 21*R*)-**4** at B3LYP/6-31G (d, p) level of theory in MeOH.

**4a**				**4b**				**4c**			
Symbol	X	Y	Z	Symbol	X	Y	Z	Symbol	X	Y	Z
C	-2.4592	2.1690	-1.1898	C	-2.5304	3.0178	-1.0094	C	-2.5307	2.3597	-1.0994
C	-1.7193	0.8442	-0.8819	C	-2.0816	1.5454	-0.8187	C	-1.8567	0.9958	-0.8107
C	-0.4193	1.0404	-0.0454	C	-0.7025	1.4068	-0.1151	C	-0.5118	1.1182	-0.0362
C	-0.5698	2.1959	1.0003	C	-0.5185	2.5060	0.9812	C	-0.5486	2.2847	1.0068
C	-0.7547	3.5324	0.2806	C	-0.4471	3.8842	0.3149	C	-0.6834	3.6252	0.2831
C	-1.9802	3.4723	-0.6461	C	-1.7409	4.1583	-0.4565	C	-1.9504	3.6342	-0.5879
C	0.4589	2.1538	2.1218	C	0.5558	2.1918	2.0137	C	0.5198	2.1894	2.0863
C	-0.1045	-0.2046	0.8426	C	-0.5724	0.0676	0.6708	C	-0.2357	-0.1444	0.8400
C	1.3350	-0.7015	0.9737	C	0.7637	-0.6700	0.6542	C	1.1801	-0.7152	0.9252
C	-3.4612	-3.9929	-6.1135	C	-2.7261	-5.6818	-3.5086	C	-2.4173	-6.1155	-3.8309
C	-2.6066	-4.6007	-5.1974	C	-1.5770	-5.2402	-2.8603	C	-3.3869	-5.1346	-4.0152
C	-2.3536	-3.9667	-3.9819	C	-1.2967	-3.8747	-2.8335	C	-3.0299	-3.7934	-3.8729
C	-2.9261	-2.7296	-3.6448	C	-2.1349	-2.9235	-3.4312	C	-1.7241	-3.3955	-3.5491
C	-3.8022	-2.1547	-4.5721	C	-3.2718	-3.3961	-4.0959	C	-0.7638	-4.3992	-3.3882
C	-4.0629	-2.7736	-5.7966	C	-3.5689	-4.7589	-4.1321	C	-1.1061	-5.7464	-3.5215
C	-2.6564	-1.9437	-2.3779	C	-1.9145	-1.4200	-3.4111	C	-1.2741	-1.9599	-3.3637
C	-1.2468	-1.4231	-2.1156	C	-2.2375	-0.6919	-2.1155	C	-1.6412	-1.2783	-2.0560
O	-1.0118	-0.7143	1.4742	O	-1.5097	-0.3158	1.3447	O	-1.1487	-0.6120	1.4958
C	0.6446	1.2318	-1.1543	C	0.2758	1.4321	-1.3149	C	0.5132	1.2291	-1.1941
N	0.1573	0.6300	-2.2661	N	-0.4381	1.0316	-2.4002	N	-0.0632	0.6592	-2.2766
C	-1.2081	0.1297	-2.1707	C	-1.8579	0.7999	-2.1677	C	-1.4522	0.2473	-2.1162
H	-2.4246	0.2004	-0.3514	H	-2.8550	1.0515	-0.2211	H	-2.5713	0.4045	-0.2301
O	1.7378	1.7907	-1.0823	O	1.4687	1.7169	-1.3178	O	1.6494	1.7022	-1.1661
C	1.7891	2.1450	1.9949	C	1.8524	1.9502	1.7865	C	1.8408	2.1007	1.9065
C	2.7429	1.8133	3.1065	C	2.7823	1.3474	2.8020	C	2.8159	1.7235	2.9842
C	3.6987	0.6096	2.8240	C	3.4425	-0.0099	2.3886	C	3.6814	0.4579	2.6833
C	3.1328	-0.8611	2.8939	C	2.5909	-1.3375	2.4233	C	3.0278	-0.9734	2.7906

C	1.6776	-1.1503	2.4165	C	1.0881	-1.3138	2.0174	C	1.5451	-1.1843	2.3554
C	4.5310	0.8363	1.5459	C	4.2231	0.1272	1.0655	C	4.4801	0.6185	1.3740
C	-3.3811	2.1288	-2.4010	C	-3.5232	3.2492	-2.1409	C	-3.4980	2.3709	-2.2758
C	-2.2967	4.7959	-1.3125	C	-1.8133	5.5613	-1.0253	C	-2.2156	4.9698	-1.2526
O	-0.9693	4.5326	1.2845	O	-0.3175	4.9170	1.2889	O	-0.7895	4.6445	1.2848
C	1.3308	-2.6294	2.5417	C	0.4905	-2.7180	2.0305	C	1.1326	-2.6455	2.4896
C	2.9252	-2.9242	4.3683	C	2.0522	-3.4258	3.7679	C	2.7363	-3.0048	4.2967
C	3.3309	-1.4503	4.3201	C	2.7239	-2.0480	3.7991	C	3.2278	-1.5577	4.2186
O	0.5986	-3.2427	1.8136	O	-0.3386	-3.1336	1.2718	O	0.3645	-3.2283	1.7732
O	3.4619	-3.7201	5.1007	O	2.4726	-4.3487	4.4220	O	3.2342	-3.8176	5.0376
C	2.5541	-0.7402	5.4512	C	2.1258	-1.2888	5.0056	C	2.5291	-0.7903	5.3632
H	0.9802	-0.6447	3.0972	H	0.5261	-0.7662	2.7856	H	0.8955	-0.6468	3.0587
H	3.8021	-1.4584	2.2602	H	3.1013	-2.0141	1.7239	H	3.6435	-1.6177	2.1488
O	-3.5989	-1.6066	-1.6794	O	-1.6490	-0.8329	-4.4435	O	-0.5285	-1.4512	-4.1845
O	4.7257	-1.4038	4.5981	O	4.1066	-2.2742	4.0329	O	4.6306	-1.5942	4.4571
O	-3.0944	2.7743	-0.0369	O	-2.9244	3.6734	0.2143	O	-3.0781	3.0094	0.0739
O	1.8835	-3.3588	3.5955	O	0.9228	-3.6070	3.0220	O	1.6650	-3.3941	3.5397
N	-1.5187	-4.6708	-3.0078	N	-0.0468	-3.4451	-2.1961	N	-4.0561	-2.7804	-4.1207
O	-1.5248	-4.2563	-1.8428	O	0.2641	-2.2520	-2.3149	O	-3.7039	-1.5935	-4.1324
O	-0.8665	-5.6436	-3.3830	O	0.6252	-4.2757	-1.5980	O	-5.2136	-3.1464	-4.3136
H	-1.5323	1.9924	1.4776	H	-1.4633	2.5022	1.5305	H	-1.5012	2.1424	1.5249
H	0.1374	3.7750	-0.3089	H	0.3975	3.9235	-0.3890	H	0.1946	3.8073	-0.3482
H	0.0342	2.0011	3.1147	H	0.1740	2.0296	3.0232	H	0.1275	2.0720	3.0973
H	2.0231	0.0619	0.6287	H	1.5498	0.0066	0.3406	H	1.8942	0.0117	0.5563
H	1.4391	-1.5511	0.2865	H	0.7002	-1.4403	-0.1245	H	1.2180	-1.5684	0.2356
H	-3.6673	-4.4790	-7.0612	H	-2.9557	-6.7423	-3.5358	H	-2.6831	-7.1617	-3.9392
H	-2.1520	-5.5625	-5.3991	H	-0.8890	-5.9269	-2.3826	H	-4.4063	-5.3881	-4.2776
H	-4.2696	-1.2052	-4.3326	H	-3.9267	-2.6823	-4.5867	H	0.2572	-4.1197	-3.1481
H	-4.7408	-2.3020	-6.5011	H	-4.4599	-5.0998	-4.6512	H	-0.3442	-6.5076	-3.3863
H	-0.9270	-1.7796	-1.1329	H	-3.3254	-0.7779	-1.9663	H	-2.6768	-1.5150	-1.7944
H	-0.5458	-1.8157	-2.8571	H	-1.7835	-1.2128	-1.2690	H	-1.0182	-1.7351	-1.2740
H	0.7040	0.5892	-3.1177	H	0.0088	0.8330	-3.2858	H	0.4429	0.4946	-3.1374
H	-1.7569	0.4490	-3.0571	H	-2.4232	1.2473	-2.9854	H	-2.0228	0.6003	-2.9754
H	2.2202	2.3037	1.0101	H	2.2442	2.0998	0.7826	H	2.2402	2.2235	0.9032
H	2.1849	1.6545	4.0356	H	2.2607	1.2463	3.7604	H	2.2869	1.6127	3.9369
H	3.4071	2.6715	3.2883	H	3.6242	2.0334	2.9800	H	3.5399	2.5397	3.1264
H	4.4267	0.6418	3.6407	H	4.2090	-0.1839	3.1509	H	4.4384	0.4509	3.4739
H	5.0044	1.8248	1.5756	H	4.8948	0.9930	1.1112	H	5.0174	1.5743	1.3764
H	3.9443	0.7838	0.6236	H	3.5847	0.2587	0.1866	H	3.8584	0.5968	0.4737
H	5.3286	0.0886	1.4735	H	4.8427	-0.7606	0.8978	H	5.2245	-0.1801	1.2814
H	-3.9068	1.1684	-2.4422	H	-4.2613	2.4386	-2.1735	H	-4.0651	1.4340	-2.3165
H	-4.1351	2.9129	-2.3219	H	-4.0699	4.1773	-1.9711	H	-4.2160	3.1837	-2.1582
H	-2.8413	2.2681	-3.3436	H	-3.0374	3.3084	-3.1211	H	-2.9862	2.5020	-3.2350

H	-1.4488	5.1179	-1.9290	H	-0.9815	5.7284	-1.7203	H	-1.3784	5.2350	-1.9094
H	-3.1815	4.7449	-1.9477	H	-2.7493	5.7488	-1.5530	H	-3.1293	4.9677	-1.8476
H	-2.4689	5.5596	-0.5478	H	-1.7185	6.2838	-0.2108	H	-2.3072	5.7483	-0.4888
H	-0.5708	5.3599	0.9762	H	0.4812	4.7083	1.8011	H	-0.3709	5.4480	0.9418
H	2.9242	0.2813	5.5582	H	2.6836	-0.3633	5.1633	H	2.9623	0.2083	5.4466
H	2.7336	-1.2658	6.3945	H	2.2362	-1.9063	5.9028	H	2.7066	-1.3162	6.3067
H	1.4735	-0.7125	5.2802	H	1.0634	-1.0511	4.8871	H	1.4470	-0.7000	5.2263
H	4.8915	-2.0717	5.2880	H	4.1570	-3.0865	4.5704	H	4.7736	-2.2530	5.1606

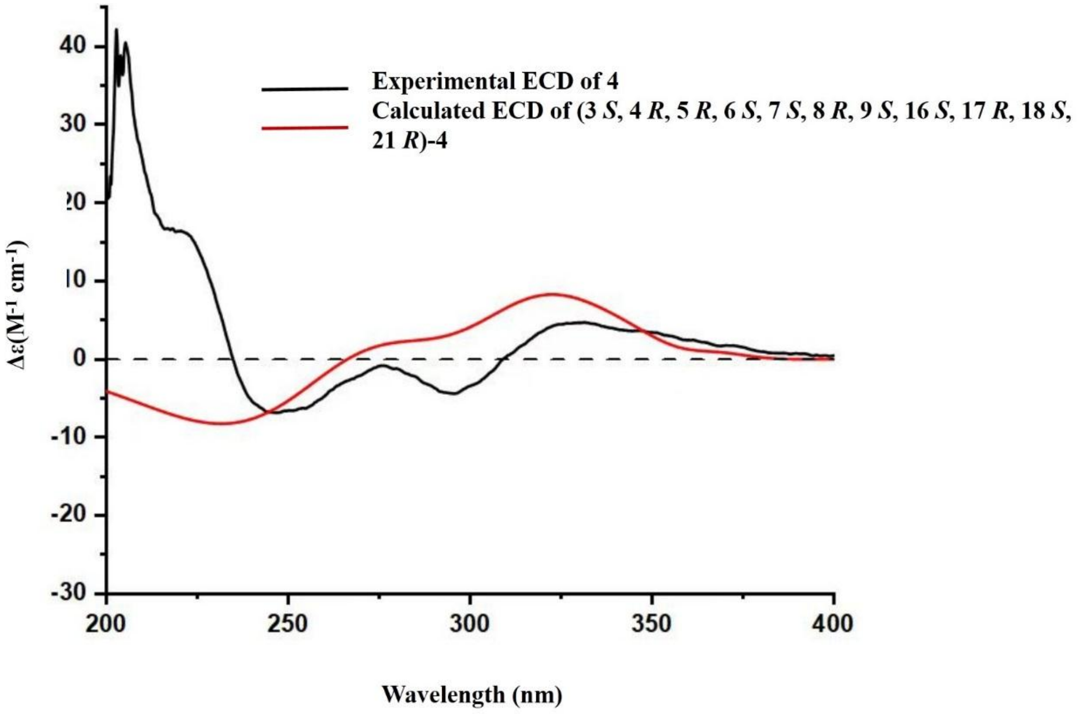

Figure S107. Experimental and calculated ECD spectra of Compound **4**

Table S7. Gibbs free energies[a] and equilibrium populations[b] of low-energy conformers of (3*S*, 4*R*, 5*R*, 6*S*, 7*S*, 8*R*, 9*S*, 16*S*, 20*S*)-**11**.

Conformers	In MeOH	
	Δ*G (Ha)*	*P* (%)/100
**11a**	-1988.753079	33.5
**11b**	-1988.751970	33.4
**11c**	-1988.746997	33.1

[a]B3LYP/6-31+G(d,p), in kcal/mol. [b]From Δ*G* values at 298.15K.

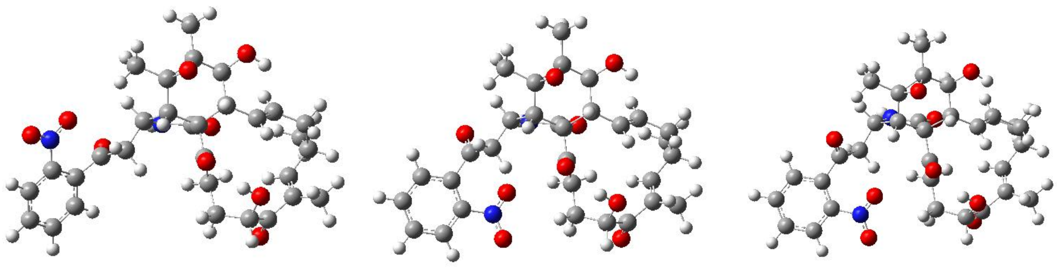

11a **11b** **11c**

Figure S108. Optimized geometries of predominant conformers for compound (3*S*, 4*R*, 5*R*, 6*S*, 7*S*, 8*R*, 9*S*, 16*S*, 20*S*)-**11** at the B3LYP/6-31G(d,p) level in the gas phase.

Table S8. Cartesian coordinates for the low-energy reoptimized MMFF conformers of (3*S*, 4*R*, 5*R*, 6*S*, 7*S*, 8*R*, 9*S*, 16*S*, 20*S*)-**11** at B3LYP/6-31G (d, p) level of theory in MeOH.

**11a**				**11b**				**11c**			
Symbol	X	Y	Z	Symbol	X	Y	Z	Symbol	X	Y	Z
C	-1.5760	2.2623	-1.8412	C	-1.8989	2.5863	-1.6267	C	-1.3917	2.5312	-2.0109
C	-1.2914	0.8464	-1.2994	C	-1.6152	1.1175	-1.2513	C	-1.0745	1.0705	-1.6091
C	-0.3851	0.7790	-0.0227	C	-0.6028	0.9054	-0.0754	C	-0.2649	0.9171	-0.2726
C	-0.4395	2.0904	0.8297	C	-0.5679	2.1177	0.9128	C	-0.4912	2.1077	0.7193
C	-0.0243	3.2981	-0.0289	C	-0.1940	3.4058	0.1577	C	-0.0915	3.4355	0.0595
C	-0.9597	3.4640	-1.2252	C	-1.2087	3.7090	-0.9431	C	-0.9177	3.6831	-1.1999
C	0.2635	2.0245	2.1775	C	0.2292	1.9067	2.1919	C	0.0897	1.9090	2.1117
C	-0.8784	-0.3664	0.9068	C	-1.0265	-0.3348	0.7664	C	-0.7279	-0.3545	0.4964
C	0.0850	-1.4722	1.2961	C	-0.0210	-1.4403	1.0110	C	0.2780	-1.4484	0.7999
C	-2.3228	-5.8605	-5.0315	C	-2.4297	-5.8521	-4.5464	C	-2.5291	-5.4762	-4.9925
C	-2.8383	-4.6281	-5.4216	C	-1.6779	-5.6368	-3.3950	C	-2.5637	-5.2056	-3.6279
C	-2.2242	-3.4614	-4.9676	C	-1.1752	-4.3608	-3.1449	C	-2.3431	-3.9014	-3.1862
C	-1.1028	-3.4831	-4.1265	C	-1.4076	-3.2779	-4.0040	C	-2.0753	-2.8448	-4.0690
C	-0.5891	-4.7350	-3.7699	C	-2.1496	-3.5264	-5.1641	C	-2.0689	-3.1422	-5.4376
C	-1.1945	-5.9124	-4.2100	C	-2.6587	-4.7974	-5.4331	C	-2.2883	-4.4406	-5.8975
O	0.7593	-2.0458	-3.8937	O	-0.2060	-1.3184	-4.5748	O	-2.4445	-0.5172	-4.1820
C	-0.3955	-2.2606	-3.5729	C	-0.9448	-1.8513	-3.7684	C	-1.7631	-1.4022	-3.7012
C	-1.1045	-1.4591	-2.4898	C	-1.5989	-1.0765	-2.6309	C	-0.4777	-1.1309	-2.9330
C	1.0170	0.4644	-0.5968	C	0.7363	0.6512	-0.8051	C	1.1964	0.8039	-0.7598
N	0.8345	-0.0072	-1.8601	N	0.4280	0.2829	-2.0806	N	1.1551	0.5238	-2.0969
C	-0.5349	-0.0346	-2.3450	C	-0.9805	0.3192	-2.4352	C	-0.1737	0.3608	-2.6725
H	-2.2723	0.4149	-1.0732	H	-2.5849	0.6893	-0.9751	H	-2.0398	0.5631	-1.5237
O	2.0950	0.5749	-0.0293	O	1.8645	0.7336	-0.3430	O	2.2197	0.9264	-0.1033
C	1.5107	2.4128	2.4769	C	1.5035	2.2438	2.4351	C	1.2679	2.3379	2.5849
C	2.0614	2.4112	3.8808	C	2.1477	2.1081	3.7922	C	1.6852	2.1775	4.0254
C	3.0969	1.2888	4.2050	C	3.1774	0.9461	3.9492	C	2.7769	1.0968	4.3036
C	2.4994	-0.0849	3.9894	C	2.5399	-0.3968	3.6618	C	2.3211	-0.2741	3.8515
C	1.5554	-0.6931	4.7273	C	1.6247	-1.0450	4.4010	C	1.3801	-1.0524	4.4119

C	1.0394	-1.9923	4.1751	C	1.0599	-2.2984	3.7891	C	1.0319	-2.3022	3.6493
C	-0.4425	-2.1017	3.7409	C	-0.4338	-2.3489	3.3921	C	-0.3951	-2.4729	3.0744
C	-0.4801	-2.5257	2.2600	C	-0.5265	-2.6050	1.8757	C	-0.2792	-2.6686	1.5497
C	4.3938	1.4482	3.3964	C	4.4207	1.1539	3.0706	C	4.1243	1.4585	3.6596
C	0.9591	-0.1786	6.0149	C	1.1078	-0.6244	5.7551	C	0.6406	-0.7720	5.6973
C	-2.1066	2.3059	-3.2709	C	-2.5382	2.7847	-2.9968	C	-1.7812	2.7326	-3.4717
C	-0.7527	4.7552	-1.9900	C	-1.0231	5.0682	-1.5861	C	-0.7274	5.0678	-1.7858
O	1.7689	-2.9489	3.9703	O	1.7604	-3.2581	3.5140	O	1.8658	-3.1546	3.3887
O	-0.1109	4.5088	0.7149	O	-0.2059	4.5325	1.0281	O	-0.3435	4.5362	0.9278
O	-2.0314	-0.3369	1.3109	O	-2.1625	-0.3677	1.2185	O	-1.8887	-0.4098	0.8683
O	-1.1386	-0.8879	3.9811	O	-1.1106	-1.1628	3.7854	O	-1.2279	-1.3813	3.4320
O	-2.3283	3.0982	-0.9385	O	-2.5620	3.3415	-0.5925	O	-2.2782	3.2093	-1.0975
N	-2.7556	-2.1781	-5.4479	N	-0.3217	-4.1813	-1.9649	N	-2.4708	-3.6482	-1.7444
O	-2.1069	-1.1589	-5.1841	O	0.3850	-3.1666	-1.9340	O	-2.6164	-2.4762	-1.3858
O	-3.8024	-2.1842	-6.0872	O	-0.3486	-5.0376	-1.0869	O	-2.4336	-4.6094	-0.9806
H	-1.5015	2.2355	1.0474	H	-1.6082	2.2506	1.2242	H	-1.5762	2.1551	0.8472
H	1.0023	3.1616	-0.4079	H	0.7998	3.2990	-0.3069	H	0.9743	3.4179	-0.2228
H	-0.3605	1.6584	2.9931	H	-0.3440	1.4746	3.0125	H	-0.5728	1.3900	2.8046
H	0.4118	-1.9518	0.3621	H	0.2903	-1.8221	0.0295	H	0.7202	-1.7629	-0.1556
H	0.9950	-0.9993	1.6824	H	0.8853	-0.9779	1.4191	H	1.1133	-0.9787	1.3334
H	-2.7923	-6.7752	-5.3795	H	-2.8220	-6.8421	-4.7564	H	-2.7050	-6.4873	-5.3461
H	-3.6954	-4.5493	-6.0790	H	-1.4590	-6.4355	-2.6967	H	-2.7742	-5.9777	-2.8980
H	0.2943	-4.7774	-3.1401	H	-2.3258	-2.7086	-5.8560	H	-1.8880	-2.3362	-6.1419
H	-0.7794	-6.8716	-3.9151	H	-3.2328	-4.9642	-6.3397	H	-2.2755	-4.6412	-6.9647
H	-2.1777	-1.4161	-2.6953	H	-2.6614	-0.9644	-2.8988	H	-0.4777	-1.7051	-2.0041
H	-0.9909	-2.0230	-1.5518	H	-1.5836	-1.6632	-1.7082	H	0.3383	-1.5537	-3.5405
H	1.5955	-0.3843	-2.4101	H	1.1431	-0.0079	-2.7343	H	2.0144	0.3438	-2.6002
H	-0.5694	0.4162	-3.3383	H	-1.0884	0.8506	-3.3818	H	-0.2253	0.8793	-3.6306
H	2.1803	2.7429	1.6840	H	2.1234	2.6347	1.6296	H	1.9774	2.8229	1.9157
H	1.2280	2.3254	4.5869	H	1.3615	1.9750	4.5434	H	0.8004	1.9338	4.6238
H	2.5483	3.3775	4.0799	H	2.6674	3.0459	4.0395	H	2.0680	3.1384	4.4005
H	3.3428	1.4110	5.2695	H	3.4966	0.9742	5.0010	H	2.9136	1.0885	5.3943
H	2.8364	-0.5979	3.0890	H	2.8214	-0.8468	2.7101	H	2.7750	-0.6282	2.9263
H	-0.8840	-2.9186	4.3361	H	-0.8626	-3.2231	3.9095	H	-0.7934	-3.4091	3.5000
H	0.1093	-3.4427	2.1770	H	0.0630	-3.4988	1.6549	H	0.3929	-3.5168	1.3928
H	-1.5135	-2.7659	1.9828	H	-1.5690	-2.8287	1.6186	H	-1.2578	-2.9425	1.1394
H	5.1352	0.6994	3.6973	H	5.1640	0.3702	3.2556	H	4.8956	0.7298	3.9337
H	4.8352	2.4399	3.5500	H	4.8929	2.1217	3.2769	H	4.4648	2.4485	3.9855
H	4.2071	1.3192	2.3240	H	4.1607	1.1205	2.0061	H	4.0481	1.4663	2.5659
H	-0.1000	0.0646	5.8861	H	0.0467	-0.3615	5.7038	H	-0.4238	-0.6071	5.5057
H	1.0257	-0.9434	6.8001	H	1.2051	-1.4491	6.4737	H	0.7209	-1.6290	6.3793
H	1.4851	0.7094	6.3755	H	1.6659	0.2271	6.1540	H	1.0482	0.1003	6.2155
H	-1.3068	2.3360	-4.0189	H	-1.8036	2.8235	-3.8084	H	-0.9092	2.8965	-4.1166

H	-2.7314	1.4312	-3.4858	H	-3.2430	1.9724	-3.2134	H	-2.3269	1.8637	-3.8493
H	-2.7288	3.1930	-3.3990	H	-3.1058	3.7163	-3.0033	H	-2.4317	3.6046	-3.5539
H	0.2789	4.8140	-2.3576	H	-0.0200	5.1440	-2.0229	H	0.3289	5.2303	-2.0330
H	-1.4300	4.8414	-2.8409	H	-1.7583	5.2582	-2.3695	H	-1.3222	5.2199	-2.6873
H	-0.9168	5.6021	-1.3196	H	-1.1134	5.8433	-0.8215	H	-1.0142	5.8155	-1.0426
H	0.3047	4.3316	1.5756	H	0.2691	4.2609	1.8317	H	-0.0089	4.2764	1.8026
H	-1.8049	-0.7814	3.2791	H	-1.8123	-0.9927	3.1320	H	-1.8050	-1.1840	2.6717

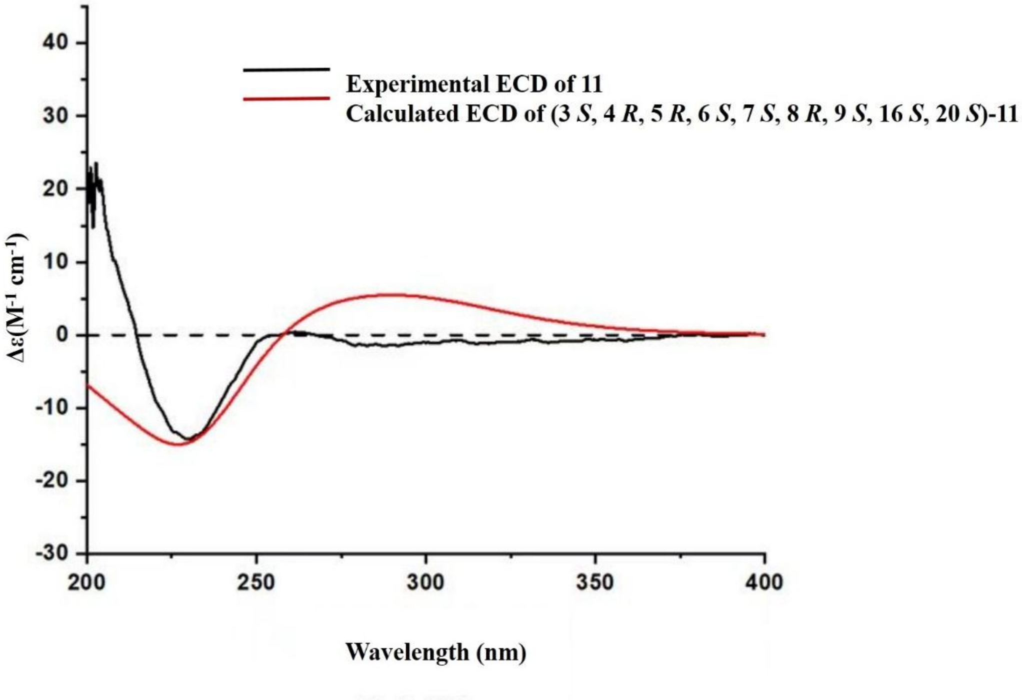

Figure S109. Experimental and calculated ECD spectra of Compound **11**
